# Anisotropic NMR as a Crucial Tool for Differentiation of Epimers With High Conformational Flexibility

**DOI:** 10.1002/anie.2111490

**Published:** 2026-05-25

**Authors:** Juan Carlos C. Fuentes‐Monteverde, Abel M. Forero, Nilamoni Nath, Antonio Hernández Daranas, Carlos Jiménez, Jaime Rodríguez, Christian Griesinger

**Affiliations:** ^1^ NMR Based Structural Biology MPI for Multidisciplinary Sciences Göttingen Germany; ^2^ Facultade de Ciencias CICA‐Centro Interdisciplinar de Química e Bioloxía e Departamento de Química Universidade Da Coruña A Coruña Spain; ^3^ Department of Chemistry Gauhati University Guwahati India; ^4^ Instituto de Productos Naturales y Agrobiología, Consejo Superior de Investigaciones Cientificas (IPNA‐CSIC) San Cristobal de La Laguna Spain

**Keywords:** circular dichroism, configuration determination, conformational flexibility, meroditerpene, RCSA/RDC

## Abstract

Identifying the correct structures of epimers is highly challenging due to the sometimes very subtle differences in their respective NMR signatures. In this context, the analysis of carbon residual chemical shift anisotropies (^13^C‐RCSAs) and residual dipolar couplings (RDCs) was essential for distinguishing the relative configurations of two tetraprenyltoluquinol epimeric meroterpenoids at C3 (**1a** and **1b**). These compounds feature two remotely located stereoclusters and exhibit significant conformational flexibility. DP4‐based approaches (DP4+ or *J*‐DP4) failed to differentiate these epimers. A geometric exploration strategy was developed to investigate the conformational diversity of **1a** and **1b** while accounting for their inherent flexibility. Then, refining the conformational space and optimizing the fitting of the anisotropic parameters of **1a**/**1b** measured in a 3 mm semi‐micro compression device using poly‐HEMA gels in DMSO enabled us to discriminate the relative configuration of them.

## Graphical abstract

Epimer discrimination remains challenging due to subtle NMR differences. Here, we propose a methodology based on ^13^C‐RCSA and RDC anisotropic parameters, enabling the assignment of two flexible tetraprenyltoluquinol epimers (**1a** and **1b**) with remote stereoclusters. Conventional DP4 methods failed, but a geometric conformational exploration combined with anisotropic data fitting allowed unambiguous configuration determination.

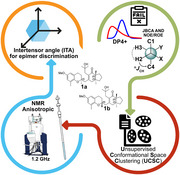

## 1 Introduction

The distinction of epimers, especially molecules exhibiting extensive conformational dynamics, is a formidable challenge in natural products structure determination. The intricate task of epimer differentiation, owing to their closely resembling characteristics, has been addressed using sophisticated techniques including computer‐assisted structure elucidation (CASE) [1], IR spectroscopy [2], GC–MS [3], NMR data analysis, Raman optical activity [4], chiral‐DOSY [5], and NOE analysis [6]. The differentiation between epimers has mainly relied on experimental NMR parameters, namely ^1^H and ^13^C chemical shifts, along with Nuclear Overhauser Effect (NOE) measurements and proton‐proton coupling constants that can be compared to their calculated counterparts from quantum mechanical calculations. Prior to the advent of the DP4 methodology by Goodman et al. [7], modest evaluation metrics like the squared linear correlation coefficient (*R* ^2^), mean absolute error (MAE), and the root mean square deviation (RMSD) served as the primary tools for correlating experimental NMR chemical shifts with their calculated counterparts in the molecular assignments. However, these correlation tools often fail to provide a definitive assignment when the NMR chemical shifts or coupling constants between the two epimers are remarkably similar. The introduction of Bayesian probabilistic approaches comparing experimental NMR data with DFT calculated values, such as CP3 [8], DP4 [7], DP4+ [9], *J*‐DP4 [10], and MIX‐*J*‐DP4 [11], among others [12, 13], represented a very significant advance in the correct assignment and distinction of diastereomeric structures. However, these methodologies should be used with special care when the compounds have a high degree of conformational freedom. More than 178 articles have been published in the last decade on the application of Bayesian methods to the assignment of natural products, 14 of which implied the structural revision or reassignment of chemical shifts of previously reported molecules. Notably, just 7 publications have dealt with epimer differentiation, corresponding to molecules whose conformations were well defined [14, 15, 16, 17].

In 2015, we reported the isolation of an epimeric mixture of two chromane meroditerpenes (**1a** and **1b**) from the brown macroalga *Sargassum muticum* (Yendo) Fensholt (SM), as well as its photodamage attenuation effect [18]. Later on, we purified **1b** from that mixture as a single epimer and corrected its structure by applying NOE methodologies along with anisotropic NMR techniques [19]. In the present study, we isolated the second epimer **1a**; however, when we applied DP4 approaches (DP4+ and i*J*‐DP4) to perform structural epimeric differentiation, it could not be distinguished from **1b**. Similar limitations have been reported during the structural elucidation of hyacinthacine alkaloids, where a careful evaluation of the conformational landscape was shown to be critical for accurate computational structure determination, particularly in systems involving intramolecular hydrogen bonding [20, 21]. Accordingly, for epimers such as **1a** and **1b**, an accurate and exhaustive description of the conformational space is a prerequisite for successful epimeric discrimination.

**SCHEME 1:**
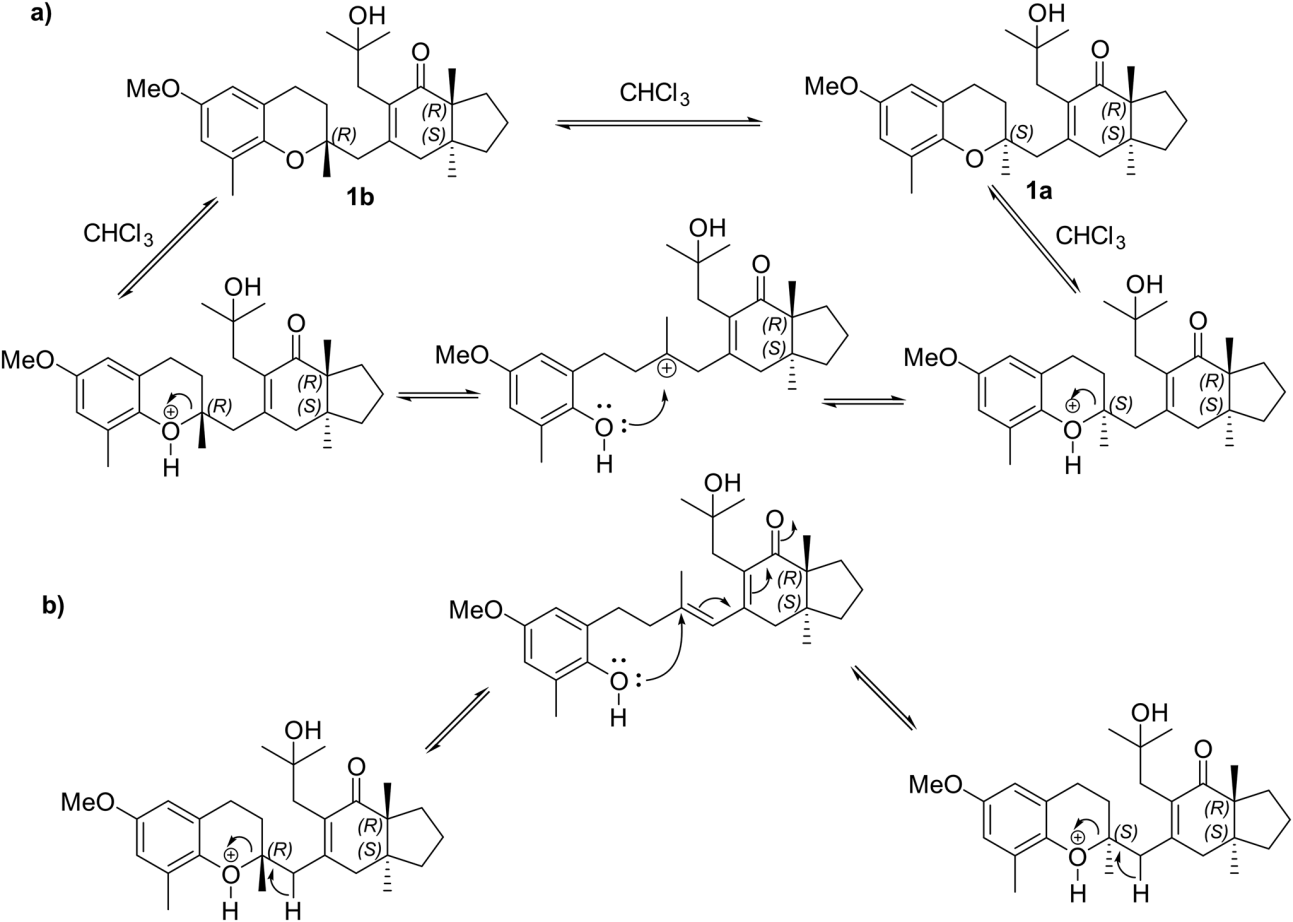
Proposed mechanisms for the epimerization at C3 of **1a** into **1b** in CHCl_3_.

This result demonstrated the drawbacks of the DP4 methodologies for differentiating conformationally flexible epimers such as **1a**/**1b** as shown in Scheme 1. To determine if this limitation also occurs in other epimers, we carried out a comprehensive analysis of NMR data, named exploratory data analysis (EDA), on selected literature reported epimeric pairs with the abovementioned conformational freedom. Furthermore, this analysis suggests a factor that could be used as a threshold at which the DP4 methodology begins to give unreliable results. Then, to overcome the limitation of the DP4 methodologies, we found that the use of anisotropic NMR parameters for **1a**/**1b**, including RDC [22] and ^13^C‐RCSA [23], allowed us to clearly differentiate the two epimers. The epimeric discrimination was achieved through a conformer selection process based on a dihedral principal components analysis (d‐PCA), in which a series of structural parameters, such as radius of gyration and number of rotatable bonds, were considered.

## 2 Results and Discussion

### 2.1 Epimeric Relationship Between **1a** and **1b**

Continuing the analysis of the meroditerpenes from the algae *Sargassum muticum* collected on the Galician coast in Spain, we isolated **1a** as a pure compound by RP‐HPLC. The identical structural constitution of **1a** and **1b**, previously isolated from the same marine organism [18], was confirmed from a 15‐µg sample using 1D NMR experiments complemented by a suite of ultra‐sensitive 2D NMR experiments. The implementation of optimal control pulses (OCP) in both HSQC and HMBC experiments played a pivotal role in the sensitivity reached (Figures S8–S11). By its implementation, OCP‐sequences reduced the experimental time by approximately 5.6‐fold [24].

Next, the relative configuration of **1a** was addressed. The same *trans* fused ring configuration in the hydrindane skeleton in **1a**, as in **1b**, was suggested by the absence of ROESY correlations between H18 and H19 methyl groups (see SI, Figure S18). To compare all proton chemical shifts of **1a** and **1b**, a PSYCHE pure shift NMR experiment [25] was measured at 800 MHz (see Figures S19 and S20), showing that they must differ in the configuration of the stereogenic center at C3. Indeed, the most relevant differences were found in the chemical shifts of protons H_2_4 (Δ*δ*_H_: 0.179), one of diastereotopic protons of H_2_14 (Δ*δ*_H_: 0.122), and Me19 (Δ*δ*_H_: 0.116) (see Tables S1 and S2; Δ*δ*_H_ average difference: 0.0417 (DMSO)/0.0625 (DCM) ppm; Δ*δ*_C_ average difference: 0.296 (DMSO)/0.298 (DCM) ppm). Moreover, we observed a rapid interconversion between **1a** and **1b** when the NMR experiments were performed in CDCl_3_. This process is probably due to the acidic nature of CDCl_3_. Opening of the pyran ring in **1b** by protonation of the oxygen at C3 followed by the subsequent cyclization would lead to the epimerization of this stereogenic center. This process was also observed in similar compounds (Scheme 1a) [26, 27]. Nevertheless, other interconversion processes are not discarded, such as an E2 elimination to give a keto‐diene intermediate, which later could act as a Michael acceptor, as is shown in (Scheme 1b). To mitigate this epimerization, and after trying different deuterated solvents, we found that this process is slowed in DCM, 1,1,2,2‐tetrachloroethane (TCE), or DMSO (Figure S15) [28]. For this reason, all the ^1^H and ^13^C chemical shifts at 1.2 GHz of **1a** and **1b** were reassigned in DMSO‐*d_6_* (Table S1).

### 2.2 Conformational Analysis of **1a**

**FIGURE 1:**
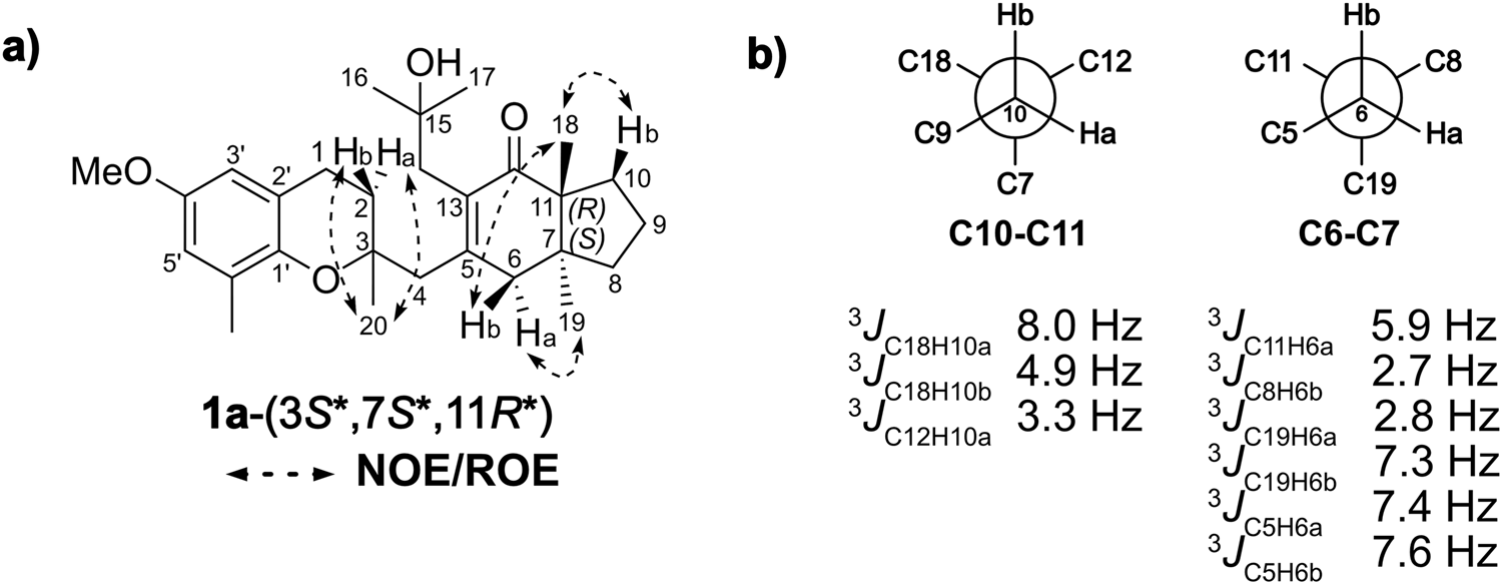
(a) Main NOE/ROE correlations in the chromane (H_3_20/H2a, strong; H_3_20/H2b, weak). (b) Newman projections of the ^n^ *J*_CH_ values involved in the C6–C7 and C10–C11 bonds in the hydrindane moiety in **1a**.

A comprehensive conformational analysis was carried out to determine the relative configuration of **1a**. The *trans*‐fused ring configuration in the hydrindane skeleton in **1a** was confirmed by NOE/ROE measurements and by *J*‐based conformational analysis (*J*‐BCA) around the C6–C7 and C10–C11 bonds measured using an IPAP‐HSQMBC‐COSY [29, 30] experiment (see Figure 1, Table S6, and Figures S21 and S22).

**FIGURE 2:**
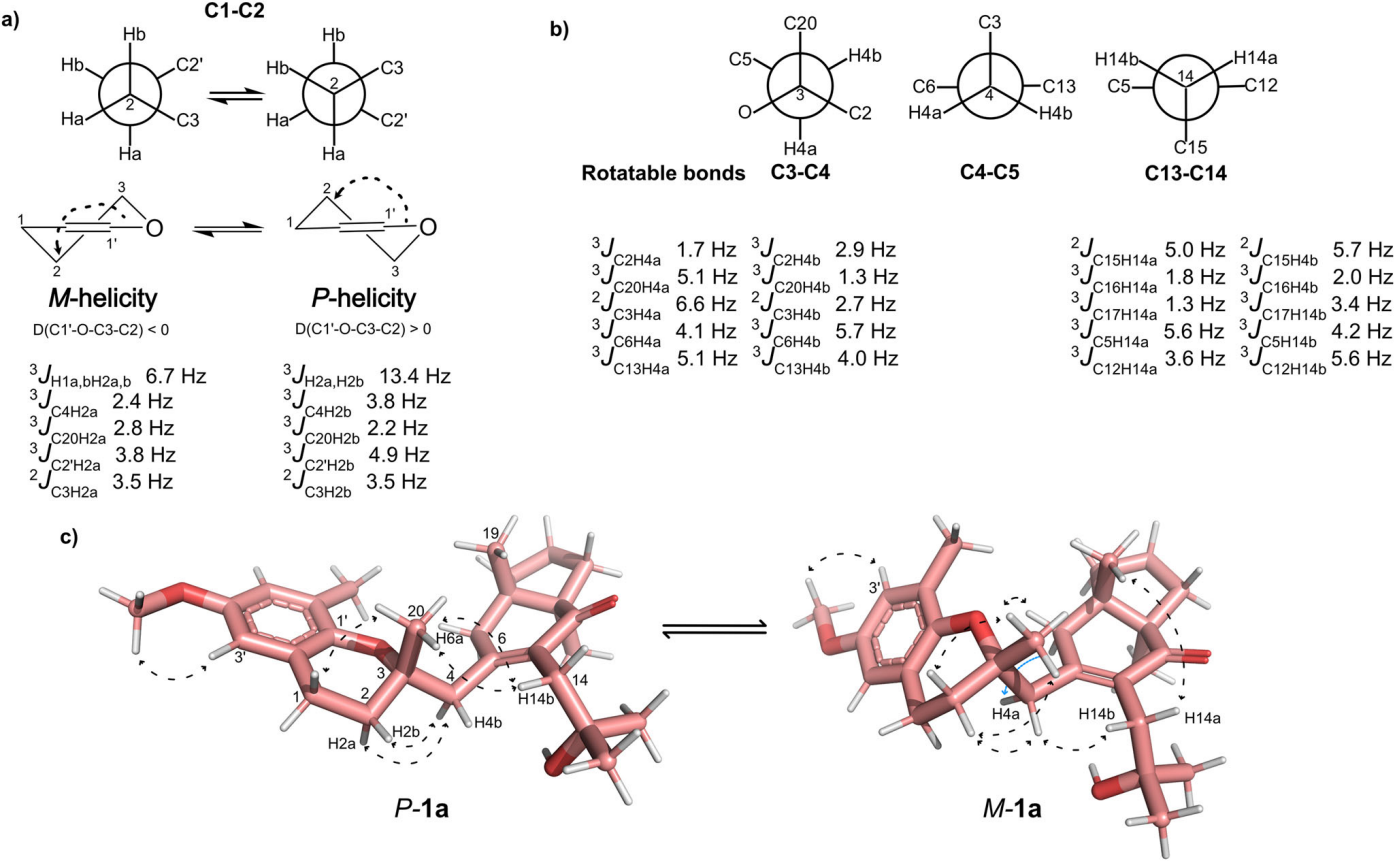
(a) Newman projections and key ^n^ *J*_CH_ values around the C1–C2 bond in the two *M*‐ and *P*‐helicities in the chromane fragment of **1a**. (b) Newman projections and key ^n^ *J*_CH_ values around the rotatable bonds C3–C4, C4–C5, and C13–C14 of **1a**. (c) ROE/NOE correlations and conformational equilibrium between the major conformers of the *M*‐ and *P*‐helices in **1a**. (A similar study based on *J*‐BCA/ROESY of **1b** is shown in the Supporting Information.)

The presence of an equilibrium in **1a** between two major sets of conformers, corresponding to two possible helicities in the pyran ring (see Figure 2), namely *P‐* (when D(C1’–O–C3–C2) > 0) and *M*‐ (when D(C1’–O–C3–C2) < 0), was deduced from the isochronous proton chemical shift at *δ*_H_ 2.7131 (see Table S1) for the two methylene protons at C1. Also, the coupling constants observed between protons H1 and H2, ^3^ *J*_H1H2a_ = ^3^ *J*_H1H2b_ = 6.7 Hz, support this conclusion.

Additionally, the medium‐range ^3^ *J*_C2’H2a_ = 3.8 Hz and ^3^ *J*_C2’H2b_ = 4.9 Hz and ^2^ *J*_C3H2a/b_ = 3.5 Hz values between H2a/b and C2’ and between H2a/b and C3, respectively, were measured from the non‐sign‐sensitive IPAP‐HSQMBC experiment [31] (Figure 2). Instead of the expected large and small ones, they were consistent with the presence of an equilibrium. This equilibrium between two helicities was further corroborated by the ROE correlations observed between H2b/H_3_20, H2a/H_3_20, and H1/H_3_20 protons after the assignment of the H2a/H2b protons using the previously mentioned ^1^H PSYCHE pure shift NMR experiment [32]. Using the *J*‐BCA methodology, the equilibrium populations were estimated to be approximately 3:2 for the *P*‐ and *M*‐helicities.

This complex conformational equilibrium could explain the lack of discrimination when the CASE study of **1a** was based on NMR isotropic constraints (Figure S24; isotropic data are presented in Tables S1, S3, and S6).

### 2.3 Attempt of Epimeric Discrimination of **1a/b** Using DP4 Methodologies

To discern between the two epimeric structures, we first applied a Bayesian Probability approach (DP4+ and i*J*‐DP4 methodologies) [7, 10] to NMR observables of **1a**/**b** run in DMSO‐*d_6_* and DCM‐*d_2_*. As described in the DP4+ methodology, a conformational search within a 5 kcal/mol window was performed, followed by a geometrical optimization at B3LYP/6‐31G** and computing the chemical shifts at mPW91PW91/6‐311+G**. This procedure generated an ensemble of conformers, considering the most Boltzmann‐populated geometries of **1b** and **1a**.

**FIGURE 3:**
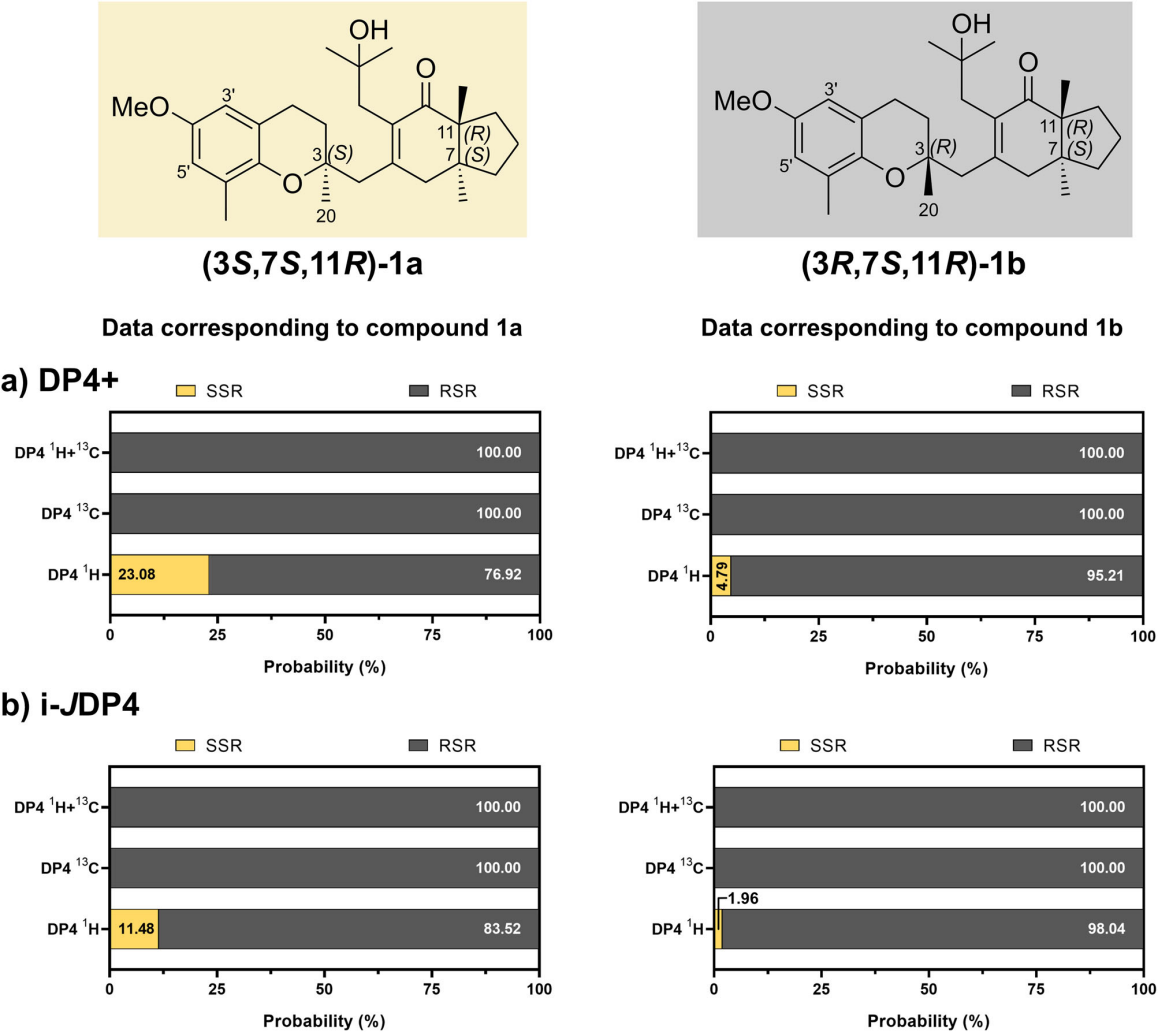
(a) DP4+ and (b) i*J‐*DP4 probability analysis of (3S*,7S*,11R*)‐**1a** (yellow) and (3R*,7S*,11R*)‐**1b** (gray) in DMSO‐*d_6_*. Calculations were performed at the B3LYP‐6/31 → PCM(IEF)‐MPW1PW1‐6/311+G** level of theory.

Comparison of the experimental ^13^C and ^1^H NMR data of **1a** and **1b** in DMSO‐*d_6_* with their calculated counterparts by applying the DP4+ methodologies gave the same relative configuration, (3R*,7S*,11R*), for both compounds, with 100% certainty (see Figure 3). The same result was obtained in DCM‐*d_2_* (Figure S25), confirming the inability of the DP4+ methodology to differentiate the two epimers at C3.

**FIGURE 4:**
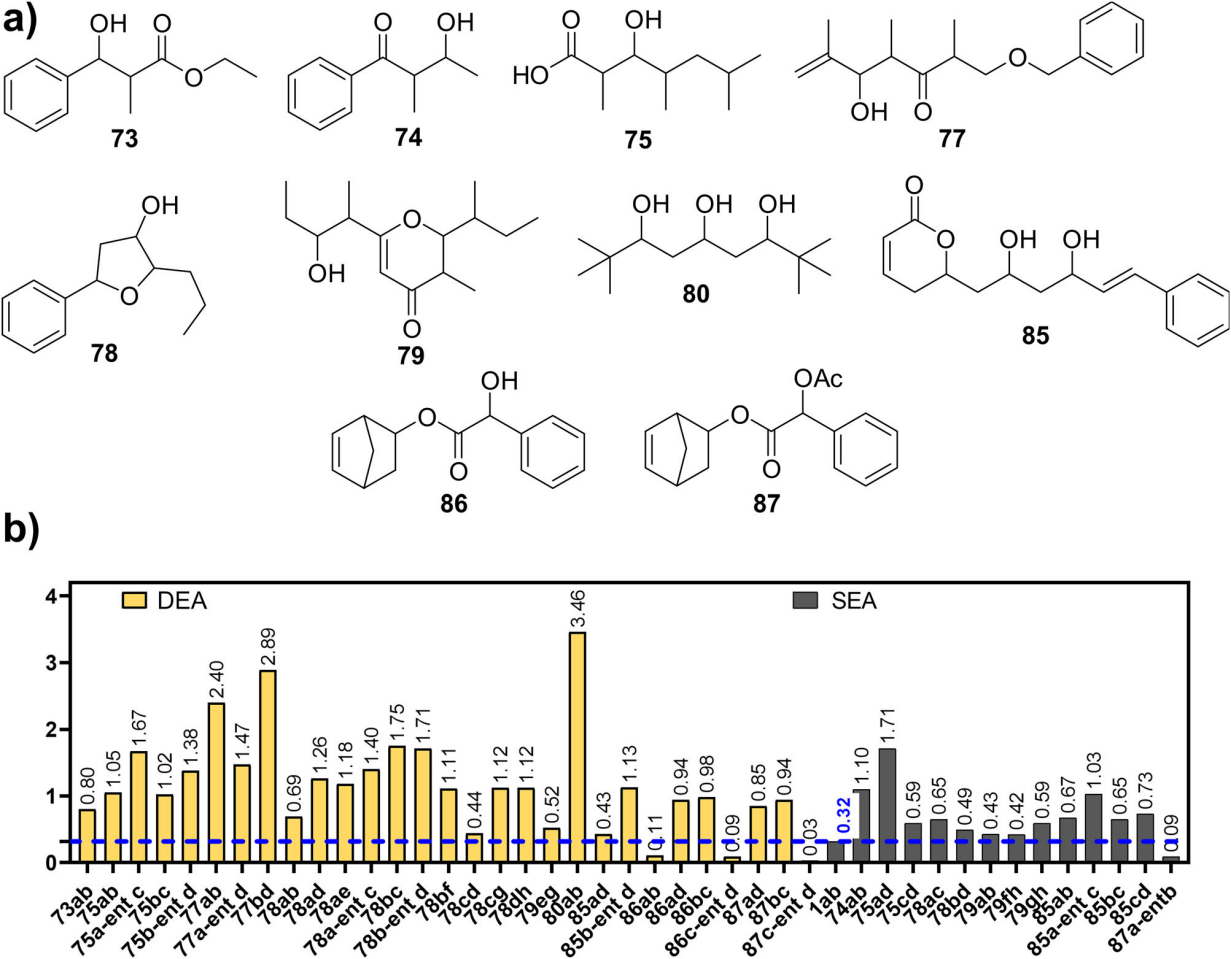
(a) Chemical structures of the selected epimer pairs. (b) Bar plot showing the $\bar{\Delta \delta_{C}}$ values of the selected pair of epimers reported by Sarotti et al. and the **1a/1b** pair. The two established case studies, DEA and SEA, are highlighted in yellow and dark gray, respectively. The $\bar{\Delta \delta_{C}}$ for **1a/1b** pair was 0.32 ppm (blue dotted line).

The limitation of the DP4 methodologies (DP4, DP4+, and i*J*‐DP4) in assigning the epimeric **1a**/**1b** prompted us to investigate their effectiveness in similar cases. For this reason, we conducted an EDA on several epimers reported by Sarotti et al. to assess the reliability of DP4+ methodologies in distinguishing them (recommended combination for DP4+ comparison: B3LYP/6‐31 → PCM(IEF)‐MPW1PW1/6‐31G**) [9]. A carefully selected set of epimer pairs was chosen from the diastereoisomers of the chemical structures **73**, **74**, **75**, **77**, **78**, **79**, **80**, **85**, **86**, and **87** and, with the introduction of the apparent epimers concept (see Figure S27), resulted in a total of 43 comparisons (we have kept the same numeration as in the original publication by Sarotti et al. [9], see Figure 4 and Figure S27).

All the epimeric pairs displayed minimal chemical shift differences [33], as evidenced by the correlation values for carbon (*R* ^2^ = 0.9999) and proton (*R* ^2^ = 0.9983) chemical shifts for **1a** and **1b**. The NMR data in DMSO‐*d_6_* are shown in Figure S26, along with the correlation values for the remaining 42 epimeric pairs (Figures S28–S66).

For each pair of epimers, the experimental ^13^C‐NMR chemical shift average difference ($\bar{\Delta \delta_{C}}$ in ppm) of carbons up to four bonds away from the epimeric center was calculated. The $\bar{\Delta \delta_{C}}$ obtained values were related to the reported DP4+ probability for each epimeric pair to evaluate its ability to discriminate them [34]. Two distinct cases were established from this analysis (see Figure 4):

- Dual Epimerical Assignment (DEA). The structure of both epimers was confidently assigned with a probability of 99.5% or higher.
- Single Epimer Assignment (SEA). Only the structure of one of the epimers was assigned with a probability of 99.5% or higher.

In the 43 comparisons performed with the model compounds together with our **1a**/**1b** pair, we observed that the correct structure could not be accurately assigned for any of the epimers.

Epimers categorized as DEA generally showed the higher $\bar{\Delta \delta_{C}}$ values, whereas they were smaller for the SEA epimers. The $\bar{\Delta \delta_{C}}$ value of 0.32 ppm for **1a**/**1b** pair, indicated by a dotted blue line in Figure 4, was lower than that of most epimer pairs selected in this analysis (Figure S27).

Then, we studied the influence of the number of rotatable bonds (RotB) on the efficiency of the DP4+ methodology. Here, we have used the default rotatable bond definition as implemented in the open‐source cheminformatics library RDKit [35]. The two case studies were further classified into three subcategories based on RotB as an indicator of the conformational flexibility that influences the NMR spectral data.

**FIGURE 5:**
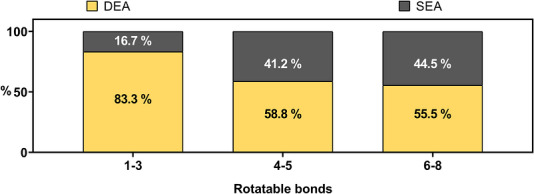
Stacked bar plot obtained from the EDA, considering the number of rotatable bonds (RotB) for the selected 43 pairs of epimers, using the DP4+ original data reported by Sarotti et al.

The higher the number of RotB in the considered molecular geometry (1–3, 4–5, or 6–8 rotatable bonds; see Figure 5), the greater its flexibility and, therefore, the greater the number of conformers involved [36]. The efficiency of the DP4+ methodology in correctly assigning both epimers with conformational flexibility decreases from 83.3% (1–3 RotB) to 58.8% (4–5 RotB), and 55.5% (6–8 RotB). Interestingly, the **1a**/**1b** pair falls into the second subcategory, where only 58.8% of the structures were accurately assigned.

### 2.4 Unsupervised Conformational Space Clustering Protocol to Analyze the Complex Conformational Flexibility of Epimers **1a** and **1b**

Unlike isotropic parameters, which are uniform regardless of molecular orientation, anisotropic parameters consider the angles related to the external magnetic field. This means that they provide valuable information about how different parts of the molecule are positioned relative to each other, even if those parts are far apart. Together with isotropic NMR parameters, anisotropic ones help to establish a more complete understanding of the molecule's configuration.

**FIGURE 6:**
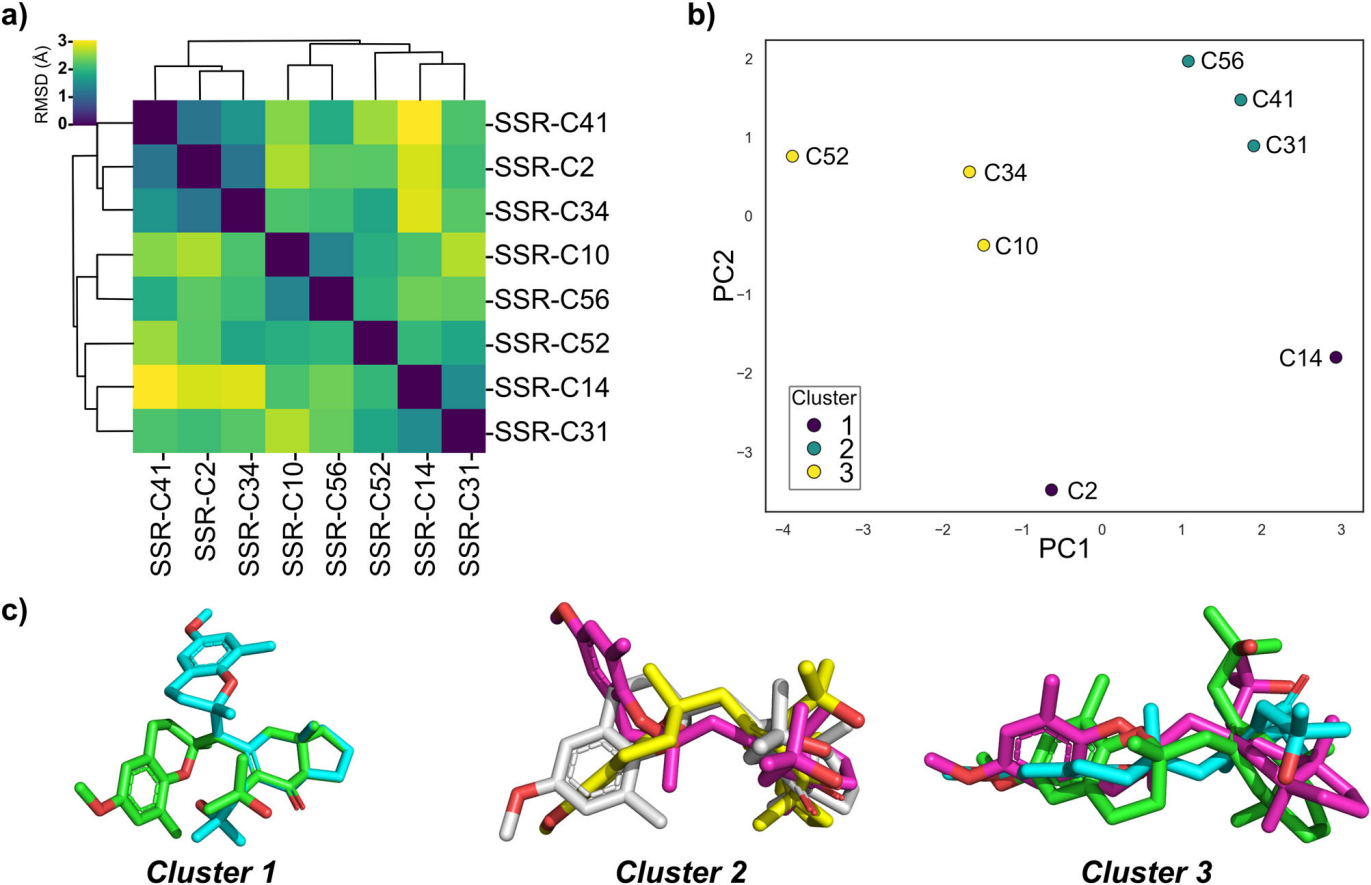
(a) Heat map hierarchically clustered showing the distribution of RMSD of conformers obtained for *SSR*‐**1a**. They are not redundant to each other to cover the full range across the conformational space within the energy threshold window (3.5 kcal/mol above the global lowest energy identified by DFT calculations). The intensity of the cell color is proportional to the RMSD of conformations. (b) d‐PCA + Rg of the conformational landscape of *SSR*‐**1a** based on significant dihedral and gyration radius. The optimal number of groups was determined using the silhouette method and structural relevance. (c) Molecular model of the conformers found for *SSR*‐**1a** Cluster 1: Conf‐2 (C2), Conf‐14 (C14); Cluster 2: Conf‐31 (C31), Conf‐41 (C41), Conf‐56 (C56), and Cluster 3: Conf‐10 (C10), Conf‐34 (C34), Conf‐52 (C52).

Epimeric pairs with molecules with conformational flexibility display very similar isotropic NMR properties, such as **1a/1b**; thus, an accurate description of the conformational space (CS) is crucial for elucidating the interconnections among isolated stereocenters and for correctly applying anisotropic NMR spectroscopy. For this reason, a new protocol, named unsupervised conformational space clustering, was needed to analyze the complex conformational flexibility of epimers **1a** and **1b**. This new protocol included:

- *Extensive conformational search* covering all relevant rotatable and torsional bonds.
- *Geometry refinement* using DFT M06‐2X [37]/6‐31+G(d,p)/IEFPCM = DMSO calculations and accurate single‐point energy computations employing an extensive basis set/solvent model (Supporting Information, page S58) at the mPW1PW91/6‐311+G(2d,p)/IEFPCM = DMSO level [38].
- *Removal of redundant conformers* showing only marginal differences in the RMSD of their atomic coordinates and energy values. Conformations within an RMSD threshold of 0.5 Å, computed over the heavy atoms, and an energy difference of 3.5 kcal/mol were selected. This selection process avoided redundancy caused by the rotation around the C14–C15 bond.
- *Visualization of the refined conformational pool through RMSD heatmaps* and hierarchical dendrogram groupings (Figure 6a and histograms in Supporting Information, Figures S73–S76).
- *Definition of the conformational space by descriptive statistics tools, including histograms and correlation matrices*. All experimentally identified rotatable bonds and ring torsions were considered, along with a molecular descriptor. The relevant torsions and rotations used to capture the structural variability of the conformational landscape of **1a** and **1b** were H4‐C4‐C3‐C20, H4‐C4‐C5‐C13, H14‐C14‐C13‐C5, H14‐C14‐C15‐C16, and H2‐C2‐C1‐C2’, along the gyration ratio (see Figures S77–S78).
- *Clustering of final conformers*. Each conformer was described by a feature vector composed of non‐redundant dihedral and torsional angles, along with the radius of gyration (Rg) (expressed as correlation matrices in the Supporting Information, Figures S79 and S82) [39]. Conformers within a 3.5 kcal/mol DFT‐SPE energy window were selected to represent the conformational landscape in a two‐dimensional manner using d‐PCA+Rg [40]. Clustering based on structural similarities (*vide supra*) was achieved through the silhouette method (Figure 6 and Figure S83). A heatmap illustrating the conformers before and after the removal of redundant ones is shown in the Supporting Information, Figures S73–S76. A closer examination of sets of conformers reveals that all significant rotatable bonds are fully represented, indicating that the proposed rotamers comprehensively represent the entire conformational space. Dihedral values found in the conformational space for H4‐C4‐C3‐C20 were consistent with the existence of both *P*‐ and *M*‐helicities in the chromane moiety. Eight conformers were found for **1a** and ten for **1b**.

It included a comprehensive conformational search, geometry refinement using DFT methods, and calculation of accurate single point energy employing an extensive basis set/solvent model (Supporting Information, page S58). The single point energy calculations at a deeper level of DFT involving continuum solvation models were essential to truthfully describe the relative weight of each conformer in the conformational space, as well as the inherent flexible molecule **1a** [38].

After DFT optimization, redundant conformers with marginal differences in the RMSD of the atomic coordinates of the heavy atoms and energy values were removed, resulting in several rotamers converging into similar geometries. For **1a** and **1b**, conformations within an RMSD threshold of 0.5 Å and an energy difference of 3.5 kcal/mol were selected. Figure 6a displays the heat map of selected conformers of *SSR*‐**1a** sorted by their RMSD value, thanks to the hierarchical agglomerative clustering with average linkage underlying patterns that correlate with structural similarities. A heatmap illustrating the conformers before and after the removal of redundant ones is shown in Figure S73–S76. A closer examination of sets of conformers reveals that all significant rotatable bonds are fully represented, indicating that the proposed rotamers comprehensively represent the entire conformational space. The implementation of histograms and Pearson correlation matrices for the exploration of the dihedral angles of rotatable bonds allowed us to detect the redundancy between H14‐C14‐C15‐C16 and H14‐C14‐C15‐C17; this selection process avoided redundancy caused by the rotation around the C14‐C15 bond (See histograms in Supporting Information, Figures S77 and S78).

The structures of **1a**/**b** feature four rotatable covalent bonds, as well as torsional flexibility around the chromane system: H4‐C4‐C5‐C12, H14‐C14‐C13‐C5, H14‐C14‐C15‐C17, and H2‐C2‐C1‐C1´.

Dihedral values found in the CS for H4‐C4‐C3‐C20 were consistent with the existence of both *P*‐ and *M*‐ helicities in the chromane moiety. Eight conformers were found for **1a** and ten for **1b**.

To explore the geometric diversity of both epimers, all conformers were represented as points in a multi‐dimensional space. The feature vectors were then compressed into a lower‐dimensional space using dihedral principal component analysis (d‐PCA) and segmented with the K‐means algorithm. d‐PCA accounts for the circular nature of dihedral angles by mapping each angle $\phi_{n}$ to the Euclidean pair $\left(x_{n},y_{n}\right)=\left(\cos\phi_{n},\sin\phi_{n}\right)$ before applying PCA [41]. This method was conceived to capture and visualize the extent and dispersion of the conformational space. Therefore, we recommend using torsional features (TF) for dimensionality reduction in molecular datasets. The analysis for the **1a** and conformational space resulted in the d‐PCA+Rg plot, which is shown in Figure 6, where geometries with similar features are grouped using the Silhouette Method (see **1b** in Supporting Information, Figure S83) [42]. Based on this approach, three main clusters were identified, and the corresponding refined conformers were used for the anisotropic NMR data analysis.

### 2.5 ^13^C‐RCSA and ^1^ *D*_CH_ Data Analysis of **1a**

To discriminate between epimers **1a** and **1b,** we first performed experiments using 900 µg of **1a** (2.04 × 10^−6^ mol), aligned in poly‐HEMA with DMSO‐*d*_6_ as the solvent [43], as this is a versatile and well‐established alignment medium within the community [44, 45, 46, 47, 48], using a standard 5 mm compression device. The experiment was run on a 600 MHz spectrometer equipped with a 5 mm cryogenic probe optimized for ^1^H detection (inverse). The RCSAs experimental error (σRCSA) estimation was obtained by averaging the ratio of peak width to signal‐to‐noise ratio for the relevant resonances. The result was expressed in ppb. The subsequent ^13^C‐NMR spectrum showed an unfavorable experimental error σRCSA = ±1.3_7_ ppb, and several resonances were hidden under polymer signals. This data analysis resulted in an ambiguous epimer assignment, as the error Monte Carlo analysis showed that epimer discrimination was not conclusive (Figure S84).

Following this, a different experiment was performed with 800 µg (1.8 × 10^−6^ mol) of both epimers aligned in poly‐HEMA/DMSO‐*d*_6_ using a 3 mm semi‐micro compression device (SMCD) in a highly sensitive 1.2 GHz Bruker spectrometer equipped with a 3 mm inverse cryogenic probe. RCSAs in Hz scale linearly with the size of the magnetic field, reducing the relative experimental error at the same signal‐to‐noise ratio. The SMCD represents an improvement of the previous version developed by us [19], in collaboration with Hilgenberg GmbH. The use of the highly sensitive 3 mm cryogenic probes in conjunction with an ultra‐high field 1.2 GHz spectrometer reduces the number of scans (NS) necessary to achieve the *σ*_RCSA_ required for epimer discrimination. The ratio of **1a** to gel peaks in the 3 mm tube was improved by a factor of 3.0 compared to the 5 mm tube. The decrease of the polymer signal reduces the experimental error, guiding an improvement in the relative configuration assignment and leading to a better distinction between epimers. The poly‐HEMA mass stick was only 41.5 mg for the SMCD versus 104.5 mg for the conventional 5 mm compression device [49].

The modified 3 mm NMR tube for the SMCD is now available from Hilgenberg GmbH (product number 2008232). Technical drawings, details, and materials are shown in Figures S85–S86.

^13^C‐RCSAs were recorded independently for **1a** and **1b**, along with RDCs, both NMR anisotropic observables were fitted together in a single analysis. The ^13^C‐RCSAs of 21 of the 28 possible were measured using a ^13^C‐NMR experiment because they were either hidden under the polymer signals or indistinguishable due to overlapping (Table 1).

**TABLE 1:** Experimental values of ^13^C‐RCSA_uncorr_ and ^1^ *D*_CH_ of **1a** and **1b**.

Atom	^13^C‐RCSA (ppb) of **1a**	^1^ *D*_CH_ (Hz) of **1a**	^13^C‐RCSA (ppb) of **1b**	^1^ *D*_CH_ (Hz) of **1b**
C1	22.118	0.68	−11.033	−0.89
C2	23.628	−6.19	^b^	−2.77
C3	9.920	—	−41.033	—
C4	^a^	1.91	−9.439	−6.76
C5	−3.595	—	2.710	—
C6	15.431	^a^	−9.055	2.91
C7	23.559	—	−20.054	—
C8	^a^	^a^	−23.106	0.72
C9	25.070	−4.26	−37.256	−3.79
C10	^a^	3.30	−8.826	3.15
C11	16.250	—	−6.871	—
C12	−7.185	—	−16.466	—
C13	^a^	—	10.317	—
C14	^a^	4.59	−2.372	3.09
C15	18.950	—	−23.623	—
C16	^a^	−0.36	−33.128	1.33
C17	^a^	−0.36	19.428	1.33
C18	21.228	1.62	−22.963	0.17
C19	17.800	1.71	−14.512	0.57
C20	20.856	−0.90	−41.033	−0.69
C1'	6.784	—	−63.698	—
C2'	27.358	—	26.959	—
C3'	25.536	−9.26	29.384	−13.01
C4'	10.634	—	−49.243	—
C5'	39.087	−12.20	40.626	−11.82
C6'	33.408	—	51.953	—
Me‐C6'	18.604	0.90	−34.874	3.57
MeO–	^b^	5.27	^a^	^a^

*Note*: NMR experiments were recorded in DMSO‐*d6* using a poly‐HEMA matrix on a 1.2 GHz Bruker spectrometer.

^a^ Not measurable due to polymer background.

^b^ Carbon used for referencing in the RCSA extraction.

In Table 1, ^13^C‐RCSA_uncorr_ represents the ^13^C‐RCSA values without isotropic correction, where the isotropic correction factor (c) was determined to be 0.135 for **1a** and 0.125 for **1b**. The ^13^C‐RCSA values ranged from −7.18 to 39.09 ppb. A significant decrease in the experimental error *σ*_RCSA_ was observed, from 1.37 ppb (600 MHz, 5 mm compression device) to 0.58 ppb (1.2 GHz, 3 mm SMCD). RDCs at 1.2 GHz were extracted from a *J*‐Res‐F1‐HSQC spectrum using non‐uniform sampling (NUS). A total of 15 ^1^ *D*_CH_ values, ranging from −12.20 to 5.27 Hz, were obtained (Table 1). RDCs for methylene groups were reported as half of the sum of the couplings of both protons (Ha and Hb), while for methyl groups, RDCs were reported as a third of the sum of the couplings of the three protons involved. The small value of the MAE of 0.09 Hz reflects the high precision of the measurements.

^13^C‐RCSA/RDC data were fitted to a set of non‐redundant molecular geometries within an energy window of 3.5 kcal/mol, as they rely on the same alignment tensor [50]. The isotropic correction factor (c) was optimized along with the alignment tensor [51], using the least‐squares Levenberg–Marquardt (LM) procedure. All conformers were superimposed on their heavy atoms, and anisotropic parameters were fitted to a single tensor [52] by singular value decomposition (SVD) [53] as implemented in MSpin software [54]. The quality of the fitting of anisotropic parameters was assessed by conformer population refinement due to Cornilescu Q‐factor minimization (“Fit population” option checked), while the experimental errors of both ^13^C‐RCSA/^1^ *D*_CH_ (σ_RCSA_/σ_RDC_) were incorporated in the alignment tensor determination by a Monte Carlo error analysis.

**FIGURE 7:**
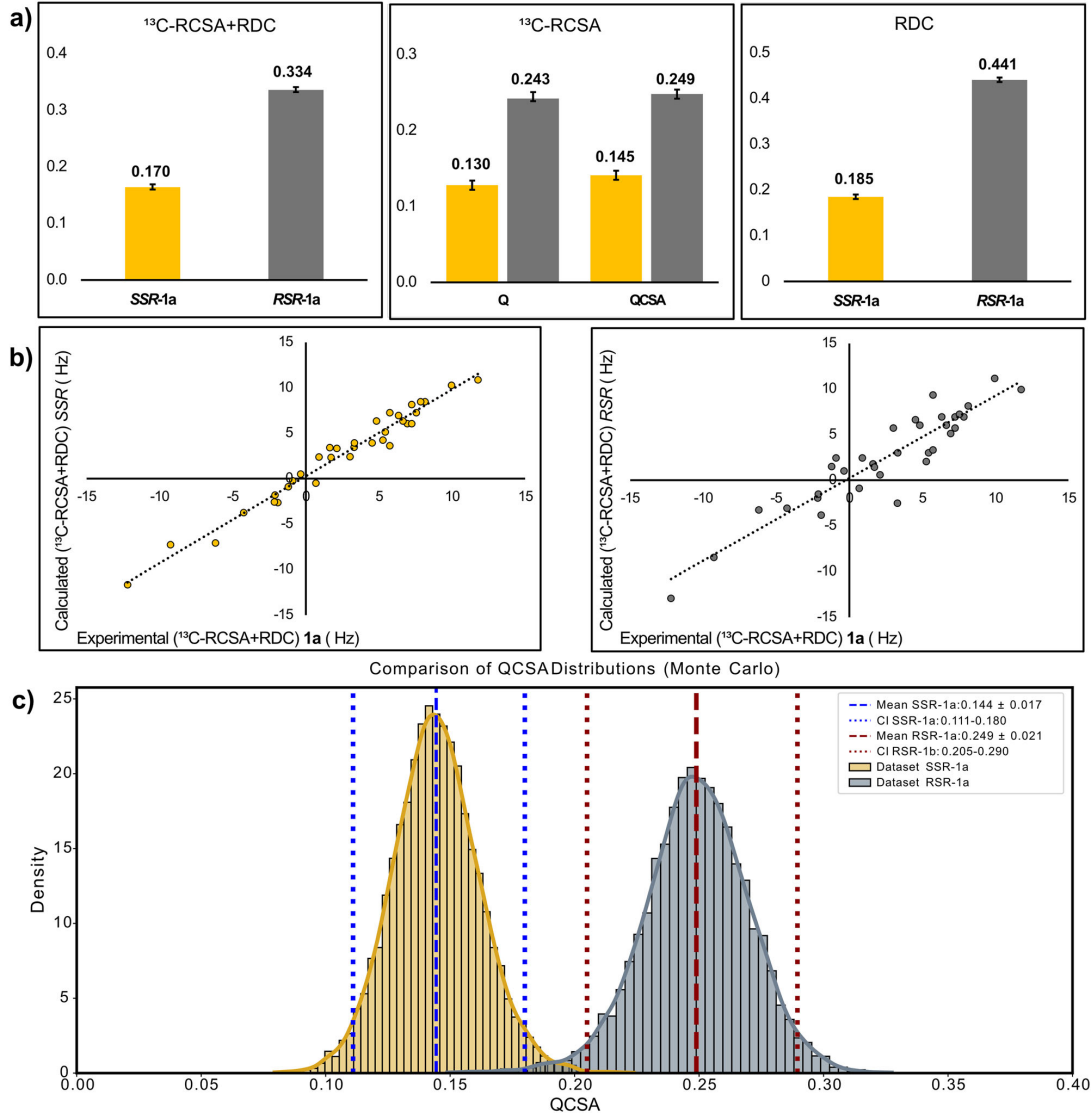
(a) Cornilescu's quality factor (Q) analyses of the ^13^C‐RCSA+RDC combined data, ^13^C‐RCSA, and RDC for **1a**. (b) The fitting curve of the experimental combined data (^13^C‐RCSA+RDC) of **1a** versus back‐calculated data (^13^C‐RCSA+RDC) for 3*S*7*S*11*R* (yellow) and 3*R*7*S*11*R* (gray) configurations, respectively. (c) Monte Carlo error propagation analysis in the ^13^C‐RCSA‐based discrimination of **1a**‐*SSR*/*RSR* epimers, assuming Gaussian error distributions. The resulting plot displays the mean QCSA value, its standard deviation (QCSA±σ), and the corresponding 95% confidence interval (CI). The random sample number of points was 500 000 generated within a ±3σ range to cover 99.7% of the expected variation.

Ultimately, the combined RCSA/RDC data were fitted to the molecular models, and the results of the ^13^C‐RCSA/^1^ *D*_CH_ study between S*S*R*‐**1a** (yellow) and R*S*R*‐**1b** (gray) are shown in Figure 7.

The same behavior was observed when ^13^C‐RCSAs and RDCs were independently fitted. The Q values of 0.130(0.145) ± 0.006(0.017) for *SSR*‐**1a** versus 0.243(0.249) ± 0.006(0.021) for *RSR*‐**1b** allowed us to discriminate them, where discrimination due to the ^13^C‐RCSA scaling at 1.2 GHz produces a smaller error‐*σ*_RCSA_ [23]. The RDC data, fitted to the same set of conformers used in the RCSA analysis, yielded a *Q*_SSR_ of 0.185 ± 0.005 for **1a** and a *Q*_RSR_ of 0.441 ± 0.005 for **1b**, showing a clear discrimination between these epimers.

These results were supported by the Monte Carlo error analysis, which showed a small associated error in the Q‐factors. As expected, for alignment tensors obtained from different sets of anisotropic parameters under the same alignment condition, the intertensor angle (ITA) between the ^13^C‐RCSA+RDC versus RCSA derived tensors was only 5.1°, while the ITA for ^13^C‐RCSA+RDC versus RDC tensors was 5.2° (Table S8) [50]. The use of anisotropic NMR spectroscopy not only confirmed the S*S*R* relative configuration assigned to **1a** but also demonstrated the robustness of epimer discrimination (Figure 7) [23].

### 2.6 ^13^C‐RCSA and ^1^ *D*_CH_ Data Analysis of **1b**

Once the relative configuration of **1a** was confirmed by anisotropic NMR parameters, the same methodology was applied to its epimer **1b** under identical conditions (poly‐HEMA in DMSO‐*d_6_* and using a SMCD).

The anisotropic data of **1b** were fitted together with a set of molecular models chosen *vide supra*, following similar criteria as for **1a**. A total of 27 ^13^C‐RCSAs were extracted from its ^13^C NMR experiment and were spread in the range between ‐63.7 ppb and 51.9 ppb. Similarly, 15^1^ *D*_CHs_ were effectively extracted from a F1‐*J*‐resolved HSQC experiment, covering the whole **1b** scaffold, and they were extended from −13.01 to 3.57  Hz. RCSAs and RDC average experimental errors were estimated to be 0.66 ppb and 0.03 Hz, respectively, and they were incorporated in the data fitting.

**FIGURE 8:**
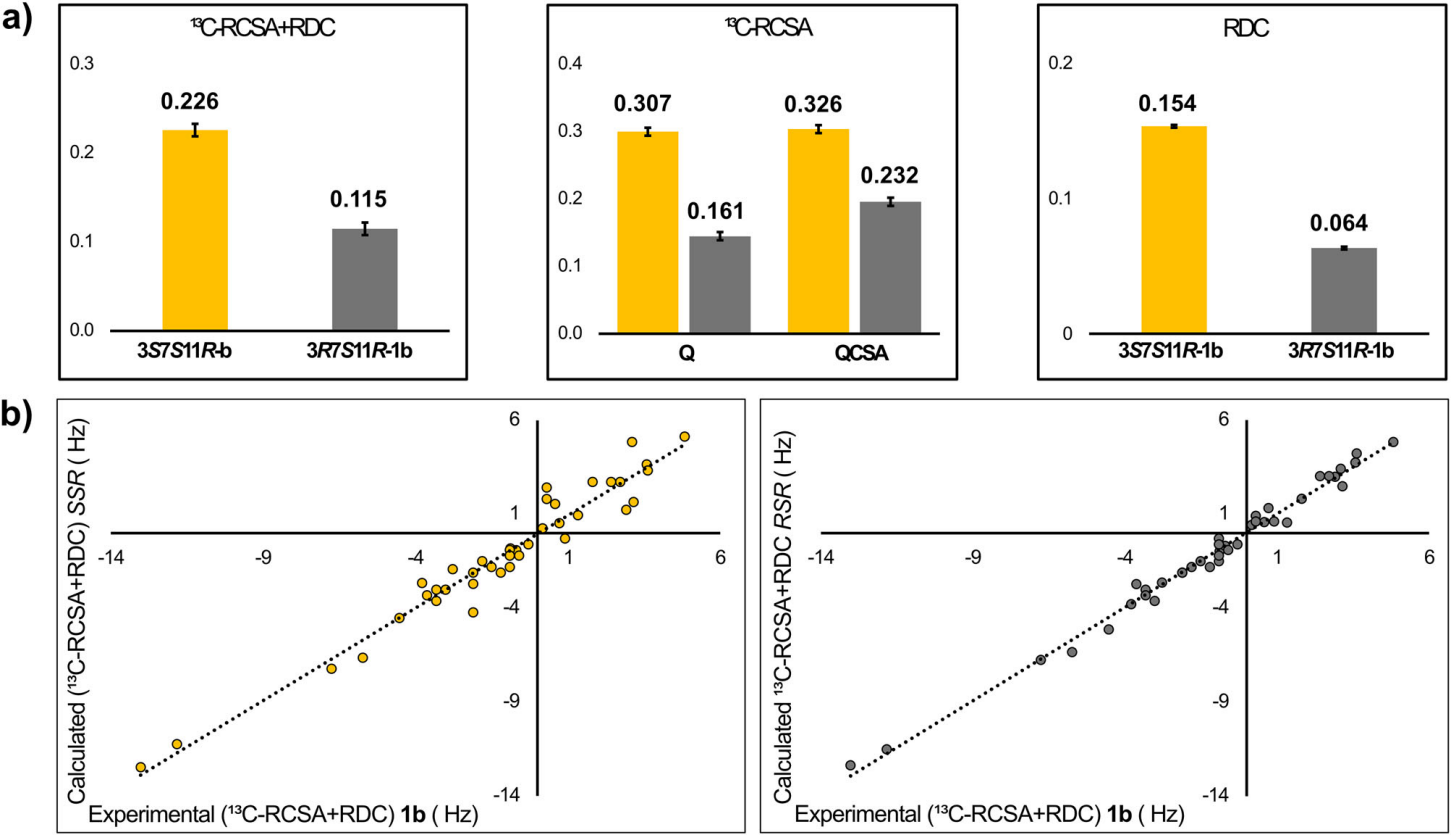
(a) Cornilescu's quality factor (Q) analyzes the ^13^C‐RCSA+RDC combined data, ^13^C‐RCSA, and RDC for **1b**. (b) Fitting curves of the experimental combined data (^13^C‐RCSA+RDC) of **1b** versus back‐calculated data (^13^C‐RCSA+RDC) for 3*S*7*S*11*R* and 3*R*7*S*11*R* configurations, respectively.

^13^C‐RCSAs and RDC data were individually fitted and combined to the minimum Q value to show discrimination in each case. Cornilescu Q‐factor minimization based on the combination of ^13^C‐RCSA and RDC parameters gave a value of 0.115 ± 0.007 and 0.226 ± 0.007 for the *RSR* and *SSR* configurations, respectively (Figure 8a). The quality of the fitting was supported by the high linearity observed between experimental and back‐computed ^13^C‐RCSAs/RDCs data (Figure 8b).

Likewise, when the set of conformers of **1a** and **1b** was fitted to RCSAs and RDCs independently, the epimeric discrimination was still observed. ^13^C‐RCSA analysis gave a Q(QCSA) values of 0.161(0.232) ± 0.015(0.035) for *RSR*‐1b and 0.307(0.326) ± 0.017(0.037) for *SSR*‐**1a**, while the RDC analysis yielded *Q* values of 0.064 ± 0.001 for *RSR*‐**1b** and 0.154 ± 0.001 for its epimeric counterpart **1a**. These results showed that the use of anisotropic parameters can unambiguously discriminate the epimers bearing stereoclusters separated by several covalent rotatable bonds.

The small angle between the ^13^C‐RCSA+RDC alignment tensor and the resulting alignment tensor from the individual RCSA/RDC fitting of 1.5°/1.8° indicated the accuracy of the recorded data. The improvement of epimeric discrimination was evidenced by the increased magnitude and number of ^13^C‐RCSA values extracted from a 1.2 GHz NMR spectrometer. These findings highlighted the effectiveness of the combination of anisotropic NMR parameters in epimeric differentiation when DP4+ analysis fails.

Unfortunately, accurate measurements of long‐range RDC values proved to be challenging due to their small magnitudes and susceptibility to error. In the future, new opportunities for utilizing ^n^ *D*_CH_ [55, 56] in the study of conformational flexibility and molecules will arise through the advances of enhancing sensitivity, increasing the magnetic field of the spectrometer, removing the polymer signal via alignment media deuteration, and using a micro‐NMR anisotropy apparatus [19, 57, 58].

### 2.7 Epimeric Differentiation of **1a** and **1b** by Comparison of the Alignment Tensor

**FIGURE 9:**
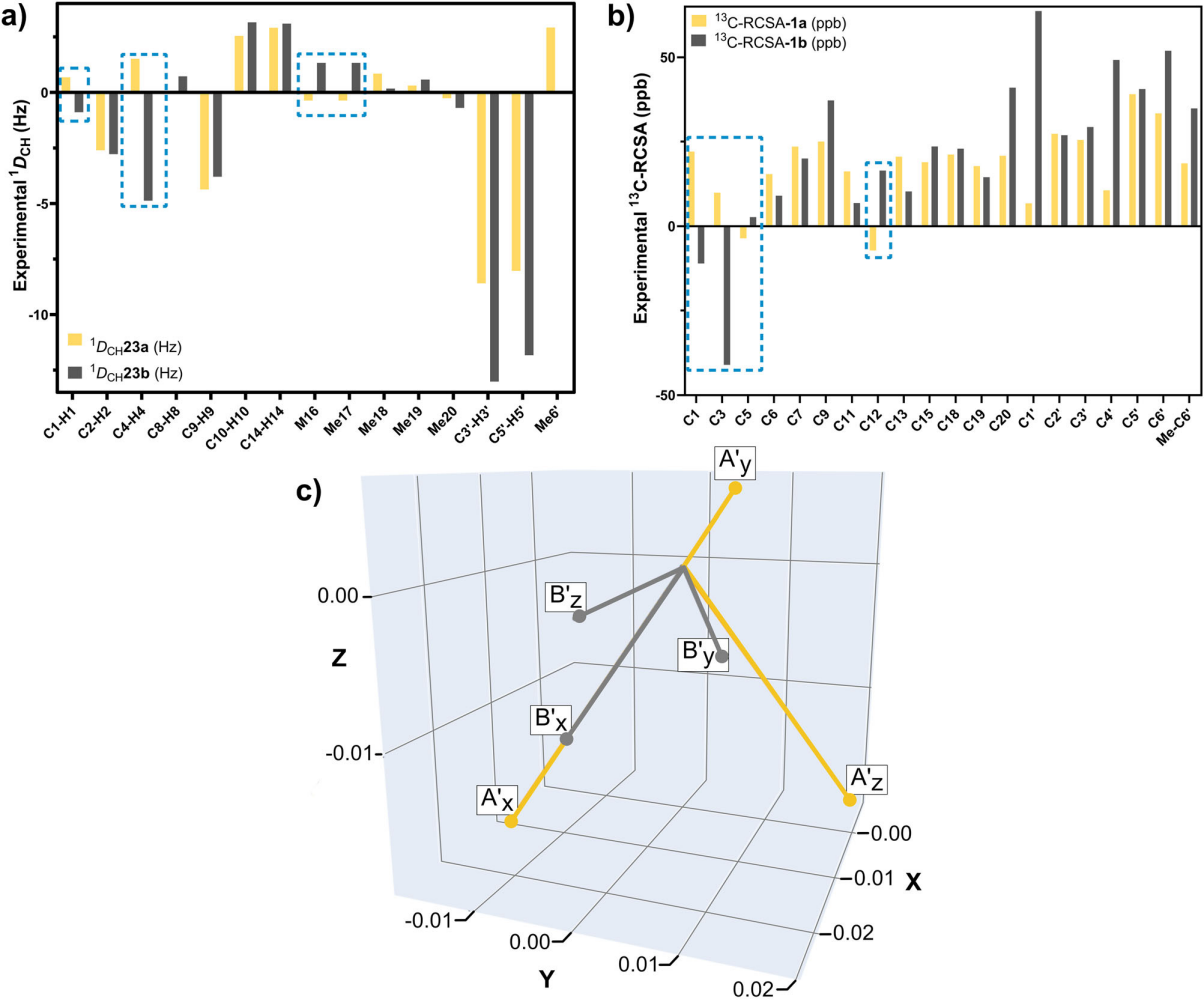
Comparison of scaled RDCs in Hz (a) and ^13^C‐RCSAs in ppb (b) between **1a** and **1b** data oriented in poly‐HEMA (DMSO). Those anisotropic parameters that display a sign inversion between **1a** and **1b** are indicated by a dashed blue line. Comparison of the alignment tensors (c) derived from ^13^C‐RCSA+RDC data for compounds **1a** (alignment tensor A’; dark yellow) and **1b** (alignment tensor B’; gray) overlapped on the principal axes (A'x and B'x), preserving eigenvalue magnitudes. Parameters were recorded in an ultra‐high field 1.2 GHz spectrometer.

The different interactions of each epimer, **1a** and **1b**, with the polymer matrix result in differences not only in the magnitude but also in the sign of anisotropic parameters, such as RDC and RCSA values (Figure 8a,b). The different spatial dispositions of the atoms in each epimer lead to variations in the interaction vectors and chemical shift anisotropy tensors. To investigate the differences between the RDC‐ and RCSA‐derived tensors of both epimers **1a** and **1b**, the Intertensor Angle (ITA) was calculated using ^13^C‐RCSA + ^1^ *D*_CH_ analysis. An ITA value of 66° was obtained, indicating that the interactions of each epimer with the polymer matrix are different, as expected from diastereomers (Figure 9c). This behavior was previously observed in the study of two epimeric tetranortriterpenoids isolated from *Xylocarpus rumphii* by RDCs [59]. While it is nice to observe a large ITA for epimers, we should stress that epimer differentiation could also be achieved with an ITA of 0°.

The principal axes of the alignment tensors derived from RCSA+RDC data for compounds **1a** (gray) and **1b** (yellow ocher, as shown in Figure 9) are represented as solid lines in panel (c). To simplify comparisons, the A'/B'x axes are oriented in the same direction. The 3D projection enhances the differentiation between the intermediate ordered axis (A'/B'y) and the most ordered axis (A'/B'z) for each epimer. Visual inspection indicates that the alignment tensors for the **1a** and **1b** meroditerpenes exhibit differing orientations relative to each other, with the ITA confirming this distinction.

As it was stated before, **1a** and **1b** exhibit significant conformational flexibility. This is evidenced by the inability of DP4‐based approaches to reliably correlate the two stereoclusters, which are separated by four covalent bonds, two of which are freely rotatable. Systems with this degree of flexibility often raise concerns regarding the applicability of the Single Tensor Multi‐Conformer approach, as the absence of a consistent superimposition frame, readily available in more rigid structures, complicates its use [60]. To address this well‐founded concern regarding the protocol outlined above, we have applied a workflow, multi tensor multi conformer (MTMC) RDC‐based, to the study of both **1a** and **1b** (detailed described in SI, Page S110). Our results confirm the assignment of the relative configuration of C3 in both **1a** and **1b**, with a lower Q‐factor, and the linearity of the fitting improved. Therefore, we have observed that both the STMC and MTMC analyses are valid and could be explained in the same terms as fibrosterol sulfate [61].

### 2.8 Absolute Configuration of **1a**

To determine the absolute configuration of **1a**, two chiroptical modalities, electronic circular dichroism (ECD) and specific rotation ([α]^D^_25_), were employed by comparing the experimental with the calculated data. However, the calculation of the chiroptical properties of **1a** became very challenging because of its high conformational flexibility, mainly due to the presence of two possible helicities in the chromane fragment and the conformations resulting from the rotation of the hydrindane and the chromane moieties around the C3–C4–C5 bonds.

For example, the Cotton effect (^1^ *L*_b_) originating from the chromane moiety in **1a** is highly sensitive to the existence of several conformers with different helicities [62, 63]. For this reason, the accurate simulation of the ECD spectrum of **1a** was achieved by considering the conformers derived from the best anisotropic fit to reproduce the experimental ECD curve correctly. These conformers are in perfect agreement with the isotropic NMR data and the observed equilibrium of helicities (*P*‐46.5% and *M*‐53.5%). Consequently, the absolute configuration of **1a** was determined using the TD‐DFT method with double hybrid functionals, where the DFT‐optimized conformers were anisotropically/*J*‐weighted.

For this calculation, we have used double hybrid functionals, which showed remarkable performance in estimating ECD spectra. For each conformer, the first 90 excited states were computed using the combination PW6B95D3/6‐311+G(3d,2p) to give spectra with a 0.22 eV linewidth at half height.

**FIGURE 10:**
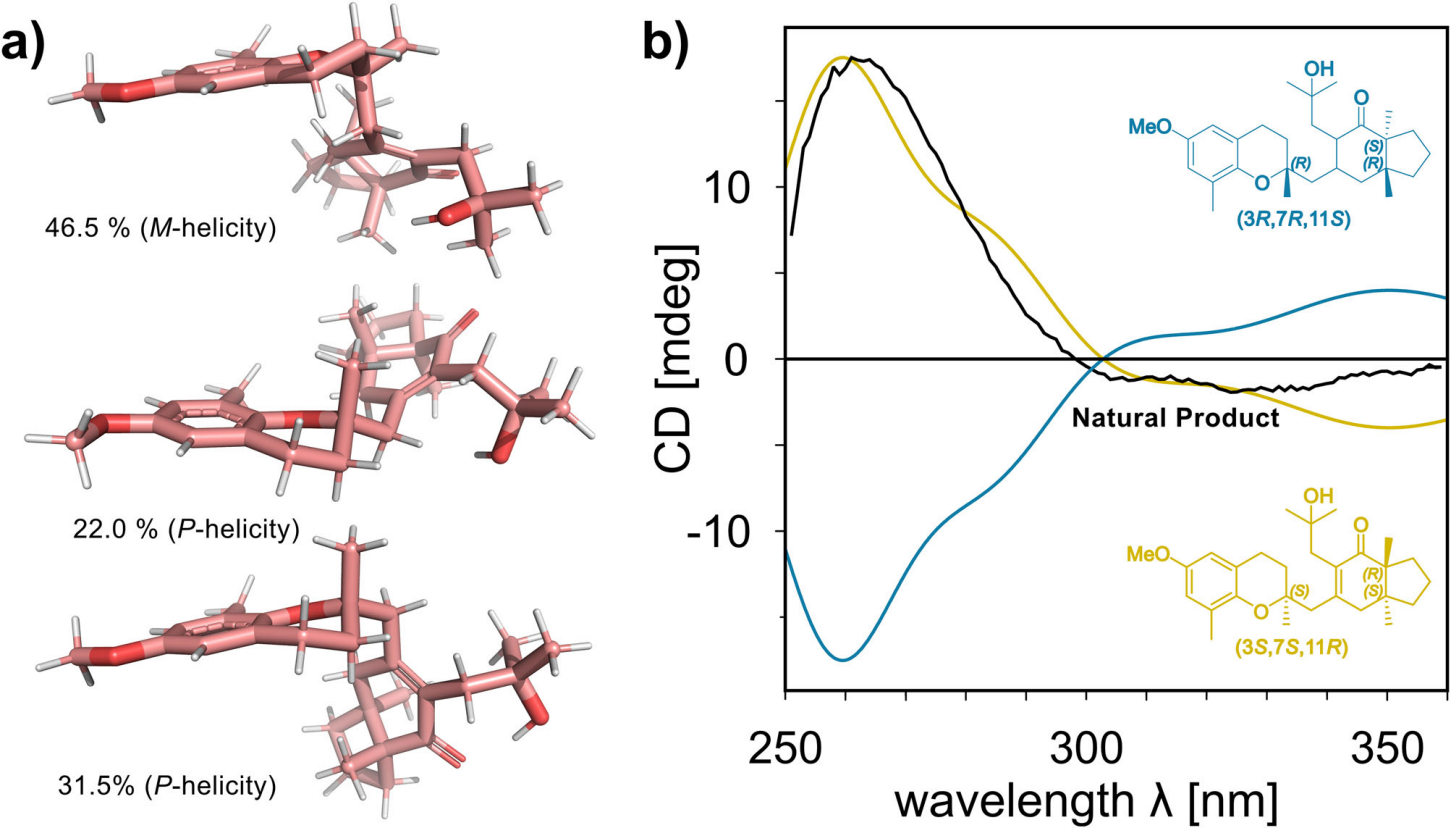
Absolute configuration assignment of **1a** based on chiroptical ECD data: (a) derived conformers from anisotropic data used to ECD calculations. (b) Comparison of the experimental (black) ECD spectrum of **1a** and those calculated for *SSR* and *RRS* configurations (yellow and blue).

As shown in Figure 10, the calculated ECD spectrum for the 3*S*,7*S*,11*R* configuration matched accurately with the experimental spectrum of **1a** across the entire wavelength range when a 6 nm wavelength shift was applied. In this way, the absolute configuration of **1a** could be established.

## 3 Conclusions

Attempts to differentiate the epimers of (3*S*7*S*11*R*)‐**1a** and (3*R*7*S*11*R*)‐**1b** using DP4‐based methodologies, including DP4, DP4+, and *J*‐DP4, proved unsuccessful once the average carbon chemical shift difference ($\bar{\Delta \delta_{C}}$) approached 0.32 ppm. At this level, the presence of the high conformational flexibility of the molecules resulted in proton and carbon chemical shifts that were too similar to allow reliable discrimination between the epimeric pairs.

The effectiveness of the DP4 methodology in the discrimination of epimers **1a** and **1b** was evaluated from their isotropic NMR data by analyzing a set of 42 reported pairs of epimers. As a result, a relationship was found between the presence of rotatable bonds and the effectiveness of the DP4+ methodology. When the number of rotatable bonds exceeds three, the likelihood of accurate discrimination between epimers drops to less than 50%.

The use of anisotropic NMR allowed the epimer discrimination of the **1a/1b** pair. First, their geometric diversity was clustered by dihedral principal component analysis (d‐PCA) + Rg to select the optimal set of conformers for each epimer. Molecular geometries were treated as points in a high‐dimensional space, which was then reduced using d‐PCA and segmented with the K‐means algorithm. The dimensions of this hyperdimensional space included dihedral angles related to each rotatable bond, torsional angles in the chromane moiety, one independent molecular descriptor, radius of gyration, and several rotatable bonds. This method aimed to capture and visualize the range and dispersion of the conformational space, removing redundant conformers.

Then, the relative configurations of **1a** and **1b** were successfully distinguished through the independent and combined utilization of ^1^ *D*_CH_ and ^13^C‐RCSA data that were obtained by using a compressed poly‐HEMA matrix. These findings underscore the critical role of experimentally constrained DFT models in achieving precise determinations of relative configurations.

RDCs exhibited a slightly better ability to differentiate the epimers compared to RCSAs when analyzing **1a**, almost the same discrimination power is found for compound **1b** (Table S12). RDCs (^1^ *D*_CH_) depend directly on the relative orientation of the involved chemical bonds with respect to the alignment tensor ($\hat{A}$), which describes how the analyte aligns within the polymeric matrix. In contrast, because ^1^ ^3^C‐RCSAs are determined by a combination of ($\hat{A}$) and the chemical shielding tensor (CST), which is less sensitive to subtle changes in molecular conformation, they exhibit a more modest discriminatory ability in some cases.

ROESY/NOESY and DFT conformational analysis of **1a** and **1b** displayed different conformational spaces. These differences affected not only bond orientations but also the internuclear vector alignment in space, leading to distinct RDC values.

Meanwhile, ^1^ ^3^C‐RCSAs were primarily influenced by the local electronic environment around the epimeric stereocenter C3, as well as the surrounding carbons, and their average orientation relative to $\hat{A}$. However, the inversion of the C3 configuration is expected to produce a more localized impact on the RCSA values, leading to a subtle change in the Q factor. With these findings, both techniques can be strategically employed to improve the accuracy of relative configuration determination and epimer discrimination of molecules with high conformational flexibility. Finally, the absolute configuration of **1a** as 3*S*7*S*11*R* was determined using chiroptical‐DFT methods.

This study improves the stereochemical analysis of complex organic molecules by highlighting the challenges and potential solutions in distinguishing epimers with high conformational flexibility.

In summary, we demonstrated in this work that the use of anisotropic NMR parameters represents a good approach to overcoming the limitation of DP4 methodologies in the assignment of the relative configuration of epimeric compounds that exhibit very similar isotropic NMR parameters.

## Author Contributions

**Juan Carlos C. Fuentes‐Monteverde**: writing – original draft, software, data curation, visualization, investigation, methodology, validation, writing – review and editing, formal analysis. **Abel M. Forero**: writing – original draft, data curation, investigation, methodology, writing – review and editing, formal analysis. **Nilamoni Nath**: writing – review and editing, investigation, writing – original draft, methodology, validation, visualization, software, formal analysis, data curation. **Antonio Hernández Daranas**: writing – review and editing, visualization. **Carlos Jiménez**: writing – review and editing, visualization. **Jaime Rodríguez**: writing – review and editing, project administration, formal analysis, conceptualization, visualization, resources, supervision. **Christian Griesinger**: funding acquisition, writing – review and editing, project administration, supervision, formal analysis, conceptualization, visualization, resources.

## Conflicts of Interest

The authors declare no conflicts of interest.

## Supporting information



The authors have cited additional references within the Supporting Information.
**Supporting File**: anie72843‐sup‐0001‐SuppMat.docx.

## Data Availability

The data that support the findings of this study are available from the corresponding author upon reasonable request.

## References

[1] N.‐T.‐H. Le , T. Vermeyen , R. Aerts , W. A. Herrebout , L. Pieters , and E. Tuenter , “Epimeric Mixture Analysis and Absolute Configuration Determination Using an Integrated Spectroscopic and Computational Approach—A Case Study of Two Epimers of 6‐Hydroxyhippeastidine,” Molecules 28 (2022): 214, 10.3390/molecules28010214. 36615407 PMC9822407

[2] H. R. H. Ali , H. G. M. Edwards , J. Kendrick , T. Munshi , and I. J. Scowen , “An Experimental and Computational Study on the Epimeric Contribution to the Infrared Spectrum of Budesonide,” Drug Testing and Analysis 2 (2010): 447–451, 10.1002/dta.152. 20812265

[3] C. Demetzos , A. Kolocouris , and T. Anastasaki , “A Simple and Rapid Method for the Differentiation of C‐13 Manoyl Oxide Epimers in Biologically Important Samples Using GC–MS Analysis Supported With NMR Spectroscopy and Computational Chemistry Results,” ACS Medicinal Chemistry Letters 12 (2002): 3605–3609, 10.1016/S0960-894X(02)00792-8. 12443786

[4] S. T. Mutter , F. Zielinski , C. Johannessen , P. L. A. Popelier , and E. W. Blanch , “Distinguishing Epimers Through Raman Optical Activity,” Journal of Physical Chemistry A 120 (2016): 1908–1916, 10.1021/acs.jpca.6b00358. 26928129

[5] R. W. Adams , J. A. Aguilar , J. Cassani , G. A. Morris , and M. Nilsson , “Resolving Natural Product Epimer Spectra by Matrix‐Assisted DOSY,” Organic & Biomolecular Chemistry 9 (2011): 7062, 10.1039/c1ob06097j. 21874201

[6] H. Kato , T. Nehira , K. Matsuo , et al., “Niphateolide A: Isolation from the Marine Sponge Niphates Olemda and Determination of Its Absolute Configuration by an ECD Analysis,” Tetrahedron 71 (2015): 6956–6960, 10.1016/j.tet.2015.07.009.

[7] S. G. Smith and J. M. Goodman , “Assigning Stereochemistry to Single Diastereoisomers by GIAO NMR Calculation: The DP4 Probability,” Journal of the American Chemical Society 132 (2010): 12946–12959, 10.1021/ja105035r. 20795713

[8] S. G. Smith and J. M. Goodman , “Assigning the Stereochemistry of Pairs of Diastereoisomers Using GIAO NMR Shift Calculation,” Journal of Organic Chemistry 74 (2009): 4597–4607, 10.1021/jo900408d. 19459674

[9] N. Grimblat , M. M. Zanardi , and A. M. Sarotti , “Beyond DP4: An Improved Probability for the Stereochemical Assignment of Isomeric Compounds Using Quantum Chemical Calculations of NMR Shifts,” Journal of Organic Chemistry 80 (2015): 12526–12534, 10.1021/acs.joc.5b02396. 26580165

[10] N. Grimblat , J. A. Gavín , A. Hernández Daranas , and A. M. Sarotti , “Combining the Power of *J* Coupling and DP4 Analysis on Stereochemical Assignments: The *J* ‐DP4 Methods” Organic Letters 21 (2019): 4003–4007, 10.1021/acs.orglett.9b0119. 31124687

[11] C. Cuadrado , A. H. Daranas , and A. M. Sarotti , “May the Force (Field) Be With You: On the Importance of Conformational Searches in the Prediction of NMR Chemical Shifts,” Marine Drugs 20 (2022): 699, 10.3390/md20110699. 36355022 PMC9694776

[12] Y.‐H. Tsai , M. Amichetti , M. M. Zanardi , R. Grimson , A. H. Daranas , and A. M. Sarotti , “ML‐ J ‐DP4: An Integrated Quantum Mechanics‐Machine Learning Approach for Ultrafast NMR Structural Elucidation,” Organic Letters 24 (2022): 7487–7491, 10.1021/acs.orglett.2c01251. 35508069

[13] B. A. Franco , E. R. Luciano , A. M. Sarotti , and M. M. Zanardi , “DP4+App: Finding the Best Balance Between Computational Cost and Predictive Capacity in the Structure Elucidation Process by DP4+. Factors Analysis and Automation,” Journal of Natural Products 86 (2023): 2360–2367, 10.1021/acs.jnatprod.3c00566. 37721602

[14] Q.‐Q. Zhou , X.‐Y. Xie , J.‐W. Zhu , et al., “Hosimosines A‐E, Structurally Diverse Cytisine Derivatives From the Seeds of *Ormosia hosiei* Hemsl. Et Wils,” Fitoterapia 170 (2023): 105661, 10.1016/j.fitote.2023.105661. 37648030

[15] M. Deng , Y. Xiao , S. Wang , et al., “Penicimides A and B, Two Novel Diels–Alder [4 + 2] Cycloaddition Ergosteroids From Penicillium Herquei,” Bioorganic Chemistry 143 (2024): 107025, 10.1016/j.bioorg.2023.107025. 38103332

[16] Y. Hu , Y. Saito , Y. Matsuo , X. Gong , and T. Tanaka , “Two New Dimeric Benzofuran Diastereomers From the Roots of *Eupatorium heterophyllum* ,” Tetrahedron Letters 102 (2022): 153924, 10.1016/j.tetlet.2022.153924.

[17] J. Sosa‐Rueda , V. Domínguez‐Meléndez , A. Ortiz‐Celiseo , et al., “Squamins C–F, Four Cyclopeptides from the Seeds of Annona Globiflora,” Phytochemistry 194 (2022): 112839, 10.1016/j.phytochem.2021.112839. 34332784

[18] E. M. Balboa , Y.‐X. Li , B.‐N. Ahn , et al., “Photodamage Attenuation Effect by a Tetraprenyltoluquinol Chromane Meroterpenoid Isolated From Sargassum Muticum,” Journal of Photochemistry and Photobiology B Biology 148 (2015): 51–58, 10.1016/j.jphotobiol.2015.03.026. 25874662

[19] J. C. C. Fuentes‐Monteverde , N. Nath , A. M. Forero , et al., “Connection of Isolated Stereoclusters by Combining 13C‐RCSA, RDC, and J‐Based Configurational Analyses and Structural Revision of a Tetraprenyltoluquinol Chromane Meroterpenoid From Sargassum Muticum,” Marine Drugs 20 (2022): 462, 10.3390/md20070462. 35877755 PMC9319238

[20] M. M. Zanardi , M. A. Sortino , and A. M. Sarotti , “On the Effect of Intramolecular H‐Bonding in the Configurational Assessment of Polyhydroxylated Compounds With Computational Methods. The Hyacinthacines Case,” Carbohydrate Research 474 (2019): 72–79, 10.1016/j.carres.2019.01.011. 30798018

[21] A. Navarro‐Vázquez , “When Not to Rely on Boltzmann Populations. Automated CASE‐3D Structure Elucidation of Hyacinthacines Through Chemical Shift Differences,” Magnetic Resonance in Chemistry 58 (2020): 139–144, 10.1002/mrc.4951. 31663628

[22] Y. Liu , A. Navarro‐Vázquez , R. R. Gil , C. Griesinger , G. E. Martin , and R. T. Williamson , “Application of Anisotropic NMR Parameters to the Confirmation of Molecular Structure,” Nature Protocols 14 (2019): 217–247, 10.1038/s41596-018-0091-9. 30552410

[23] N. Nath , M. Schmidt , R. R. Gil , et al., “Determination of Relative Configuration From Residual Chemical Shift Anisotropy,” Journal of the American Chemical Society 138 (2016): 9548–9556, 10.1021/jacs.6b04082. 27294984

[24] D. Joseph and C. Griesinger , “Optimal Control Pulses for the 1.2‐GHz (28.2‐T) NMR Spectrometers,” Science Advances 9 (2023): eadj1133, 10.1126/sciadv.adj1133. 37948513 PMC10637738

[25] M. Foroozandeh , R. W. Adams , N. J. Meharry , D. Jeannerat , M. Nilsson , and G. A. Morris , “Ultrahigh‐Resolution NMR Spectroscopy,” Angewandte Chemie International Edition 53 (2014): 6990–6992, 10.1002/anie.201404111. 24861024 PMC4320760

[26] C. Bruno de Sousa , K. N. Gangadhar , T. R. Morais , et al., “Antileishmanial Activity of Meroditerpenoids From the Macroalgae Cystoseira Baccata,” Experimental Parasitology 174 (2017): 1–9, 10.1016/j.exppara.2017.01.002. 28126391

[27] J. H. Simpson , Organic Structure Determination Using 2‐D NMR Spectroscopy (Elsevier, 2012), 21–57.

[28] R. Valls , L. Piovetti , B. Banaigst , and A. Praud , “Secondary Metabolites from Morocco Brown Algae of the Genus Cystoseira,” Phytochemistry 32 (1993): 961–966, 10.1016/0031-9422(93)85236-K.

[29] A. M. Torres , W. A. Bubb , and P. W. Kuchel , “Experiments to Detect Long‐Range Heteronuclear Shift Correlations: LR‐J‐HSMQC,” Journal of Magnetic Resonance 156 (2002): 249–257, 10.1006/jmre.2002.2563. 12165260

[30] V. V. Krishnamurthy , D. J. Russell , C. E. Hadden , and G. E. Martin , “2J,3J‐HMBC: A New Long‐Range Heteronuclear Shift Correlation Technique Capable of Differentiating 2JCH from 3JCH Correlations to Protonated Carbons,” Journal of Magnetic Resonance 146 (2000): 232–239, 10.1006/jmre.2000.2141. 10968978

[31] S. Gil , J. F. Espinosa , and T. Parella , “IPAP–HSQMBC: Measurement of Long‐Range Heteronuclear Coupling Constants From Spin‐State Selective Multiplets,” Magnetic Resonance (MR) 207 (2010): 312–321, 10.1016/j.jmr.2010.09.017. 20952232

[32] I. Alkorta and J. Elguero , “Essential Versus Accidental Isochrony of Diastereotopic Nuclei in NMR Spectroscopy,” Structural Chemistry 27 (2016): 671–679, 10.1007/s11224-015-0607-7.

[33] M. O. Marcarino , S. Cicetti , M. M. Zanardi , and A. M. Sarotti , “A Critical Review on the Use of DP4+ in the Structural Elucidation of Natural Products: The Good, the Bad and the Ugly. A Practical Guide,” Natural Product Reports 39 (2022): 58–76, 10.1039/D1NP00030F. 34212963

[34] A. F. Zuur , E. N. Ieno , and C. S. Elphick , “A Protocol for Data Exploration to Avoid Common Statistical Problems,” Methods in Ecology and Evolution 1 (2010): 3–14, 10.1111/j.2041-210X.2009.00001.x.

[35] J. Braun , P. Katzberger , G. A. Landrum , and S. Riniker , “Understanding and Quantifying Molecular Flexibility: Torsion Angular Bin Strings,” Journal of Chemical Information and Modeling 64 (2024): 7917–7924, 10.1021/acs.jcim.4c01513. 39390326 PMC11523068

[36] T. I. Oprea , “Property Distribution of Drug‐Related Chemical Databases*,” Journal of Computer‐Aided Molecular Design 14 (2000): 251–264, 10.1023/A:1008130001697. 10756480

[37] Y. Zhao and D. G. Truhlar , “The M06 Suite of Density Functionals for Main Group Thermochemistry, Thermochemical Kinetics, Noncovalent Interactions, Excited States, and Transition Elements: Two New Functionals and Systematic Testing of Four M06‐Class Functionals and 12 Other Functionals,” Theoretical Chemistry Accounts 120 (2008): 215–241, 10.1007/s00214-007-0310-x.

[38] M. Bursch , J. Mewes , A. Hansen , and S. Grimme , “Best‐Practice DFT Protocols for Basic Molecular Computational Chemistry,” Angewandte Chemie International Edition 61 (2022): e202205735, 10.1002/ange.202205735. 36103607 PMC9826355

[39] R. Todeschini and V. Consonni , Handbook of Molecular Descriptors (Wiley, 2000).

[40] A. Altis , P. H. Nguyen , R. Hegger , and G. Stock , “Dihedral Angle Principal Component Analysis of Molecular Dynamics Simulations,” Journal of Chemical Physics 126 (2007): 244111, 10.1063/1.2746330. 17614541

[41] E. Glimm and N. I. Fisher , Statistical Analysis of Circular Data (Cambridge University Press, 1993).

[42] P. J. Rousseeuw , “Silhouettes: A Graphical Aid to the Interpretation and Validation of Cluster Analysis,” Journal of Computational and Applied Mathematics 20 (1987): 53–65, 10.1016/0377-0427(87)90125-7.

[43] L. F. Gil‐Silva , R. Santamaría‐Fernández , A. Navarro‐Vázquez , and R. R. Gil , “Collection of NMR Scalar and Residual Dipolar Couplings Using a Single Experiment,” Chemistry – A European Journal 22 (2016): 472–476, 10.1002/chem.201503449. 26515991

[44] I. E. Ndukwe , M. V. S. Elipe , K. Quasdorf , et al., “Anisotropic Nuclear Magnetic Resonance Spectroscopy and Density Functional Theory Methodologies Combine With CASE‐3D Analysis for Unambiguous Diastereomeric Differentiation of AMG 176 Macrocycles,” Chemistry – A European Journal 3 (2025): e202500074, 10.1002/ceur.202500074.

[45] L. Venturi , E. Bua , G. Caputo , and V. Mileo , “Residual Dipolar Coupling Based Conformational Analysis Allows the Configurational Assessment of Steroids With Up to Eight Stereocenters,” ChemPlusChem 88 (2023): e202200391, 10.1002/cplu.202200391. 36811319

[46] J. B. Tuttle , C. Allais , C. M. N. Allerton , et al., “Discovery of Nirmatrelvir (PF‐07321332): A Potent, Orally Active Inhibitor of the Severe Acute Respiratory Syndrome Coronavirus 2 (SARS CoV‐2) Main Protease,” Journal of Medicinal Chemistry 68 (2025): 7003–7030, 10.1021/acs.jmedchem.4c02561. 40019854

[47] D. J. Wasilko , B. S. Gerstenberger , K. A. Farley , et al., “Structural Basis for CCR6 Modulation by Allosteric Antagonists,” Nature Communications 15 (2024): 7574, 10.1038/s41467-024-52045-7. PMC11365967 39217154

[48] Y. Liu , J. Saurí , E. Mevers , et al., “Unequivocal Determination of Complex Molecular Structures Using Anisotropic NMR Measurements,” Science 356 (2017): eaam5349, 10.1126/science.aam5349. 28385960 PMC6596297

[49] C. Gayathri , N. V. Tsarevsky , and R. R. Gil , “Residual Dipolar Couplings (RDCs) Analysis of Small Molecules Made Easy: Fast and Tuneable Alignment by Reversible Compression/Relaxation of Reusable PMMA Gels,” Chemistry – A European Journal 16 (2010): 3622–3626, 10.1002/chem.200903378. 20209530

[50] L. N. Wirz and J. R. Allison , “Fitting Alignment Tensor Components to Experimental RDCs, CSAs and RQCs,” Journal of Biomolecular NMR 62 (2015): 25–29, 10.1007/s10858-015-9907-x. 25652903

[51] F. Hallwass , R. R. Teles , E. Hellemann , C. Griesinger , R. R. Gil , and A. Navarro‐Vázquez , “Measurement of Residual Chemical Shift Anisotropies in Compressed Polymethylmethacrylate Gels. Automatic Compensation of Gel Isotropic Shift Contribution,” Magnetic Resonance in Chemistry 56 (2018): 321–328, 10.1002/mrc.4711. 29327368

[52] J. A. Losonczi , M. Andrec , M. W. F. Fischer , and J. H. Prestegard , “Order Matrix Analysis of Residual Dipolar Couplings Using Singular Value Decomposition,” Journal of Magnetic Resonance 138 (1999): 334–342, 10.1006/jmre.1999.1754. 10341140

[53] J. R. Tolman , J. M. Flanagan , M. A. Kennedy , and J. H. Prestegard , “NMR Evidence for Slow Collective Motions in Cyanometmyoglobin,” Natural Structural Biology 4 (1997): 292–297, 10.1038/nsb0497-292. 9095197

[54] A. Navarro‐Vázquez , “MSpin‐RDC. A Program for the Use of Residual Dipolar Couplings for Structure Elucidation of Small Molecules,” Magnetic Resonance in Chemistry 50 (2012): S73–S79, 10.1002/mrc.3905. 23280663

[55] L. Verdier , P. Sakhaii , M. Zweckstetter , and C. Griesinger , “Measurement of Long Range H,C Couplings in Natural Products in Orienting Media: A Tool for Structure Elucidation of Natural Products,” Journal of Magnetic Resonance 163 (2003): 353–359, 10.1016/S1090-7807(03)00063-6. 12914852

[56] N. Nath , E. J. d'Auvergne , and C. Griesinger , “Long‐Range Residual Dipolar Couplings: A Tool for Determining the Configuration of Small Molecules,” Angewandte Chemie International Edition 54 (2015): 12706–12710, 10.1002/anie.201504432. 26359945

[57] H. M. Ge , H. Sun , N. Jiang , et al., “Relative and Absolute Configuration of Vatiparol (1 mg): A Novel Anti‐Inflammatory Polyphenol,” European Journal 18 (2012): 5213–5221, 10.1002/chem.201104078. 22434621

[58] A. Das and N. Nath , “Elucidating Natural Product Structures Using a Robust Measurement of Carbon Residual Chemical Shift Anisotropy Combined With DFT,” Magnetic Resonance in Chemistry 59 (2021): 569–576, 10.1002/mrc.4975. 31758720

[59] W. Waratchareeyakul , E. Hellemann , R. R. Gil , K. Chantrapromma , M. K. Langat , and D. A. Mulholland , “Application of Residual Dipolar Couplings and Selective Quantitative NOE to Establish the Structures of Tetranortriterpenoids From Xylocarpus Rumphii,” Journal of Natural Products 80 (2017): 391–402, 10.1021/acs.jnatprod.6b00906. 28121439

[60] A. Kolmer , L. J. Edwards , I. Kuprov , and C. M. Thiele , “Conformational Analysis of Small Organic Molecules Using NOE and RDC Data: A Discussion of Strychnine and α ‐Methylene‐ γ ‐Butyrolactone,” Journal of Magnetic Resonance 261 (2015): 101–109, 10.1016/j.jmr.2015.10.007. 26556179

[61] H. Sun , U. M. Reinscheid , E. L. Whitson , et al., “Challenge of Large‐Scale Motion for Residual Dipolar Coupling Based Analysis of Configuration: The Case of Fibrosterol Sulfate A,” Journal of the American Chemical Society 133 (2011): 14629–14636, 10.1021/ja205295q. 21776994 PMC3173584

[62] M. Górecki , A. Suszczyńska , M. Woźnica , et al., “Chromane Helicity Rule—Scope and Challenges Based on an ECD Study of Various Trolox Derivatives,” Organic & Biomolecular Chemistry 12 (2014): 2235–2254, 10.1039/C3OB42376J. 24569389

[63] J. T. Vázquez , “Features of Electronic Circular Dichroism and Tips for Its Use in Determining Absolute Configuration,” Tetrahedron: Asymmetry 28 (2017): 1199–1211, 10.1016/j.tetasy.2017.09.015.

