# Supplementary material for: Anisotropic NMR as a Crucial Tool for Differentiation of Epimers With High Conformational Flexibility
Source: Angew Chem Int Ed Engl. 2026 May 25;65(30):e2111490. doi: 10.1002/anie.2111490 (PMC13382916; doi:10.1002/anie.2111490)
Supplement: Supplementary file 1 — The authors have cited additional references within the Supporting Information. Supporting File: anie72843‐sup‐0001‐SuppMat.docx. [file ANIE-65-e2111490-s001.docx]

***Supplementary Material***

Anisotropic NMR as a Crucial Tool for Differentiation of Epimers with High Conformational Flexibility

Juan Carlos C. Fuentes-Monteverde,^[a] [b] +^ Abel M. Forero,^[b] +^ Nilamoni Nath,^[c]^ Antonio Hernández Daranas, ^[d]^ Carlos Jiménez,^*[b]^ Jaime Rodríguez ^*[b]^ and Christian Griesinger *^[a]^

[a] Prof. Dr. C. Griesinger, Dr. J.C Fuentes-Monteverde
NMR based Structural Biology
MPI for Multidisciplinary Sciences
Am Fassberg 11, 37077 Göttingen, Germany
E-mail: cigr@mpinat.mpg.de

[b] Prof. Dr. J. Rodríguez, Prof. Dr. C Jiménez, Dr. A.M. Forero
CICA-Centro Interdisciplinar de Química e Bioloxía e Departamento de Química, Facultade de Ciencias
Universidade da Coruña
A Coruña, 15071, Spain
E-mail: jaime.rodriguez@udc.es; carlos.jimenez@udc.es

[c] Dr. N. Nath
Department of Chemistry
Gauhati University
Gopinath Bardoloi Nagar, Guwahati 781014, India

[d] Dr. A.H. Hernández Daranas
Instituto de Productos Naturales y Agrobiología. Consejo Superior de Investigaciones Científicas (IPNA-CSIC), San Cristóbal de La Laguna, 38206, Spain

[+] These authors contributed equally

| **List of acronyms** |
| --- |

**ECD:** Electrical circular dichroism

**OR:** Optical rotation

**SAM**: Sample mass

**NS**: Number of scans

**-OCP**: Denote pulse sequences where optimal control pulse was used

**NUS**: Non-uniform sampling. E.g.: 17.5%/45/512, indicates: 17.5% of points sampled, 45 number of complex points, and 512 increments in the indirect dimension. When no NUS parameter is indicated a traditional acquisition should be assumed.

**∆H_Q_**: Maximum ^2^H splitting (∆HQ)

**CDM**: Dichloromethane

**TCE**: 1,1,2,2-tetrachloroethane

**INDEX**

[**Figure S1.** Structure of meroditerpene **1a**; stereoclusters are indicated in blue (Chromane skeleton) and green (Hydrindane skeleton). 11](#_Toc230251134)

[**Figure S2.** Detection/isolation scheme of meroditerpene **1a** from Sargassum muticum by ^1^H-NMR and LC-HRMS. 11](#_Toc230251135)

[**Figure S3.** HPLC/FT-HRMS (FS mode) for the detection of the meroditerpenes (**1a** and **1b**) in the hexane fraction of *Sargassum muticum*. Total ion chromatogram **(a)**. Extracted mass chromatogram, from a selected mass range, showing a peak at a retention time of 14.35 min, assigned to the epimeric pair **1a/1b** (b). Expanded region of the (+)-HRESIMS of the peak at 14.35 min, identified as both meroditerpenes **1a** and **1b**. The peak (14.35 min) shows a characteristic [M+H]^+^ at m/z 441.2975 Da (^12^C Δ*m/z* = 5.5 Da; ^12^C_n_^13^C_1_ Δ*m/z* = 5.9 Da) (**c**). 12](#_Toc230251136)

[**Figure S4.** Semi-preparative RP-HPLC chromatogram of fraction 20 (eluted with Et_2_O/hexane 2:3) from the chromatography column of the hexane fraction of Sargassum muticum. The chromatogram shows the isolation of **1a** (t_R_ = 4.9 min). Conditions: Atlantis RP-C18 column 10x100 mm; Mobile phase (red line): 2.2 min isocratic step (80:20 ACN/H_2_O) and then a 15.8 min-gradient step (from 80:20 to 0:100 ACN/H_2_O)); flow rate: 4.6 mL/min; detection wavelength: 320 nm. 12](#_Toc230251137)

[**Figure S5.** ^1^H-NMR spectrum of compound **1a** (300 MHz, CD_2_Cl_2_) **(a-right)**. The inset shows the characteristic resonances of the aromatic protons of the chromane moiety in the tetraprenyltoluquinol meroterpenoids (6.561 ppm and 6.444 ppm). Both resonances were used to detect the presence of **1a** during its isolation (**a-left**). ESI-(+)-HRFTMS of compound **1a**; [M+H]^+^ (**b-i**), [M+Na]^+^ (**b-ii**) and [M-OH]^+^ (**b-iii**), all were detected are within the accepted *m*/*z* error (Δ *m*/*z* < 5 ppm). ^1^H-NMR 1D spectrum of chromatography column fraction 20 (Fr. 20) eluted with 17:8 Hex/Et_2_O (**c-right**). Inset shows the resonances of the aromatic protons and the methoxy group (-OMe) associated with the meroditerpenes found in *Sargassum muticum* (**c-left**). 13](#_Toc230251138)

[**Figure S6.** ECD and UV spectra of **1a** and **1b** recorded in DMSO. 14](#_Toc230251139)

[**Figure S7.** Main ^1^H-^13^C HMBC (Black arrows), and ^1^H-^1^H COSY (solid blue lines) correlations found in compound **1a.** Sample mass: 15 µg. 14](#_Toc230251140)

[**Figure S8.** ^1^H 1D NMR spectrum of compound **1a** (*lc1prf2*, C_2_D_2_Cl_4_, SAM: 15 µg, NS: 720, 800 MHz). 15](#_Toc230251141)

[**Figure S9.** ^1^H-^1^H-DQFCOSY spectrum of compound **1a** (cosyqf45, SAM: 15 µg, C_2_D_2_Cl_4_, NS: 320, 800 MHz). The spectrum on top is a 1D ^1^H spectrum. 16](#_Toc230251142)

[**Figure S10.** ^1^H-^13^C HSQC spectrum of compound **1a** (hsqcetgpsisp2.2-OCP, SAM: 15 µg, C_2_D_2_Cl_4_, NS: 1350, 800 MHz). Parameters: ^1^J_CH_=140 Hz. (NUS: 17.5%/45/512) (S/N: 306.3). The spectrum on top is a 1D ^1^H spectrum. 17](#_Toc230251143)

[**Figure S11.** A. ^1^H-^13^C HMBC spectrum of compound **1a** and B. Methyl region of compound **1a.** (hmbcetgpnd-OCP, SAM: 15 µg, C_2_D_2_Cl_4_, NS: 768, 800 MHz). Parameters: ^n^J_CH_ = 8 Hz. (NUS: 30%/153/1024) (S/N: 91.9). The spectrum on top is a 1D ^1^H spectrum. 18](#_Toc230251144)

[**Figure S12.** ^1^H 1D NMR spectrum of **1a** (zg, DMSO-d_6_, SAM: 1.5 mg, N S: 40, 1.2 GHz). 21](#_Toc230251145)

[**Figure S13.** Homodecoupled 1D ^1^H NMR spectrum of **1a** (zghd.2, DMSO-d_6_, SAM: 3 mg, NS: 16, 800 MHz), in each case, irradiated protons are indicated with *. 1D ^1^H selective homonuclear decoupling spectrum of **1a** upon irradiation of H9 (1.663 ppm). The resonances of H10a (1.8521 ppm) and H10b (1.2805 ppm) collapse into doublets (^2^J_HH_=11.0 Hz) **(a)**. 1D ^1^H selective homonuclear decoupling spectrum of **1a** upon irradiation of H1 (2.7180 ppm). The resonances of H2a (1.7752 ppm) and H2b (1.8076 ppm) collapse into doublets (^2^J_HH_=17.9 Hz) **(b)**. 21](#_Toc230251146)

[**Figure S14**. 1D ^13^C-{^1^H} NMR spectrum of **1a** (zgpg30, DMSO-d_6_, SAM: 1.5 mg, NS: 3K, 1.2 GHz) **(a)**. APT carbon spectrum of **1a** (jmod, DMSO-d_6_, SAM: 3 mg, NS: 8K, ^1^J_CH_ = 145 Hz, 800 MHz) **(b)**. 22](#_Toc230251147)

[**Figure S15.** 1D ^1^H NMR spectrum of epimeric mixture of **1a** and **1b (a).** Epimerization was induced by dissolving a pure sample of **1a** in 1,1,2,2-tetrachloroethane at room temperature for 7 days. As expected, all the resonances in the spectrum are duplicated. Duplicated resonance of MeO-C4’ (**b**) (zg30, DMSO-d_6_, NS: 32, 900 MHz). 22](#_Toc230251148)

[**Figure S16.** a) 2D NOESY spectrum of **1a** (noesyetgp, DMSO-d_6_, mixing time: 400 ms, NS: 48, NUS: 72%,184,512. 800 MHz). b) 2D NOESY spectrum of **1a** (noesyetgp, DMSO-d_6_, mixing time: 200 ms, NS: 48, NUS: 72%,184,512. 800 MHz). c) 1D NOE build-up curves of **1a** for protons H-14, H-6, Me17 and Me18. 23](#_Toc230251149)

[**Figure S17**. Selected NOESY contacts related to conformational equilibrium, of compound **1a**, and two possible conformers that satisfy the weak NOE contacts from H20 to H6b (noesyph, DMSO-d_6_, mixing time: 470 ms, NS: 32, 800 MHz). 25](#_Toc230251150)

[**Figure S18.** 2D ROESY spectrum of compound **1a** (roesyetgp, DMSO-d_6_, NS: 48. 800 MHz) 26](#_Toc230251151)

[**Figure S19.** 1D ^1^H PSYCHE pure-shift spectrum of **1a** (DMSO-d_6_, 800 MHz, NS 16, TD1: 32) (**a**). The inset shows resonances from 0.7 ppm to 1.2 ppm **(b)**. Resonances from 1.6 ppm to 1.9 ppm **(c)**. Collapsing of protons H2a (1.775 ppm), H2b (1.808 ppm), and H9 (1.659 ppm). Resonances from 2.7 ppm to 2.9 ppm **(d)**. The pure-shift experiment allows the straightforward assignment of the isochronic protons of H1. 27](#_Toc230251152)

[**Figure S20.** Comparison of the ^1^H 1D spectrum of **1a** (black) and **1b** (blue) **(a)**. The inset on the left shows the assignment of the methyl groups in both compounds (b). (zg, 800 MHz, DCM) 27](#_Toc230251153)

[**Figure S21.** 2D IPAP-HSQMBC-COSY spectrum (optimized to 5 Hz) after selective inversion of the H4b (2.870 ppm) proton of **1a (a)**. In the IPAP-HSQMBC-COSY, the α (red-blue) and β (yellow-green) multiplets for the cross-peaks involving C20, C2, C6, C3, C13, and C5 are shown. (hsqccoetgpiajclrndsp, DMSO-d_6_, NS: 72, NUS: 14%/35/512) 28](#_Toc230251154)

[**Figure S22.** 2D IPAP-HSQMBC spectrum (optimized to 7 Hz) after selective inversion of the H2a/H2b pair (1.808 and 1.775 ppm) of **1a**. In the figure, α (red-blue) and β (yellow-green) multiplets for the cross-peaks involving C1, C20, C4, C3, and C2’ are shown *(hsqcetgpiajclrndsp,* 900 MHz, DMSO-*d_6_*, NS: 64, NUS: 25%, 64, 512). 29](#_Toc230251155)

[**Figure S23.** 2D ROESY spectrum of **1b** in DMSO recorded at 1.2 GHz (**a**), and main ROE contacts observed (**b**). 31](#_Toc230251156)

[**Figure S24.** Computer-Assisted Structure Elucidation (CASE) study for the epimeric discrimination of **1a** **(a)** and **1b** **(b)** using NMR isotropic observables based on NMR isotropic data. 32](#_Toc230251157)

[**Figure S25**. DP4+ Bayesian probability analysis of (3S,7S,11R)-**1a** and (3R,7S,11R)-**1b** in CD_2_Cl_2_. 32](#_Toc230251158)

[**Figure S26.** Carbon (**left**) and proton (**right**) chemical shift correlation plot between Meroditerpene-**1a** and **-1b** in DMSO and CDM. The coefficient of correlation (R²) is shown in both cases. 33](#_Toc230251159)

[**Figure S27.** Chemical structure of the molecules used in the epimeric reciprocal assignment analysis. To expand the number of epimers in the epimeric reciprocal assignment, we introduce the concept of apparent epimers by NMR, referring to a situation within a set of diastereoisomers where two compounds are not epimers of each other, yet one becomes an epimer of the other when its mirror image is considered. For example, among the diastereoisomeric set 75a–75d, the pairs 75a–75d, 75a–75b, and 75c–75d are true epimers. However, 75a and 75c are not epimers themselves. Interestingly, the mirror image of 75c (ent-75c) forms an epimeric relationship with 75a, thereby creating a new, indirect pair of epimers. This concept is particularly useful when interpreting subtle NMR differences in stereoisomeric mixtures. 34](#_Toc230251160)

[**Figure S28.** Chemical shift correlation plot between experimental (δ) and DFT calculated (σ) epimers **73a** and **73b**. The coefficient of correlation (R²) is shown in both cases. 35](#_Toc230251161)

[**Figure S29.** Chemical shift correlation plot between experimental (δ) and DFT calculated (σ) epimers **74a** and **74b**. The coefficient of correlation (R²) is shown in both cases. 36](#_Toc230251162)

[**Figure S30.** Chemical shift correlation plot between experimental (δ) and DFT calculated (σ) epimers **75a** and **75b**. The coefficient of correlation (R²) is shown in both cases. 36](#_Toc230251163)

[**Figure S31.** Chemical shift correlation plot between experimental (δ) and DFT calculated (σ) epimers **75c** and **75d**. The coefficient of correlation (R²) is shown in both cases. 37](#_Toc230251164)

[**Figure S32.** Chemical shift correlation plot between experimental (δ) and DFT calculated (σ) of the apparent epimers **75a** and **75 *ent* c**. The coefficient of correlation (R²) is shown in both cases. 37](#_Toc230251165)

[**Figure S33.** Chemical shift correlation plot between experimental (δ) and DFT calculated (σ) epimers **75a** and **75d**. The coefficient of correlation (R²) is shown in both cases. 38](#_Toc230251166)

[**Figure S34.** Chemical shift correlation plot between experimental (δ) and DFT calculated (σ) epimers **75b** and **75c**. The coefficient of correlation (R²) is shown in both cases. 38](#_Toc230251167)

[**Figure S35**. Chemical shift correlation plot between experimental (δ) and DFT calculated (σ) epimers **75b** and **75 *ent* d**. The coefficient of correlation (R²) is shown in both cases. 39](#_Toc230251168)

[**Figure S36.** Chemical shift correlation plot between **75c** and **75d**. The coefficient of correlation (R²) is shown in both cases. 39](#_Toc230251169)

[**Figure S37.** Chemical shift correlation plot between experimental (δ) and DFT calculated (σ) epimers **77a** and **77b**. The coefficient of correlation (R²) is shown in both cases. 40](#_Toc230251170)

[**Figure S38**. Chemical shift correlation plot between experimental (δ) and DFT calculated (σ) epimers **77a** and **77 *ent* d**. The coefficient of correlation (R²) is shown in both cases. 40](#_Toc230251171)

[**Figure S39.** Chemical shift correlation plot between experimental (δ) and DFT calculated (σ) epimers **77a** and **77 *ent* d**. The coefficient of correlation (R²) is shown in both cases. 41](#_Toc230251172)

[**Figure S40.** Chemical shift correlation plot between experimental (δ) and DFT calculated (σ) epimers **77b** and **77d**. The coefficient of correlation (R²) is shown in both cases. 41](#_Toc230251173)

[**Figure S41.** Chemical shift correlation plot between experimental (δ) and DFT calculated (σ) epimers **78a** and **78b**. The coefficient of correlation (R²) is shown in both cases. 42](#_Toc230251174)

[**Figure S42.** Chemical shift correlation plot between experimental (δ) and DFT calculated (σ) epimers **78c** and **78d**. The coefficient of correlation (R²) is shown in both cases. 42](#_Toc230251175)

[**Figure S43**. Chemical shift correlation plot between experimental (δ) and DFT calculated (σ) epimers **78a** and **78c**. The coefficient of correlation (R²) is shown in both cases. 43](#_Toc230251176)

[**Figure S44.** Chemical shift correlation plot between experimental (δ) and DFT calculated (σ) epimers **78a** and **78d**. The coefficient of correlation (R²) is shown in both cases. 43](#_Toc230251177)

[**Figure S45.** Chemical shift correlation plot between experimental (δ) and DFT calculated (σ) epimers **78a** and **78e**. The coefficient of correlation (R²) is shown in both cases. 44](#_Toc230251178)

[**Figure S46**. Chemical shift correlation plot between experimental (δ) and DFT calculated (σ) epimers **78b** and **78c**. The coefficient of correlation (R²) is shown in both cases. 44](#_Toc230251179)

[**Figure S47.** Chemical shift correlation plot between experimental (δ) and DFT calculated (σ) epimers **78b** and **78d.** The coefficient of correlation (R²) is shown in both cases. 45](#_Toc230251180)

[**Figure S48.** Chemical shift correlation plot between experimental (δ) and DFT calculated (σ) epimers **78b** and **78 *ent* d**. The coefficient of correlation (R²) is shown in both cases. 45](#_Toc230251181)

[**Figure S49.** Chemical shift correlation plot between experimental (δ) and DFT calculated (σ) epimers **78a** and **78d**. The coefficient of correlation (R²) is shown in both cases. 46](#_Toc230251182)

[**Figure S50.** Chemical shift correlation plot between experimental (δ) and DFT calculated (σ) epimers **79a** and **79b**. The coefficient of correlation (R²) is shown in both cases. 46](#_Toc230251183)

[**Figure S51.** Chemical shift correlation plot between experimental (δ) and DFT calculated (σ) epimers **79c** and **79d**. The coefficient of correlation (R²) is shown in both cases. 47](#_Toc230251184)

[**Figure S52.** Chemical shift correlation plot between experimental (δ) and DFT calculated (σ) epimers **79e** and **79f**. The coefficient of correlation (R²) is shown in both cases. 47](#_Toc230251185)

[**Figure S53.** Chemical shift correlation plot between experimental (δ) and DFT calculated (σ) epimers **79f** and **79h**. The coefficient of correlation (R²) is shown in both cases. 48](#_Toc230251186)

[**Figure S54.** Chemical shift correlation plot between experimental (δ) and DFT calculated (σ) epimers **79g** and **79h**. The coefficient of correlation (R²) is shown in both cases. 48](#_Toc230251187)

[**Figure S55.** Chemical shift correlation plot between experimental (δ) and DFT calculated (σ) epimers **80a** and **80b**. The coefficient of correlation (R²) is shown in both cases. 49](#_Toc230251188)

[**Figure S56.** Chemical shift correlation plot between experimental (δ) and DFT calculated (σ) epimers **85a** and **85b**. The coefficient of correlation (R²) is shown in both cases. 49](#_Toc230251189)

[**Figure S57.** Chemical shift correlation plot between experimental (δ) and DFT calculated (σ) epimers **85c** and **85d** The coefficient of correlation (R²) is shown in both cases. 50](#_Toc230251190)

[**Figure S 58.** Chemical shift correlation plot between experimental (δ) and DFT calculated (σ) epimers **85b** and **85 *ent* d**. The coefficient of correlation (R²) is shown in both cases. 50](#_Toc230251191)

[**Figure S 59** Chemical shift correlation plot between experimental (δ) and DFT calculated (σ) epimers **85a** and **85 *ent* c**. The coefficient of correlation (R²) is shown in both cases. 51](#_Toc230251192)

[**Figure S60.** Chemical shift correlation plot between experimental (δ) and DFT calculated (σ) epimers **86a** and **86d.** The coefficient of correlation (R²) is shown in both cases. 51](#_Toc230251193)

[**Figure S61.** Chemical shift correlation plot between experimental (δ) and DFT calculated (σ) epimers **86b** and **86c.** The coefficient of correlation (R²) is shown in both cases. 52](#_Toc230251194)

[**Figure S 62.** Chemical shift correlation plot between experimental (δ) and DFT calculated (σ) epimers **86c** and **86 *ent* d**. The coefficient of correlation (R²) is shown in both cases. 52](#_Toc230251195)

[**Figure S63.** Chemical shift correlation plot between experimental (δ) and DFT calculated (σ) epimers **86a** and **86d.** The coefficient of correlation (R²) is shown in both cases. 53](#_Toc230251196)

[**Figure S64.** Chemical shift correlation plot between experimental (δ) and DFT calculated (σ) epimers **87b** and **87c.** The coefficient of correlation (R²) is shown in both cases. 53](#_Toc230251197)

[**Figure S 65.** Chemical shift correlation plot between experimental (δ) and DFT calculated (σ) epimers **87a** and **87 *ent* b**. The coefficient of correlation (R²) is shown in both cases. 54](#_Toc230251198)

[**Figure S66.** Chemical shift correlation plot between experimental (δ) and DFT calculated (σ) epimers **87c** and **87 *ent* d**. The coefficient of correlation (R²) is shown in both cases. 54](#_Toc230251199)

[**Figure S67.** ^1^H 1D NMR spectrum of compound **1b** (zg, DMSO-d_6_, NS: 80, 1.2 GHz). The inset (1.60-1.85 ppm) shows the comparison between both spectra: the ^1^H 1D and the PSYCHE pure-shift (800 MHz). 55](#_Toc230251200)

[**Figure S68.** ^13^C-{^1^H} NMR spectrum of **1b** (zgpg30, DMSO-d_6_, NS: 8K, 1.2 GHz). 55](#_Toc230251201)

[**Figure S69**. ^1^H-^13^C HSQC spectrum of compound **1b** (hsqcetgp, DMSO-d_6_, NS: 16, 800 MHz). Parameters: ^1^J_CH_ =138 Hz. (NUS: 15.6%/40/512). The spectrum on top is a 1D ^1^H spectrum, and the spectrum on the left is a carbon-proton decoupled spectrum. 56](#_Toc230251202)

[**Figure S70.** ^1^H-^13^C HMBC spectrum of compound **1b** (hmbcetgpl3nd, DMSO, NS: 24, 800 MHz). Parameters: ^1^J_CH_ (Min) = 120 Hz; ^1^J_CH_ (Max) = 168 Hz; ^n^J_CH_ (Min) = 8 Hz; (NUS: 25%/64/512). The spectrum on top is a 1D ^1^H spectrum, and the spectrum on the left is a carbon-proton decoupled spectrum. 57](#_Toc230251203)

[**Figure S71.** 2D ROESY spectrum of compound **1a** (roesyphpp.2, DMSO-d_6_, NS: 80; mixing time: 200 ms; 1.2 GHz). The inset shows the assignment of the relative orientation of protons H8a, H9a, and H10a to Me19, thanks to ROE contacts. The spectrum on top and on the left is a 1D ^1^H spectrum, 58](#_Toc230251204)

[**Figure S72.** Workflow to determine the conformational landscape. 60](#_Toc230251205)

[**Figure S73.** Heatmap of the RMSD analysis of the DFT-optimized set of the SSR-**1a** conformational pool. The color scale indicates the RMSD values (Å). A hierarchical dendrogram is shown at the top and on the left, where grouping was achieved using the Unweighted Pair Group Method with Arithmetic Mean. Conformers labeled as redundant are colored blue (Approx. RMSD < 0.5 Å). 61](#_Toc230251206)

[**Figure S74.** Heatmap of the RMSD analysis of the DFT-optimized set of the RSR-**1b** conformational pool. The color scale indicates the RMSD values (Å). A hierarchical dendrogram is shown at the top and on the left, where grouping was achieved using the Unweighted Pair Group Method with Arithmetic Mean. Conformers labeled as redundant are colored blue (Approx. RMSD < 0.5 Å). 62](#_Toc230251207)

[**Figure S75.** Heatmap of the RMSD analysis of the DFT-optimized set of the SSR-**1a** conformational space. The color scale indicates the RMSD values (Å). A hierarchical dendrogram is shown at the top and on the left, where grouping was achieved using the Unweighted Pair Group Method with Arithmetic Mean. 63](#_Toc230251208)

[**Figure S76.** Heatmap of the RMSD analysis of the DFT-optimized set of the RSR-**1b** conformational space. The color scale indicates the RMSD values (Å). A hierarchical dendrogram is shown at the top and on the left, where grouping was achieved using the Unweighted Pair Group Method with Arithmetic Mean. 63](#_Toc230251209)

[**Figure S77.** Histograms showing the dihedral angle distribution among the representative conformers of SSR-**1a**. After inspection of D(H14-C14-C15-C16) and D(H14-C14-C15-C17), its redundancy becomes evident. 64](#_Toc230251210)

[**Figure S78.** Histograms showing the dihedral angle distribution among the representative conformers of *RSR-***1b**. After inspection of D(41-11-18-19) and D(41-11-18-21), its redundancy becomes evident. 64](#_Toc230251211)

[**Figure S79.** Correlation matrix among molecular and torsional descriptors of *SSR*-**1a** conformers. The level of correlation is expressed as the Pearson correlation coefficient. The correlation between dihedrals becomes evident upon visual inspection of the matrix. Dihedrals H14-C14-C15-C16 and H14-C14-C15-C17 are perfectly correlated (PCC = 1.00), meaning that they redundantly contribute to the description of the molecular landscape. Interestingly, dihedrals H14-C14-C13-C5 and H4-C4-C5-C14 appear to be highly correlated (PCC = 0.90), which can be attributed to steric hindrance within the molecular geometry. 65](#_Toc230251212)

[**Figure S80.** Correlation matrix among molecular and geometrical descriptors of conformers of *SSR*-**1a**, expressed in Pearson correlation coefficient (PCC), after dihedral H14-C14-C15-C17 removal. 65](#_Toc230251213)

[**Figure S81.** Correlation matrix among molecular and torsional descriptors of RSR-**1b** conformers. The level of correlation is expressed as the Pearson correlation coefficient (PCC). The correlation between dihedrals becomes evident upon visual inspection of the matrix. Dihedrals H14-C14-C15-C16 and H14-C14-C15-C17 are perfectly correlated (PCC = 1.00), meaning that they redundantly contribute to the description of the molecular landscape. Interestingly, dihedrals H14-C14-C13-C5 and H4-C4-C5-C14 appear to be highly correlated (PCC = 0.89), which can be attributed to steric hindrance within the molecular geometry. 66](#_Toc230251214)

[**Figure S82**. Correlation matrix among molecular and geometrical descriptors of conformers of RSR-**1b**, expressed in Pearson correlation coefficient (PCC), after dihedral H14-C14-C15-C17 removal. 66](#_Toc230251215)

[**Figure S83.** PCA of the conformational landscape of **1b** (*RSR*) based on significant dihedral and torsional descriptors and other molecular descriptors. The optimal number of groups was determined using the silhouette method and structural relevance. **Cluster 0**: *RSR*-Conf-45, *RSR*-Conf-48; **Cluster 1:** *RSR*-Conf-3, *RSR*-Conf-52, *RSR*-Conf-4, *RSR*-Conf-10, *RSR*-Conf-39, and **Cluster 2:** *RSR*-Conf-18, *RSR*-Conf-11, and RSR-Conf-17 67](#_Toc230251216)

[**Figure S84.** 1D ¹³C-{¹H} NMR spectrum of **1a** oriented in a Poly-HEMA matrix (DMSO-*d_6_*; 5 mm compression device, NS: 32k) measured at 600 MHz (a). Bar plot derived from the ¹³C-RCSA analysis of 1a in a 5 mm compression device (Poly-HEMA/DMSO) recorded at 600 MHz (b). Error bars, derived from the Monte Carlo error analysis, are shown. This analysis was carried out with a sample size of 10 K, assuming a normally distributed error. The average experimental error for the ¹³C RCSA was estimated to be 1.37 ppb. 68](#_Toc230251217)

[**Figure S85**. The 3 mm Semi-Micro Compression Device and its components. 69](#_Toc230251218)

[**Figure S86.** Technical drawings of the 3 mm Semi-Micro Compression Device. 72](#_Toc230251219)

[**Figure S87.** Relaxed micro gel stick of Poly-HEMA swollen in a sample of 1a in DMSO-d₆ **(a).** Ideally, the gel stick for the compression device should be 45 mm long. After compression, the gel stick shortens from 45 mm to 34 mm **(b)** and is then ready for anisotropic NMR experiments. The Semi-Micro Compression Device, where the lock nut and the base-body nut are kept tight by being wrapped in Parafilm paper **(c)**, prevents tiny changes in the level of alignment during NMR experiments. 73](#_Toc230251220)

[**Figure S88.** 1D ²H spectrum of DMSO-d₆ in a 3 mm compression device recorded on a 1.2 GHz Bruker spectrometer (zg2h, Poly-HEMA, NS = 8). The experiment in the relaxed state of the gel sample is shown in blue (a). Differences in magnetic susceptibility between DMSO-d₆ inside () and outside (*) the alignment medium become evident by observing two deuterium signals. The spectrum of the sample after compression is shown in red (b). The ²H residual quadrupolar coupling observed for the analysis of 1a was 6.0 Hz. Interestingly, a tiny deuterium signal from outside the gel stick is still visible (black arrow) 74](#_Toc230251221)

[**Figure S89** ¹³C-{¹H} NMR spectrum of **1a** oriented in a Poly-HEMA matrix (DMSO-d_6_) using a 3 mm semi-micro compression device. The experiment was recorded on a 1.2 GHz spectrometer with 32 K. 76](#_Toc230251222)

[**Figure S90** ^1^H-^13^C F1-resolved HSQC (hsqcbietgpjcsp.2) spectrum of **1a** oriented in Poly-HEMA (DMSO), recorded on a 1.2 GHz spectrometer using a 3 mm semi micro compression device. Experiment parameters: NS: 72; ^1^J_CH_ = 140 Hz; scaling factor = 6. The stripes along F2 are baseline humps from the gel, which have not been removed since they don’t overlap with peaks of interest. 77](#_Toc230251223)

[**Figure S91** ¹³C-{¹H} NMR spectrum of **1b** oriented in a Poly-HEMA matrix (DMSO-d_6_) using a 3 mm semi-micro compression device. The experiment was recorded on a 1.2 GHz spectrometer with 16 K. 78](#_Toc230251224)

[**Figure S92** ^1^H-^13^C F1-resolved HSQC (hsqcbietgpjcsp.2) spectrum of **1b** oriented in Poly-HEMA (DMSO), recorded on a 1.2 GHz spectrometer using a 3 mm semi micro compression device. Experiment parameters: NS: 64; ^1^J_CH_ = 140 Hz; scaling factor = 6. 79](#_Toc230251225)

[**Figure S93.** RDC-based multiple-tensor multi-conformer analysis workflow used for configurational assignment of Meroditerpene**-1a** and **-1b**. Conformers after DFT optimization are selected that show the least violations of experimental isotropic NMR parameters based on CASE approach. 110](#_Toc230251226)

[**Figure S94.** Cornilescu’s quality factor (Q) analyses of the MTMC RDC-based analysis for **1a** (right) and **1b** (left). 110](#_Toc230251227)

[**Figure S95.** Conformer overlapping of geometries used in the multi tensor multi conformer analysis of the stereochemical assignment of **1a** (right) and **1b** (left). Conformers were constrained using quantitative NOEs, ^2,3^J_CH,_ and ^1^H/^13^C chemical shift. 112](#_Toc230251228)

[**Figure S96.** Bar plots from the CASE study of **1a** and **1b**, from which conformer populations were derived. The analysis was based on isotropic NMR constraints, including quantitative NOEs, ^2,3^*J*_CH_ couplings, and ^1^H/^13^C chemical shifts. The resulting population estimates are reported in **Table S13** and were used in the MTMC analysis. 112](#_Toc230251229)

[**Figure S97.** Anisotropic ^13^C-^1^H CLIP/CLAP-HSQC experiment (hsqcetgpiajcsp.2) of **1a** (i) and **1b** (ii), swollen in poly-HEMA in a 3 mm compression device, recorded in a 1.2 GHz Bruker spectrometer. Both anisotropic NMR experiments were recorded with a ^2^H quadrupolar splitting of 6.1 Hz. Spectra acquisition time was sped up by implementing NUS (512/39/97). 113](#_Toc230251230)

[**Figure S98.** Correlation plot of the experimental and back-calculated RDCs derived from the ^1^*D*_CH_-MTMC fitting of **1a** (Left column) and **1b** (Right column). 115](#_Toc230251231)

[**Figure S99.** The bootstrapping error analysis for the MTMC analysis of both possible configurations: 3S,7S,11R-**1a** (a) and 3R,7S,11R-**1a** (b), using an RDC experimental error of 0.16 Hz. 115](#_Toc230251232)

[**Figure S100.** The bootstrapping error analysis for the MTMC analysis of both possible configurations: 3*R*,7*S*,11*R*-**1b** (a) and 3*S*,7*S*,11*R*-**1b** (b), using an RDC experimental error of 0.18 Hz. 116](#_Toc230251233)

[**Figure S101.** Monte Carlo Error Propagation Analysis in the ^13^C-RCSA-Based Discrimination of **1a**-SSR/RSR Epimers. The simulation was performed by independently propagating the uncertainty associated with each variable in the QCSA equation, assuming Gaussian error distributions. A total of 500,000 random samples were generated within a ±3σ range to cover 99.7% of the expected variation. The resulting plot displays the mean QCSA value, its standard deviation, and the corresponding 95% confidence interval. 126](#_Toc230251234)

[**Figure S102.** Monte Carlo Error Propagation Analysis in the ^13^C-RCSA-Based Discrimination of **1b**-SSR/RSR Epimers. The simulation was performed by independently propagating the uncertainty associated with each variable in the QCSA equation, assuming Gaussian error distributions. A total of 500,000 random samples were generated within a ±3σ range to cover 99.7% of the expected variation. The resulting plot displays the mean QCSA value, its standard deviation, and the corresponding 95% confidence interval. 127](#_Toc230251235)

[**Figure S103.** Molecular structures of the compounds used to determine the absolute error and standard deviation associated with the axial chemical shift anisotropy. 128](#_Toc230251236)

[**Figure S104.** Correlation plot between the absolute value of the experimental and computed axial chemical shift anisotropy of several carbon atoms (│CSAax,i^cacl.^│). Theoretical CSAax values were computed at DFT level using mPW1PW91/6311+(2d,p) iefpcm = dmso. 129](#_Toc230251237)

[**Figure S105.** Individual ECD spectra of the derived conformers from anisotropic data were used to absolute configuration of **1a.** 130](#_Toc230251238)

| **General Methods** |
| --- |

^1^H, ^13^C, and 2D NMR spectra were recorded on Bruker spectrometers at various magnetic fields, including 800, 600, 500, and 300 MHz equipped with cryogenic probes (3 mm or 5 mm), using NEO or AVANCE III/HD consoles. Spectra were referenced to residual solvent signals: DMSO-*d_6_*, CDCl_3_, or CD_2_Cl_2_ as appropriate. Spectral assignments were based on COSY, HSQC, HMBC, NOESY, ROESY, and HECADE experiments, processed with TopSpin software.

Anisotropic sample: RDC and RCSA experiments were done in a 1.2 GHz Bruker spectrometer equipped with a 3 mm Cryogenic probe equipped. Description of sample preparation using the 3 mm compression device can be found on YouTube (<https://www.youtube.com/@juancarlosfuentes-montever3057>)

| **Compound 1a** |
| --- |

Hydrindane moiety


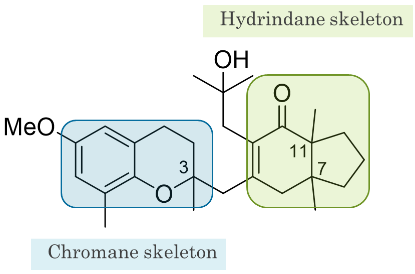


Chromane moiety

**Figure S1.** Structure of meroditerpene **1a**; stereoclusters are indicated in blue (Chromane skeleton) and green (Hydrindane skeleton).

RP-HPLC-UV

**Figure S2.** Detection/isolation scheme of meroditerpene **1a** from Sargassum muticum by ^1^H-NMR and LC-HRMS.

**(a)**

**(b)**

**(c)**

**Figure S3.** HPLC/FT-HRMS (FS mode) for the detection of the meroditerpenes (**1a** and **1b**) in the hexane fraction of *Sargassum muticum*. Total ion chromatogram **(a)**. Extracted mass chromatogram, from a selected mass range, showing a peak at a retention time of 14.35 min, assigned to the epimeric pair **1a/1b** (b). Expanded region of the (+)-HRESIMS of the peak at 14.35 min, identified as both meroditerpenes **1a** and **1b**. The peak (14.35 min) shows a characteristic [M+H]^+^ at m/z 441.2975 Da (^12^C Δ*m/z* = 5.5 Da; ^12^C_n_^13^C_1_ Δ*m/z* = 5.9 Da) (**c**).

**Solvent profile**

**1a**

**Figure S4.** Semi-preparative RP-HPLC chromatogram of fraction 20 (eluted with Et_2_O/hexane 2:3) from the chromatography column of the hexane fraction of Sargassum muticum. The chromatogram shows the isolation of **1a** (t_R_ = 4.9 min). Conditions: Atlantis RP-C18 column 10x100 mm; Mobile phase (red line): 2.2 min isocratic step (80:20 ACN/H_2_O) and then a 15.8 min-gradient step (from 80:20 to 0:100 ACN/H_2_O)); flow rate: 4.6 mL/min; detection wavelength: 320 nm.

**Compound 1a**

Compound **1a** was isolated as a brownish oil; UV (DMSO) λ_max_ (log ε) 293, 303; $\left[ \alpha\right]_{D}^{23}$= +25.5 (DCM) and +43.5 (DMSO) degrees; ^1^H NMR and ^13^C NMR **Table S1** and **Table S*2***; ESIHRMS *m/z* 441.2999 [M + H ]+ (Calc. for C_28_H_41_O_4_^+^, 441.2999); *m/z* 463.2813 [M + H ]+ (Calc. for C_28_H_40_NaO_4_^+^, 463.2819); and *m/z* 423.2888 [M - OH]+ (Calc. for C_28_H_40_NaO_4_^+^, 423.2894).

| 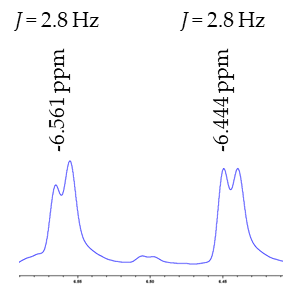  **(a)** | 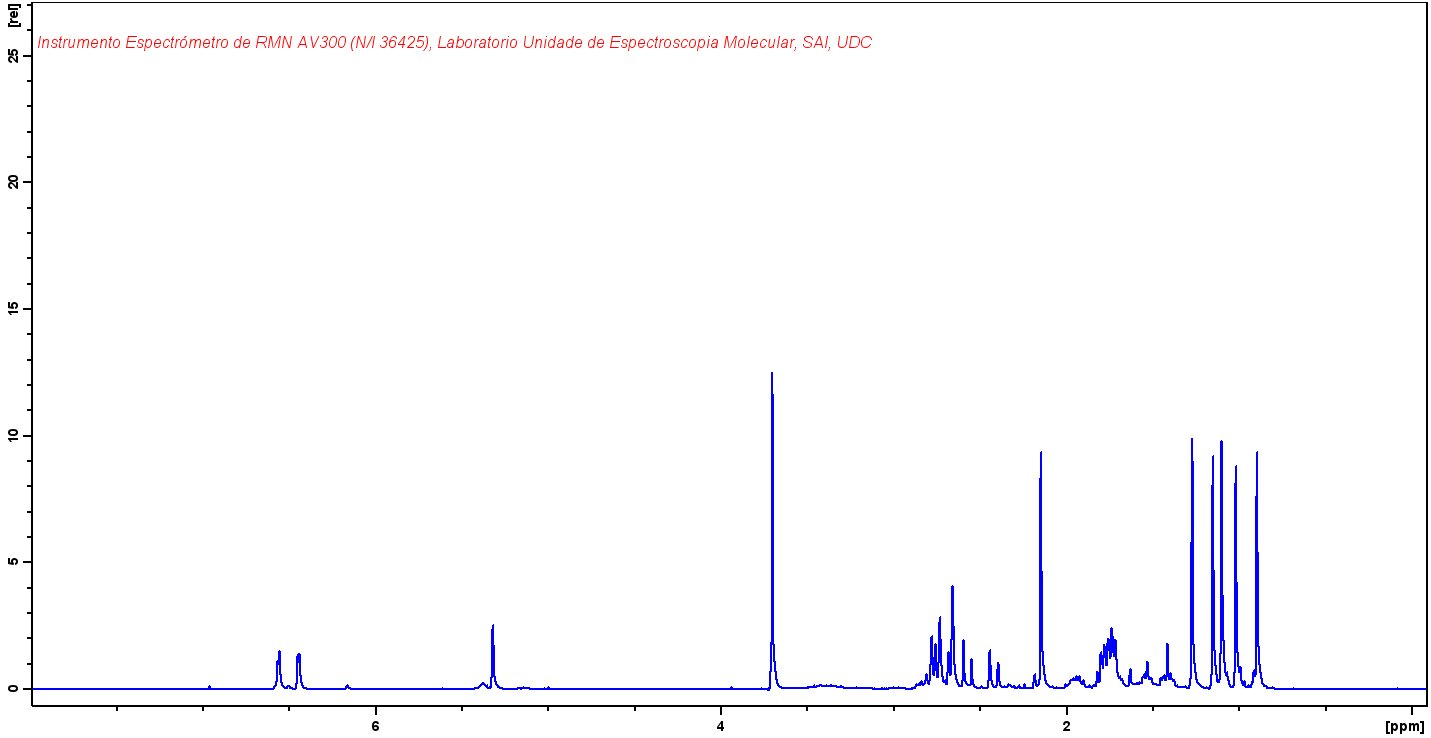 |
| --- | --- |
| 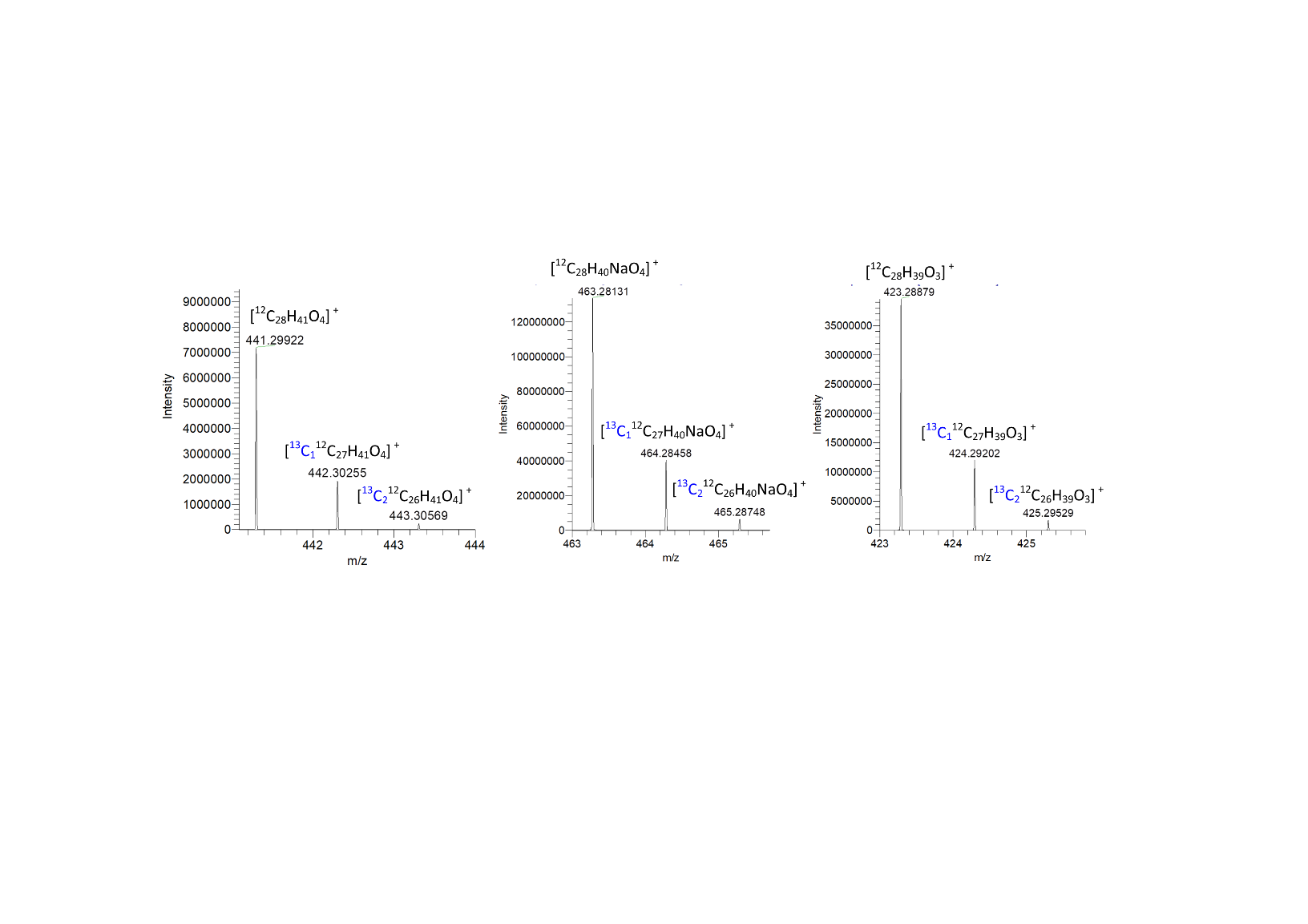  (*iii*)  **(b)**  (*ii*)  (*i*) | |
| 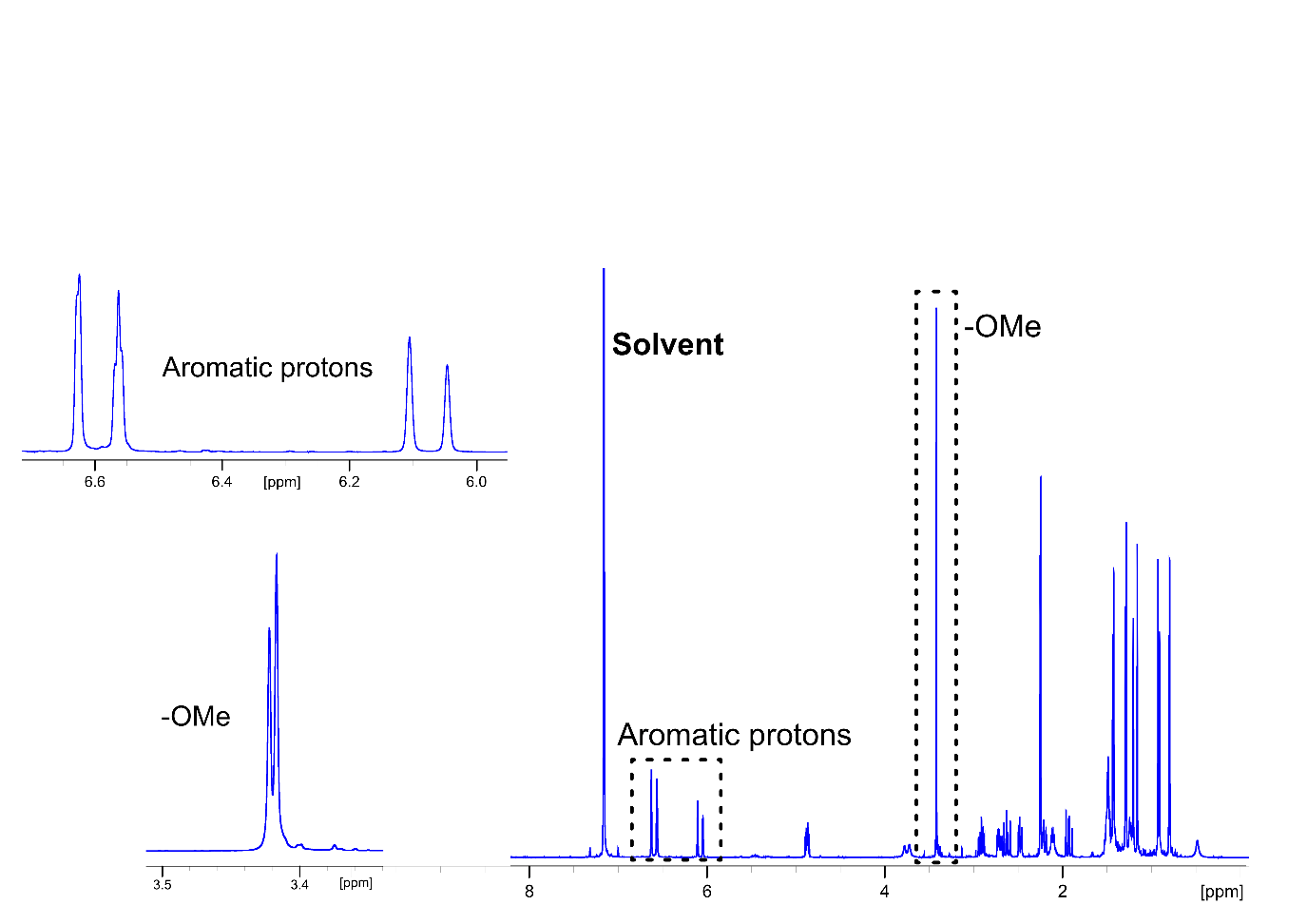  **(c)** | |
| **Figure S5.** ^1^H-NMR spectrum of compound **1a** (300 MHz, CD_2_Cl_2_) **(a-right)**. The inset shows the characteristic resonances of the aromatic protons of the chromane moiety in the tetraprenyltoluquinol meroterpenoids (6.561 ppm and 6.444 ppm). Both resonances were used to detect the presence of **1a** during its isolation (**a-left**). ESI-(+)-HRFTMS of compound **1a**; [M+H]^+^ (**b-i**), [M+Na]^+^ (**b-ii**) and [M-OH]^+^ (**b-iii**), all were detected are within the accepted *m*/*z* error (Δ *m*/*z* < 5 ppm). ^1^H-NMR 1D spectrum of chromatography column fraction 20 (Fr. 20) eluted with 17:8 Hex/Et_2_O (**c-right**). Inset shows the resonances of the aromatic protons and the methoxy group (-OMe) associated with the meroditerpenes found in *Sargassum muticum* (**c-left**). | |


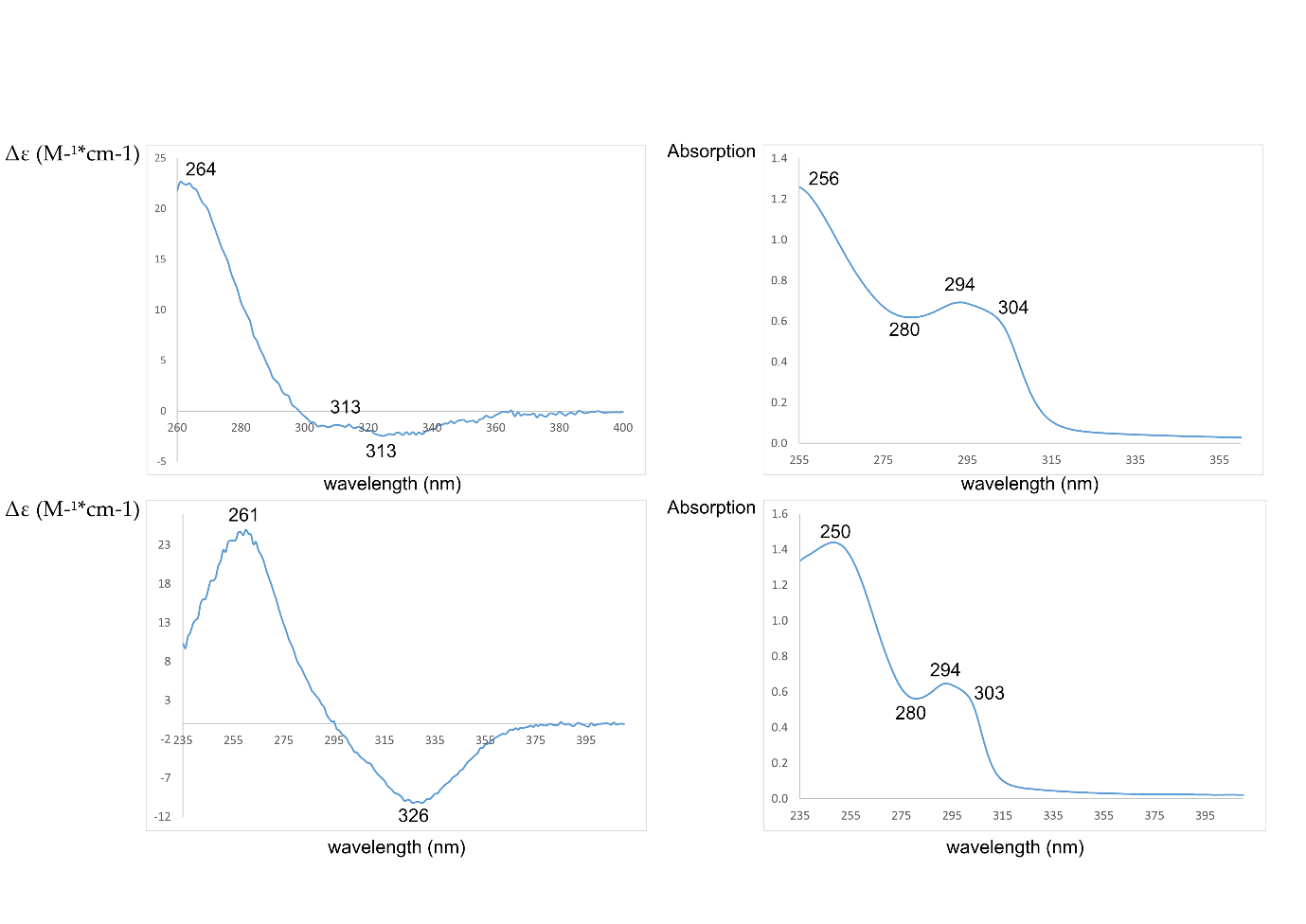


**1b**

**1a**

**UV spectrum**

**ECD spectrum**

**Figure S6.** ECD and UV spectra of **1a** and **1b** recorded in DMSO.

| **Compound 1a (15-µg in TCE in a 1.7 mm NMR capillary tube.)** |
| --- |

**Sample preparation:** The 15-µg sample was prepared in TCE by serial dilution of a stock solution (1 mg/mL) and analyzed in a 1.7 mm NMR capillary tube.

**
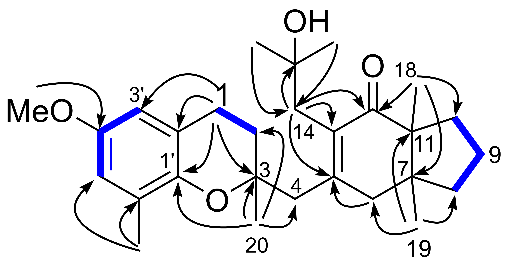
**

**Figure S7.** Main ^1^H-^13^C HMBC (Black arrows), and ^1^H-^1^H COSY (solid blue lines) correlations found in compound **1a.** Sample mass: 15 µg.


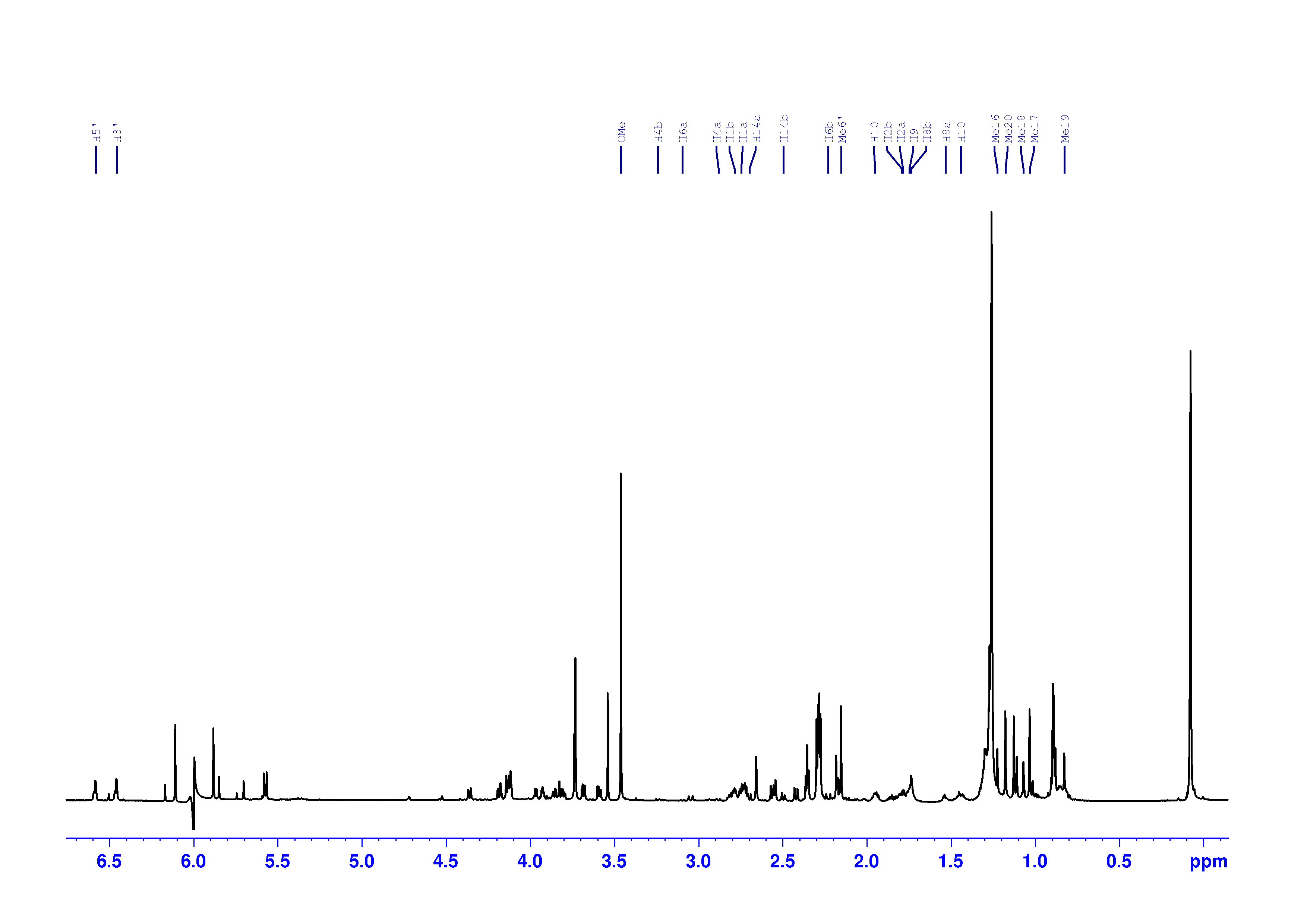


**Figure S8.** ^1^H 1D NMR spectrum of compound **1a** (*lc1prf2*, C_2_D_2_Cl_4_, SAM: 15 µg, NS: 720, 800 MHz).


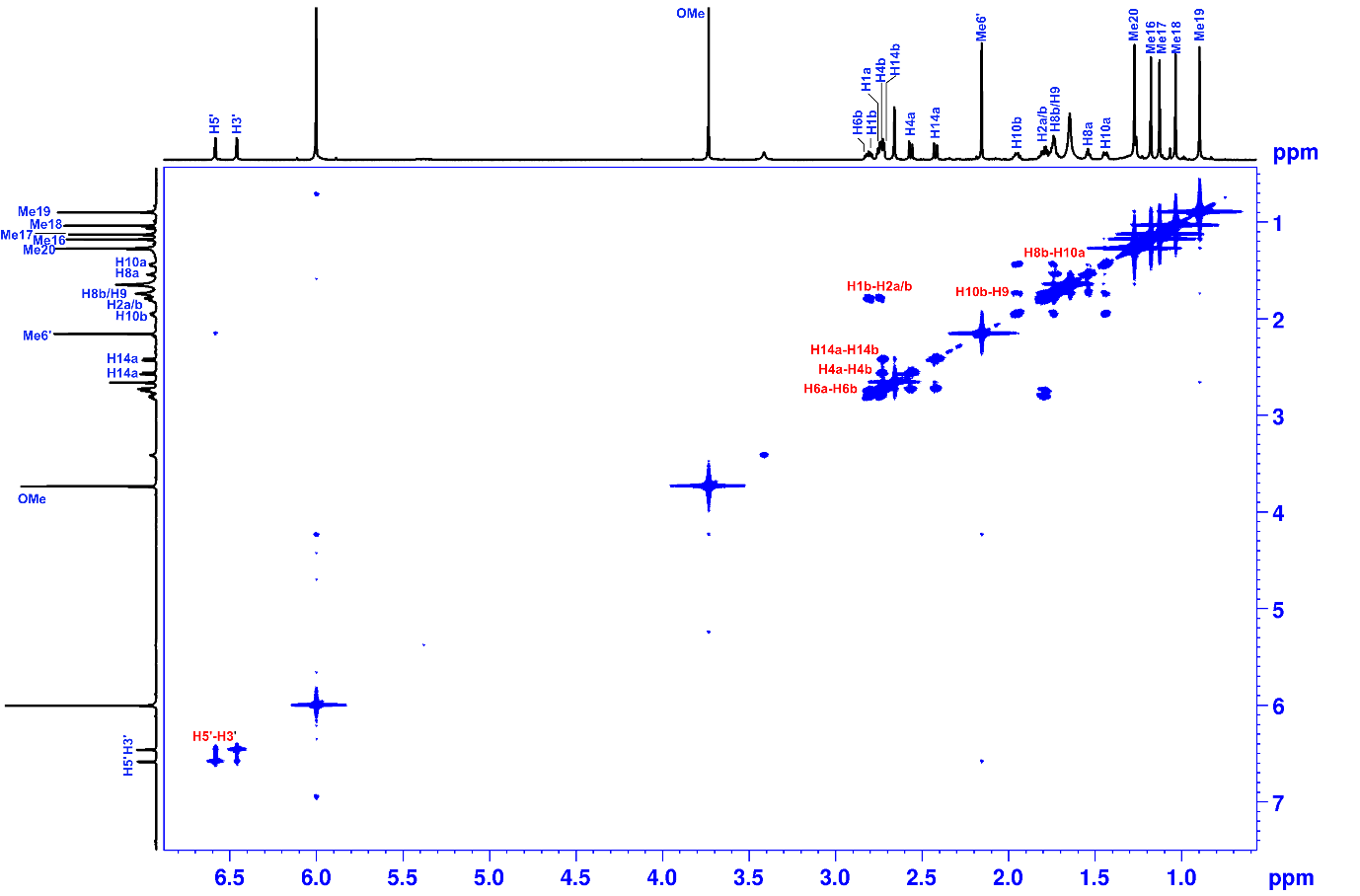


**Figure S9.** ^1^H-^1^H-DQFCOSY spectrum of compound **1a** (cosyqf45, SAM: 15 µg, C_2_D_2_Cl_4_, NS: 320, 800 MHz). The spectrum on top is a 1D ^1^H spectrum.


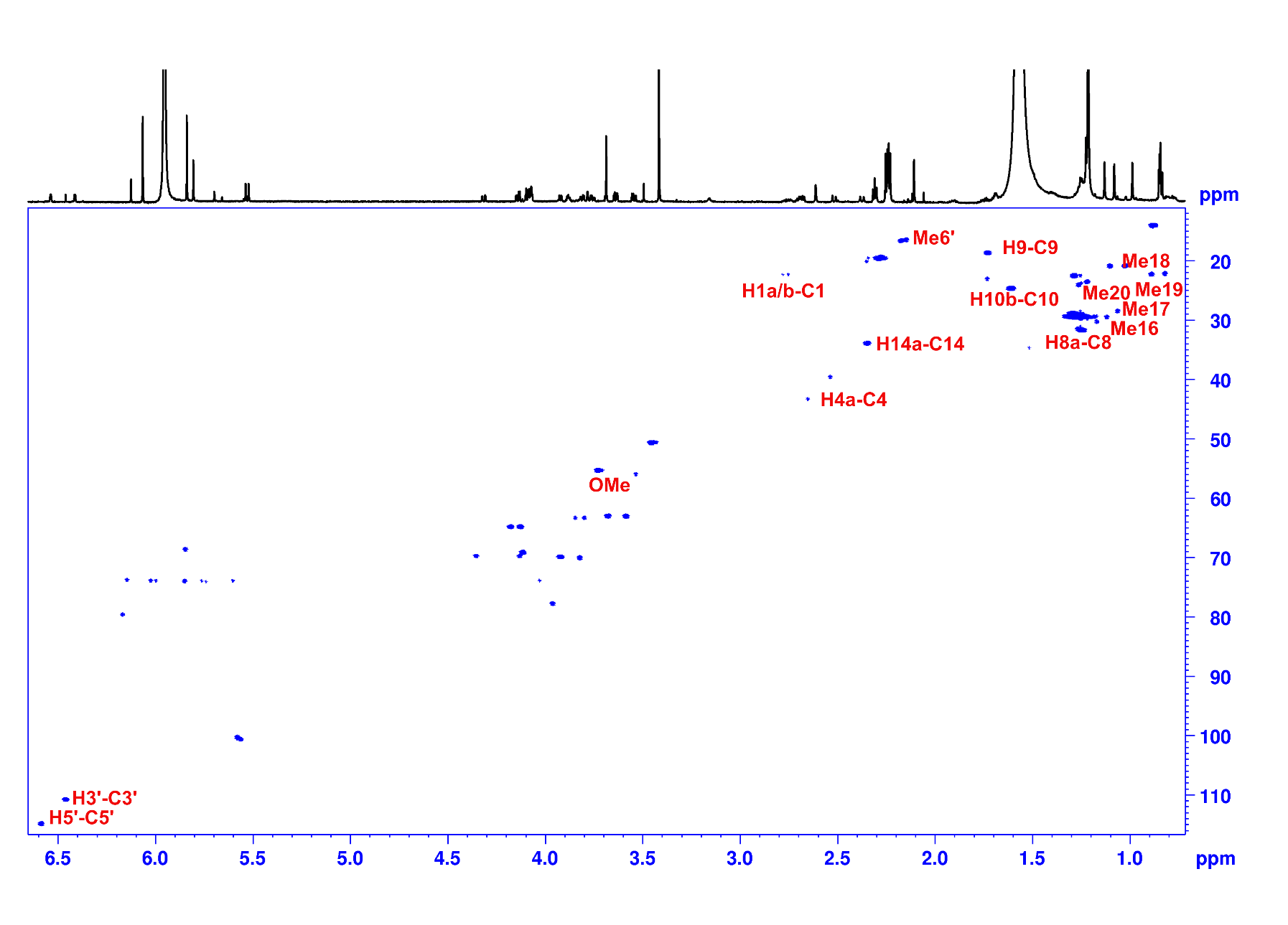


**Figure S10.** ^1^H-^13^C HSQC spectrum of compound **1a** (hsqcetgpsisp2.2-OCP, SAM: 15 µg, C_2_D_2_Cl_4_, NS: 1350, 800 MHz). Parameters: ^1^J_CH_=140 Hz. (NUS: 17.5%/45/512) (S/N: 306.3). The spectrum on top is a 1D ^1^H spectrum.


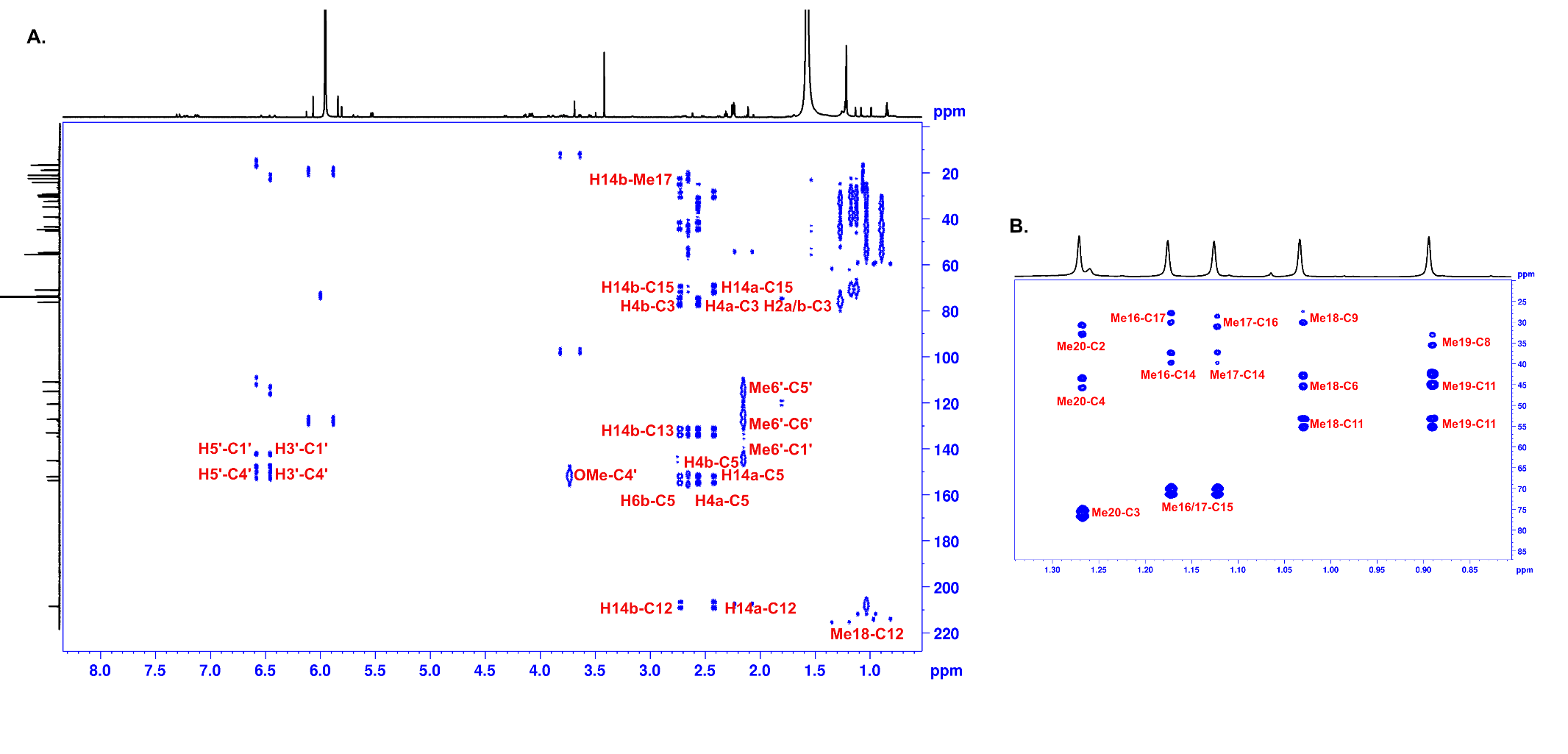


**Figure S11.** A. ^1^H-^13^C HMBC spectrum of compound **1a** and B. Methyl region of compound **1a.** (hmbcetgpnd-OCP, SAM: 15 µg, C_2_D_2_Cl_4_, NS: 768, 800 MHz). Parameters: ^n^J_CH_ = 8 Hz. (NUS: 30%/153/1024) (S/N: 91.9). The spectrum on top is a 1D ^1^H spectrum.

**Table S1.** NMR spectroscopic data for compound **1a** and **1b** (DMSO-d_6_).

| **Position** | **1a** | | **1b** | |
| --- | --- | --- | --- | --- |
|  | ***δ*_C_, mult. ^a,b^** | ***δ*_H_, mult., *J* (in Hz) ^c^** | ***δ*_C_, mult. ^a,b^** | ***δ*_H_, mult., *J* (in Hz) ^c^** |
| 1 | 22.03 CH_2_ | 2.7131 (*t*, 6.7) | 21.96 CH_2_ | 2.7156 (*t*, 6.9) |
| 2* | 32.76 CH_2_ | Ha: 1.7739 (*dt*, 13.4, 6.7) | 32.05 CH_2_ | 1.7760 (*dt*, 12.7, 6.9)  1.7852 (*dt,* 12.7, 6.9) |
|  |  | Hb: 1.8013 (*dt*, 13.4, 6.7) |  |  |
| 3 | 76.44 qC | - | 76.42 qC | - |
| 4 | 42.61 CH_2_ | H4a: 2.4633 (*d*, 14.1) | 43.80 CH_2_ | H4a: 2.6427 (d, 14.0) |
|  |  | H4b: 2.8701 (*d*, 14.1) |  | H4b: 2.7850 (d, 14.0) |
| 5 | 152.09 qC | - | 152.84 qC | - |
| 6 | 41.78 CH_2_ | H6b: 2.3824 (*d*, 18.3) | 43.20 CH_2_ | H6a: 2.3186 (*d*, 18.5) |
|  |  | H6a: 2.7734 (*d*, 18.3) |  | H6b: 2.8196 (*d*, 18.5) |
| 7 | 44.63 qC | - | 44.35 qC | - |
| 8 | 34.53 CH_2_ | H8a: 1.4260 (*m*) | 34.33 CH_2_ | H8a: 1.4345 (*m*) |
|  |  | H8b: 1.6931 (*m*) ^d^ |  | H8b: 1.7063 (*m*) |
| 9 | 18.46 CH_2_ | 1.6588 (*m*) ^d^ | 18.41 CH_2_ | H9a: 1.6470 (*m*) |
|  |  |  |  | H9b: 1.6816 (*m*) |
| 10 | 29.18 CH_2_ | Ha: 1.8528 (*m*) | 29.35 CH_2_ | Ha: 1.8184 (*m*) |
|  |  | Hb: 1.2796 (*ddd*, 12.0, 9.0, 3.0) |  | Hb: 1.3213 (ddd, 11.1, 8.5, 2.6) |
| 11 | 54.11 qC | - | 54.10 qC | - |
| 12 | 204.62 qC | - | 204.85 qC | - |
| 13 | 133.00 qC | - | 132.78 qC | - |
| 14 | 37.92 CH_2_ | H14b: 2.3149 (*d*, 13.5) | 38.06 CH_2_ | H14a: 2.3843 (*d*, 13.5) |
|  |  | H14a: 2.7266 (*d*, 13.5) ^d^ |  | H14b: 2.6052 (*d*, 13.6) |
| 15 | 70.82 qC | - | 70.75 qC | - |
| Me16- | 30.68 CH_3_ | 1.0411 (*s*) | 30.54 CH_3_ | 1.0464 (*s*) |
| Me17- | 29.20 CH_3_ | 0.9496 (*s*) | 29.55 CH_3_ | 0.9921 (*s*) |
| Me18- | 20.57 CH_3_ | 0.9720 (*s*) | 20.85 CH_3_ | 1.0392 (*s*) |
| Me19- | 22.28 CH_3_ | 0.8045 (*s*) | 22.04 CH_3_ | 0.6885 (*s*) |
| Me20- | 23.70 CH_3_ | 1.1718 (*s*) | 23.90 CH_3_ | 1.2063 (*s*) |
| 1’ | 144.77 qC | - | 144.85 qC | - |
| 2’ | 120.72 qC | - | 120.54 qC | - |
| 3’ | 111.20 CH | 6.4861 (*d*, 2.8) | 111.11 CH | 6.4793 (*d*, 3.1) |
| 4’ | 152.01 qC | - | 151.89 qC | - |
| 5’ | 114.90 CH | 6.5751 (*d*, 2.8) | 114.85 CH | 6.5722 (*d*, 3.1) |
| 6’ | 125.88 qC | - | 125.82 qC | - |
| MeO-4’ | 55.14 CH_3_ | 3.6426 (s) | 55.09 CH_3_ | 3.6439 (*s*) |
| Me-6’ | 16.37 CH_3_ | 2.0904 (s) | 16.29 CH_3_ | 2.0963 (s) |
| OH^C15^ |  | 4.1286 (s) |  | 4.1630 (s) |

^a^ Multiplicities inferred from APT- ^13^C [^1^H] experiment. Solvent as internal standard. ^b^ Recorded at 300 MHz. ^c^ Recorded at 1.2 GHz ^d^ Chemical shift assigned from a 1D psyche pure-shift experiment at 800 MHz. ^e^ Measured from a ^1^H 1D with homodecoupling.

| **Compound 1a (2.0 mg in 0.3 mL of CD_2_Cl_2_ in a 3.0 mm NMR tube)** |
| --- |

**Table S2** NMR spectroscopic data for compounds **1a** and **1b** in CD_2_Cl_2_.

| **Carbon** | **1a** | | **1b** | |
| --- | --- | --- | --- | --- |
|  | *δ*_C_, mult. *^a,b^* | *δ*_H_, mult., *J* (in Hz) *^c^* | *δ*_C_, mult. *^a,b^* | *δ*_H_, mult., *J* (in Hz) *^c^* |
| 1 | 23.06 CH_2_ | 2.7319 (dd, *J=* 16.6, 6.6 Hz)  2.8038 (dd, J= 16.6, 6.6 Hz) | 23.06 CH_2_ | 2.7742 (t, *J=* 6.7 Hz) |
|  |  |  |  | 2.8038 (dd, J= 16.6, 6.6 Hz) |
| 2 | 33.01 CH_2_ | 1.7624 (dd, *J* 13.3, 6.6 Hz) | 34.04 CH_2_ | 1.8051 (dd, *J*= 13.5, 6.7 Hz) |
|  |  | 1.8103 (dd, *J*= 13.3, 6.6 Hz) |  | 1.8588 (dd, *J*= 13.5, 6.7 Hz) |
| 3 | 77.03 qC | - | 76.8 qC | - |
| 4 | 45.70 CH_2_ | 2.5721 (d, *J=* 14.1 Hz) | 45.14 CH_2_ | 2.5141 (d, *J=* 13.8 Hz) |
|  |  | 2.7509 (d, *J=* 14.1 Hz) |  | 2.7050 (d, *J*= 13.9 Hz) |
| 5 | 154.3 qC |  | 155.10 qC |  |
| 6 | 44.22 CH_2_ | 2.7341 (d, *J*= 18.4 Hz) | 44.77 CH_2_ | 2.2232 (d, *J=* 18.7 Hz) |
|  |  | 2.8357 (d, *J*= 18.4 Hz) |  | 3.0276 (d, *J*= 18.7 Hz) |
| 7 | 45.3 qC | - | 45.25 qC | - |
| 8 | 35.45 CH_2_ | 1.5334 (m) | 35.40 CH_2_ | 1.52 (dd, *J*= 15.8, 3.1 Hz) |
|  |  | 1.7298 (m) |  | 1.7535 (m) |
| 9 | 19.33 CH_2_ | 1.7319 (m) | 19.31 CH_2_ | 1.7443 (m) |
| 10 | 29.90 CH_2_ | 1.4157 (d, *J*= 14.6 Hz) | 30.02 CH_2_ | 1.4414 (m) |
|  |  | 1.9462 (d, *J*= 14.6 Hz) |  | 1.9435 (m) |
| 11 | 55.35 qC | - | 55.42 qC | - |
| 12 | 208.85 qC | - | 209.32 qC | - |
| 13 | 133.90 qC | - | 133.45 qC | - |
| 14 | 39.97 CH_2_ | 2.4196 (d, *J*= 14.4 Hz) | 40.37 CH_2_ | 2.5060 (d, *J*= 14.5 Hz) |
|  |  | 2.7032 (d, *J=* 14.4 Hz) |  | 2.5698 (d, *J*= 14.5 Hz) |
| 15 | 71.42 qC |  | 71.17 qC |  |
| 16 | 30.85 CH_3_ | 1.1513 (s) | 31.88 CH_3_ | 1.2348 (s) |
| 17 | 30.01 CH_3_ | 1.1002 (s) | 29.1 CH_3_ | 1.0406 (s) |
| 18 | 21.47 CH_3_ | 1.0127 (s) | 21.51 CH_3_ | 1.1054 (s) |
| 19 | 22.85 CH_3_ | 0.8944 (s) | 22.70 CH_3_ | 0.8038 (s) |
| 20 | 24.79 CH_3_ | 1.2702 (s) | 24.27 CH_3_ | 1.2252 (s) |
| 1’ | 145.84 qC | - | 145.76 qC | - |
| 2’ | 121.19 qC | - | 121.16 qC | - |
| 3’ | 111.53 CH | 6.4441 (d, *J=* 2.8 Hz) | 111.60 CH | 6.4502 (d, *J*= 2.8 Hz) |
| 4’ | 153.14 qC | - | 153.13 qC | - |
| 5’ | 115.54 CH | 6.5594 (d, *J*= 2.8 Hz) | 115.64 qC | 6.5633 (d, *J*= 2.8 Hz) |
| 6’ | 127.60 qC | - | 127.42 qC | - |
| MeO-4’ | 56.0 CH_3_ | 3.7008 (s) | 56.0 CH_3_ | 3.701 (s) |
| Me-6’ | 16.91 CH_3_ | 2.1463 (s) | 17.08 CH_3_ | 2.165 (s) |

*^a^* Multiplicities inferred either from DEPT-135 or HSQC experiments. Solvent as internal standard. *^b^* Measured at 200 MHz. *^c^* Measured at 800 MHz. ^d^ Chemical shift assigned from a 1D psyche pure-shift experiment


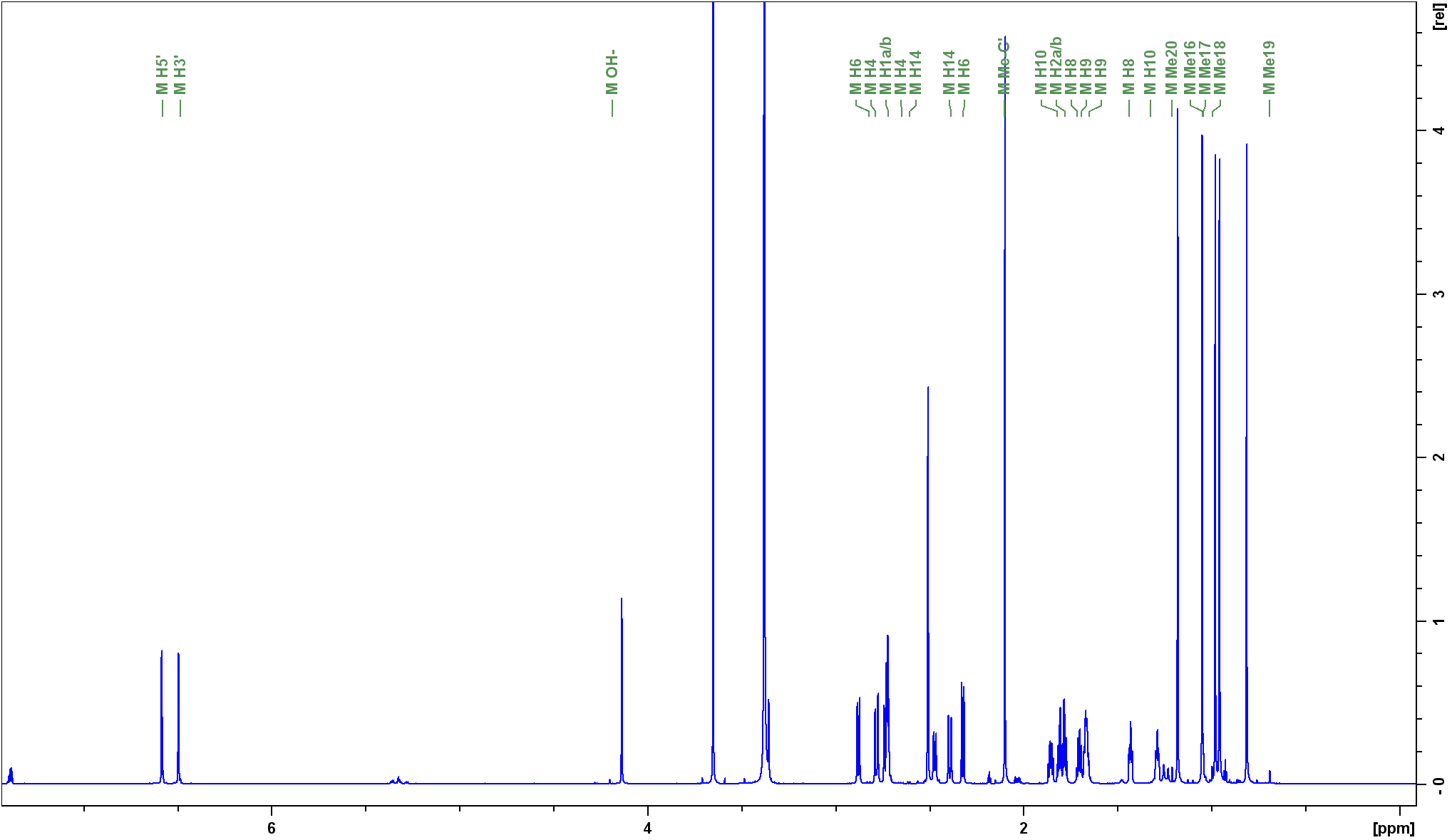


**Figure S12.** ^1^H 1D NMR spectrum of **1a** (zg, DMSO-d_6_, SAM: 1.5 mg, N S: 40, 1.2 GHz).


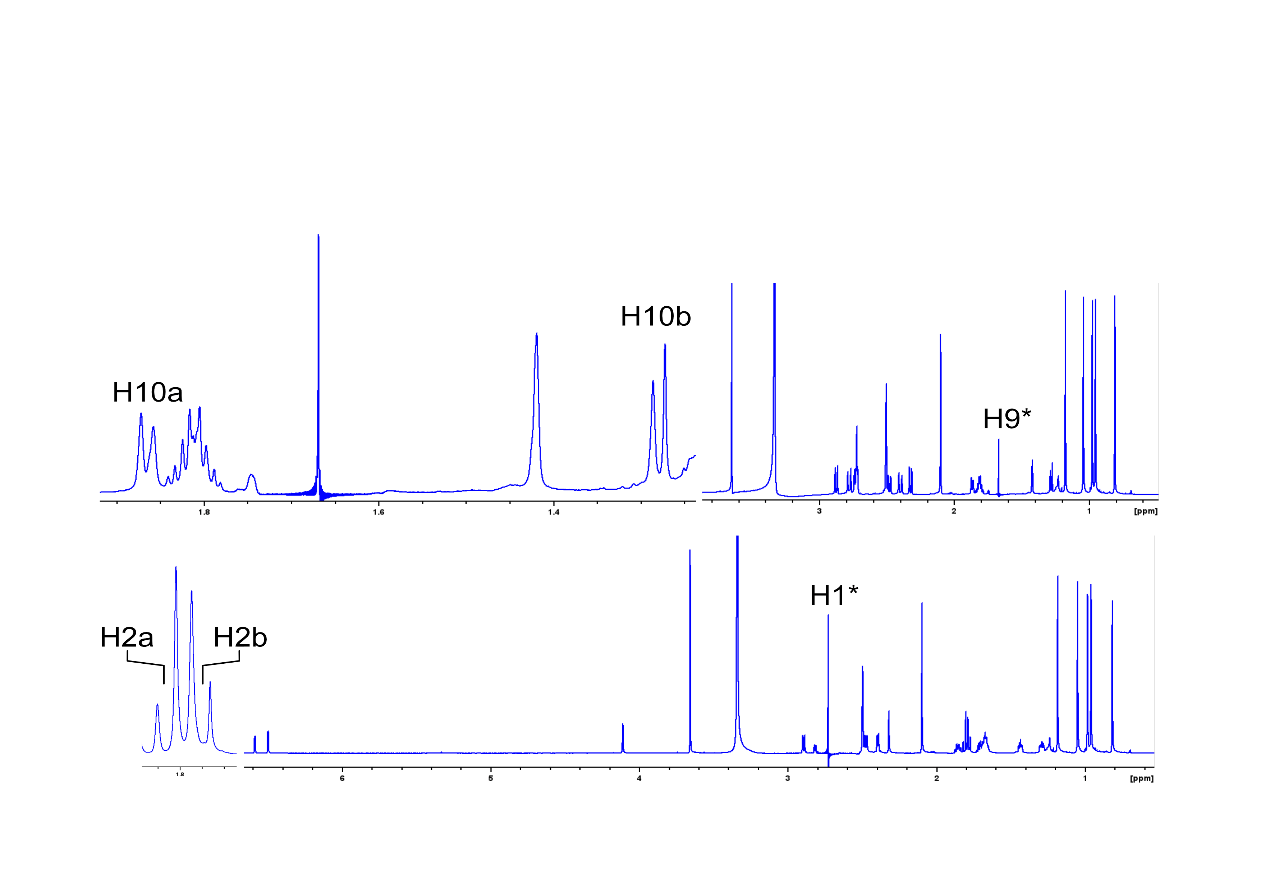


b)

a)

**Figure S13.** Homodecoupled 1D ^1^H NMR spectrum of **1a** (zghd.2, DMSO-d_6_, SAM: 3 mg, NS: 16, 800 MHz), in each case, irradiated protons are indicated with *. 1D ^1^H selective homonuclear decoupling spectrum of **1a** upon irradiation of H9 (1.663 ppm). The resonances of H10a (1.8521 ppm) and H10b (1.2805 ppm) collapse into doublets (^2^J_HH_=11.0 Hz) **(a)**. 1D ^1^H selective homonuclear decoupling spectrum of **1a** upon irradiation of H1 (2.7180 ppm). The resonances of H2a (1.7752 ppm) and H2b (1.8076 ppm) collapse into doublets (^2^J_HH_=17.9 Hz) **(b)**.


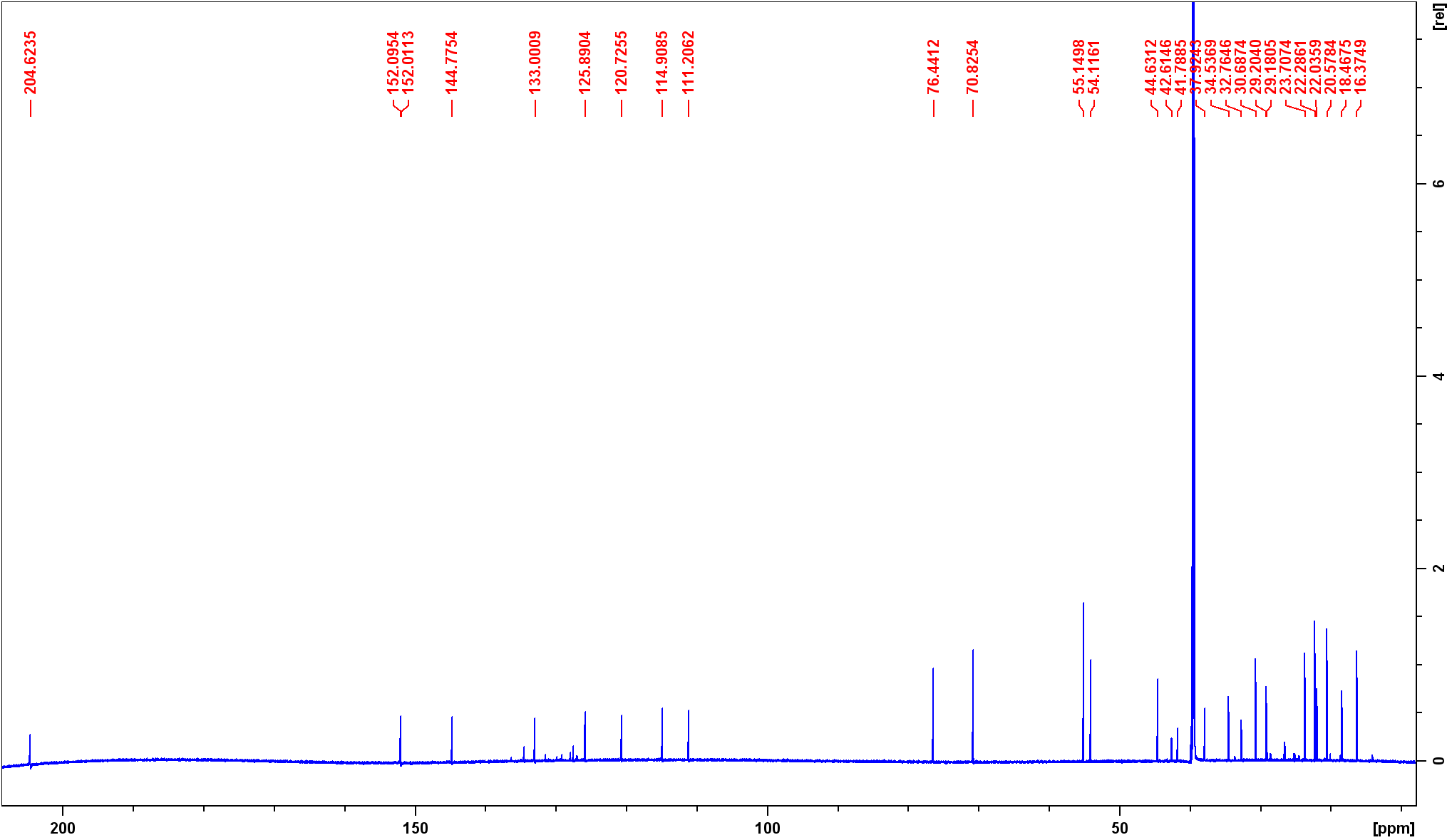


(a)

**
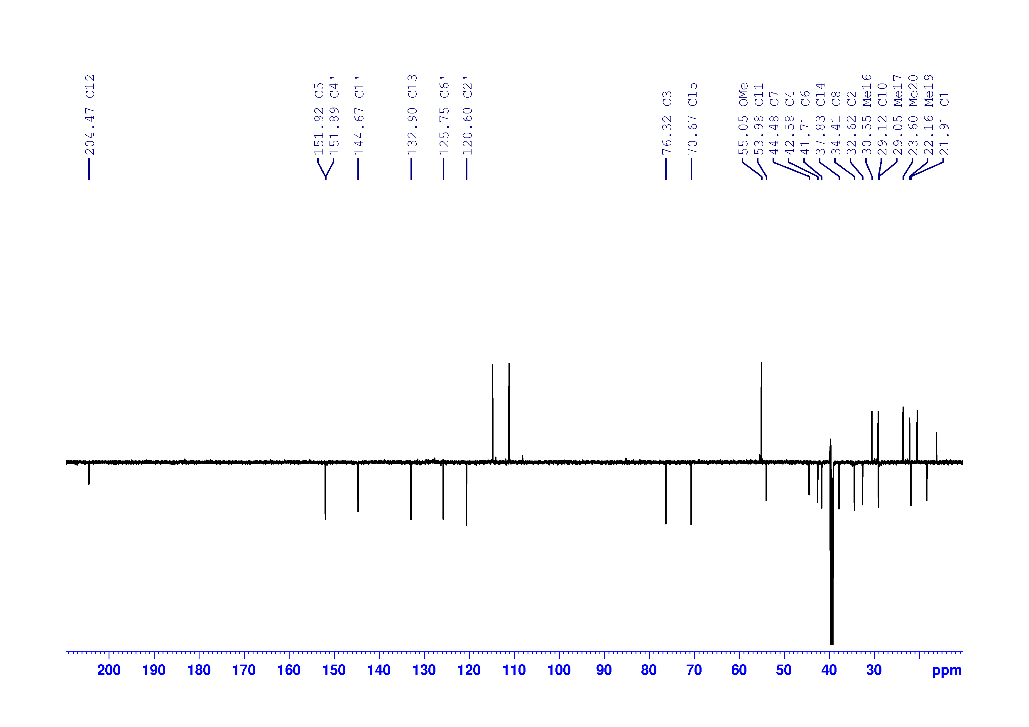
**

(b)

**Figure S14**. 1D ^13^C-{^1^H} NMR spectrum of **1a** (zgpg30, DMSO-d_6_, SAM: 1.5 mg, NS: 3K, 1.2 GHz) **(a)**. APT carbon spectrum of **1a** (jmod, DMSO-d_6_, SAM: 3 mg, NS: 8K, ^1^J_CH_ = 145 Hz, 800 MHz) **(b)**.


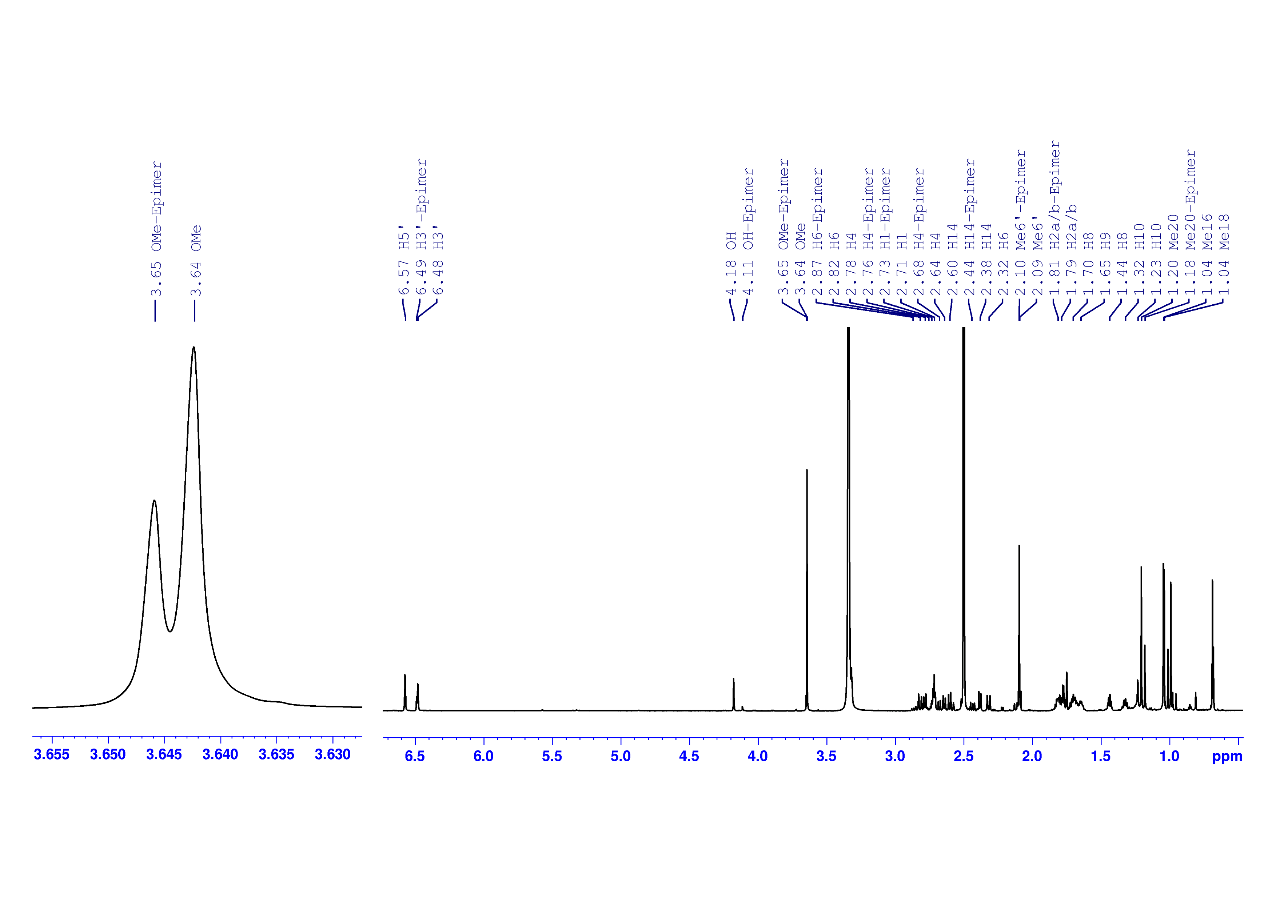


(b)

(a)

**Figure S15.** 1D ^1^H NMR spectrum of epimeric mixture of **1a** and **1b (a).** Epimerization was induced by dissolving a pure sample of **1a** in 1,1,2,2-tetrachloroethane at room temperature for 7 days. As expected, all the resonances in the spectrum are duplicated. Duplicated resonance of MeO-C4’ (**b**) (zg30, DMSO-d_6_, NS: 32, 900 MHz).


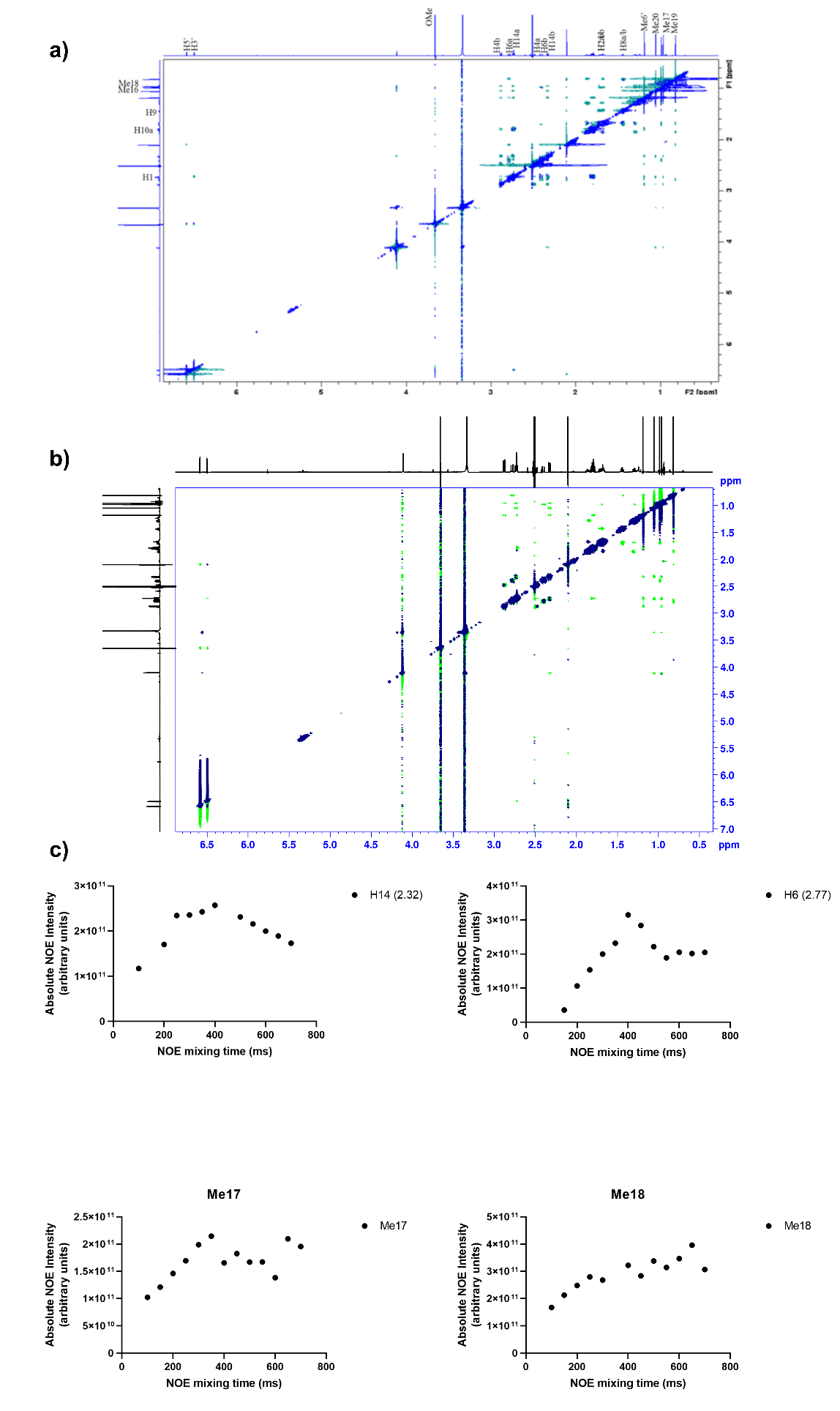


**Figure S16****.** a) 2D NOESY spectrum of **1a** (noesyetgp, DMSO-d_6_, mixing time: 400 ms, NS: 48, NUS: 72%,184,512. 800 MHz). b) 2D NOESY spectrum of **1a** (noesyetgp, DMSO-d_6_, mixing time: 200 ms, NS: 48, NUS: 72%,184,512. 800 MHz). c) 1D NOE build-up curves of **1a** for protons H-14, H-6, Me17 and Me18.

**Table S3.** Proton distance information derived from a NOESY spectrum at 200 ms mixing time. All distances were referenced to H4a/H4b.

| **Proton 1** | **Proton 2** | **Distance [Å]-1a** | **Distance [Å]-1b** |
| --- | --- | --- | --- |
| H4b | H6b | 2.65 | 2.11 |
| H4b | H14b | 2.33 | 2.41 |
| H4b | H2 | 2.91 | 3.26 |
| H4b | Me20 | 3.45 | 3.65 |
| H4b | Me16 | 2.63 | 2.37 |
| H4b | Me17 | 1.85 | 2.37 |
| H4b | Me19 | 4.56 | 4.52 |
| H14b | H6a | 2.24 | 2.76 |
| H14b | Me20 | 2.98 | 2.68 |
| H14b | Me16 | 2.47 | 2.45 |
| H14b | Me18 | 4.07 | 4.40 |
| H14b | Me17 | 2.87 | 2.82 |
| H6b | H14a | 1.76 | 2.34 |
| H6b | Me6’ | 4.23 | 3.81 |
| H6b | H2 | 4.46 | 4.38 |
| H6b | Me20 | 3.41 | 3.20 |
| H6b | Me16 | 4.37 | 4.21 |
| H6b | Me18 | 2.58 | 2.84 |
| H6b | Me17 | 3.95 | 3.89 |


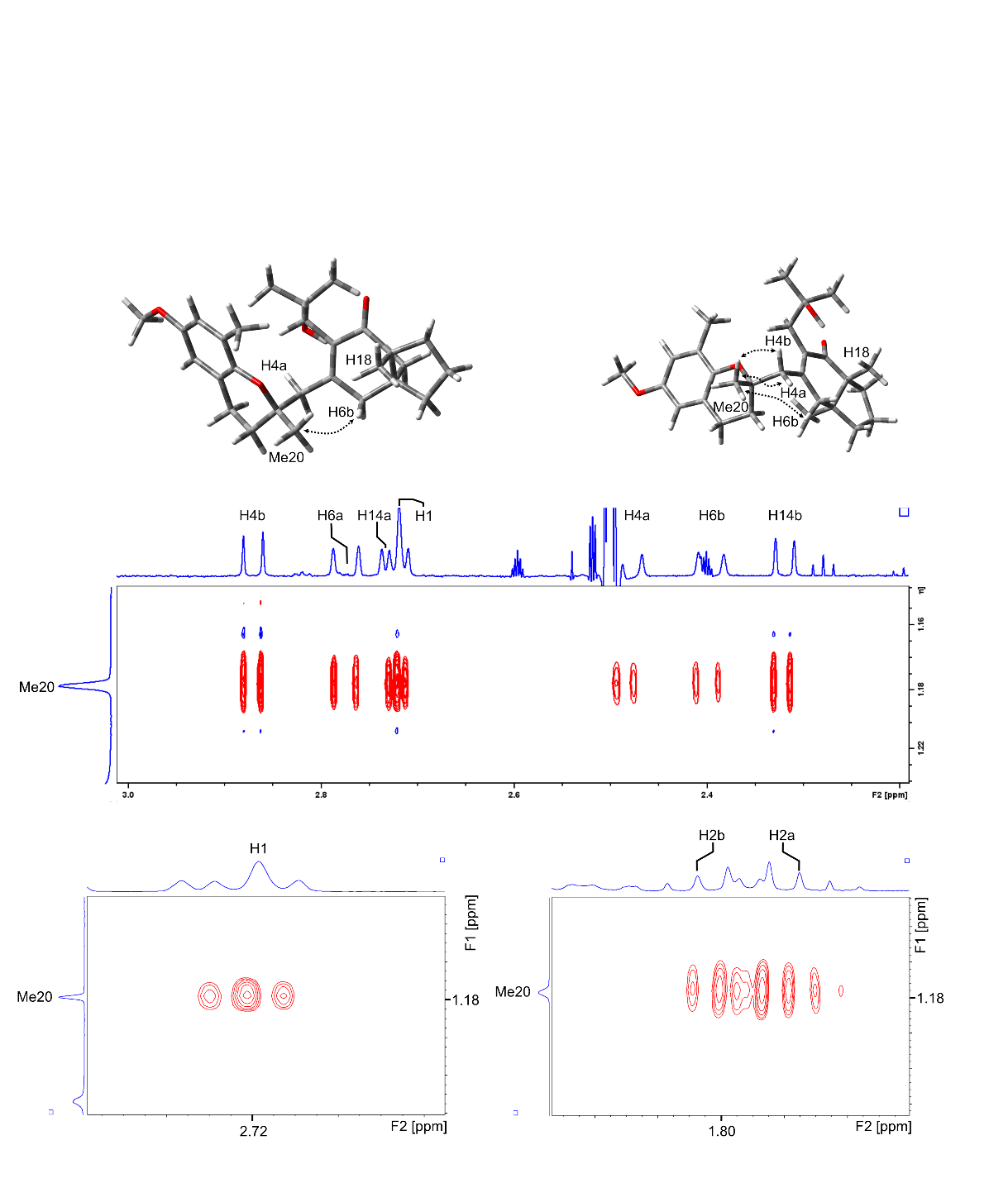


**Figure S17**. Selected NOESY contacts related to conformational equilibrium, of compound **1a**, and two possible conformers that satisfy the weak NOE contacts from H20 to H6b (noesyph, DMSO-d_6_, mixing time: 470 ms, NS: 32, 800 MHz).

**Table S4** Relevant computed coupling constants of the chromane fragment in compound **1a** for both M- and P-helicities

| **Chromane helicities** | **^2^*J*_C3H2a_ (Hz)** | **^2^*J*_C3H2b_ (Hz)** | **^2^*J*_C1H2a_ (Hz)** | **^2^*J*_C1H2b_ (Hz)** | **^3^*J*_C2’H2a_ (Hz)** | **^3^*J*_C2’H2b_ (Hz)** |
| --- | --- | --- | --- | --- | --- | --- |
| *M-* | -5.4 | -1.2 | -3.5 | -3.5 | 1.1 | 7.6 |
| *P-* | -1.1 | -5.9 | -3.8 | -3.7 | 7.9 | 1.0 |

*J*_CH_ were computed with the mixed method at MPW1PW91/6-311+G(2d,p) (iefpcm=dmso). Values are shown as obtained from DFT calculations.


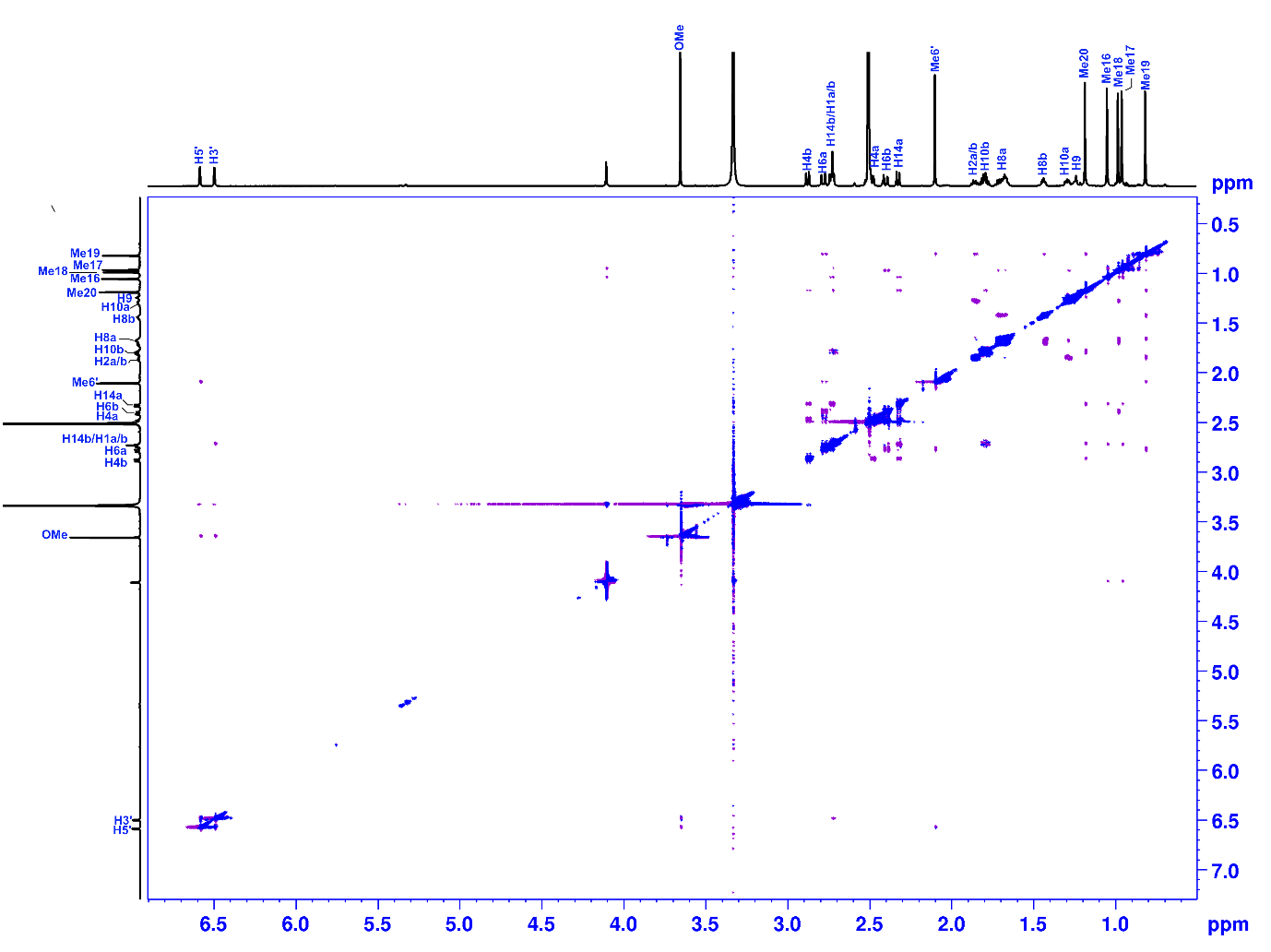


**Figure S18.** 2D ROESY spectrum of compound **1a** (roesyetgp, DMSO-d_6_, NS: 48. 800 MHz)


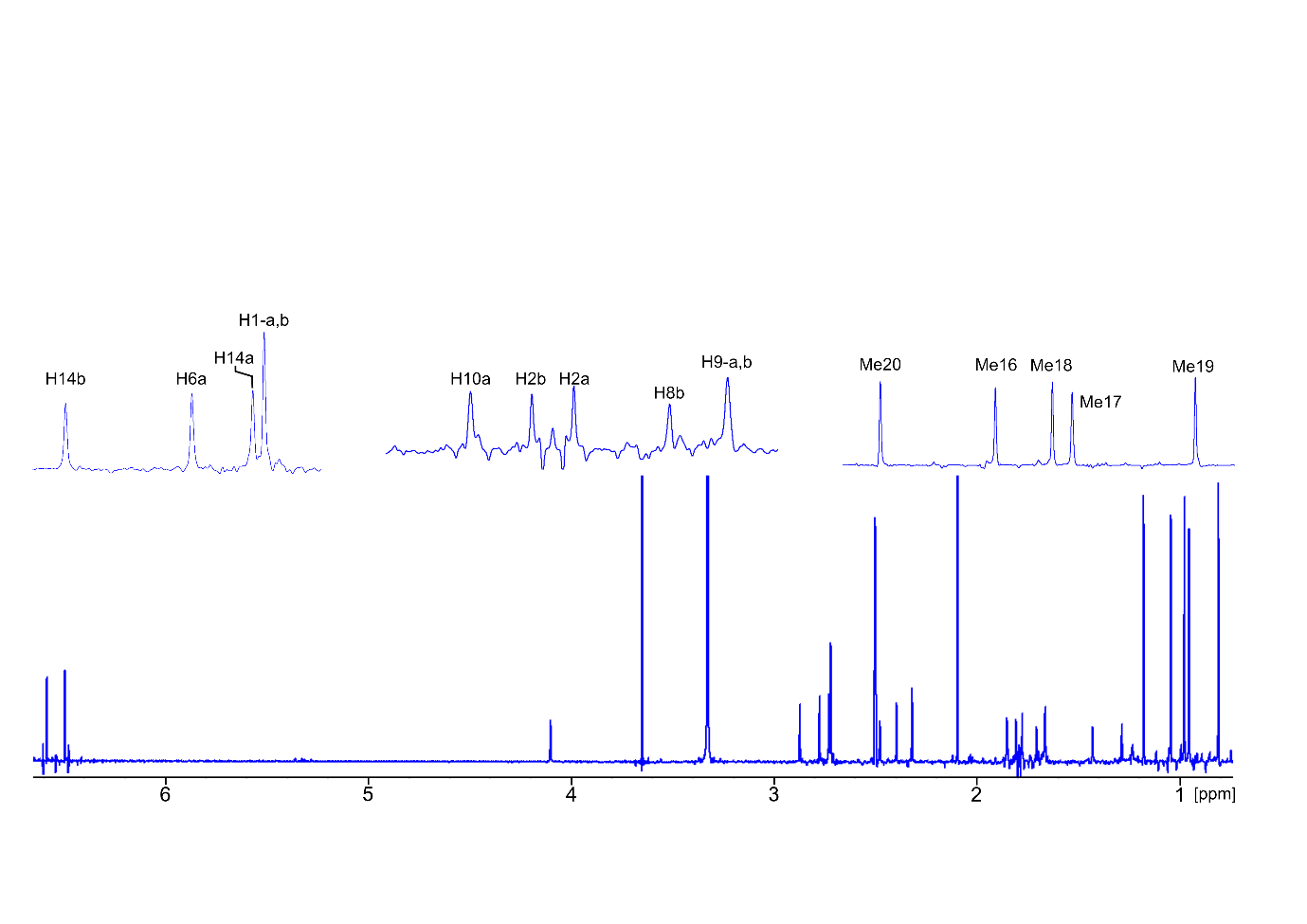


(a)

(d)

(c)

(b)

**Figure S19.** 1D ^1^H PSYCHE pure-shift spectrum of **1a** (DMSO-d_6_, 800 MHz, NS 16, TD1: 32) (**a**). The inset shows resonances from 0.7 ppm to 1.2 ppm **(b)**. Resonances from 1.6 ppm to 1.9 ppm **(c)**. Collapsing of protons H2a (1.775 ppm), H2b (1.808 ppm), and H9 (1.659 ppm). Resonances from 2.7 ppm to 2.9 ppm **(d)**. The pure-shift experiment allows the straightforward assignment of the isochronic protons of H1.


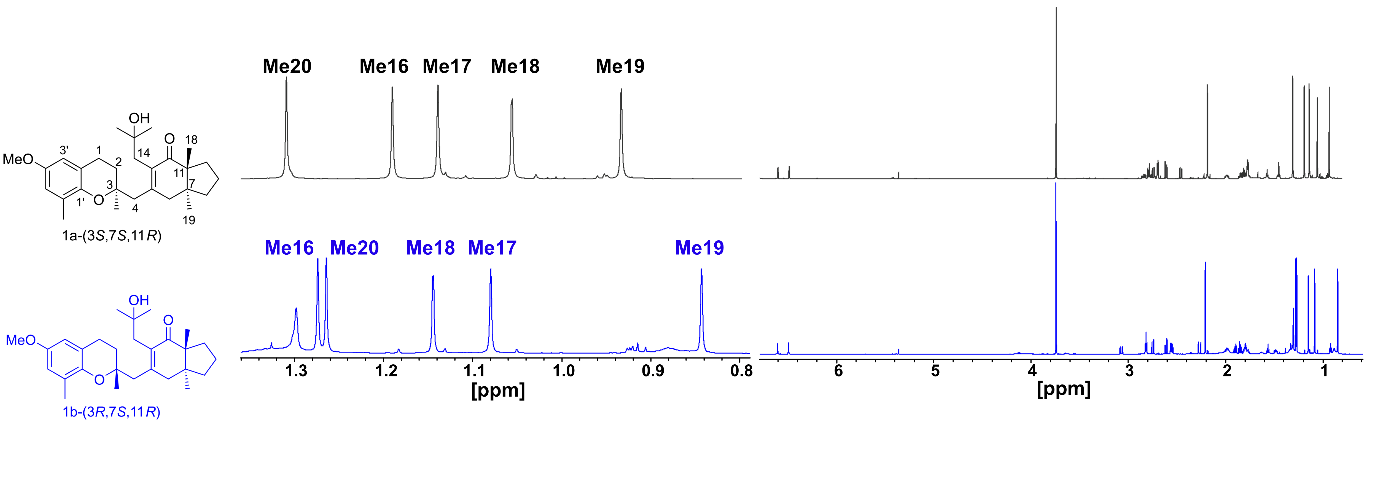


**Figure S20.** Comparison of the ^1^H 1D spectrum of **1a** (black) and **1b** (blue) **(a)**. The inset on the left shows the assignment of the methyl groups in both compounds (b). (zg, 800 MHz, DCM)

**Table S5** J-couplings considered in the CASE-based analysis of **1a**.

| **Proton to** | **Carbon** | **Proton to** | **Carbon** | **Proton to** | **Proton** |
| --- | --- | --- | --- | --- | --- |
| H2a,H2b | C2' | H4a,H4b | C2 | H2a,H2b | H1a |
| H2a,H2b | C20 | H4a,H4b | C6 | H2a,H2b | H1b |
| H2a,H2b | C3 | H4a,H4b | C13 |  |  |
| H4a,H4b | C3 | H4a,H4b | C5 |  |  |
| H4a,H4b | C20 |  |  |  |  |

*
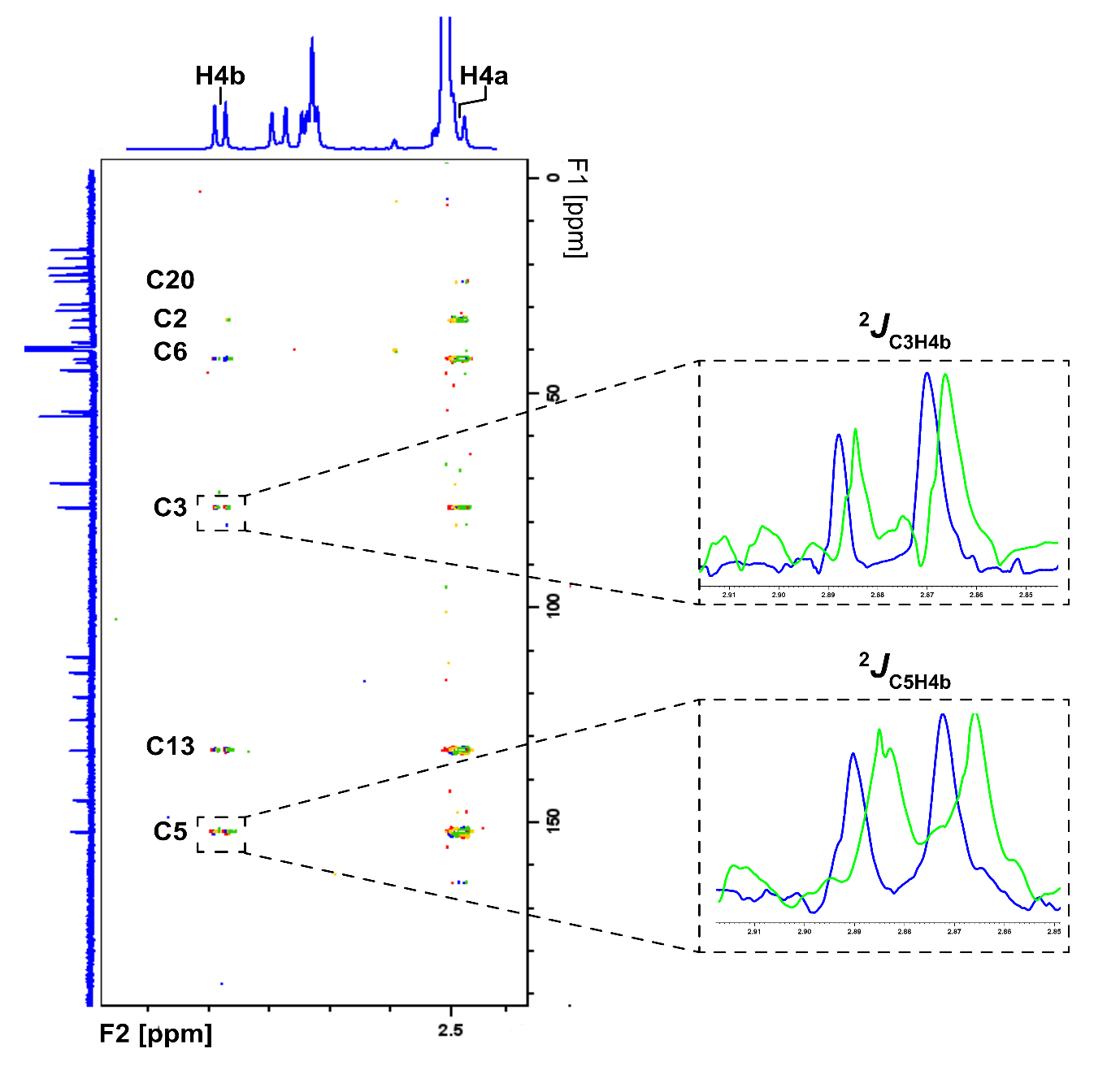
*

**Figure S21.** 2D IPAP-HSQMBC-COSY spectrum (optimized to 5 Hz) after selective inversion of the H4b (2.870 ppm) proton of **1a (a)**. In the IPAP-HSQMBC-COSY, the α (red-blue) and β (yellow-green) multiplets for the cross-peaks involving C20, C2, C6, C3, C13, and C5 are shown. (hsqccoetgpiajclrndsp, DMSO-d_6_, NS: 72, NUS: 14%/35/512)


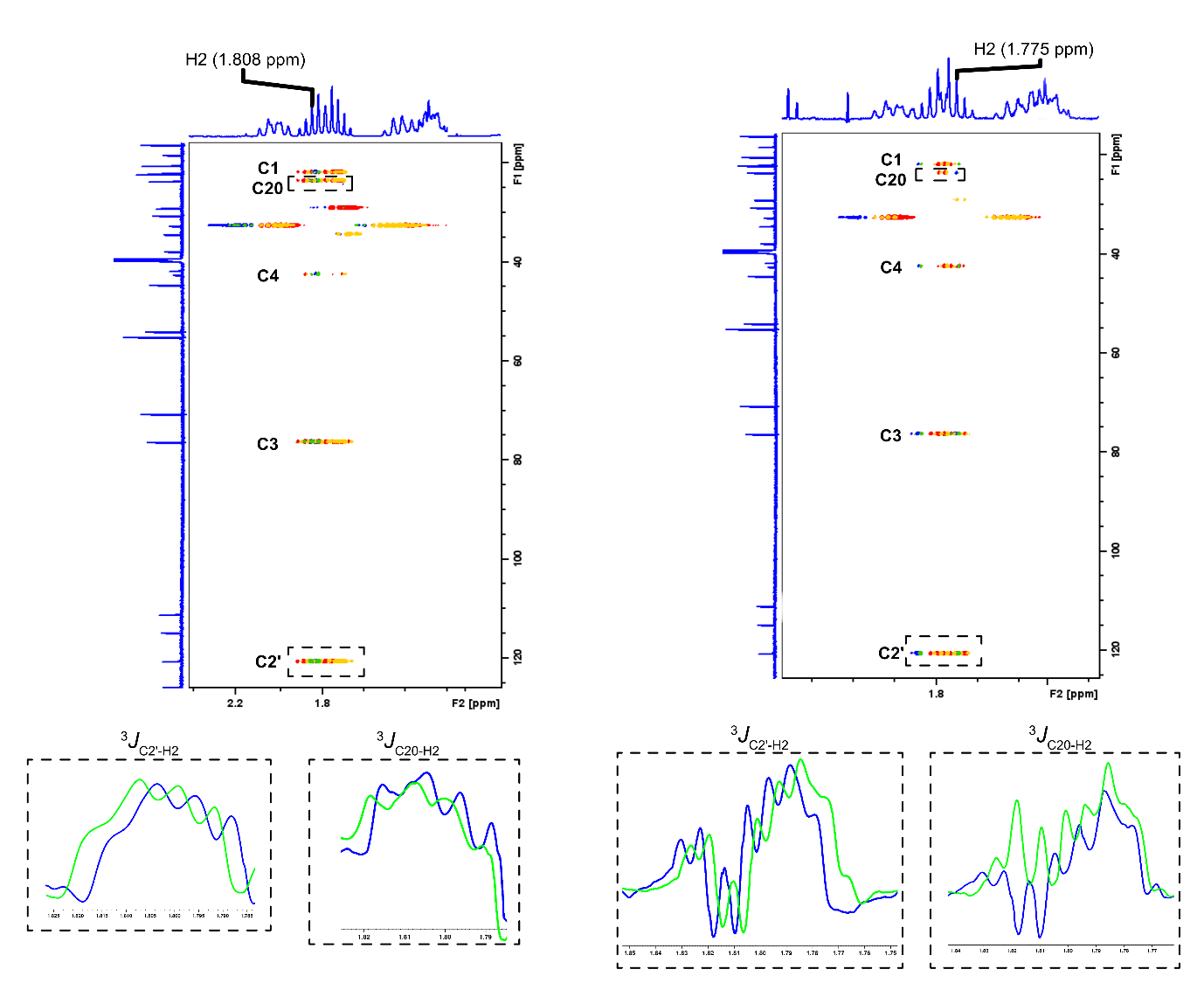


**Figure S22.** 2D IPAP-HSQMBC spectrum (optimized to 7 Hz) after selective inversion of the H2a/H2b pair (1.808 and 1.775 ppm) of **1a**. In the figure, α (red-blue) and β (yellow-green) multiplets for the cross-peaks involving C1, C20, C4, C3, and C2’ are shown *(hsqcetgpiajclrndsp,* 900 MHz, DMSO-*d_6_*, NS: 64, NUS: 25%, 64, 512).

**Table S6.** ^13^C-^1^H coupling constants for epimers **1a** and **1b**.

| **^2,3^*J*_CH_ coupling constant** | **Spin-Spin Coupling value (Hz)-1a*** | **Spin-Spin Coupling value (Hz)-1b** |
| --- | --- | --- |
| C2’-H2a | 3.8 | 4.1 |
| C2’-H2b | 5.1 | 4.4 |
| C20-H2a | 2.8 | 3.2 |
| C20-H2b | 2.2 | 1.8 |
| C3-H2a | 3.5 | 3.4 |
| C3-H2b | 3.5 | 3.4 |
| C3-H4a | 6.6 | 5.9 |
| C3-H4b | 2.7 | 3.5 |
| C20-H4a | 5.1 | 4.5 |
| C20-H4b | 1.3 | 2.4 |
| C2-H4a | 1.7 | 2.6 |
| C2-H4b | 2.9 | 2.9 |
| C6-H4a | 4.1 | 4.2 |
| C6-H4b | 5.7 | 5.3 |
| C13-H4a | 5.1 | 5.6 |
| C13-H4b | 4.0 | 4.2 |
| C5-H4a | 4.2 | 5.3 |
| C5-H4b | 6.5 | 5.4 |
| C15-H14a | 5.0 | 4.1 |
| C15-H14b | 5.7 | 5.9 |
| C16-H14a | 1.8 | 1.3 |
| C16-H14b | 2.0 | 1.8 |
| C17-H14a | 1.3 | 2.4 |
| C17-H14b | 3.4 | 4.5 |
| C5-H14a | 5.6 | 5.2 |
| C5-H14b | 4.2 | 3.9 |
| C12-H14a | 3.6 | 3.7 |
| C12-H14b | 5.6 | 5.3 |

*Spin-spin coupling values are presented as absolute values because they were measured from an IPAP-HSQMBC experiment.


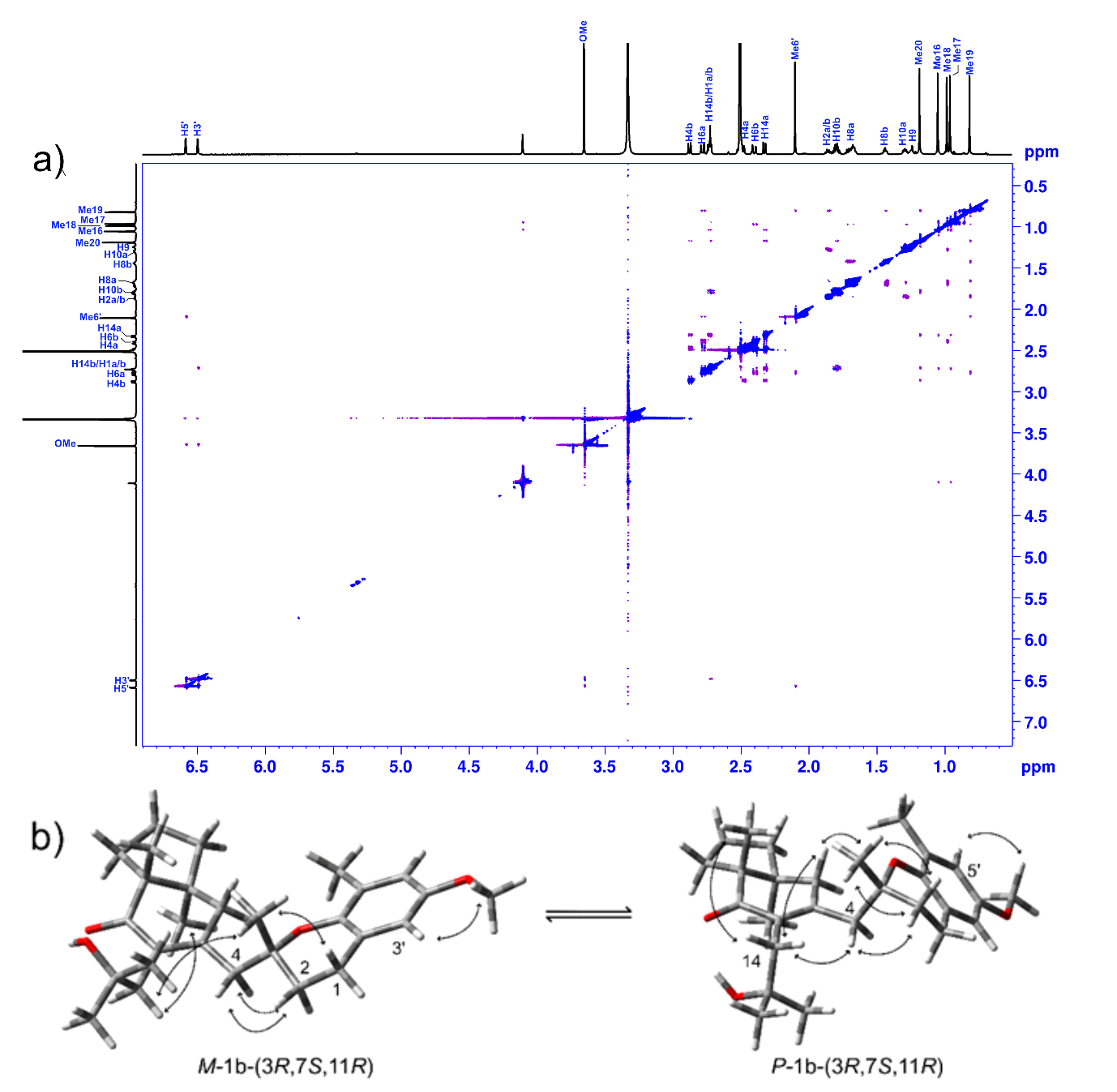


**Figure S23.** 2D ROESY spectrum of **1b** in DMSO recorded at 1.2 GHz (**a**), and main ROE contacts observed (**b**).


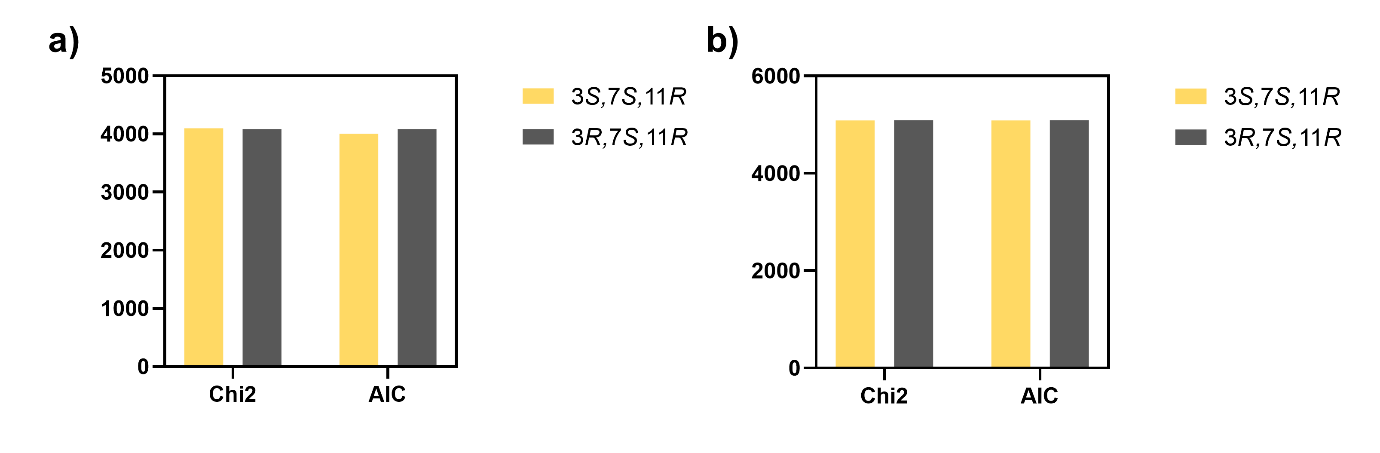


**Figure S24.** Computer-Assisted Structure Elucidation (CASE) study for the epimeric discrimination of **1a** **(a)** and **1b** **(b)** using NMR isotropic observables based on NMR isotropic data.

| **Exploratory data analysis of DP4+ data (reciprocal epimer distinction)** |
| --- |


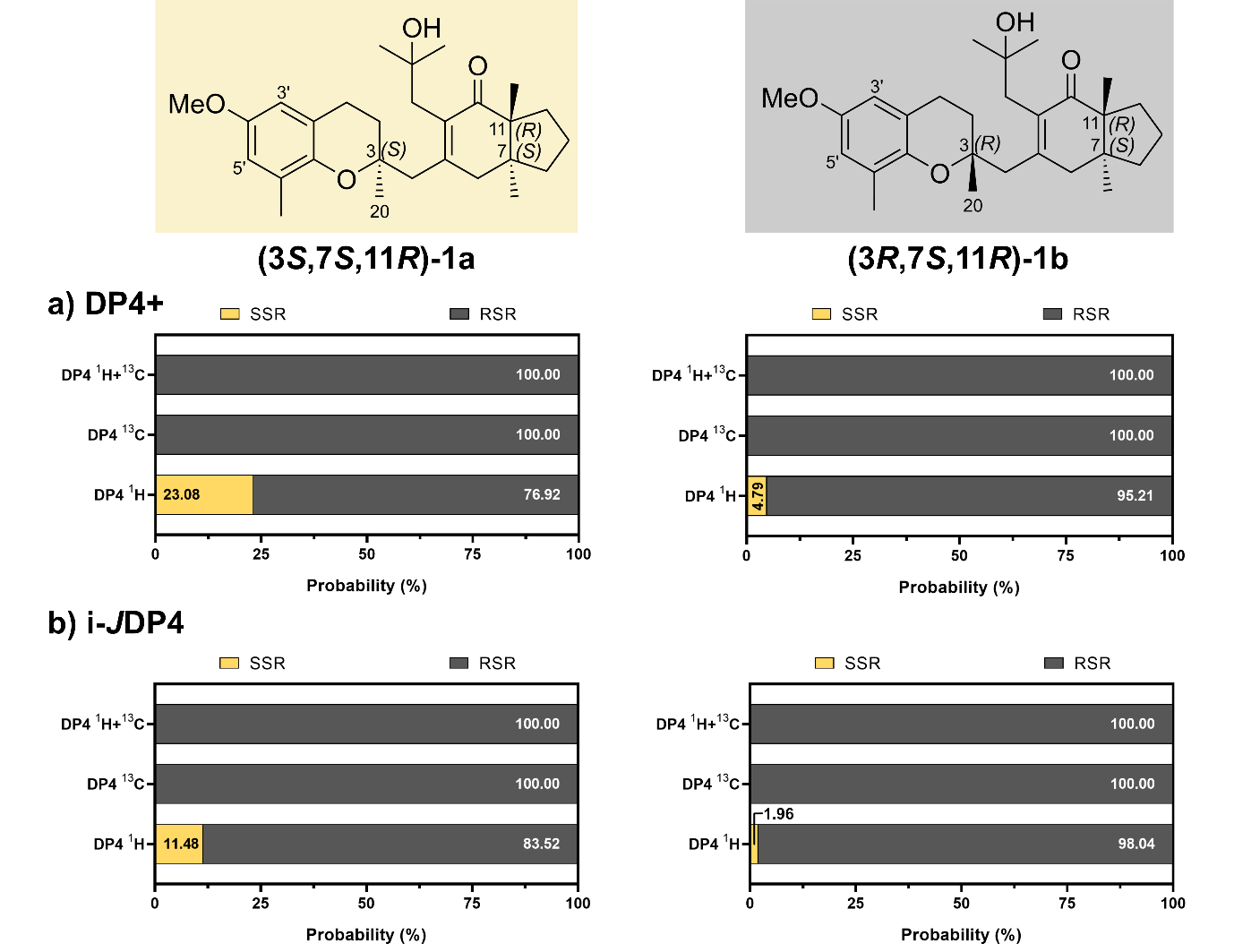


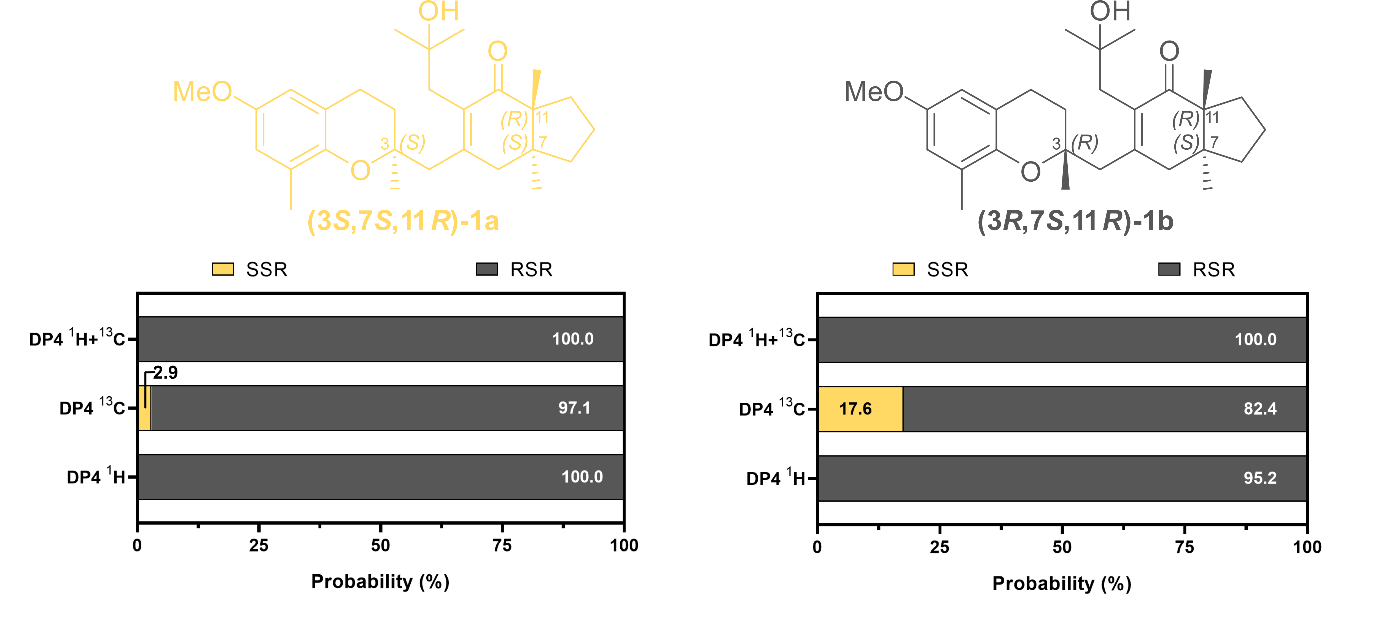


**Figure S25**. DP4+ Bayesian probability analysis of (3S,7S,11R)-**1a** and (3R,7S,11R)-**1b** in CD_2_Cl_2_.

**Our findings**:

| 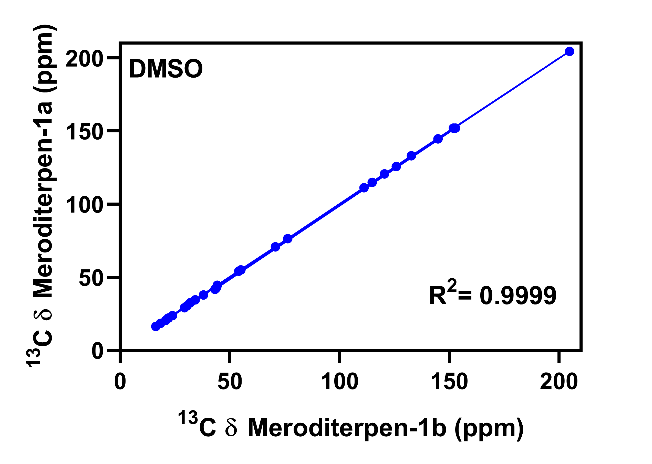 | 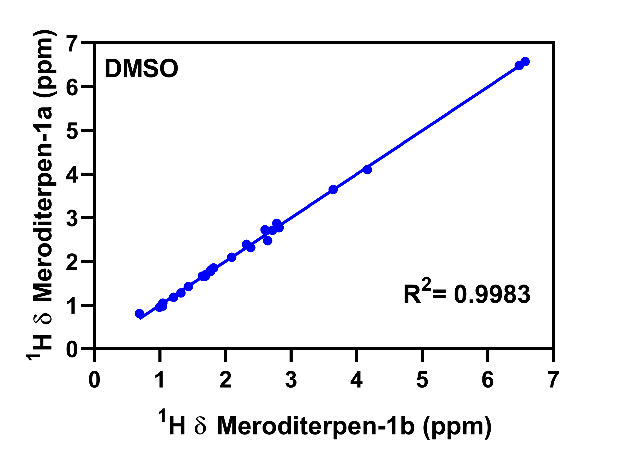 |
| --- | --- |
| 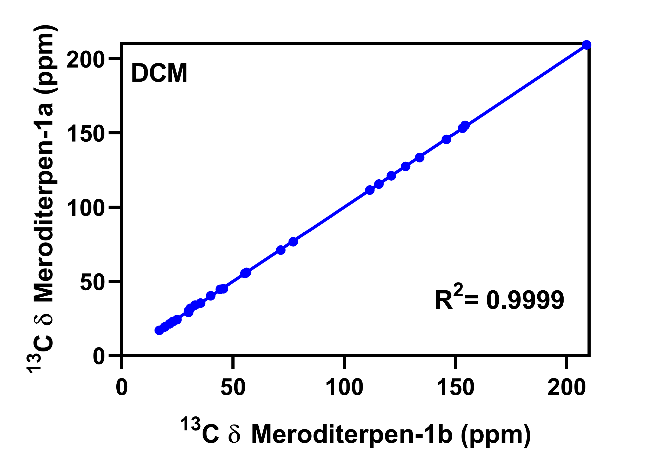 | 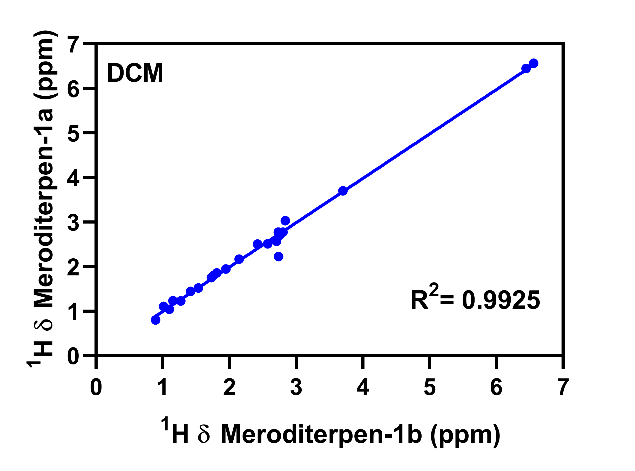 |

**Figure S26.** Carbon (**left**) and proton (**right**) chemical shift correlation plot between Meroditerpene-**1a** and **-1b** in DMSO and CDM. The coefficient of correlation (R²) is shown in both cases.

**Figure S27.** Chemical structure of the molecules used in the epimeric reciprocal assignment analysis. To expand the number of epimers in the epimeric reciprocal assignment, we introduce the concept of apparent epimers by NMR, referring to a situation within a set of diastereoisomers where two compounds are not epimers of each other, yet one becomes an epimer of the other when its mirror image is considered. For example, among the diastereoisomeric set 75a–75d, the pairs 75a–75d, 75a–75b, and 75c–75d are true epimers. However, 75a and 75c are not epimers themselves. Interestingly, the mirror image of 75c (ent-75c) forms an epimeric relationship with 75a, thereby creating a new, indirect pair of epimers. This concept is particularly useful when interpreting subtle NMR differences in stereoisomeric mixtures.


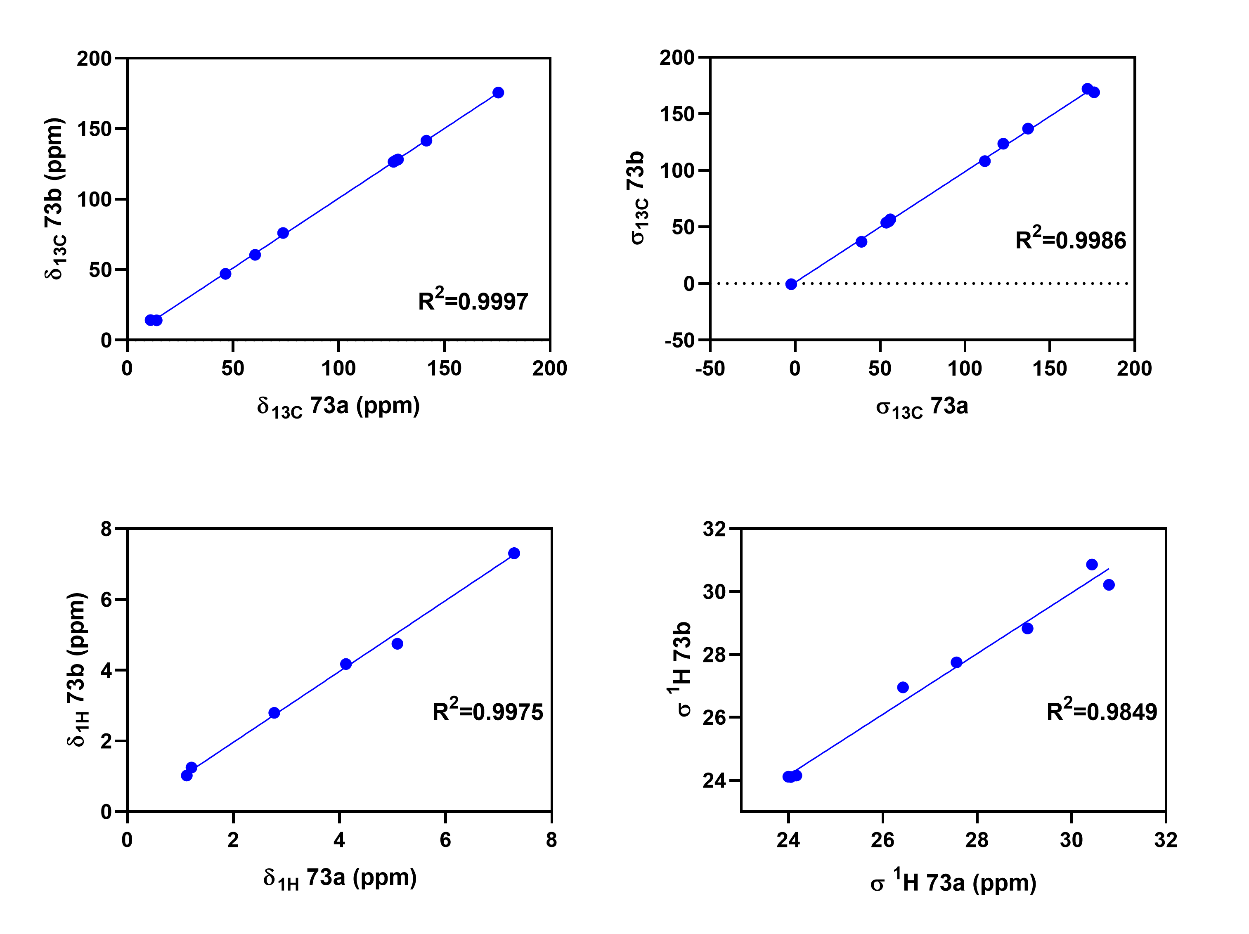


**Figure S28.** Chemical shift correlation plot between experimental (δ) and DFT calculated (σ) epimers **73a** and **73b**. The coefficient of correlation (R²) is shown in both cases.


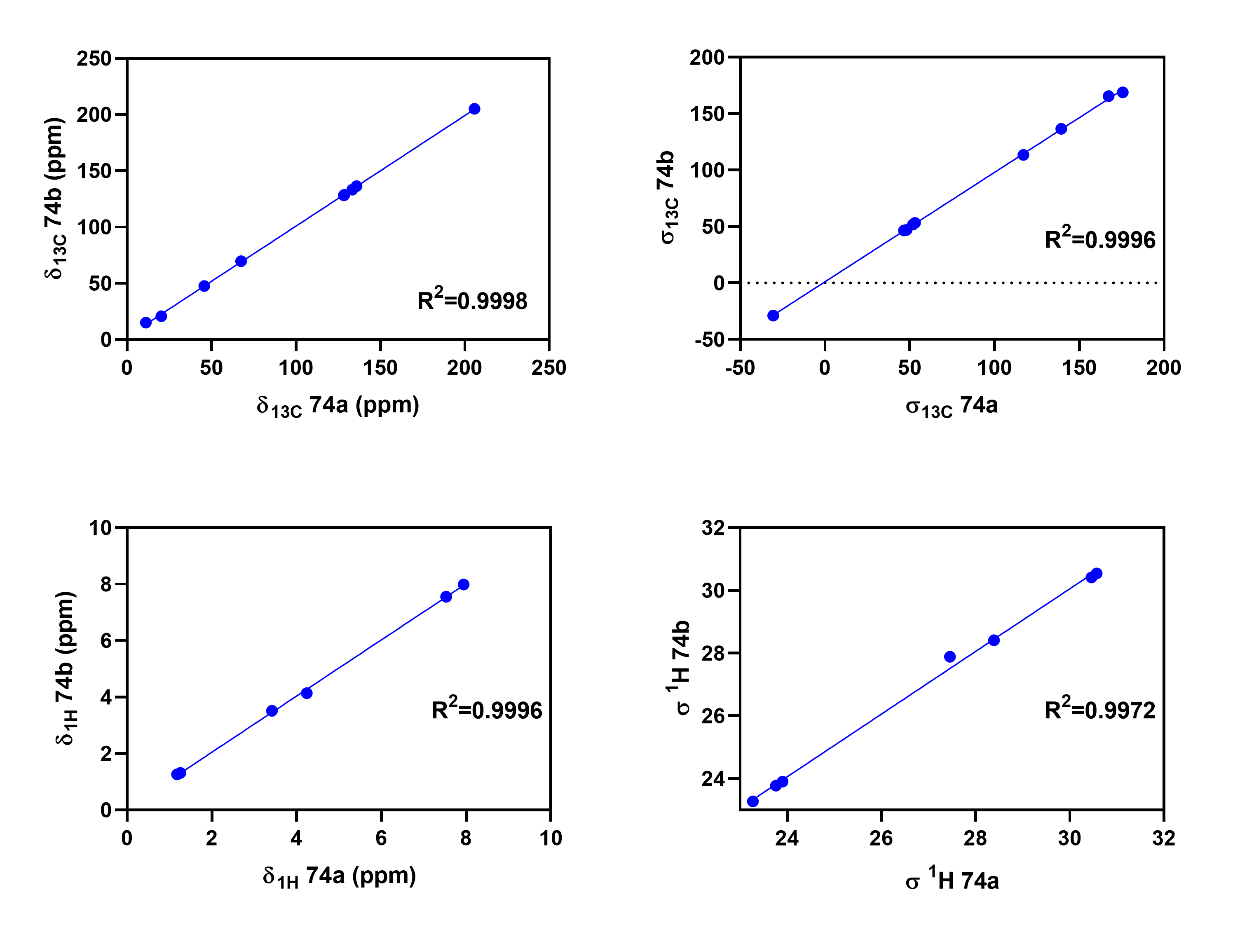


**Figure S29.** Chemical shift correlation plot between experimental (δ) and DFT calculated (σ) epimers **74a** and **74b**. The coefficient of correlation (R²) is shown in both cases.


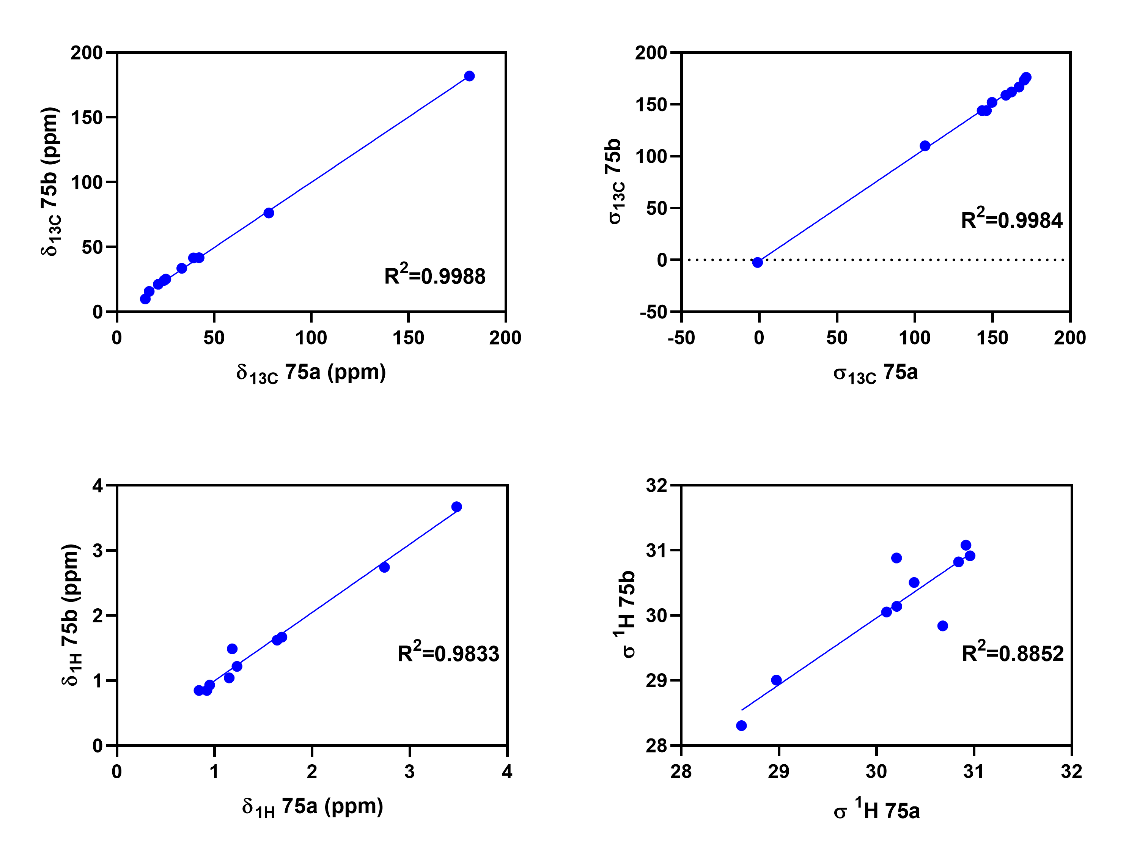


**Figure S30.** Chemical shift correlation plot between experimental (δ) and DFT calculated (σ) epimers **75a** and **75b**. The coefficient of correlation (R²) is shown in both cases.


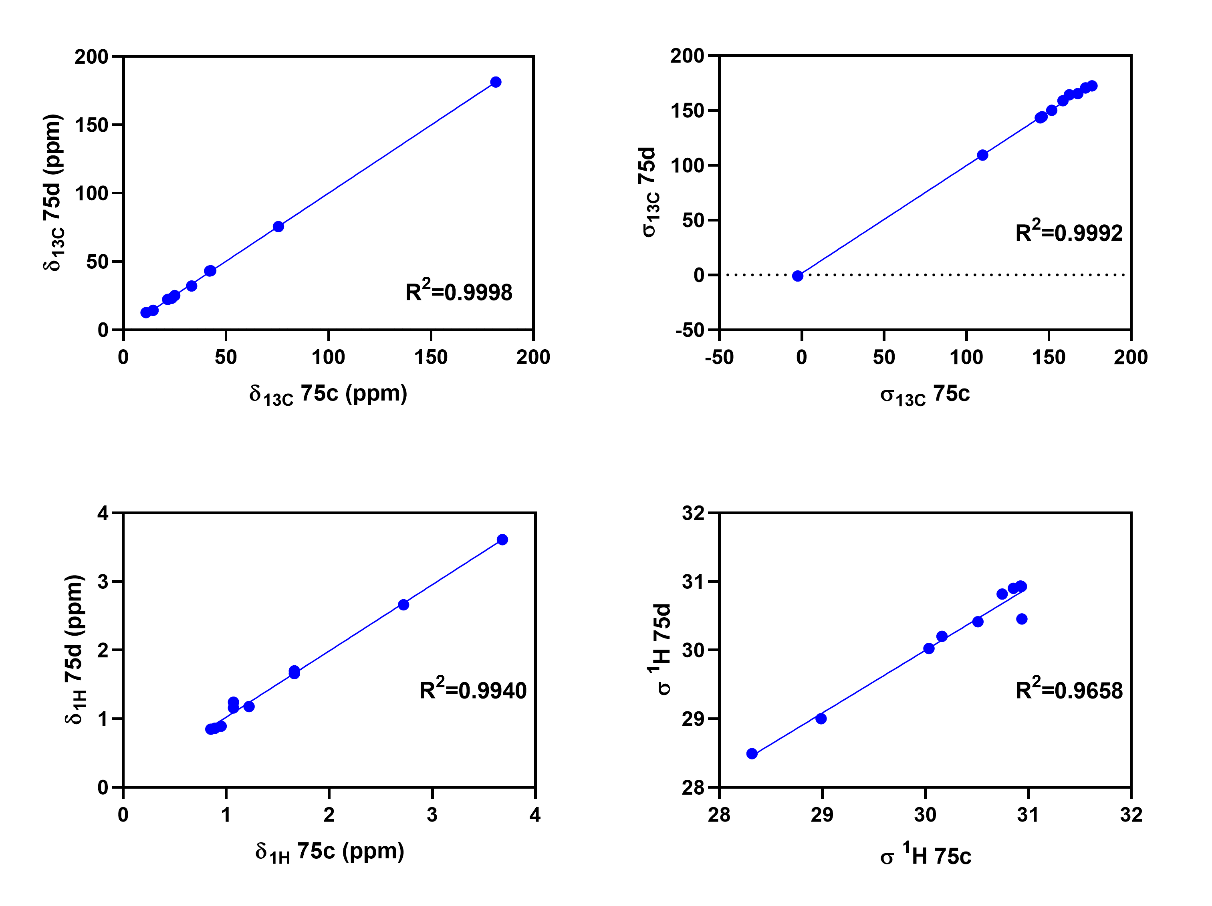


**Figure S31.** Chemical shift correlation plot between experimental (δ) and DFT calculated (σ) epimers **75c** and **75d**. The coefficient of correlation (R²) is shown in both cases.


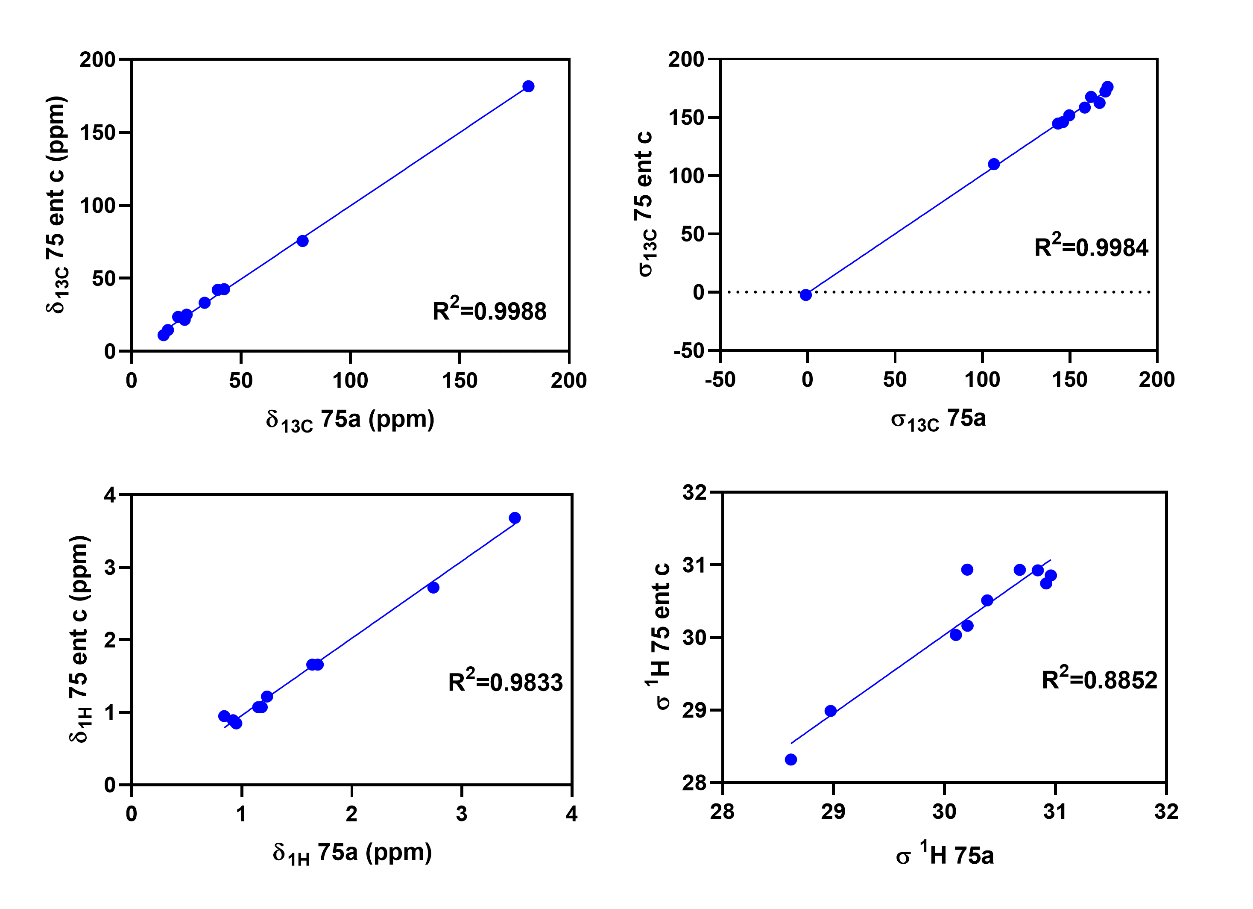


**Figure S32****.** Chemical shift correlation plot between experimental (δ) and DFT calculated (σ) of the apparent epimers **75a** and **75 *ent* c**. The coefficient of correlation (R²) is shown in both cases.


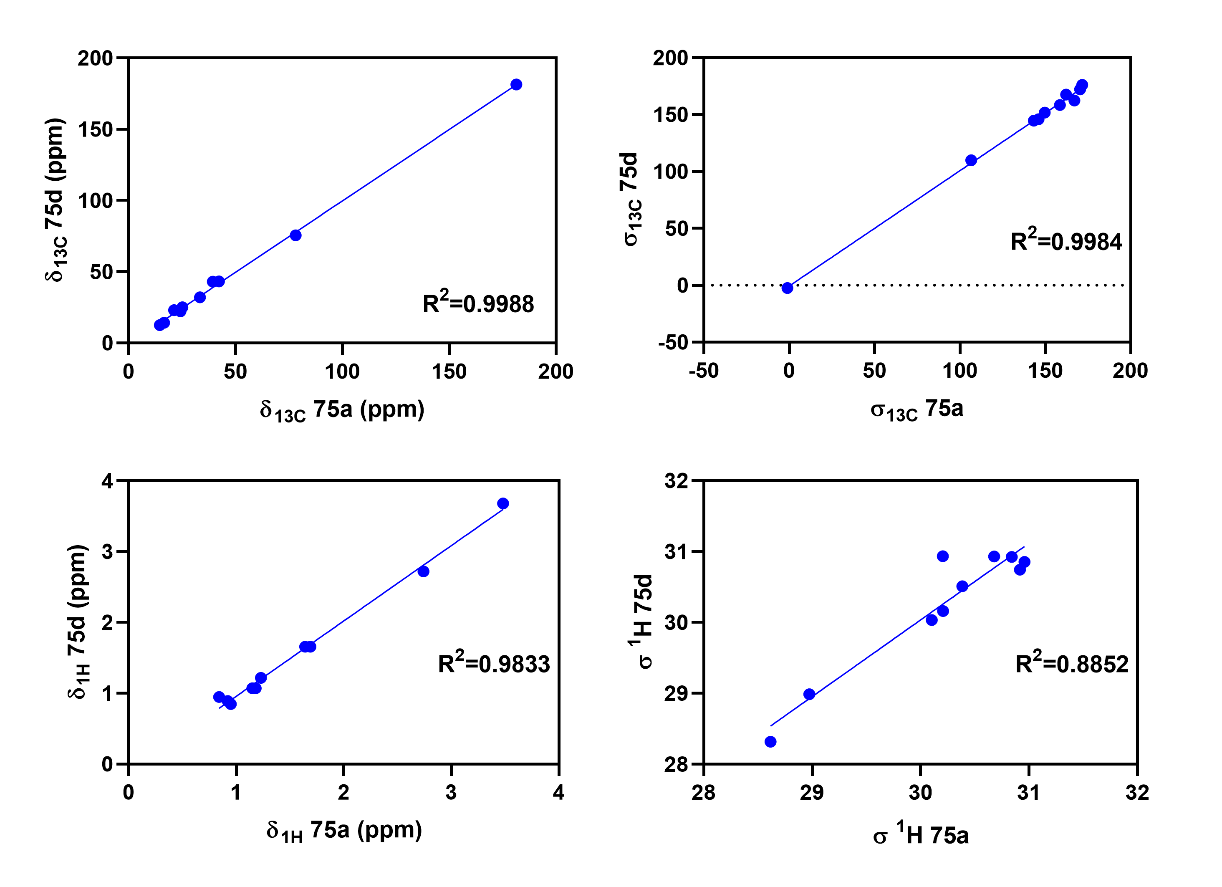


**Figure S33.** Chemical shift correlation plot between experimental (δ) and DFT calculated (σ) epimers **75a** and **75d**. The coefficient of correlation (R²) is shown in both cases.


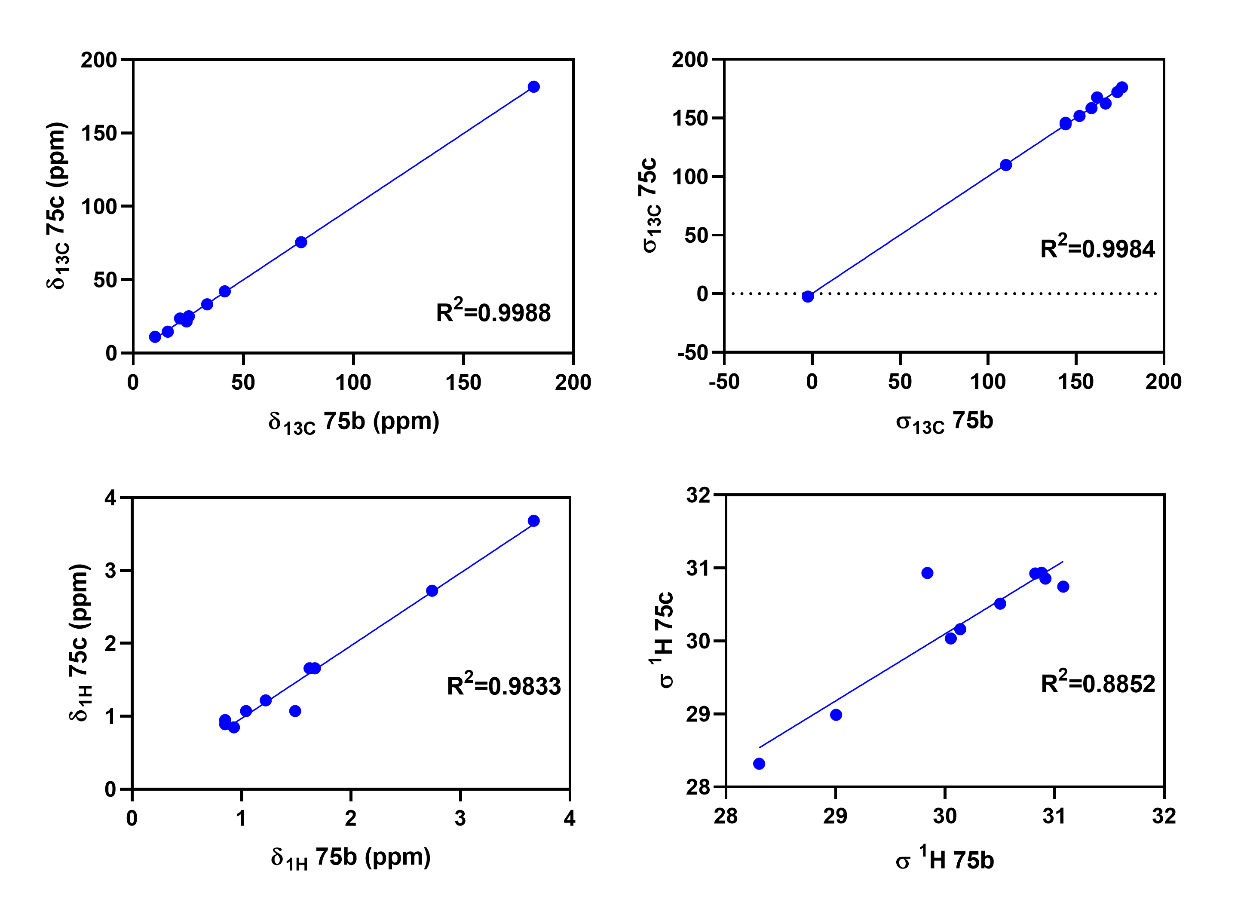


**Figure S34.** Chemical shift correlation plot between experimental (δ) and DFT calculated (σ) epimers **75b** and **75c**. The coefficient of correlation (R²) is shown in both cases.


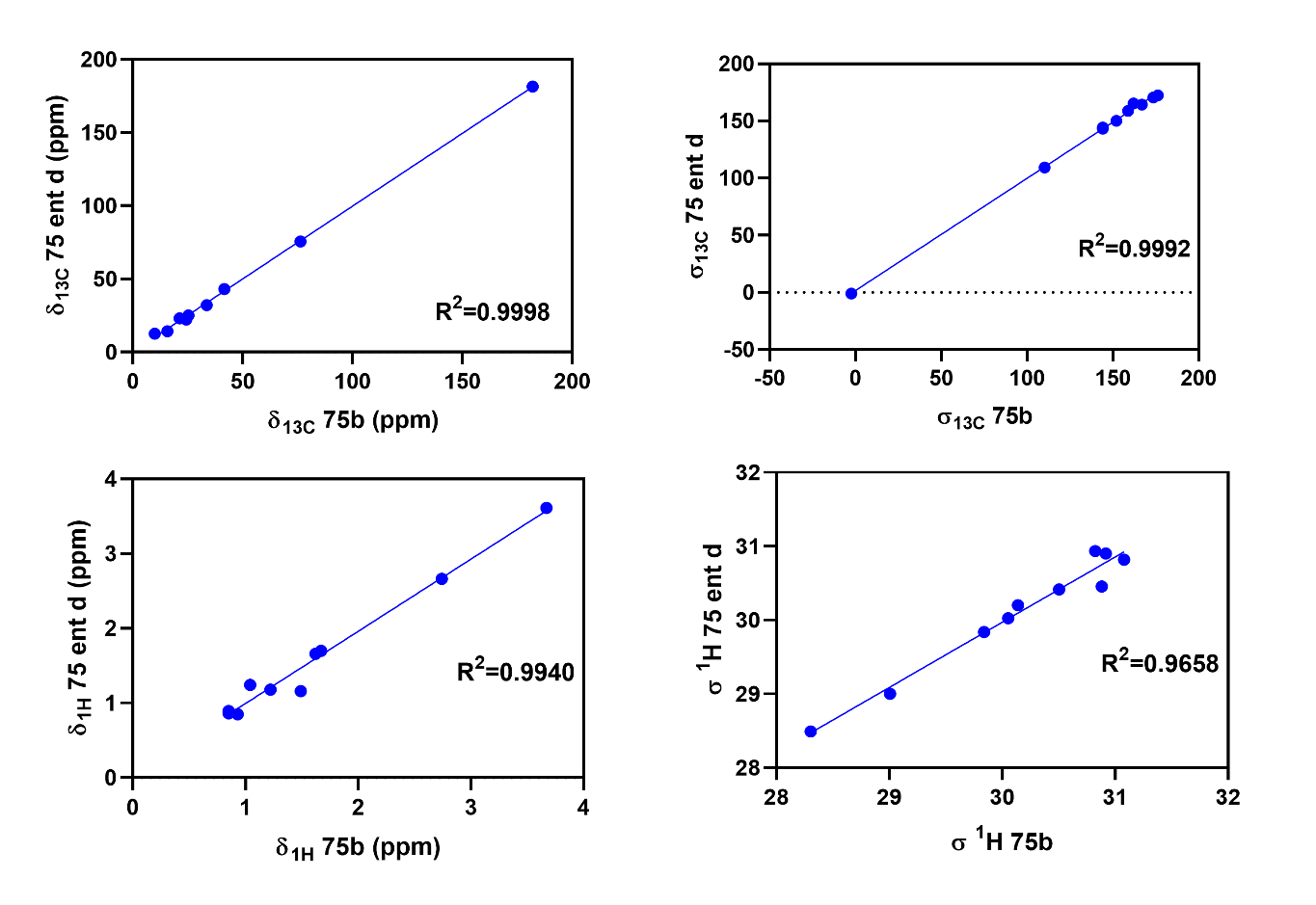


**Figure S35**. Chemical shift correlation plot between experimental (δ) and DFT calculated (σ) epimers **75b** and **75 *ent* d**. The coefficient of correlation (R²) is shown in both cases.


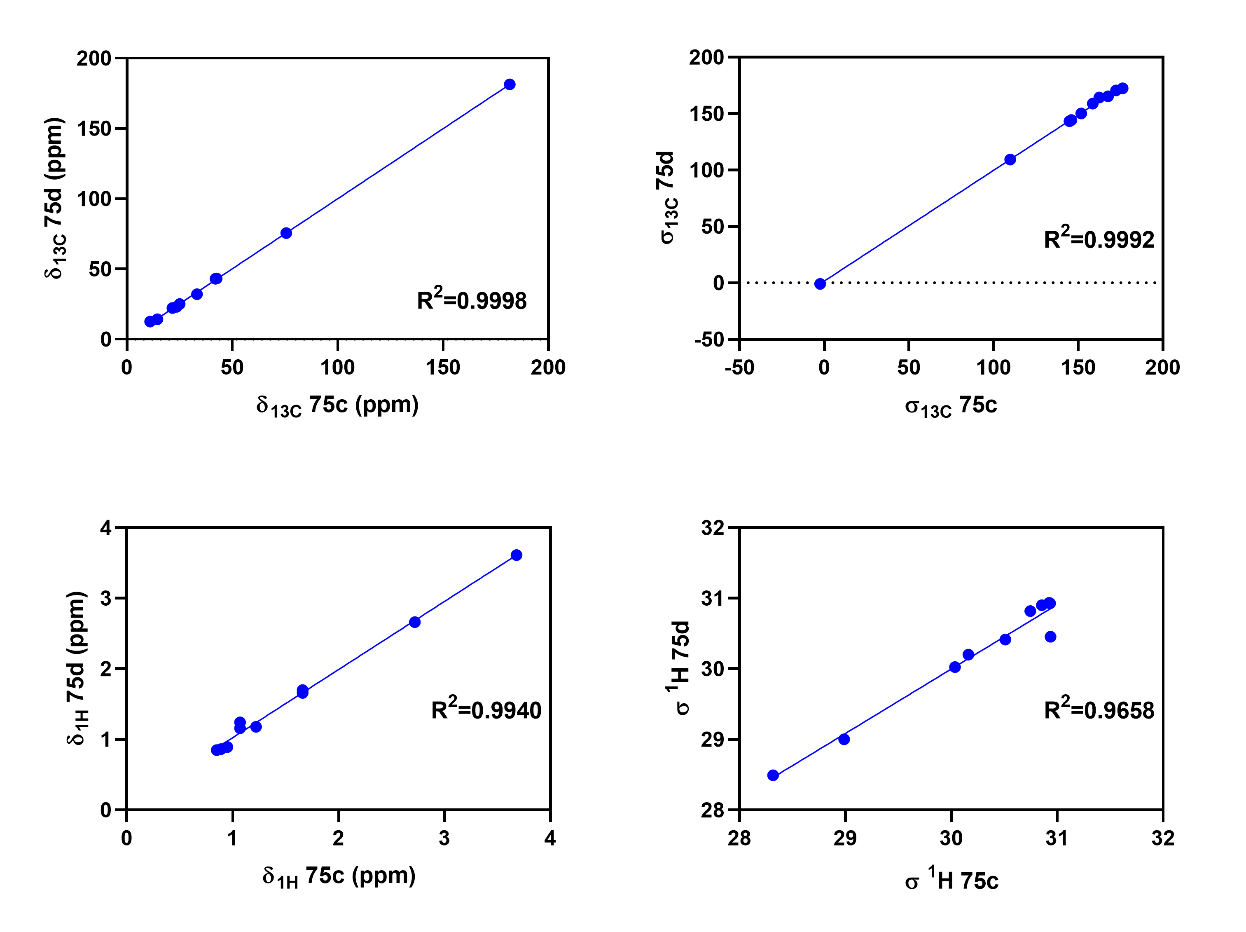


**Figure S36.** Chemical shift correlation plot between **75c** and **75d**. The coefficient of correlation (R²) is shown in both cases.


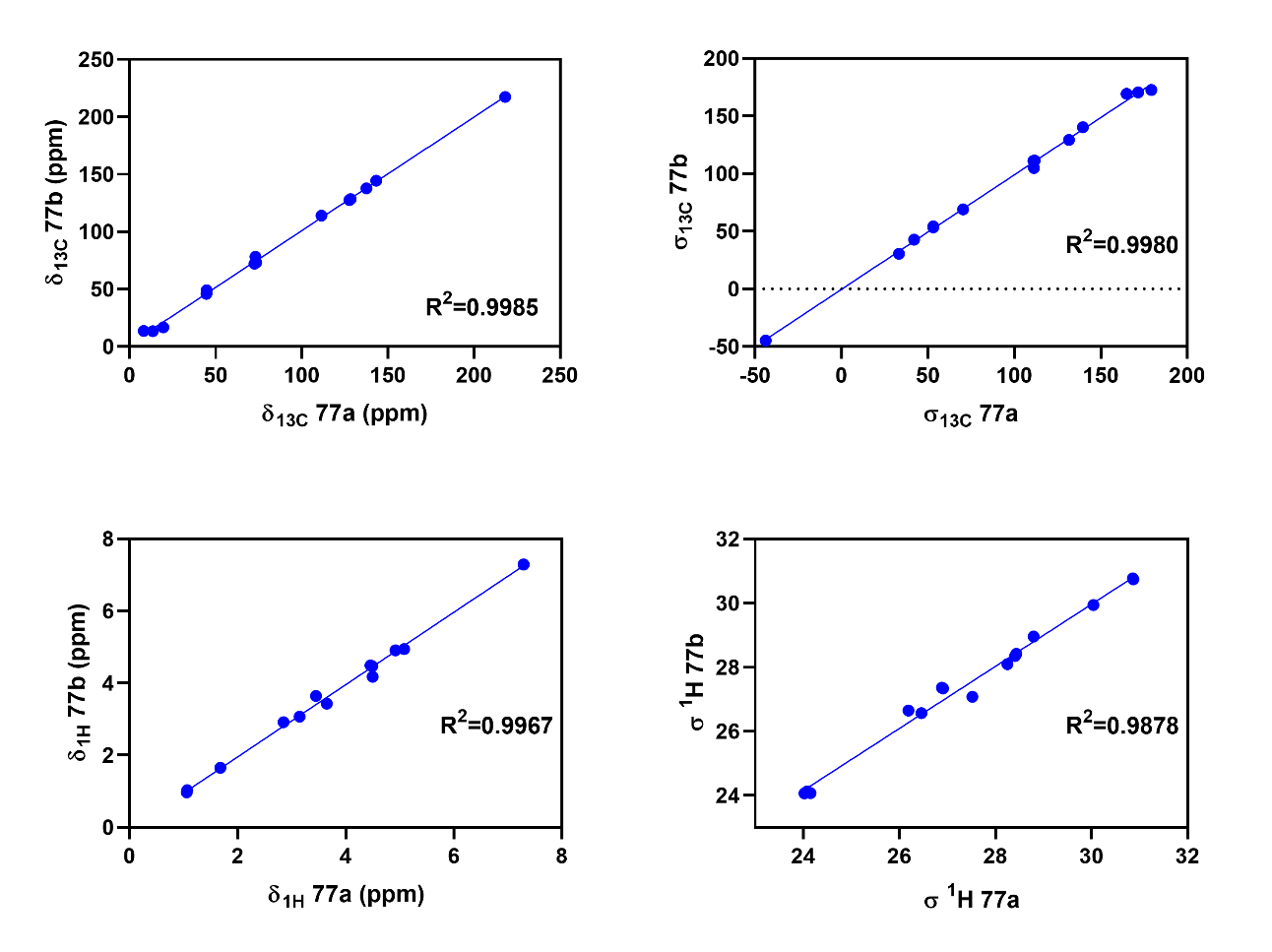


**Figure S37.** Chemical shift correlation plot between experimental (δ) and DFT calculated (σ) epimers **77a** and **77b**. The coefficient of correlation (R²) is shown in both cases.


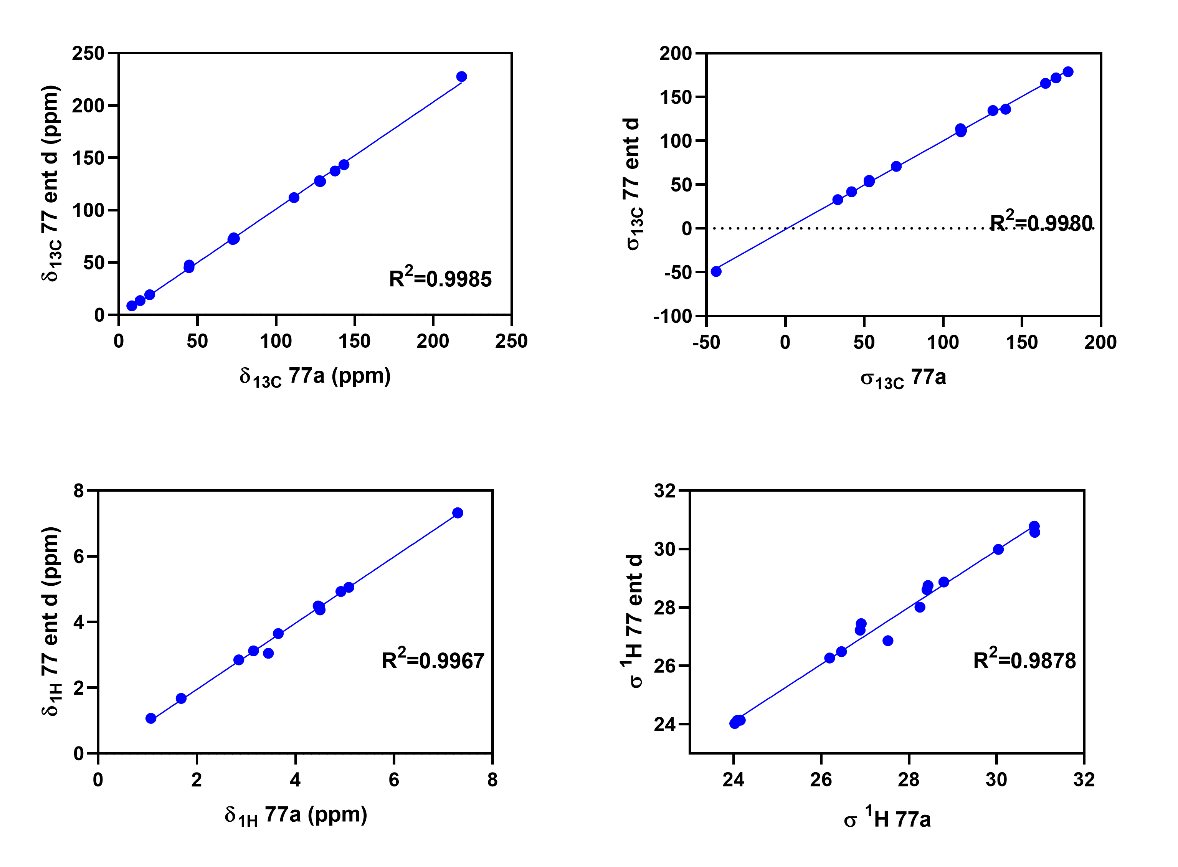


**Figure S38**. Chemical shift correlation plot between experimental (δ) and DFT calculated (σ) epimers **77a** and **77 *ent* d**. The coefficient of correlation (R²) is shown in both cases.


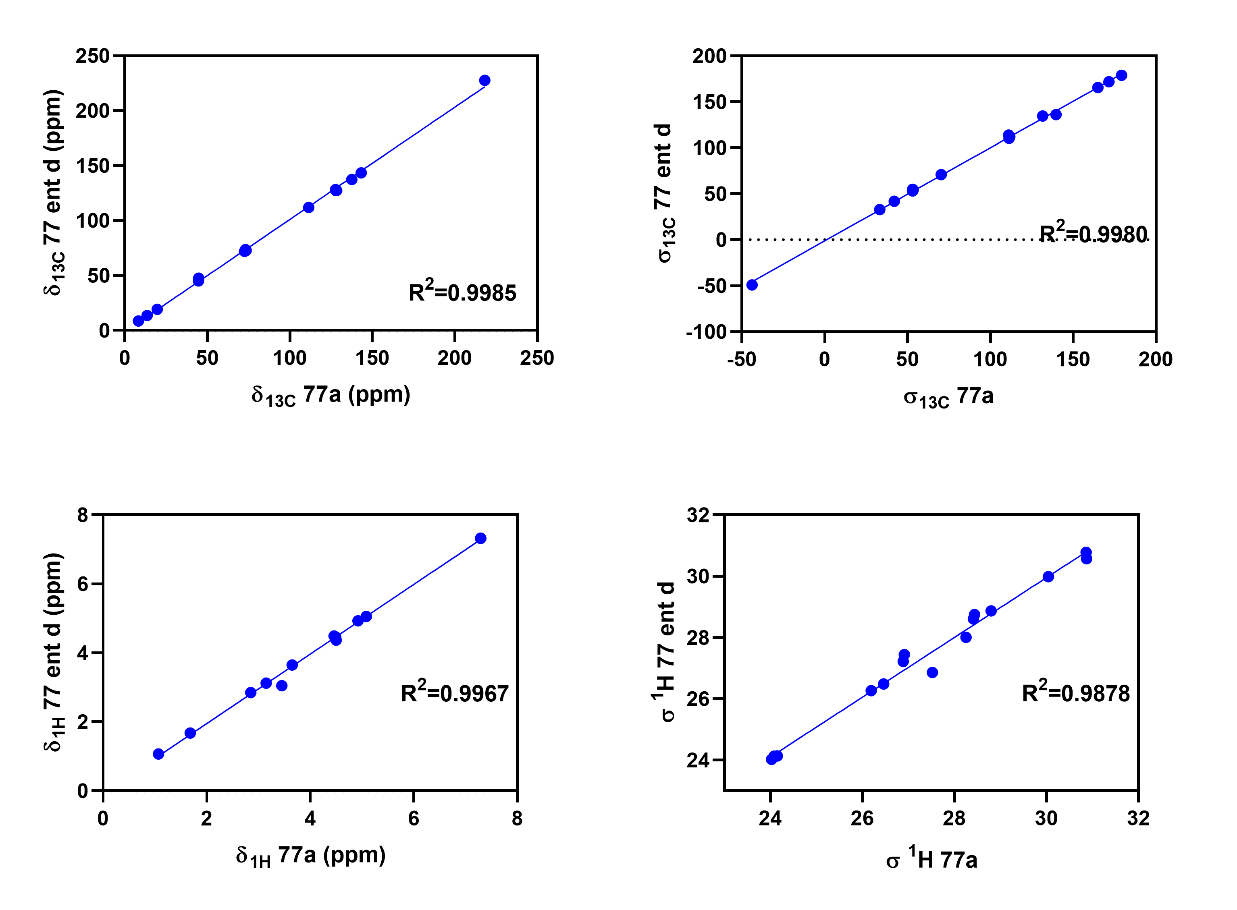


**Figure S39.** Chemical shift correlation plot between experimental (δ) and DFT calculated (σ) epimers **77a** and **77 *ent* d**. The coefficient of correlation (R²) is shown in both cases.


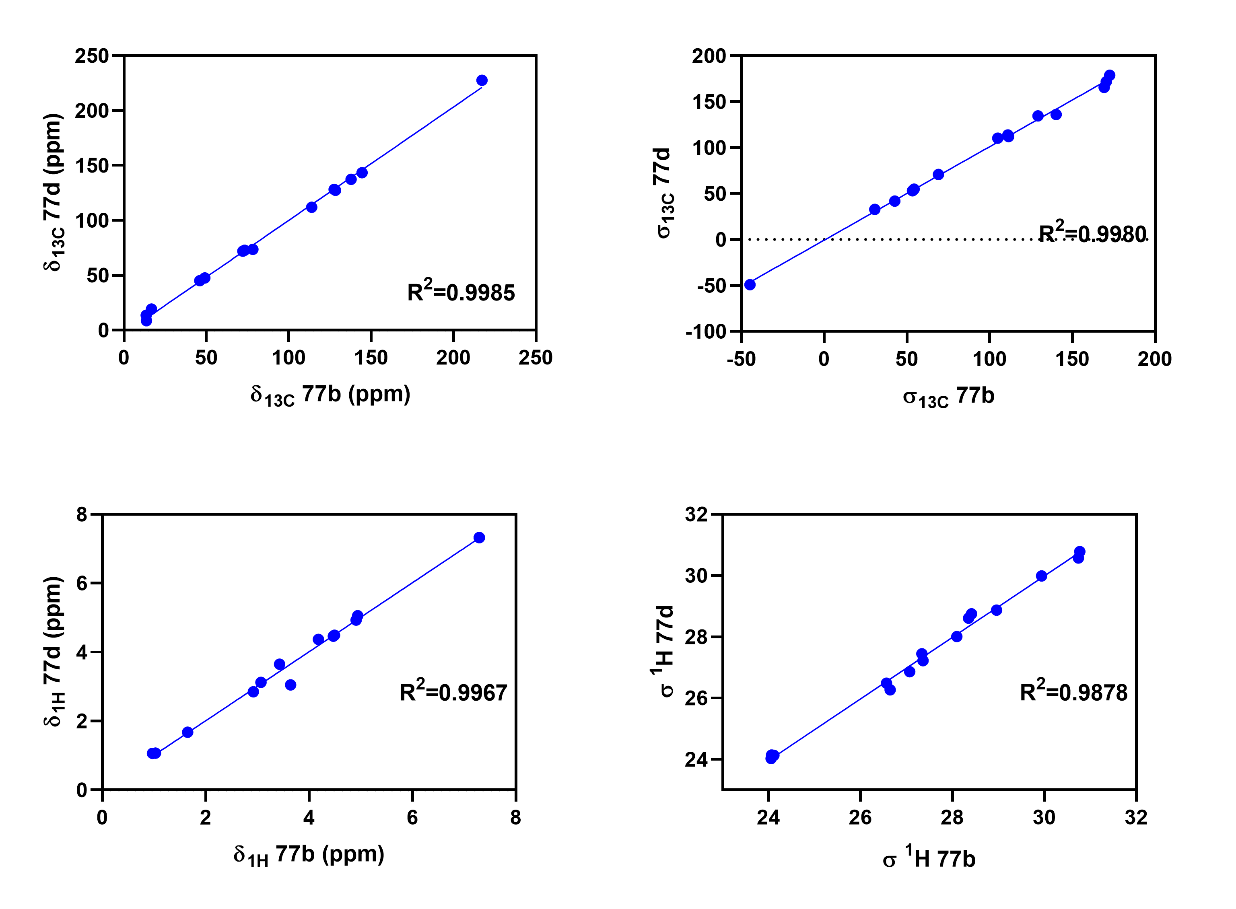


**Figure S40.** Chemical shift correlation plot between experimental (δ) and DFT calculated (σ) epimers **77b** and **77d**. The coefficient of correlation (R²) is shown in both cases.


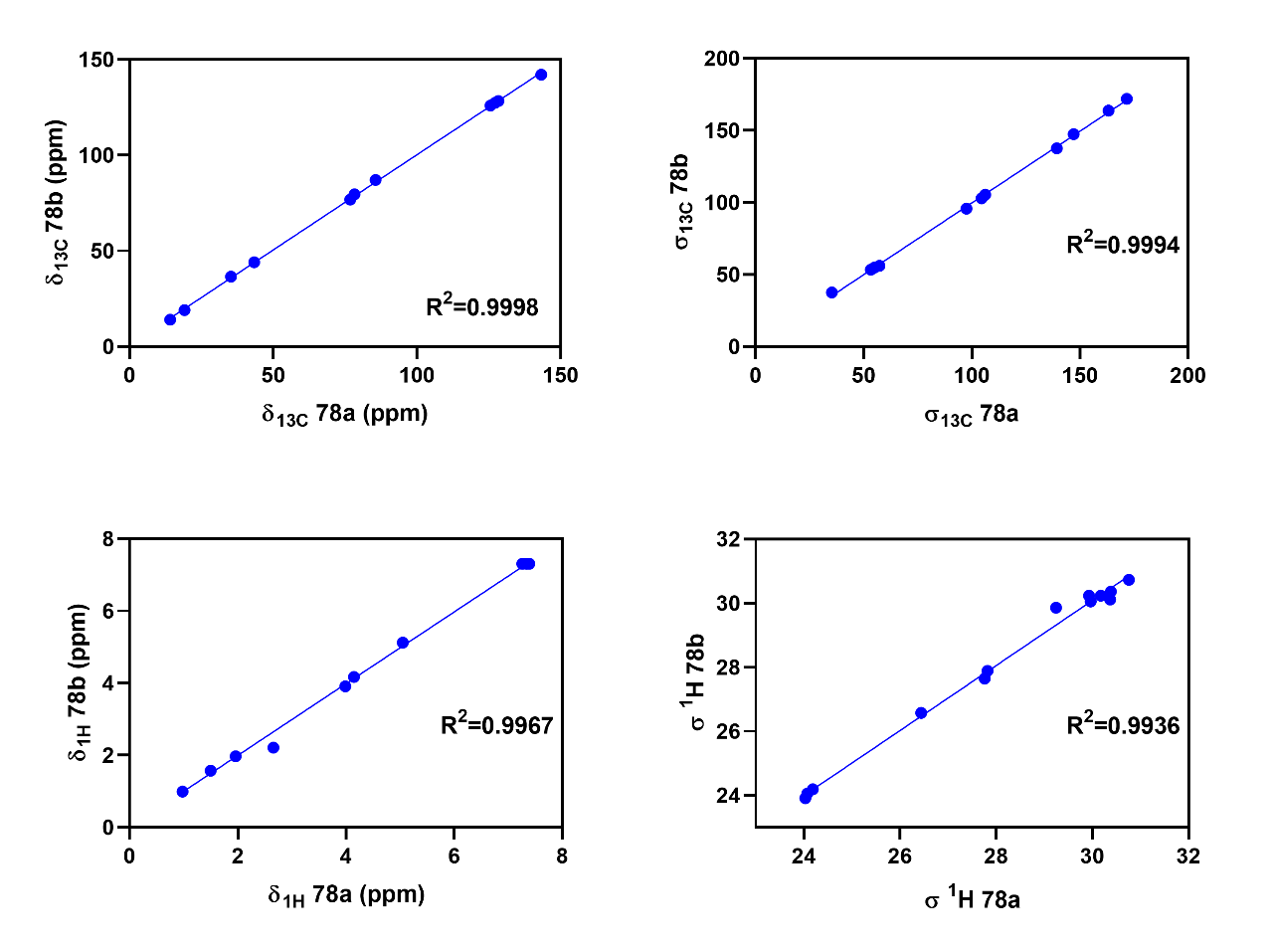


**Figure S41.** Chemical shift correlation plot between experimental (δ) and DFT calculated (σ) epimers **78a** and **78b**. The coefficient of correlation (R²) is shown in both cases.


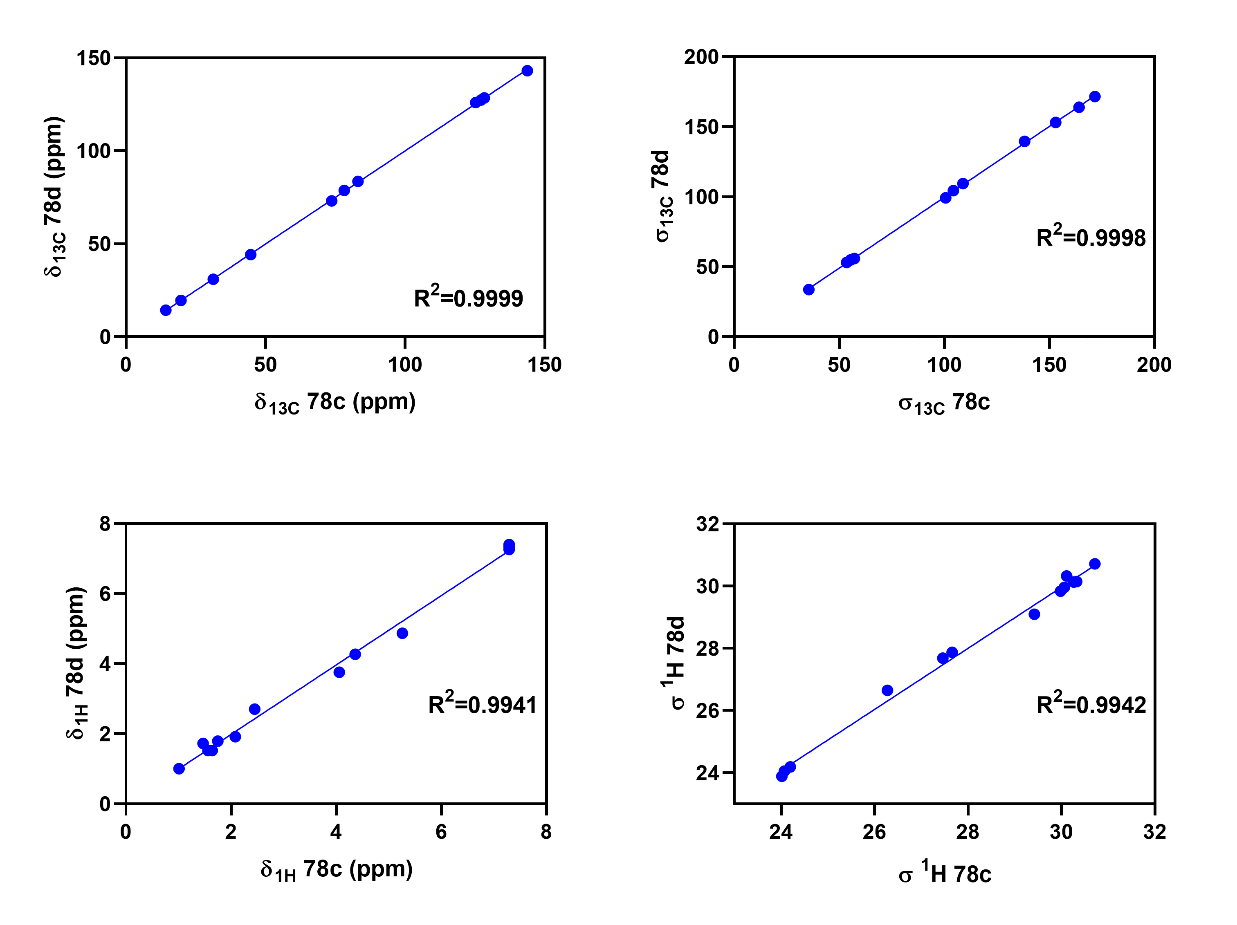


**Figure S42.** Chemical shift correlation plot between experimental (δ) and DFT calculated (σ) epimers **78c** and **78d**. The coefficient of correlation (R²) is shown in both cases.


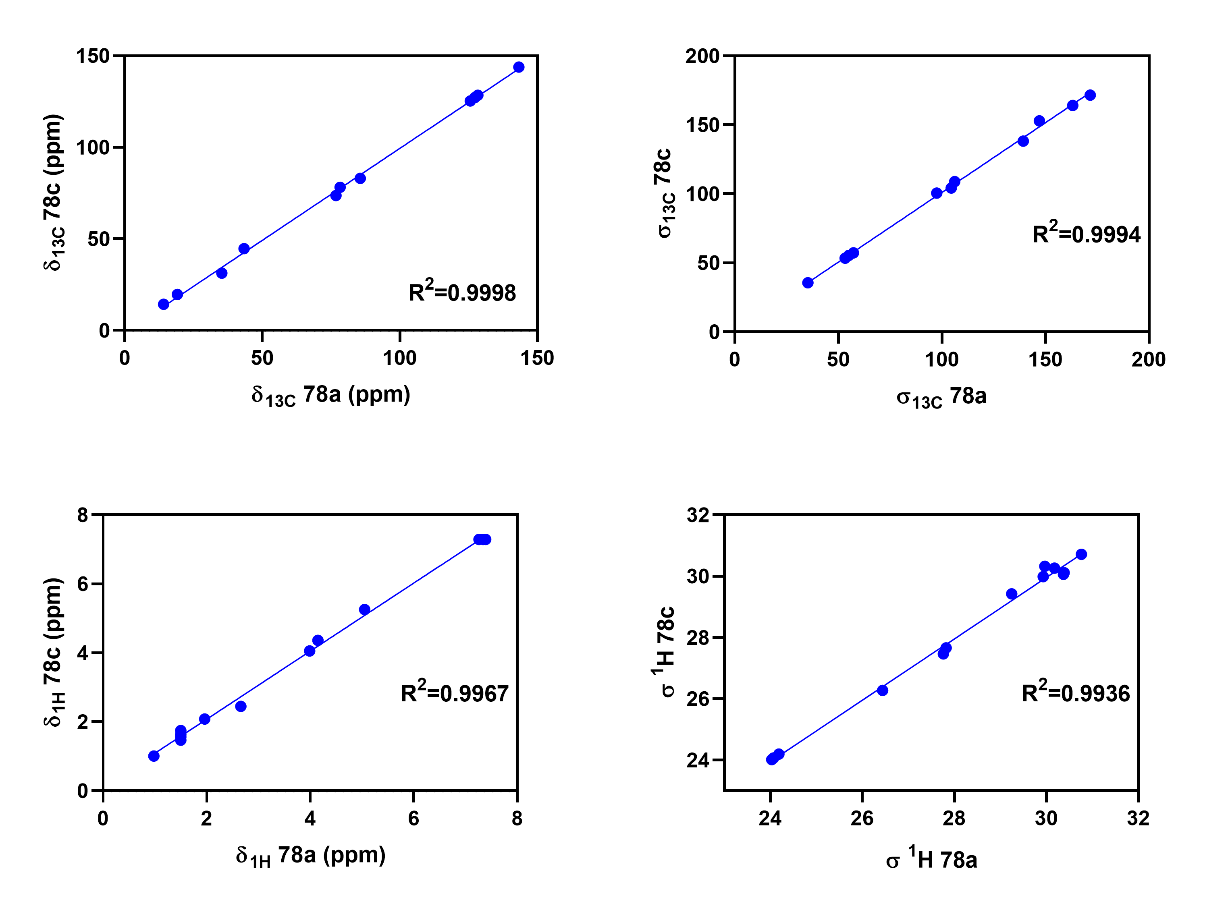


**Figure S43**. Chemical shift correlation plot between experimental (δ) and DFT calculated (σ) epimers **78a** and **78c**. The coefficient of correlation (R²) is shown in both cases.


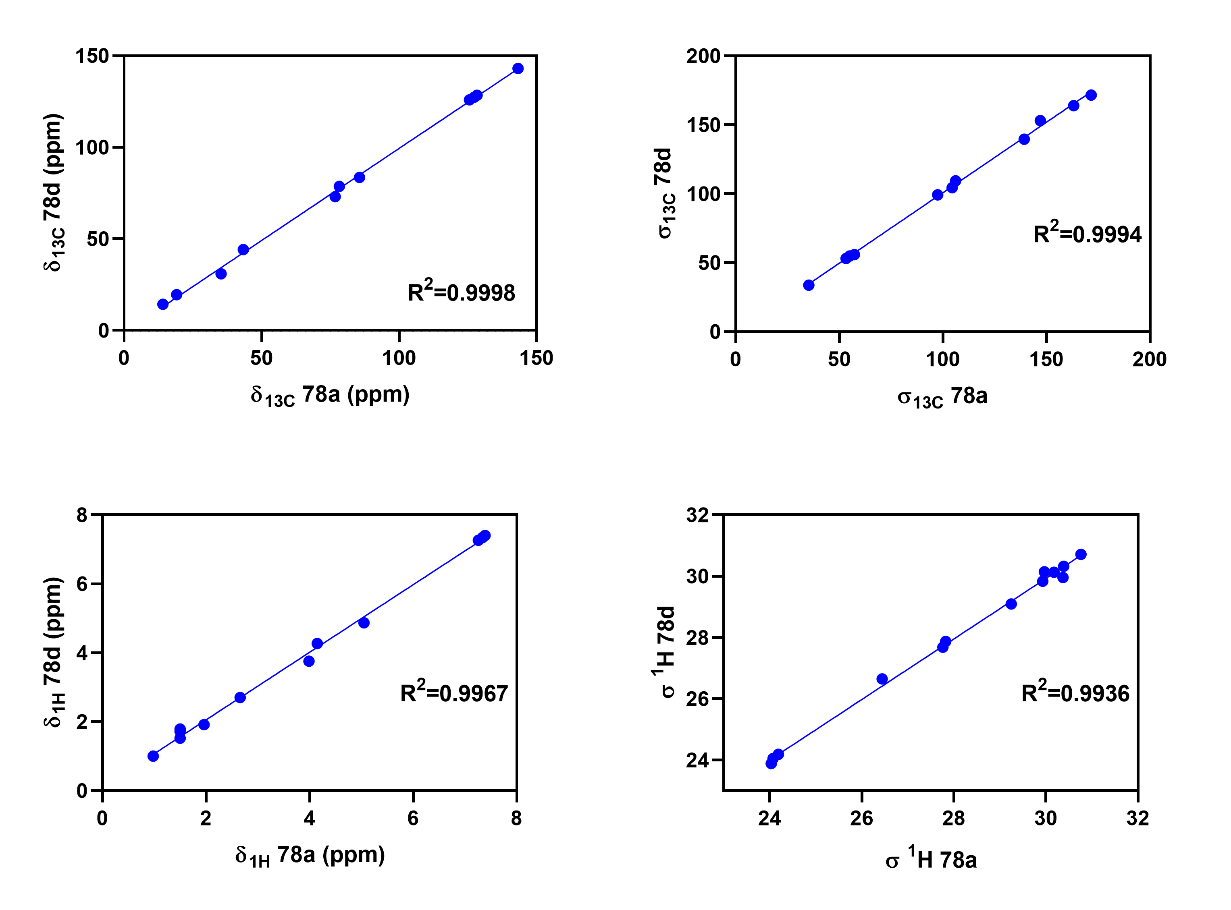


**Figure S44.** Chemical shift correlation plot between experimental (δ) and DFT calculated (σ) epimers **78a** and **78d**. The coefficient of correlation (R²) is shown in both cases.


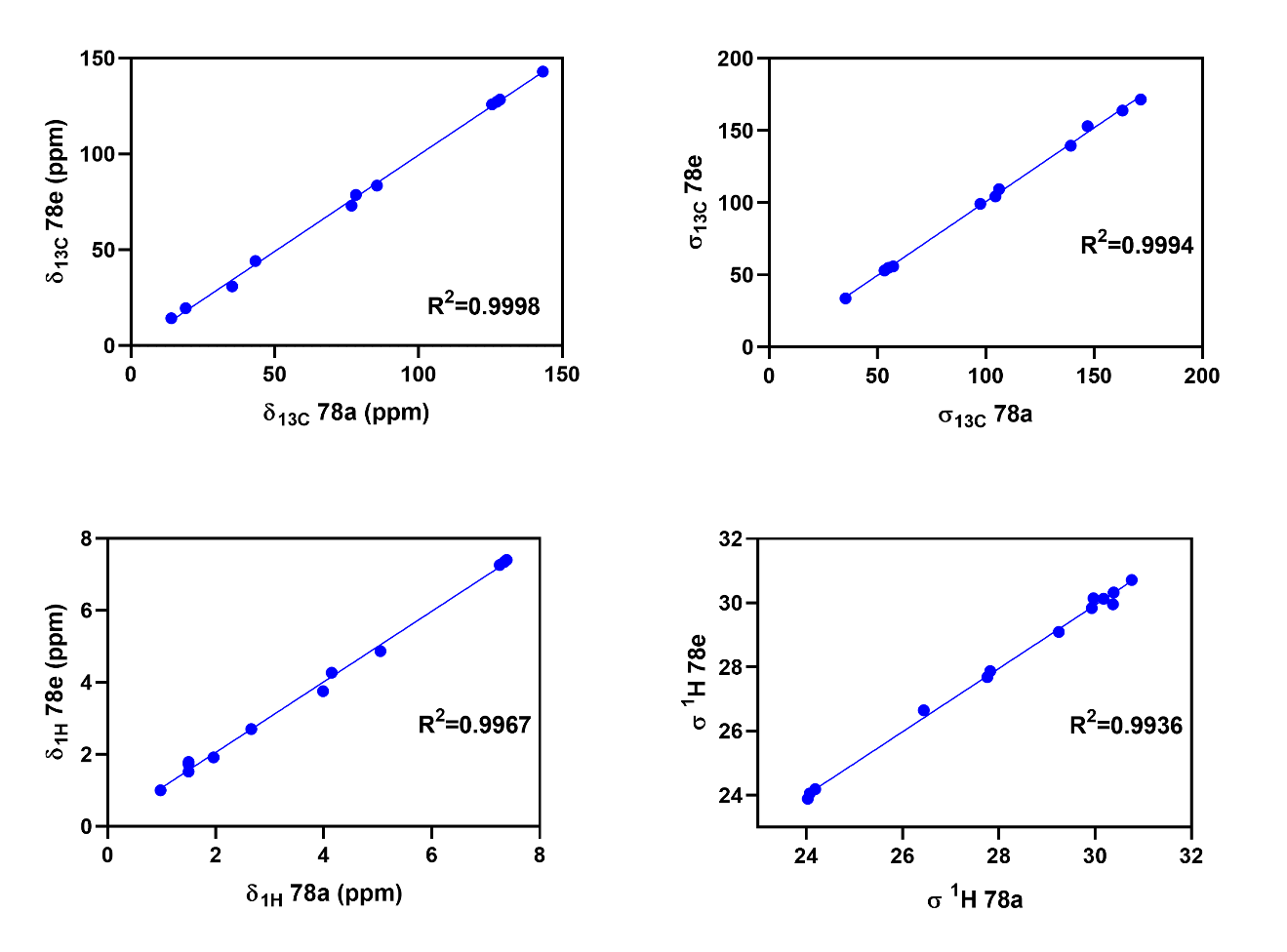


**Figure S45.** Chemical shift correlation plot between experimental (δ) and DFT calculated (σ) epimers **78a** and **78e**. The coefficient of correlation (R²) is shown in both cases.


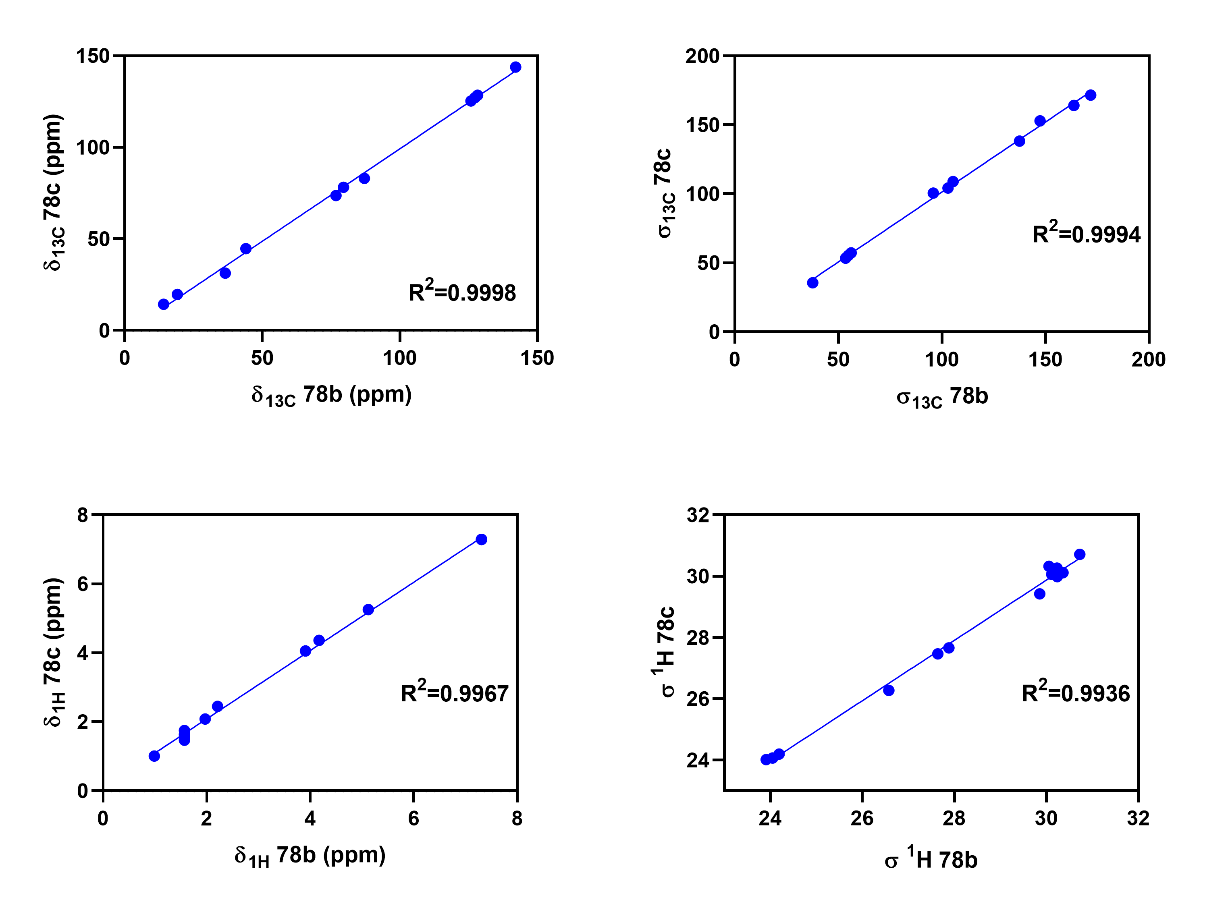


**Figure S46**. Chemical shift correlation plot between experimental (δ) and DFT calculated (σ) epimers **78b** and **78c**. The coefficient of correlation (R²) is shown in both cases.


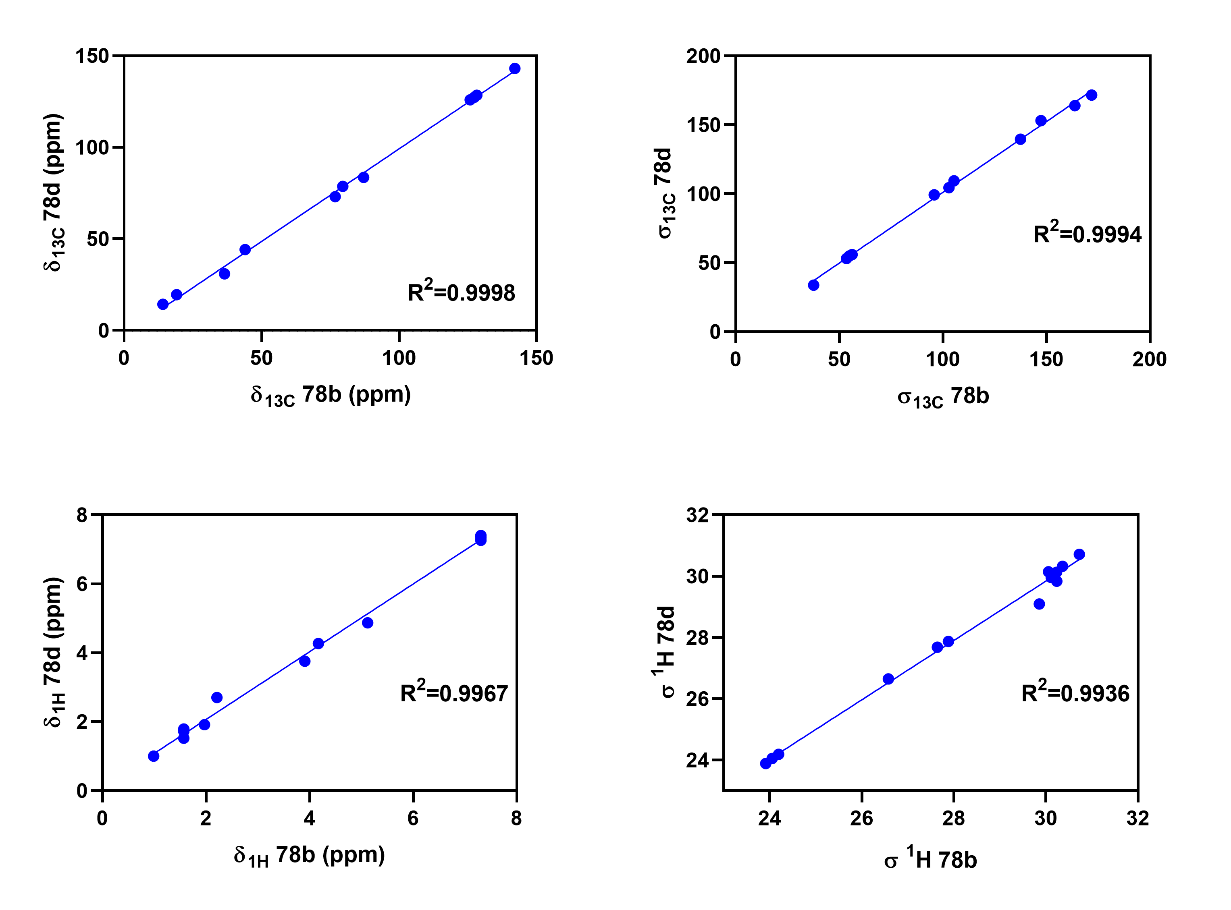


**Figure S47.** Chemical shift correlation plot between experimental (δ) and DFT calculated (σ) epimers **78b** and **78d.** The coefficient of correlation (R²) is shown in both cases.


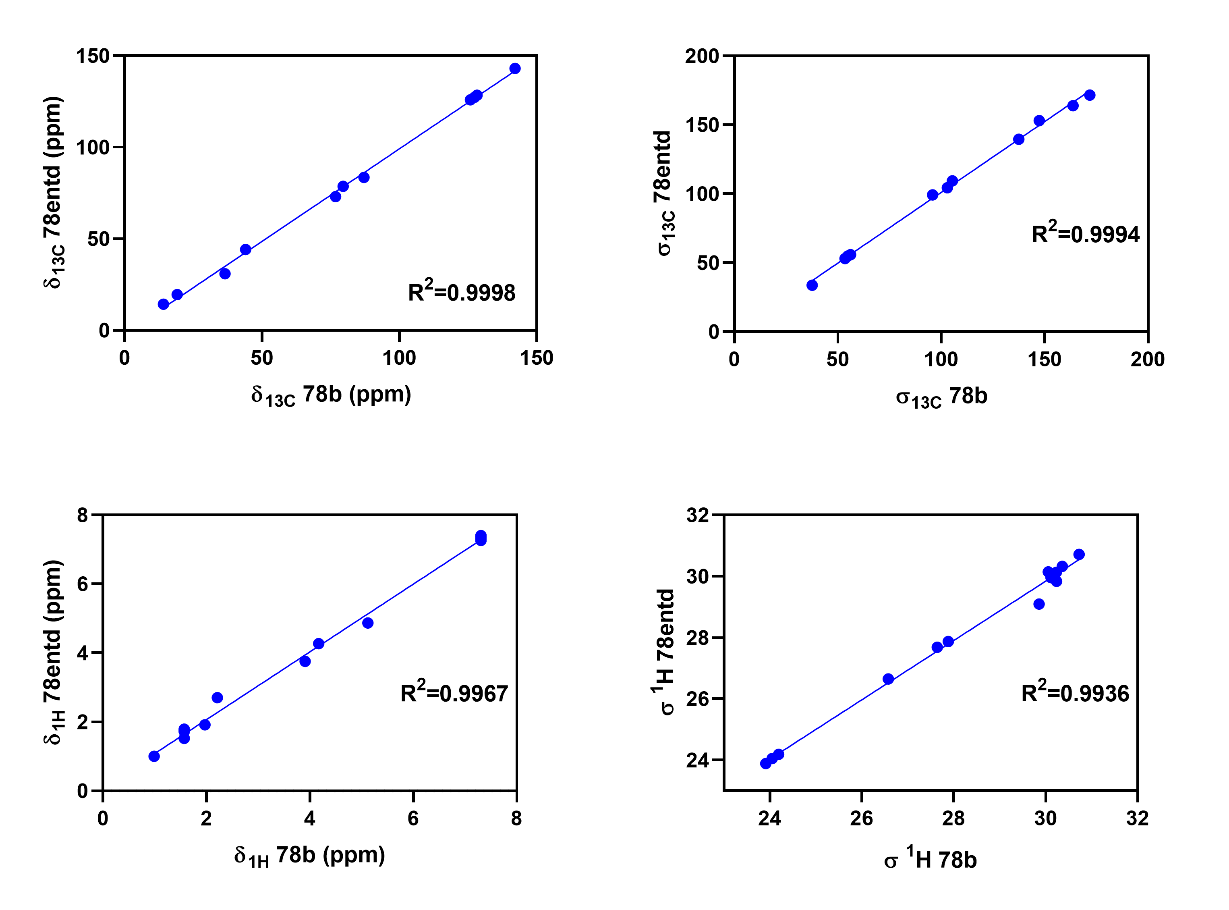


**Figure S48.** Chemical shift correlation plot between experimental (δ) and DFT calculated (σ) epimers **78b** and **78 *ent* d**. The coefficient of correlation (R²) is shown in both cases.


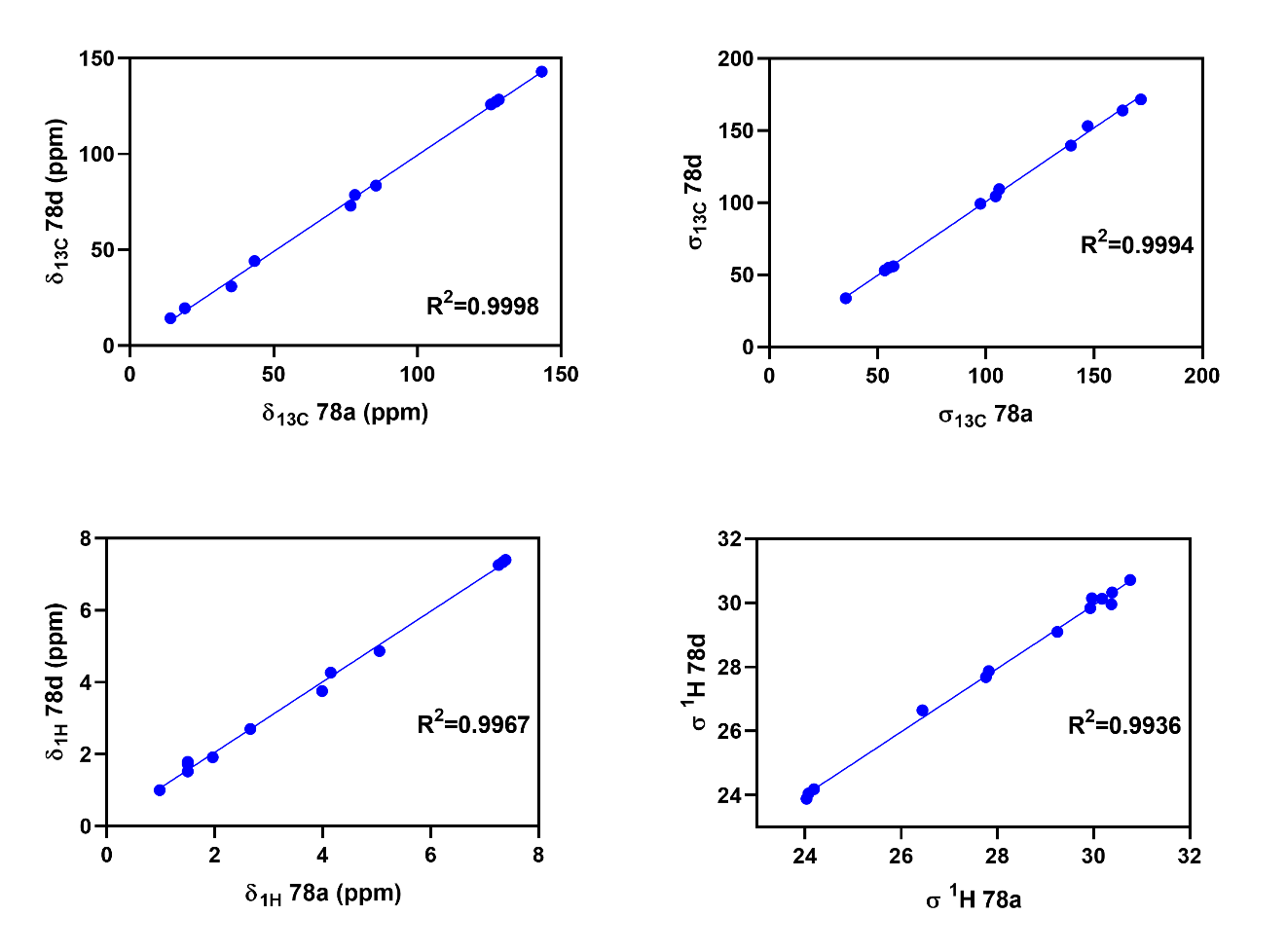


**Figure S49.** Chemical shift correlation plot between experimental (δ) and DFT calculated (σ) epimers **78a** and **78d**. The coefficient of correlation (R²) is shown in both cases.


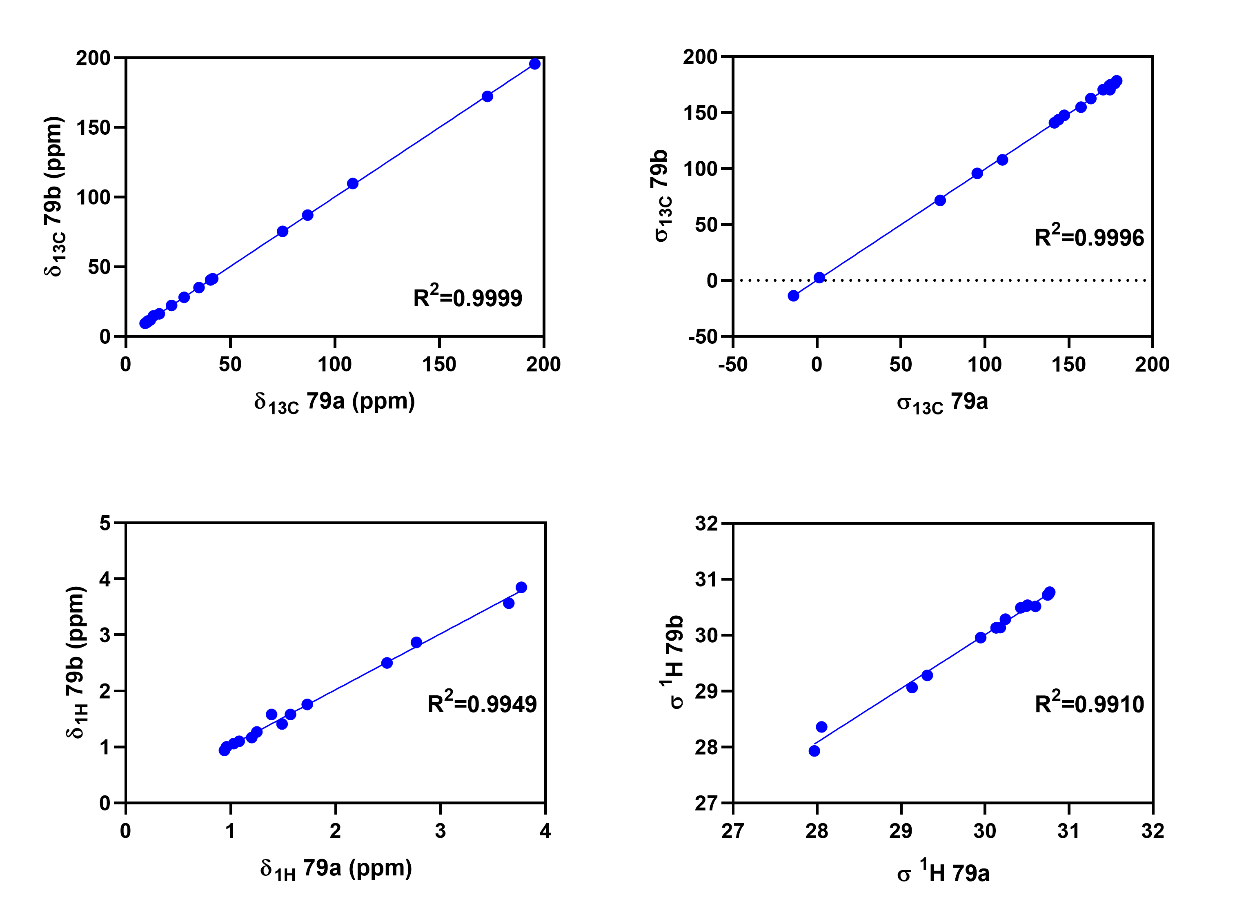


**Figure S50.** Chemical shift correlation plot between experimental (δ) and DFT calculated (σ) epimers **79a** and **79b**. The coefficient of correlation (R²) is shown in both cases.


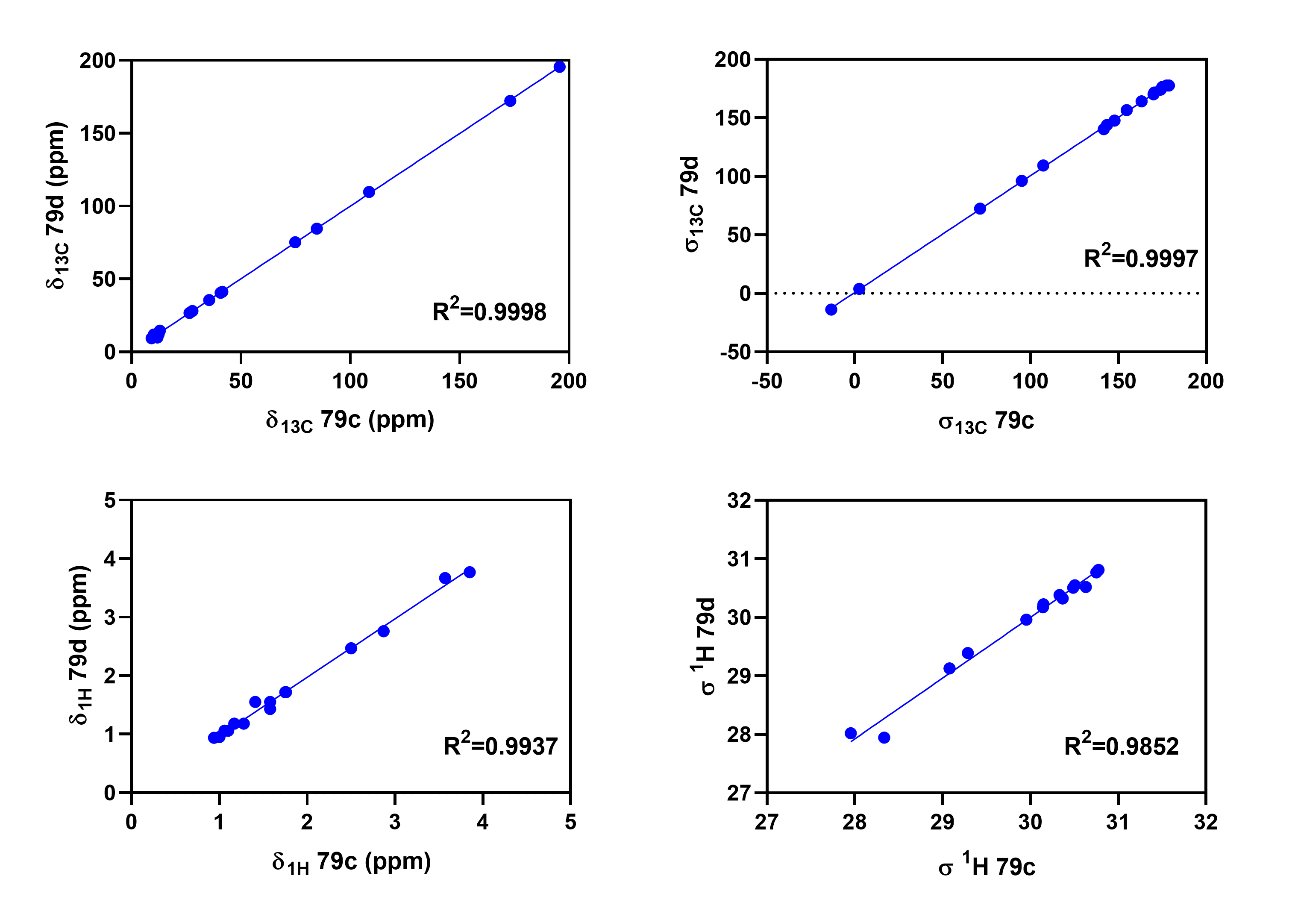


**Figure S51.** Chemical shift correlation plot between experimental (δ) and DFT calculated (σ) epimers **79c** and **79d**. The coefficient of correlation (R²) is shown in both cases.


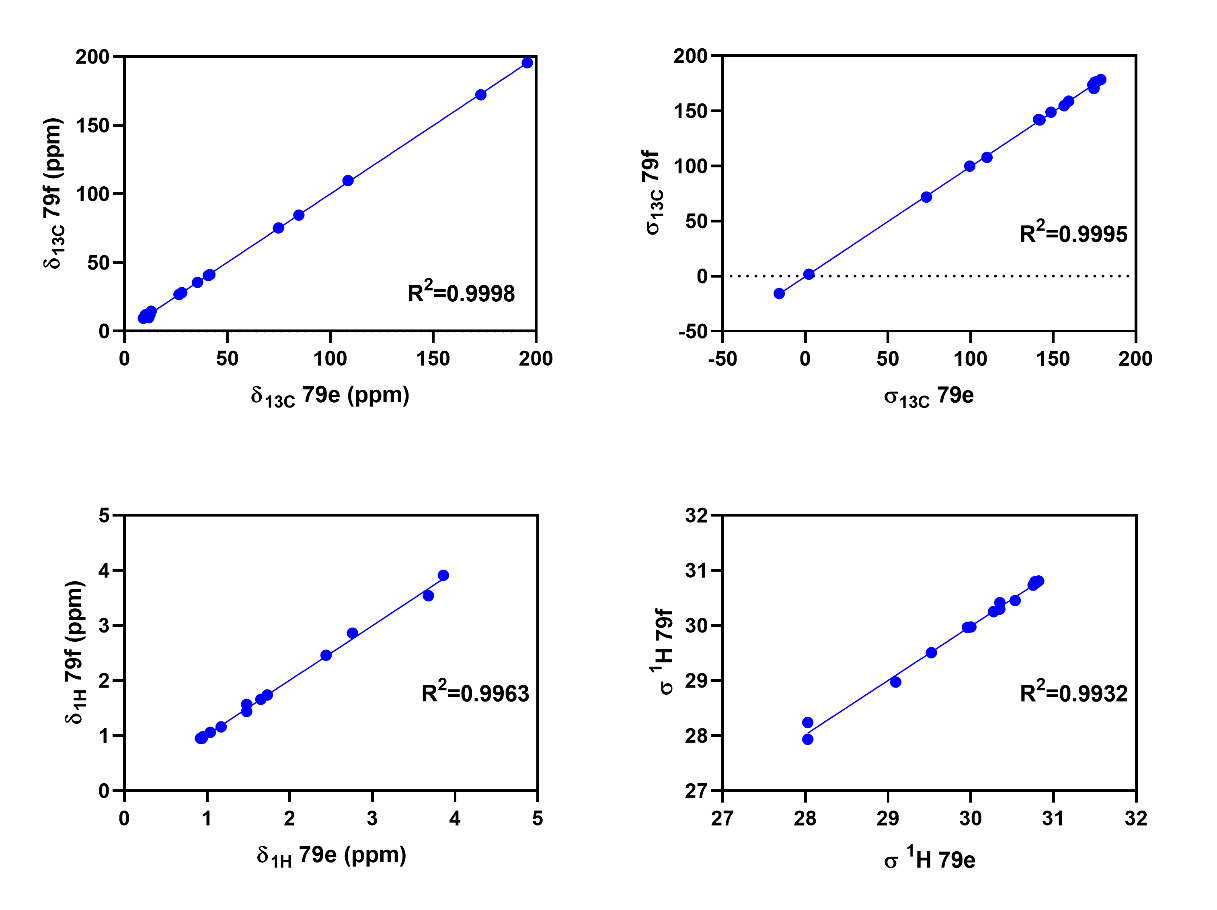


**Figure S52.** Chemical shift correlation plot between experimental (δ) and DFT calculated (σ) epimers **79e** and **79f**. The coefficient of correlation (R²) is shown in both cases.


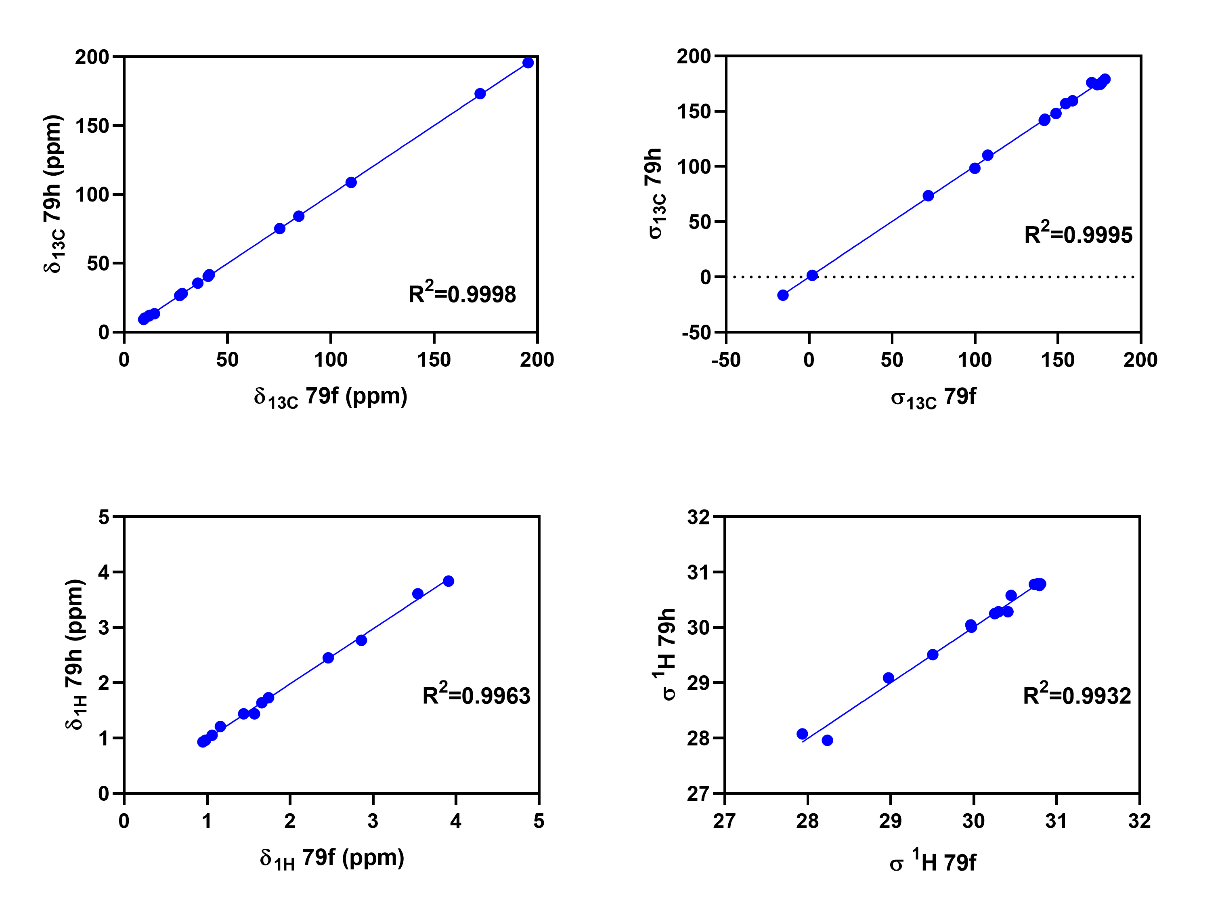


**Figure S53.** Chemical shift correlation plot between experimental (δ) and DFT calculated (σ) epimers **79f** and **79h**. The coefficient of correlation (R²) is shown in both cases.


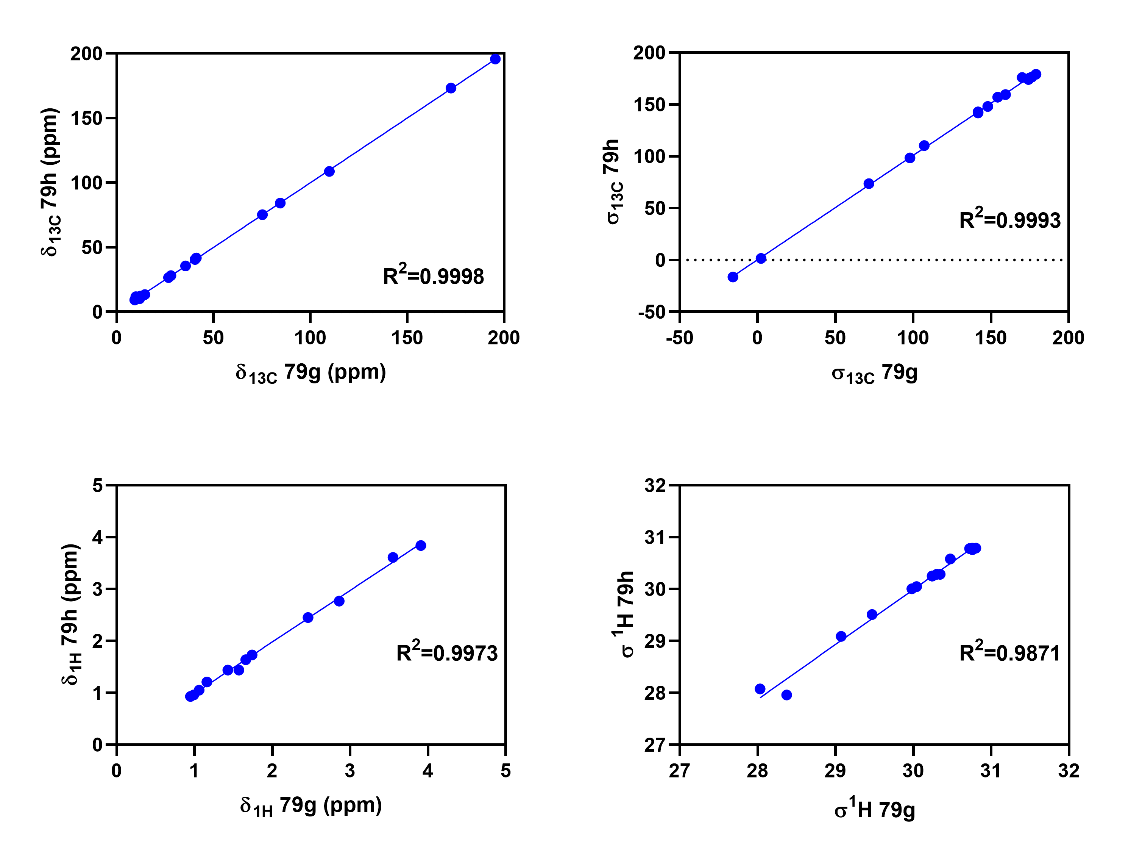


**Figure S54.** Chemical shift correlation plot between experimental (δ) and DFT calculated (σ) epimers **79g** and **79h**. The coefficient of correlation (R²) is shown in both cases.


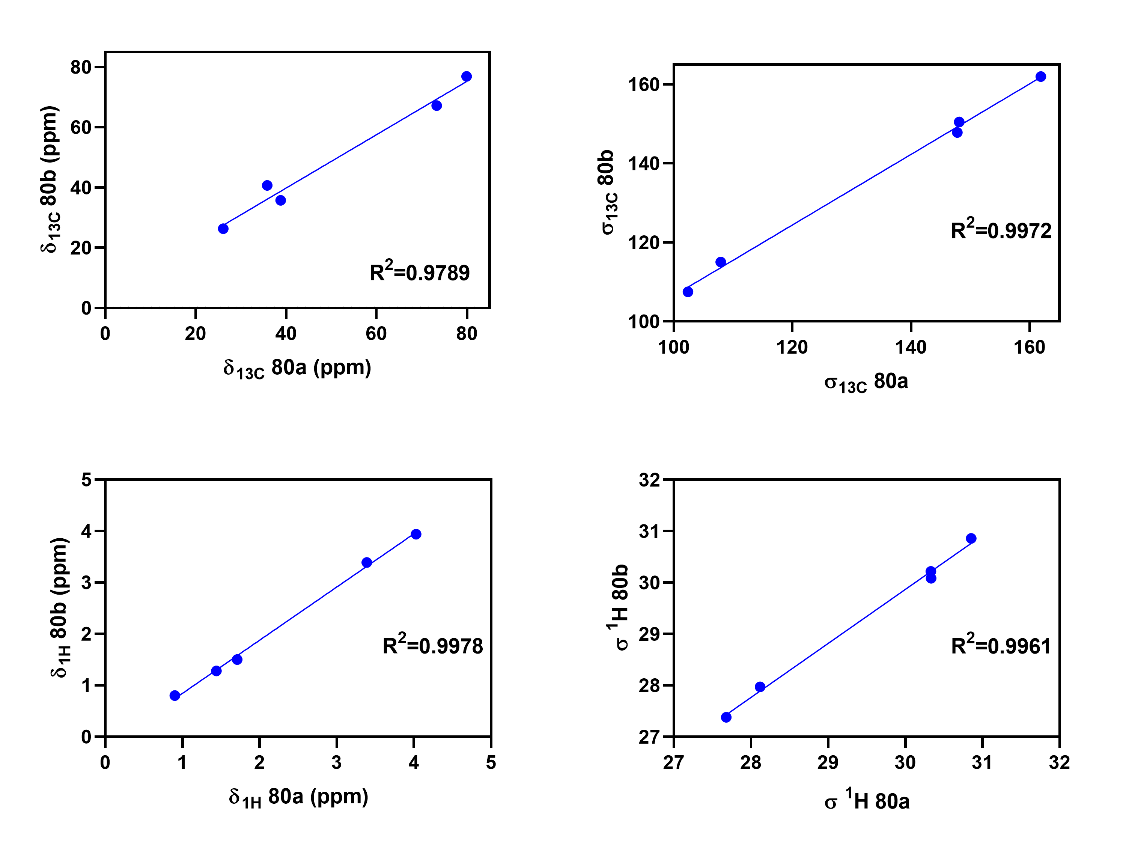


**Figure S55.** Chemical shift correlation plot between experimental (δ) and DFT calculated (σ) epimers **80a** and **80b**. The coefficient of correlation (R²) is shown in both cases.


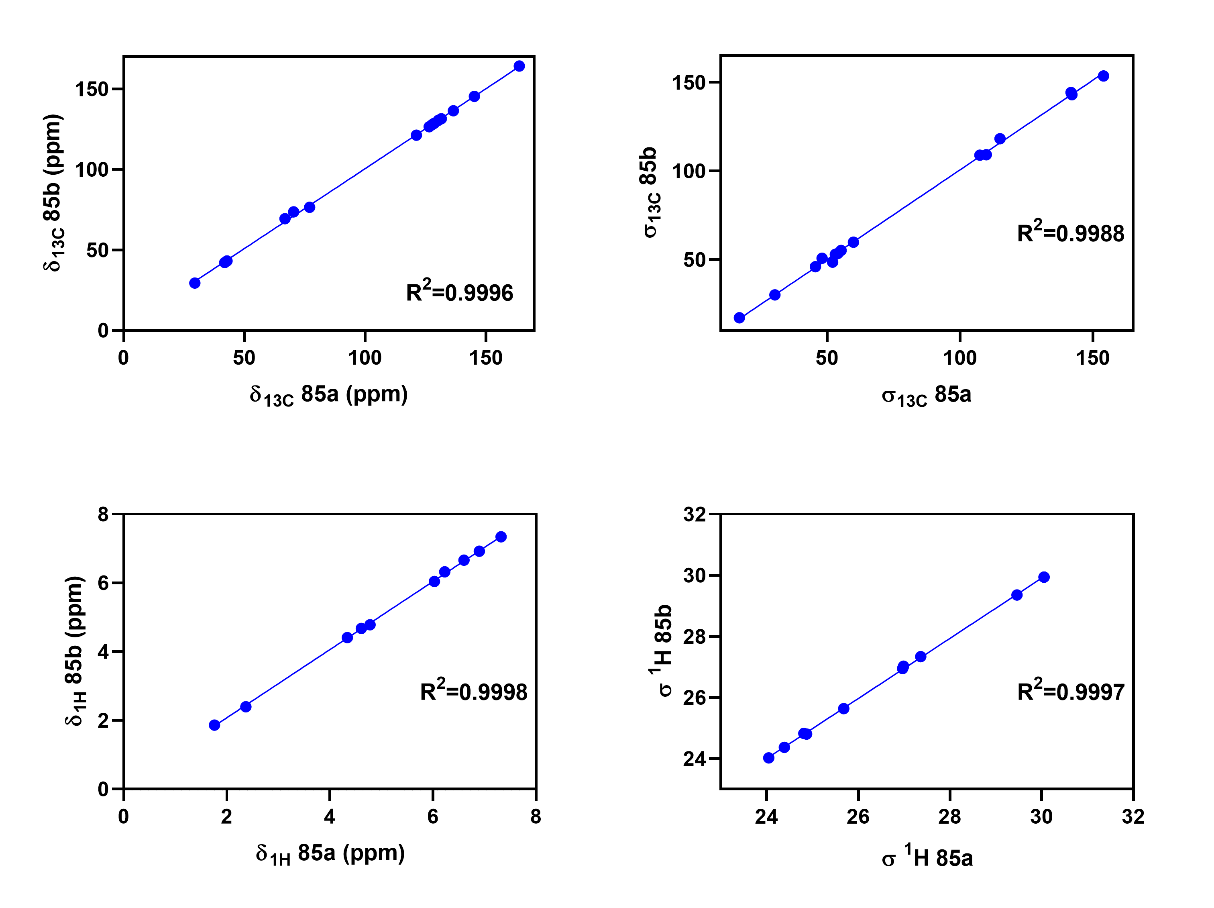


**Figure S56.** Chemical shift correlation plot between experimental (δ) and DFT calculated (σ) epimers **85a** and **85b**. The coefficient of correlation (R²) is shown in both cases.


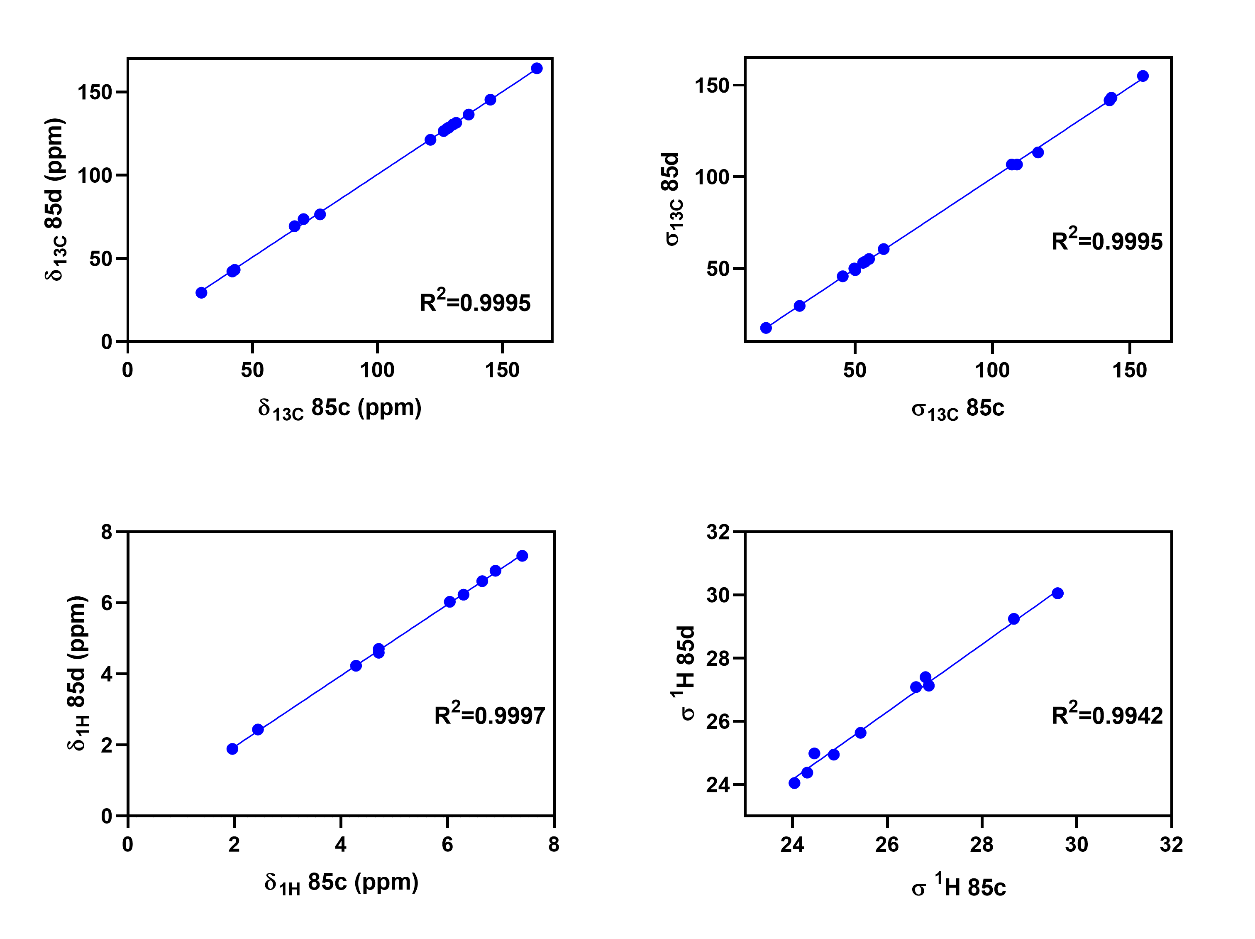


**Figure S57.** Chemical shift correlation plot between experimental (δ) and DFT calculated (σ) epimers **85c** and **85d** The coefficient of correlation (R²) is shown in both cases.


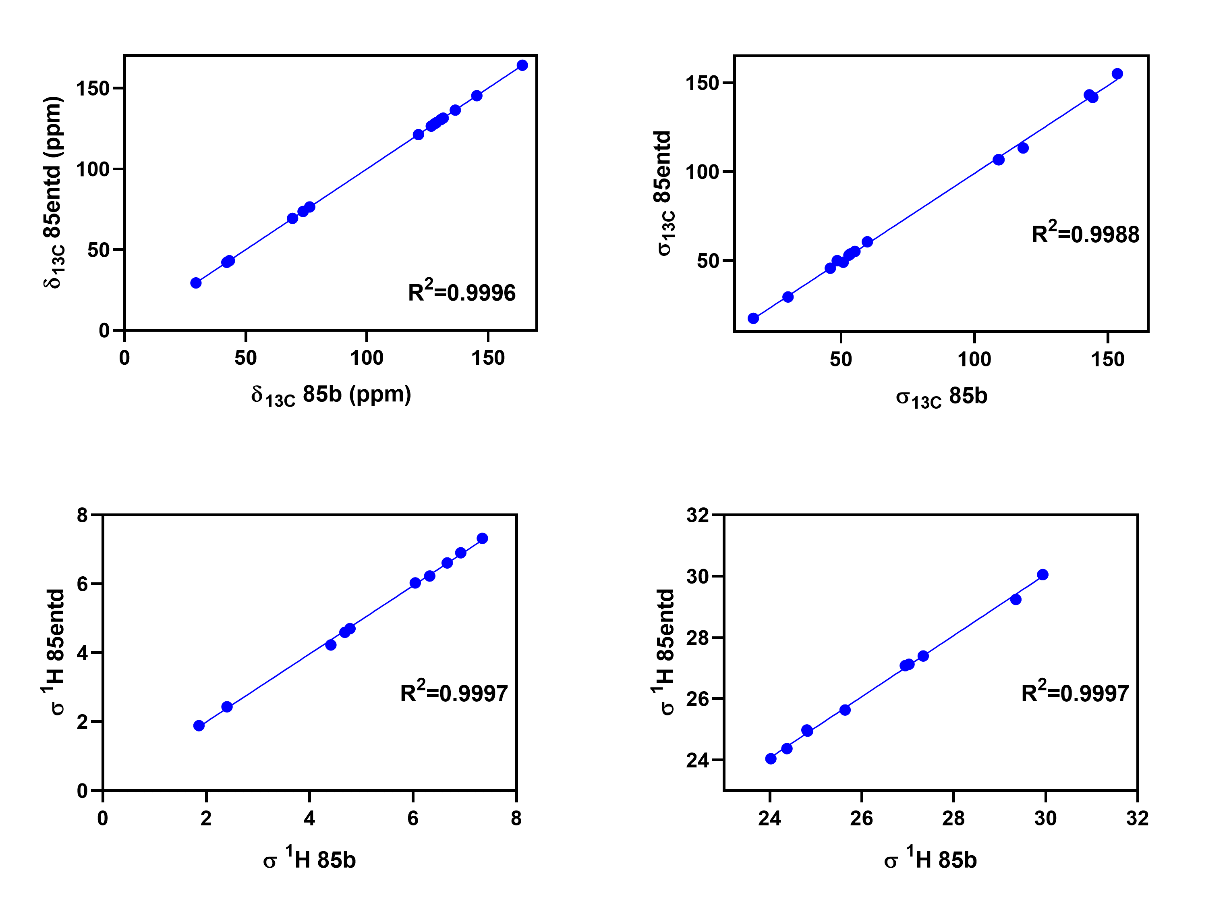


**Figure S 58.** Chemical shift correlation plot between experimental (δ) and DFT calculated (σ) epimers **85b** and **85 *ent* d**. The coefficient of correlation (R²) is shown in both cases.


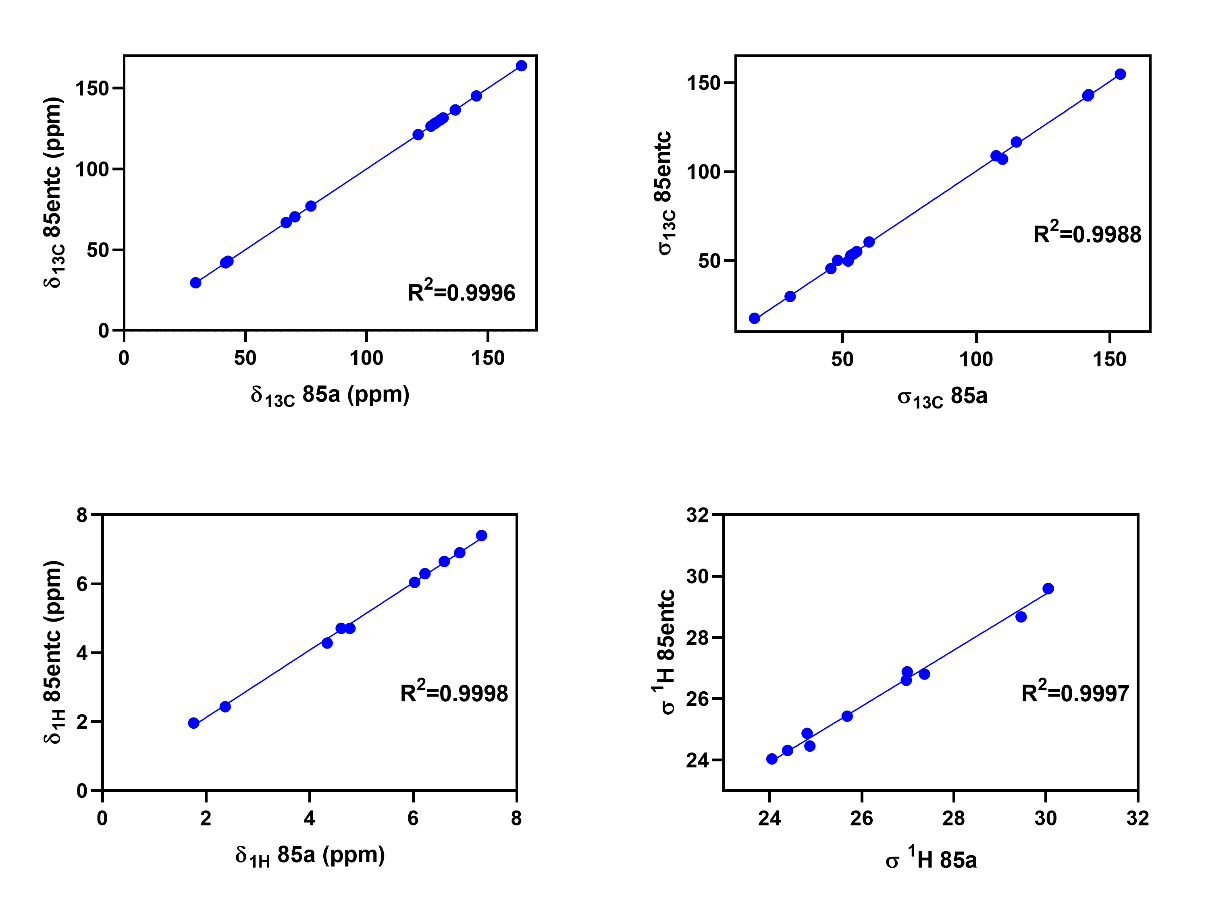


**Figure S 59** Chemical shift correlation plot between experimental (δ) and DFT calculated (σ) epimers **85a** and **85 *ent* c**. The coefficient of correlation (R²) is shown in both cases.


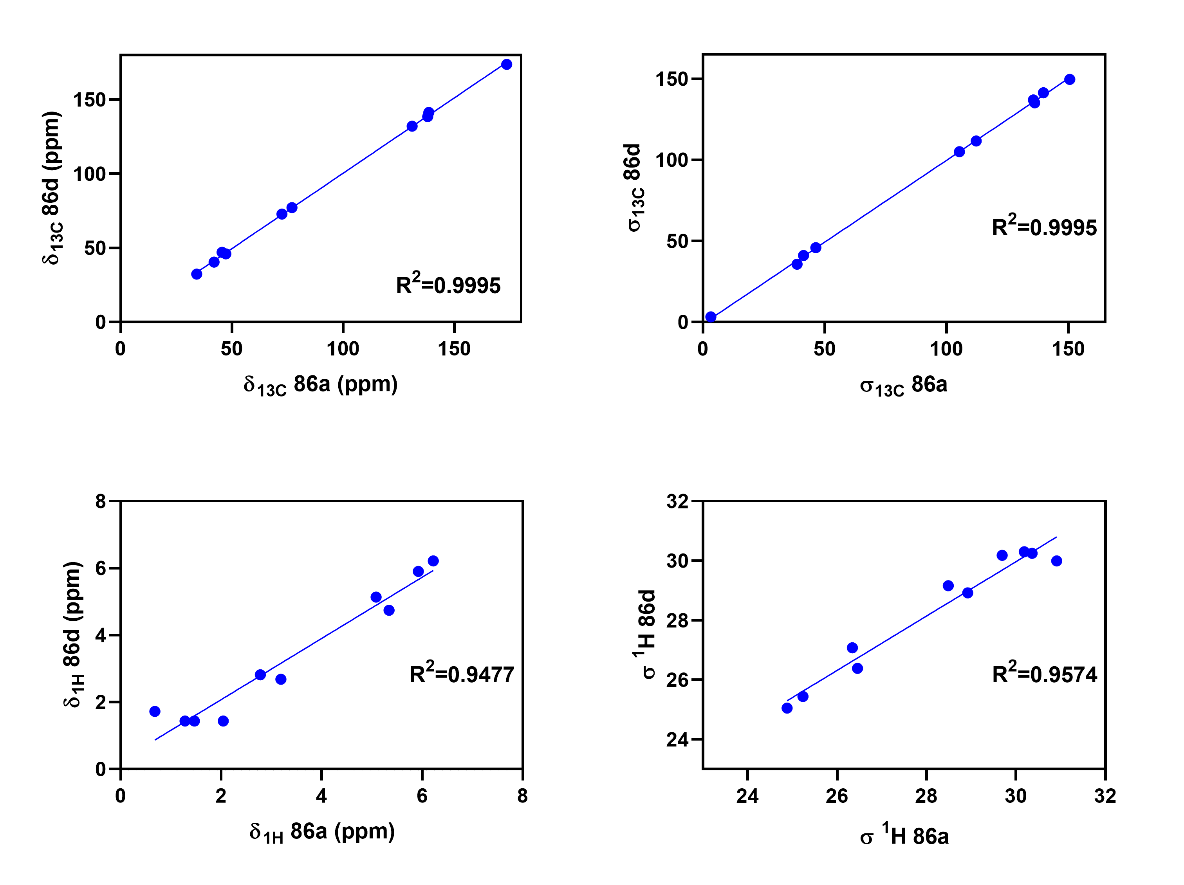


**Figure S60.** Chemical shift correlation plot between experimental (δ) and DFT calculated (σ) epimers **86a** and **86d.** The coefficient of correlation (R²) is shown in both cases.


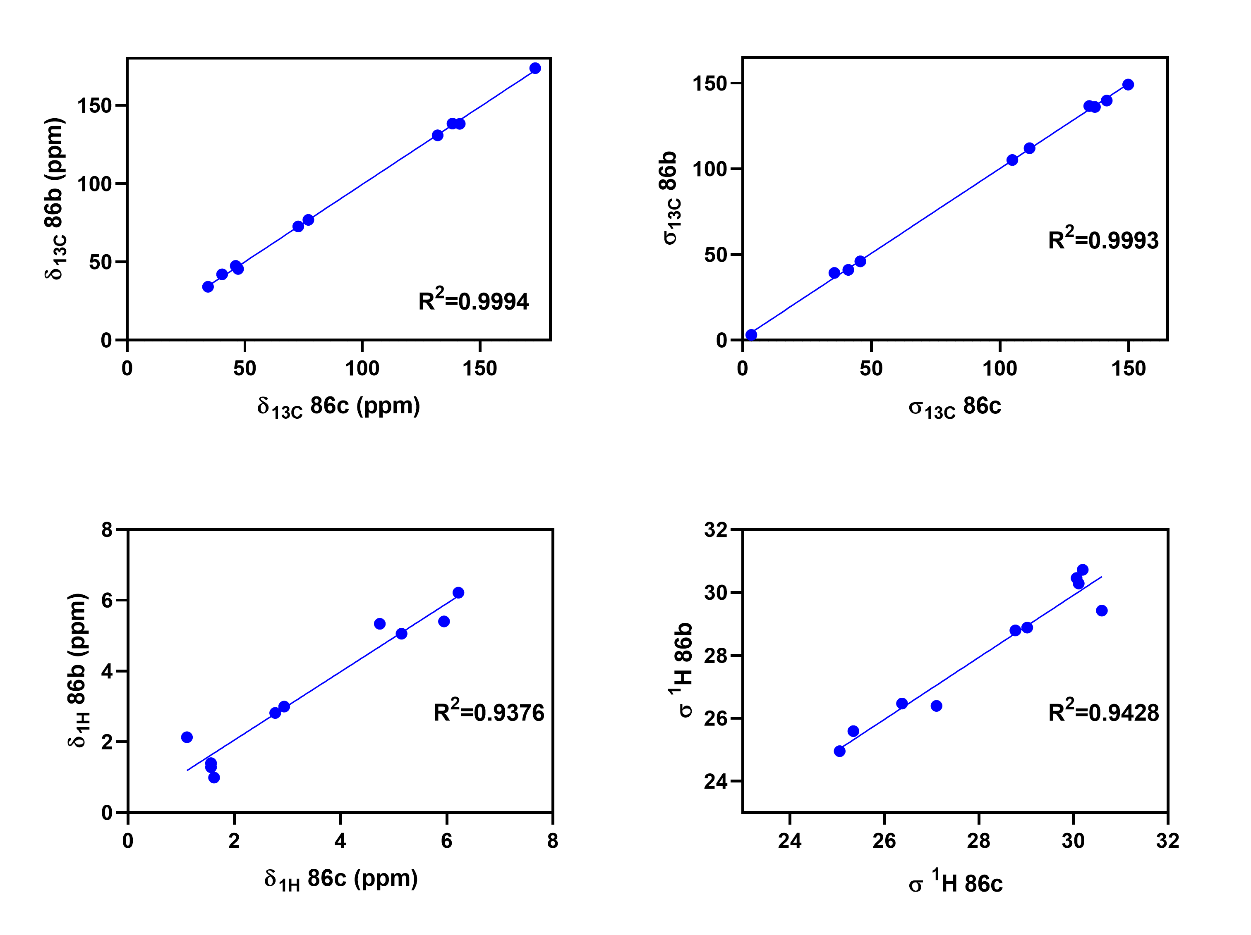


**Figure S61.** Chemical shift correlation plot between experimental (δ) and DFT calculated (σ) epimers **86b** and **86c.** The coefficient of correlation (R²) is shown in both cases.


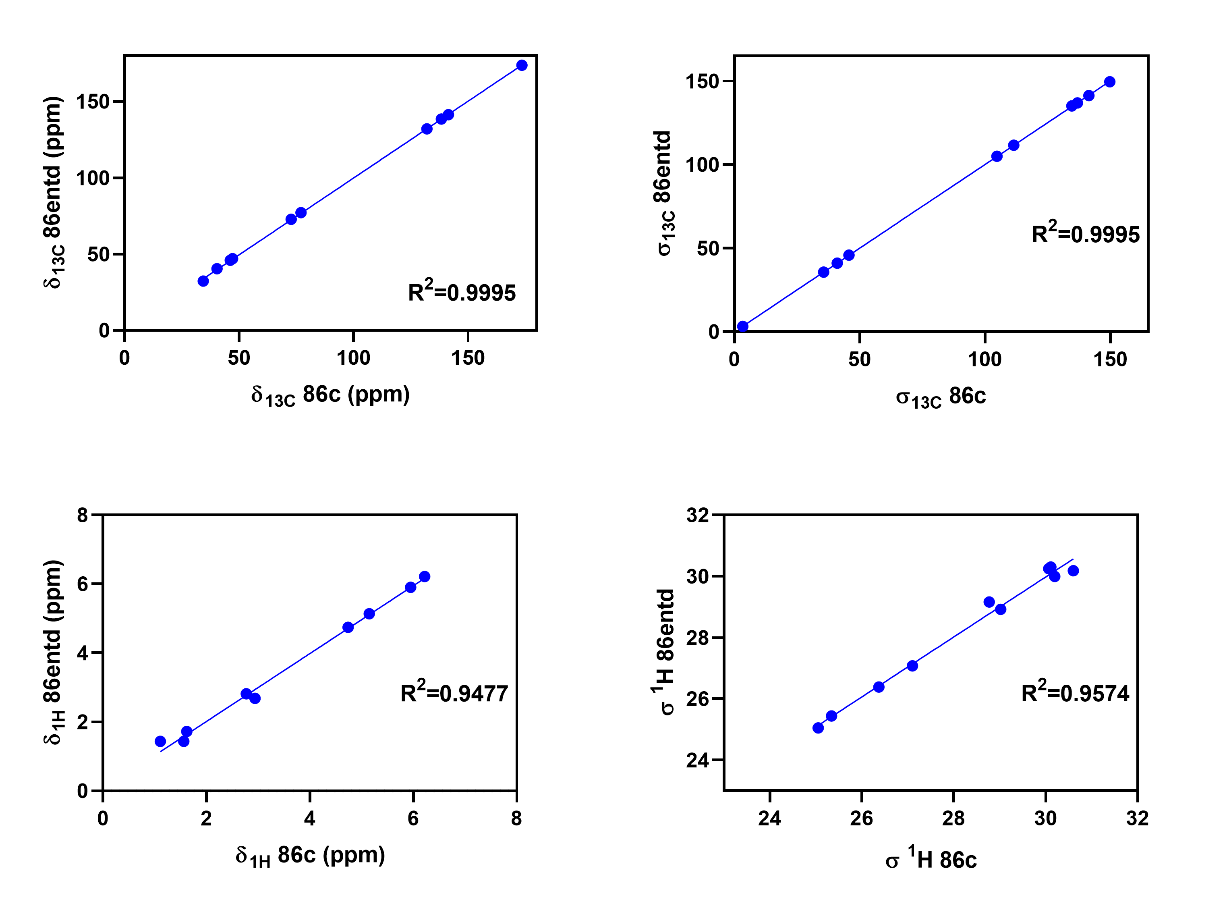


**Figure S 62.** Chemical shift correlation plot between experimental (δ) and DFT calculated (σ) epimers **86c** and **86 *ent* d**. The coefficient of correlation (R²) is shown in both cases.


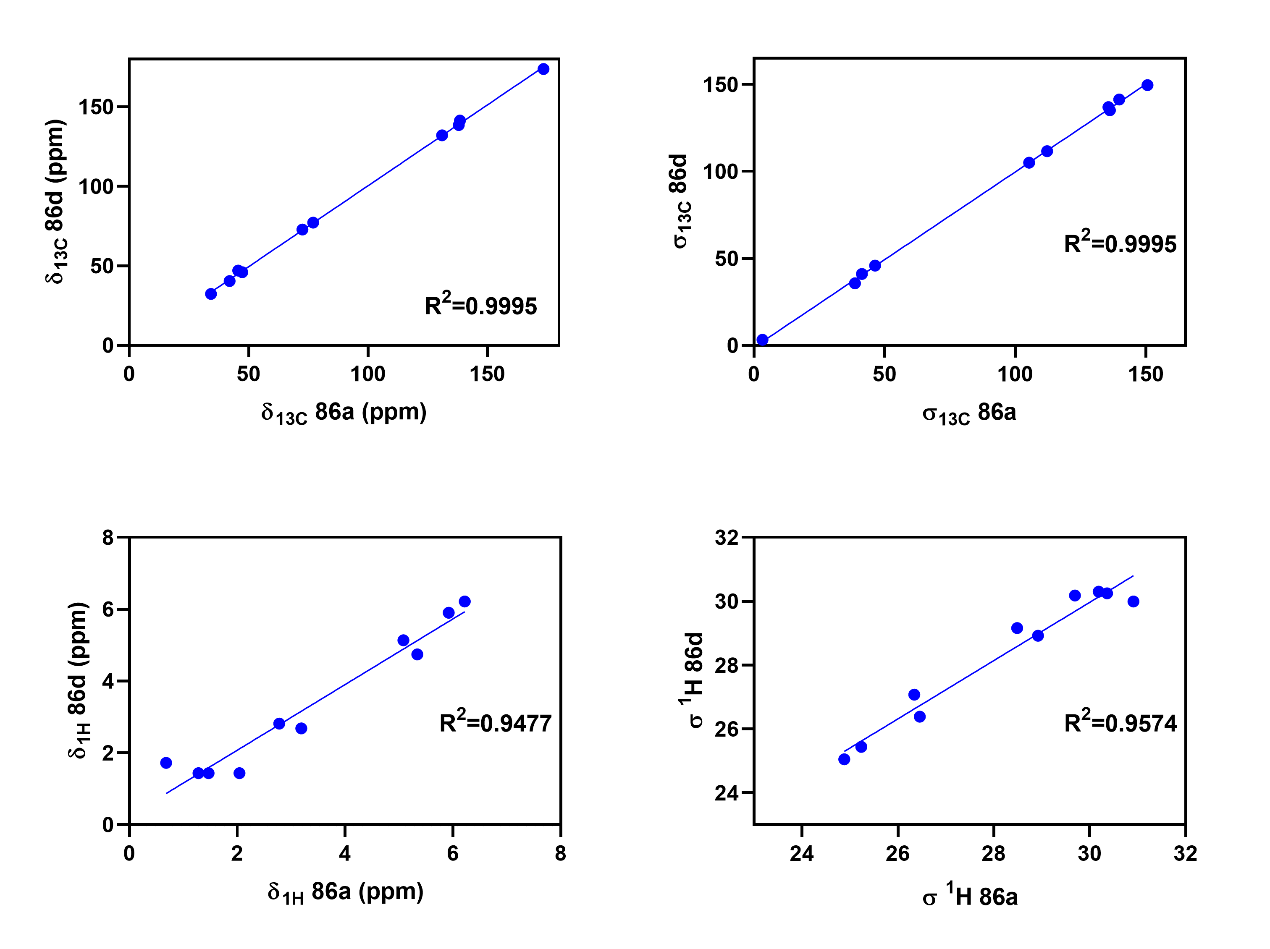


**Figure S63.** Chemical shift correlation plot between experimental (δ) and DFT calculated (σ) epimers **86a** and **86d.** The coefficient of correlation (R²) is shown in both cases.


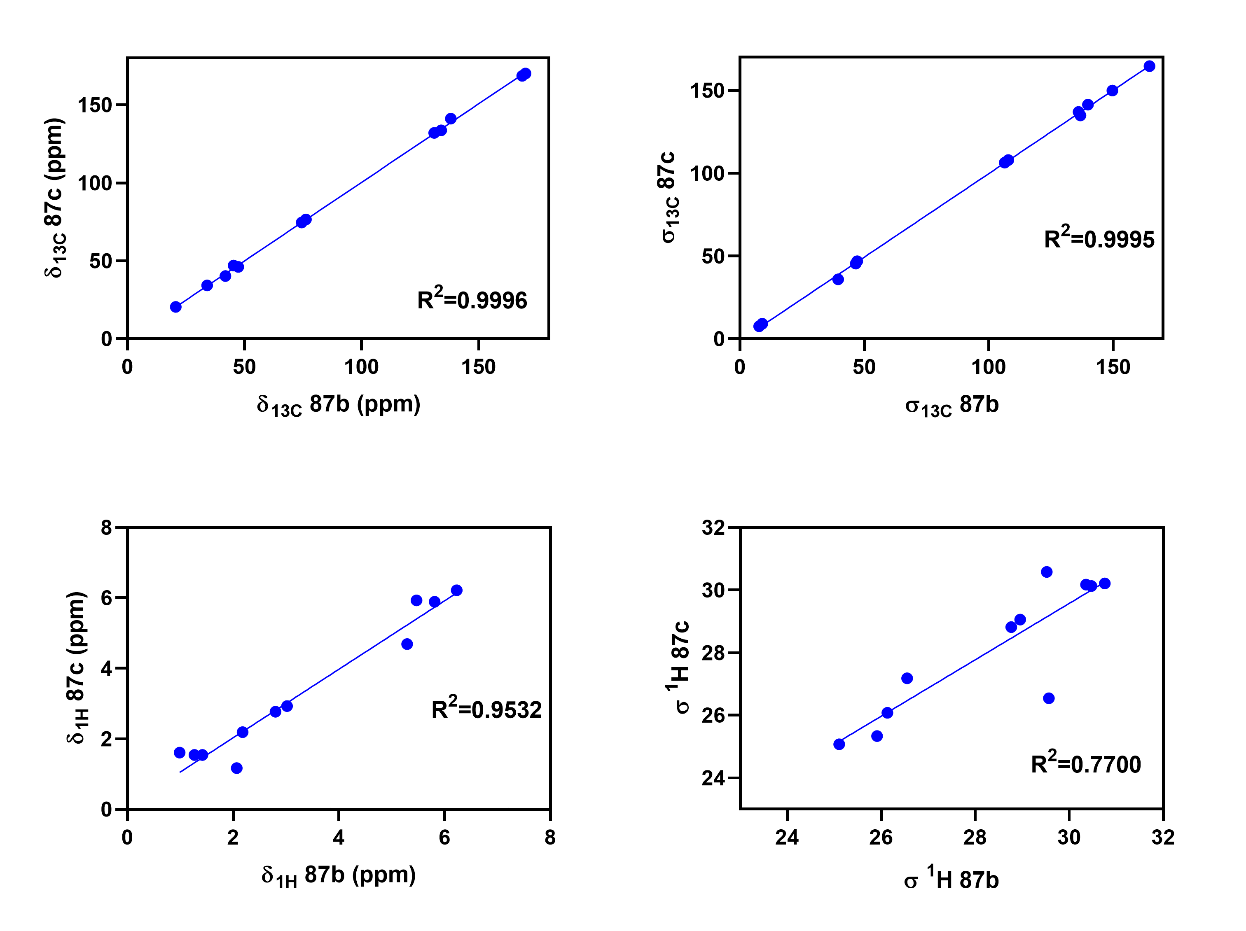


**Figure S64.** Chemical shift correlation plot between experimental (δ) and DFT calculated (σ) epimers **87b** and **87c.** The coefficient of correlation (R²) is shown in both cases.


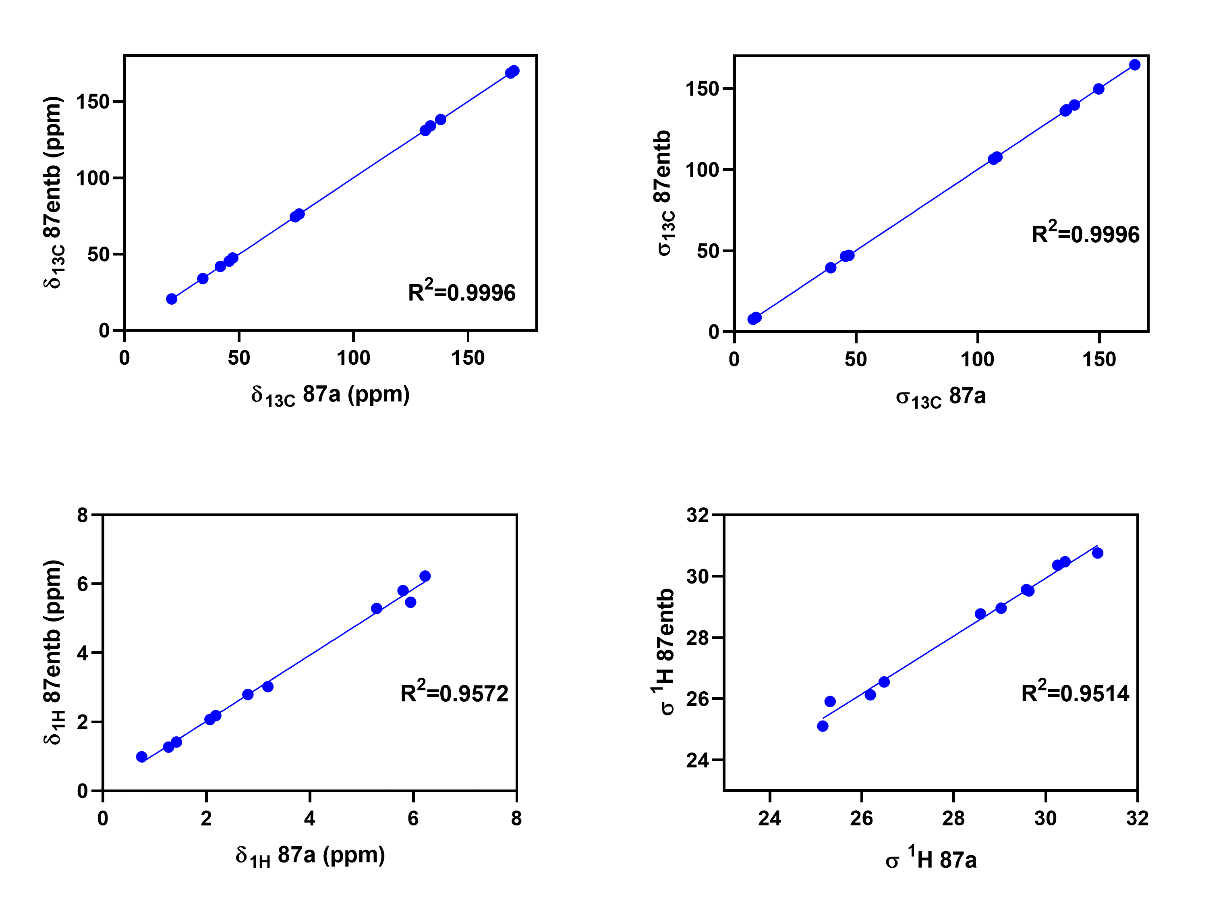


**Figure S 65.** Chemical shift correlation plot between experimental (δ) and DFT calculated (σ) epimers **87a** and **87 *ent* b**. The coefficient of correlation (R²) is shown in both cases.


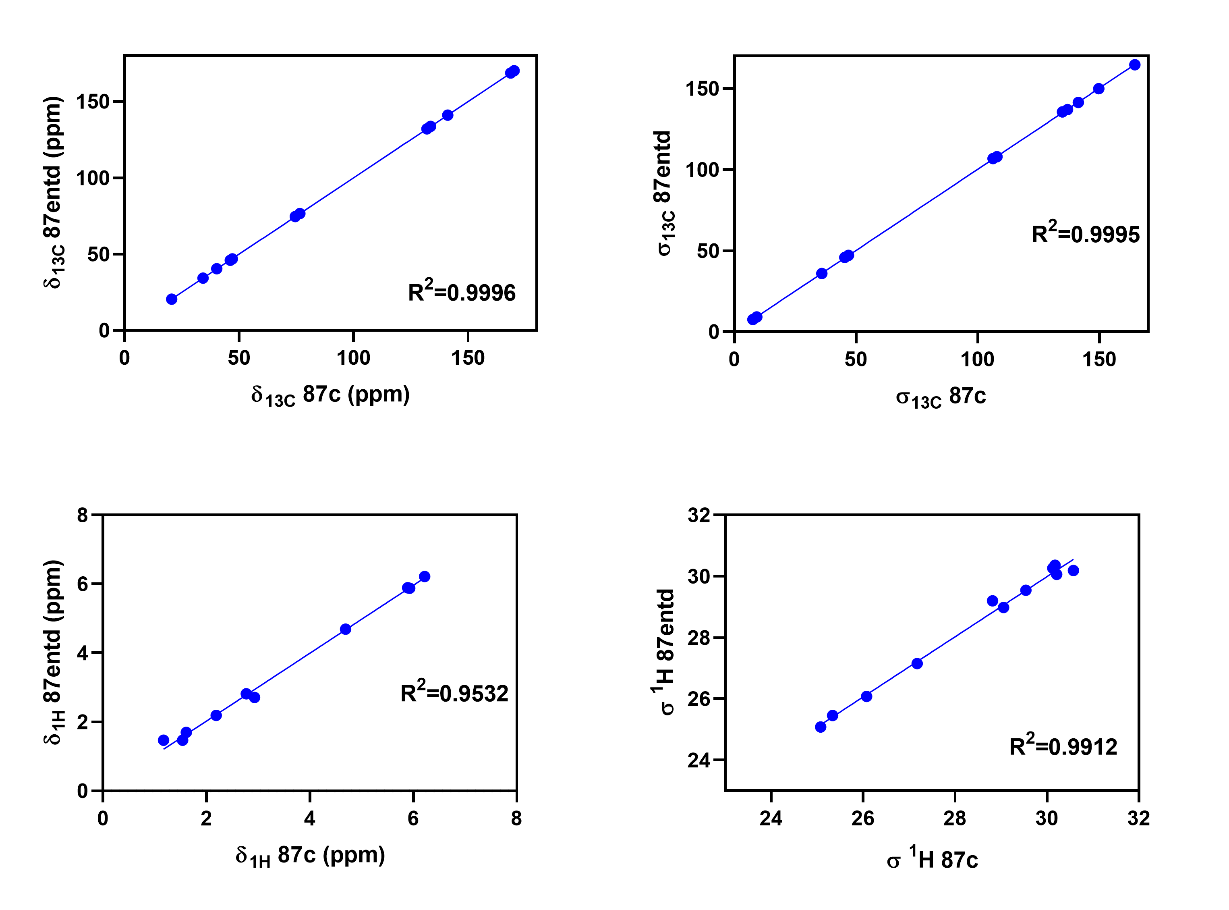


**Figure S66.** Chemical shift correlation plot between experimental (δ) and DFT calculated (σ) epimers **87c** and **87 *ent* d**. The coefficient of correlation (R²) is shown in both cases.

**Assignment of 1b in DMSO:**

**
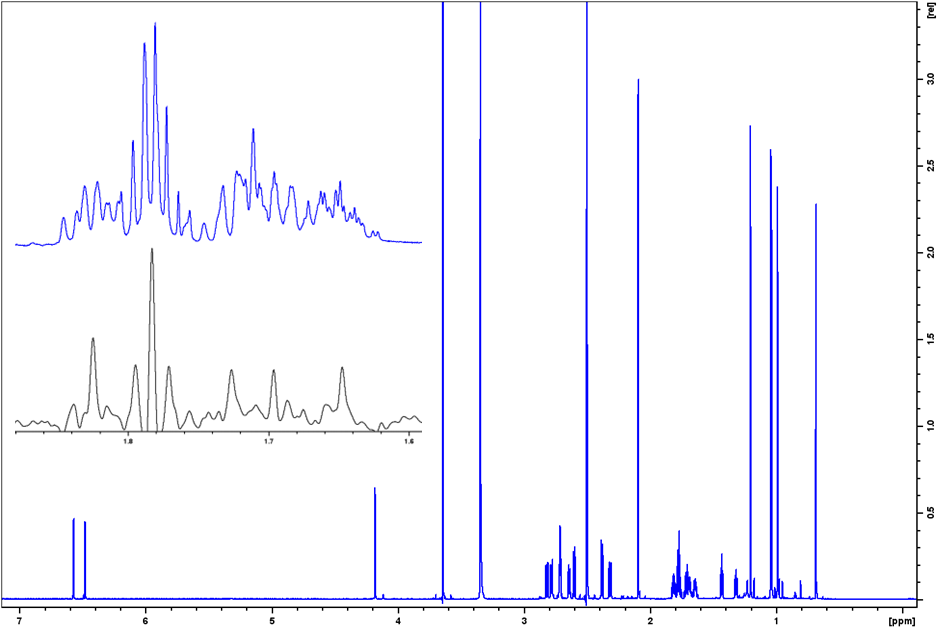
**

H9 (1.64972 ppm)

H9 (1.68737 ppm)

**Figure S67.** ^1^H 1D NMR spectrum of compound **1b** (zg, DMSO-d_6_, NS: 80, 1.2 GHz). The inset (1.60-1.85 ppm) shows the comparison between both spectra: the ^1^H 1D and the PSYCHE pure-shift (800 MHz).


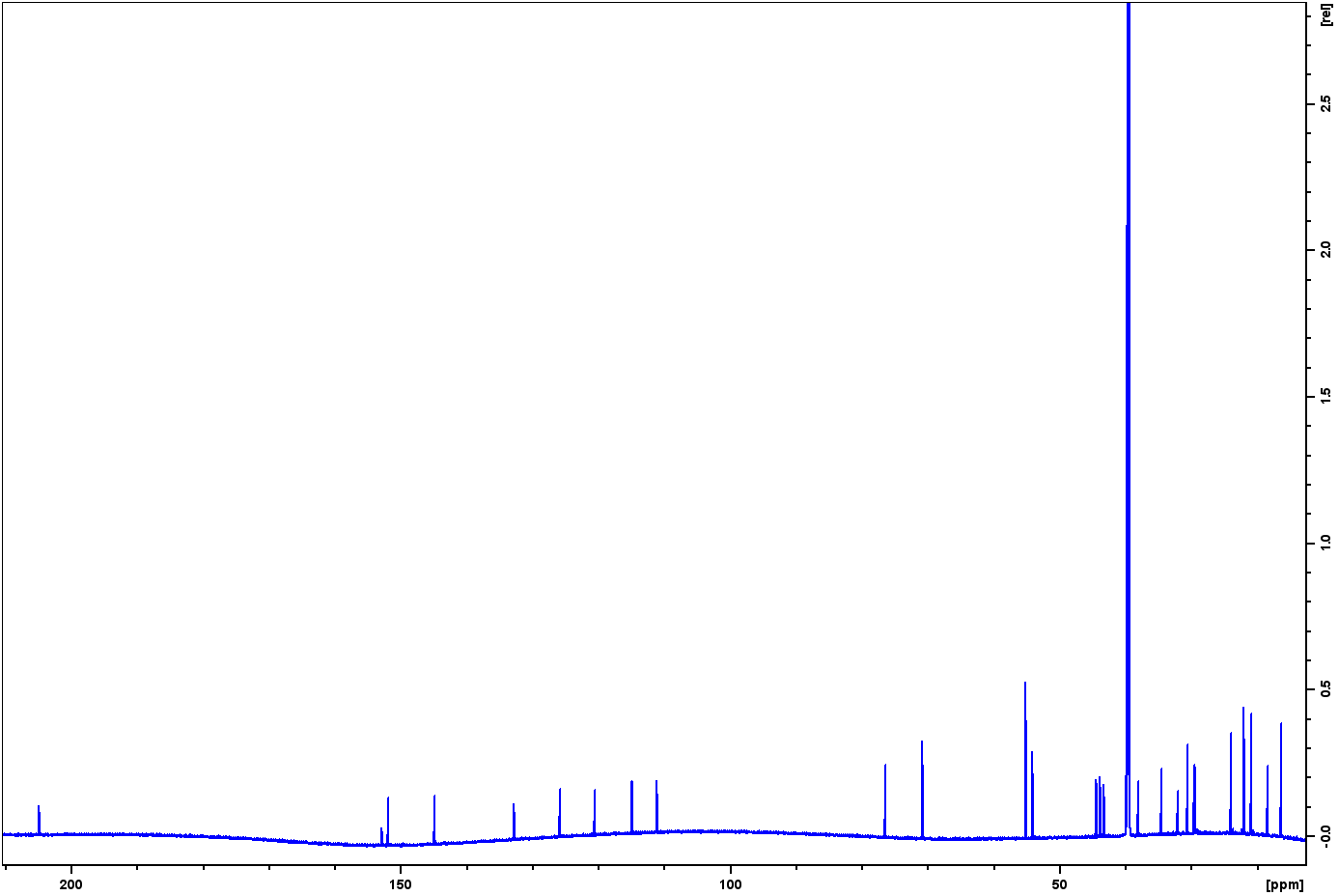


**Figure S68.** ^13^C-{^1^H} NMR spectrum of **1b** (zgpg30, DMSO-d_6_, NS: 8K, 1.2 GHz).


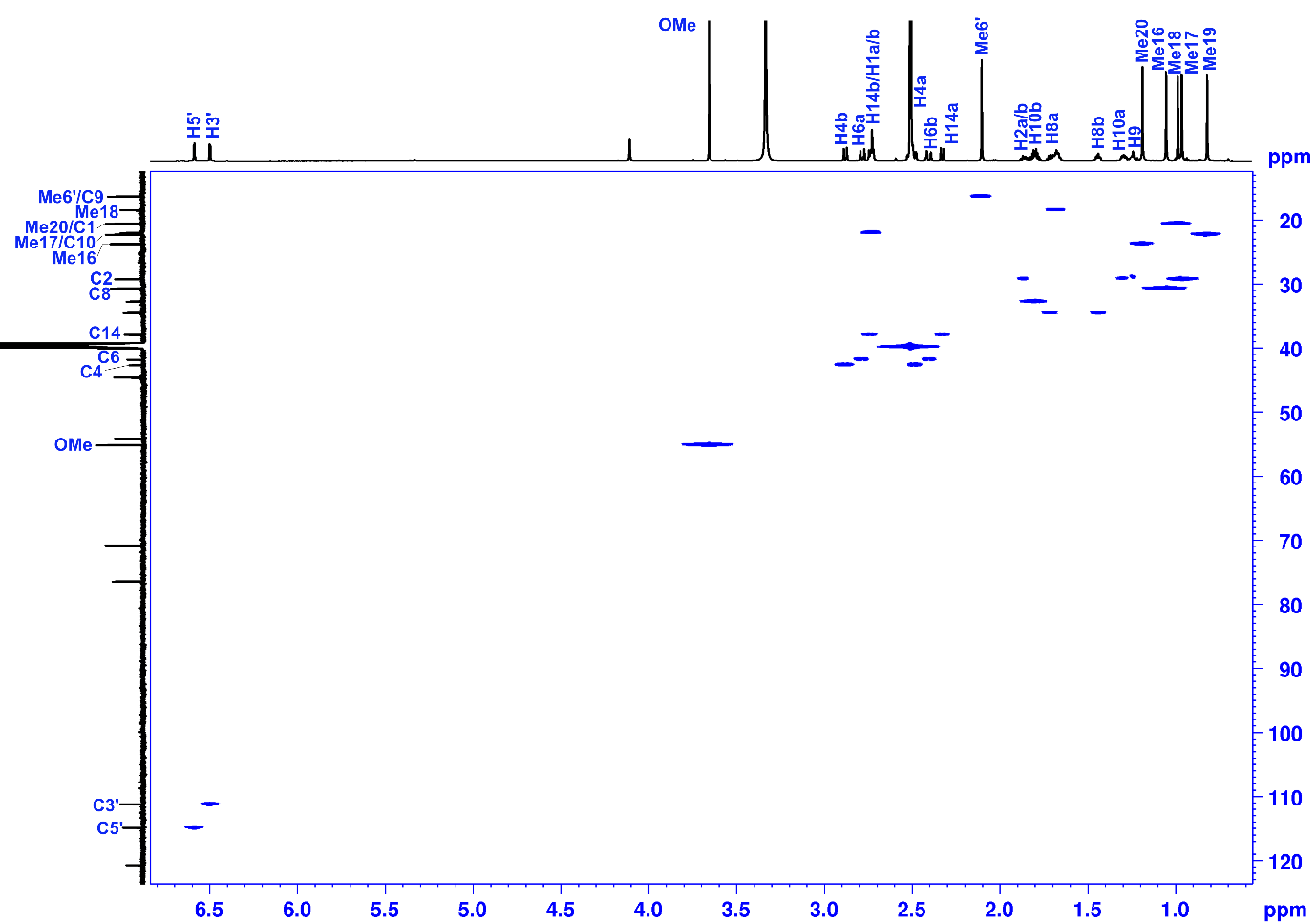


**Figure S69**. ^1^H-^13^C HSQC spectrum of compound **1b** (hsqcetgp, DMSO-d_6_, NS: 16, 800 MHz). Parameters: ^1^J_CH_ =138 Hz. (NUS: 15.6%/40/512). The spectrum on top is a 1D ^1^H spectrum, and the spectrum on the left is a carbon-proton decoupled spectrum.

**
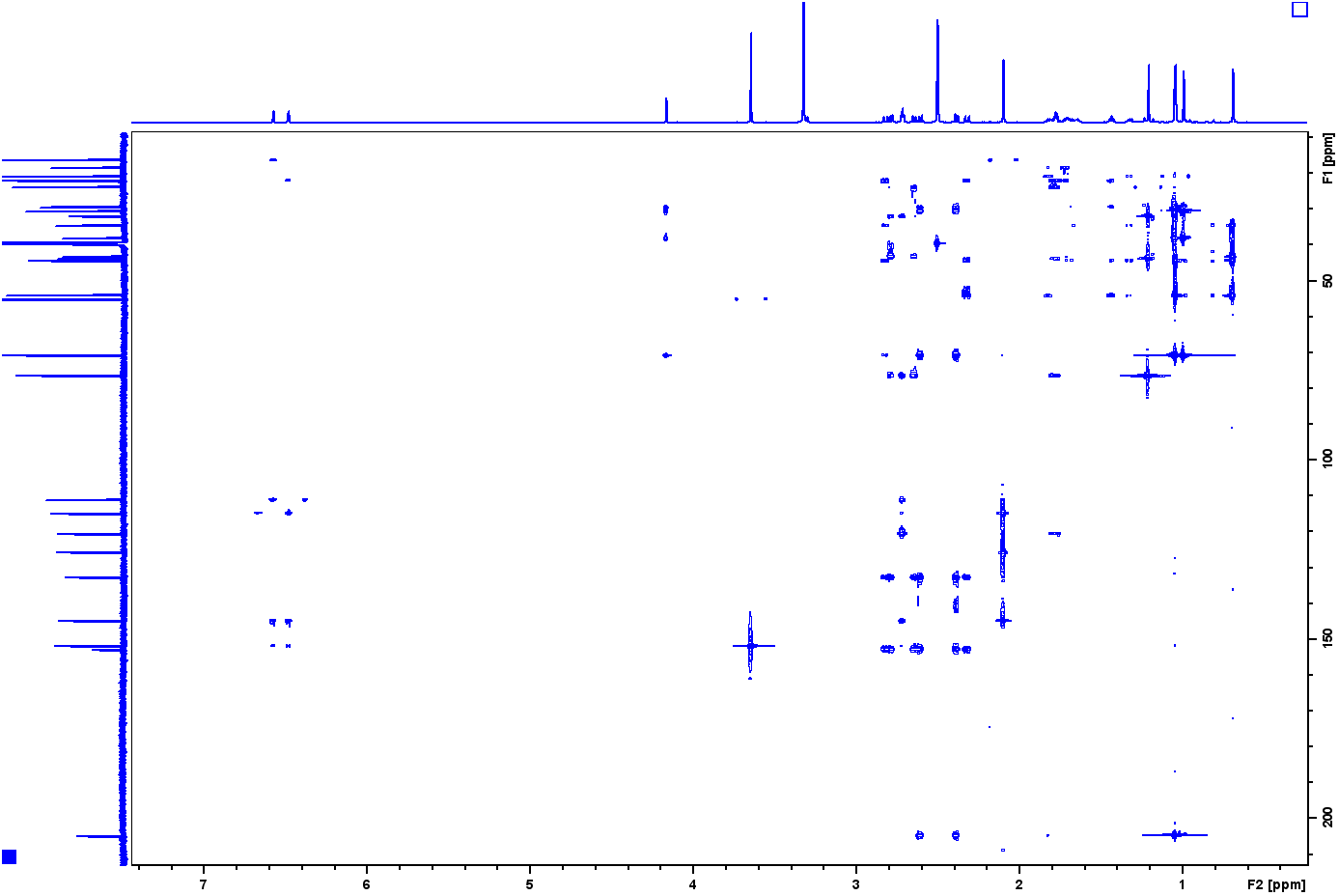
**

**Figure S70.** ^1^H-^13^C HMBC spectrum of compound **1b** (hmbcetgpl3nd, DMSO, NS: 24, 800 MHz). Parameters: ^1^J_CH_ (Min) = 120 Hz; ^1^J_CH_ (Max) = 168 Hz; ^n^J_CH_ (Min) = 8 Hz; (NUS: 25%/64/512). The spectrum on top is a 1D ^1^H spectrum, and the spectrum on the left is a carbon-proton decoupled spectrum.


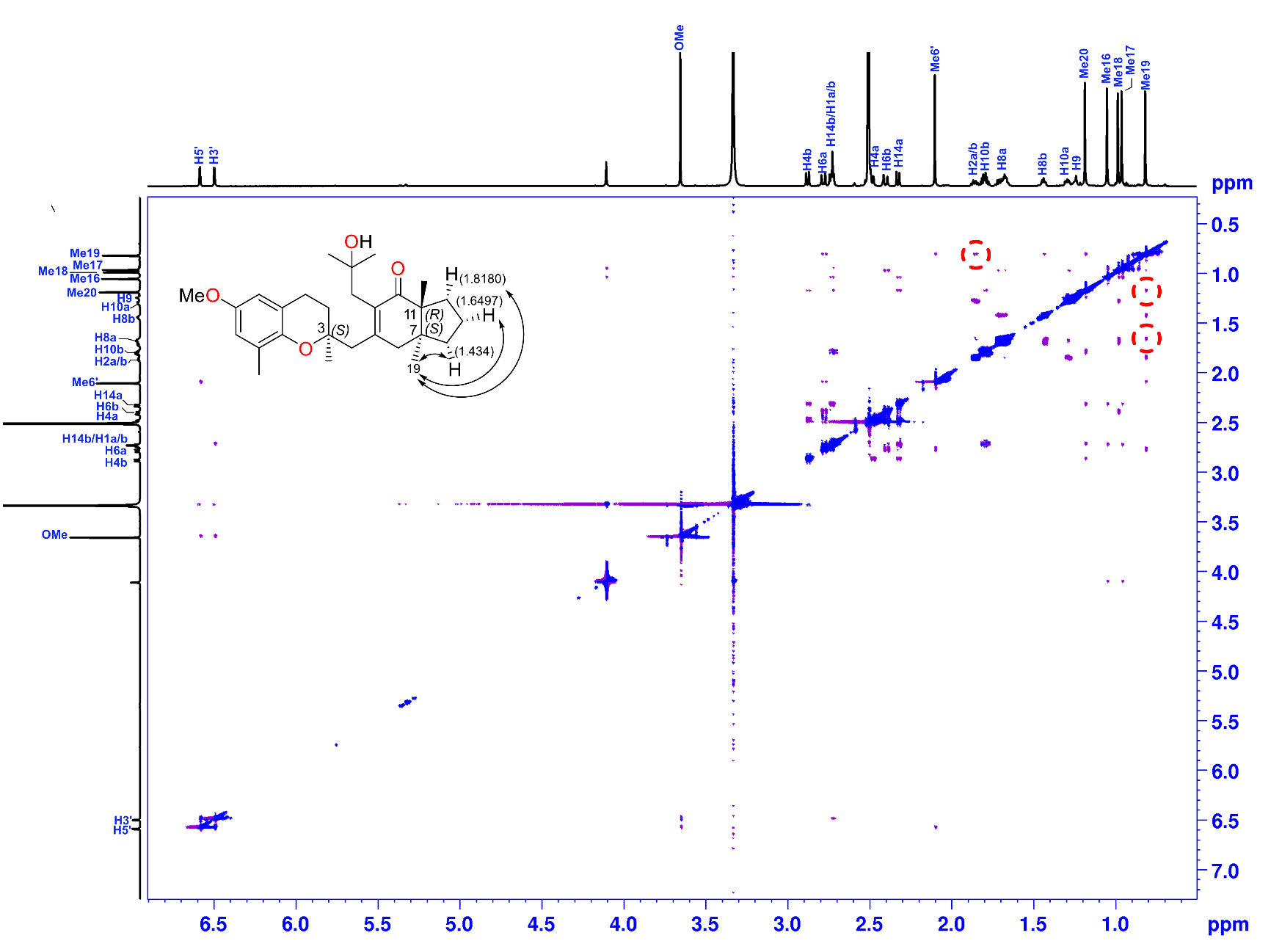


**Figure S71.** 2D ROESY spectrum of compound **1a** (roesyphpp.2, DMSO-d_6_, NS: 80; mixing time: 200 ms; 1.2 GHz). The inset shows the assignment of the relative orientation of protons H8a, H9a, and H10a to Me19, thanks to ROE contacts. The spectrum on top and on the left is a 1D ^1^H spectrum,

**A protocol for determining the conformational landscape**

**1^rst^: Generating the conformational pool:** An extensive systematic conformational search (CS) covering all relevant rotatable bonds and torsional bonds was initially conducted using Maestro-Schrödinger^[[1]](#footnote-1)^.^[1,2]^ This was followed by a DFT geometry optimization at the M06-2X^[3]^/6-31+G(d,p)/IEFPCM=DMSO level for all the conformers found (conformational pool). Subsequently, a Single Point Energy (SPE) calculation was performed to provide a more precise description of the relative populations of the geometries at the mPW1PW91^[4]^/6-311+G(2d,p)/IEFPCM=DMSO level.

**2^nd^: Cleaning the conformational pool:** The root mean square deviation (RMSD) of all geometries in the conformational pool was calculated using Kabsch's algorithm to achieve the necessary conformer alignment. The first step of conformer filtering, based on RMSD and DFT-SPE energy similarities, involved recursively comparing all conformers in pairs; when a pair of conformers had the same RMSD, the one with the higher DFT-based relative energy was excluded. For the second filtering step, geometries with non-significant variations in bond torsion and bond rotations were ignored, while those experimentally known were preserved. For example, this approach preserved the rotation around C14 and the helicities in the chromane moiety. At this stage, the conformational pool is refined. It can be visualized through RMSD heatmaps and hierarchical dendrogram groupings (Figure **S54**-**S57**).**3^rd^: Defining the conformational space**: Descriptive statistics tools, including histograms and correlation matrices, were used to portray the geometric variability of the remaining geometries. All experimentally identified rotatable bonds and ring torsions were considered, along with one molecular descriptor. The relevant torsions and rotations used to capture the structural variability of the conformational landscape of 1a and 1b were: H4-C4-C3-C20, H4-C4-C5-C13, H14-C14-C13-C5, H14-C14-C15-C16, and H2-C2-C1-C2’, one geometric descriptor (Gyration Ratio) (Figure S58-S61). It is worth noting that for other molecular geometries, different molecular and torsional descriptors may be more suitable to depict inherent conformational differences. We encourage the reader to explore specialized literature on this topic (e.g., Handbook of Molecular Descriptors. DOI:10.1002/9783527613106) and apply them strategically. Their redundancy can be detected by inspecting the correlation matrices as the one shown in Figures S60–S61. For example, the redundancy between the dihedrals H14-C14-C15-C16 and H14-C14-C15-C17 can be easily identified in the conformational space study of SSR, as their Pearson correlation coefficient (PCC) is 1.00 (Figure S60). Meanwhile, a less evident correlation between dihedrals H14-C14-C13-C5 and H4-C4-C5-C14 is observed (PCC = 0.89), which can be attributed to steric hindrance within the molecular geometry. This finding highlights the importance of statistical tools for the study of conformational spaces.

**4^th^: Appearance of the conformational landscape:** Finally, conformers within a 3.5 kcal/mol DFT-SPE energy window were selected to represent the conformational landscape in a two-dimensional manner using PCA. Clustering based on structural similarities (*vide supra*) was achieved through the silhouette method^[5]^ (Figure S83 and Figure 6 in the manuscript).


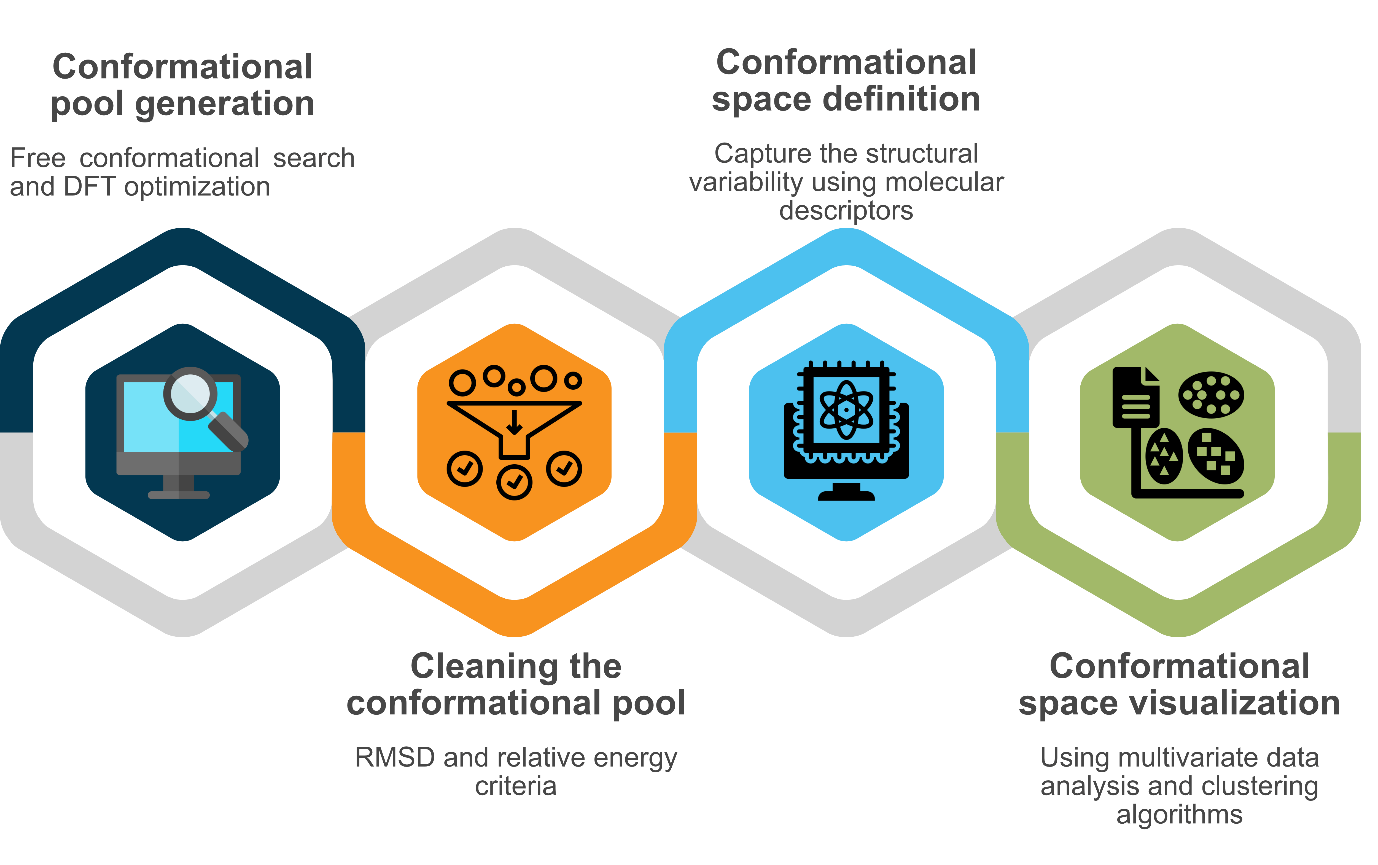


**Figure S72.** Workflow to determine the conformational landscape.


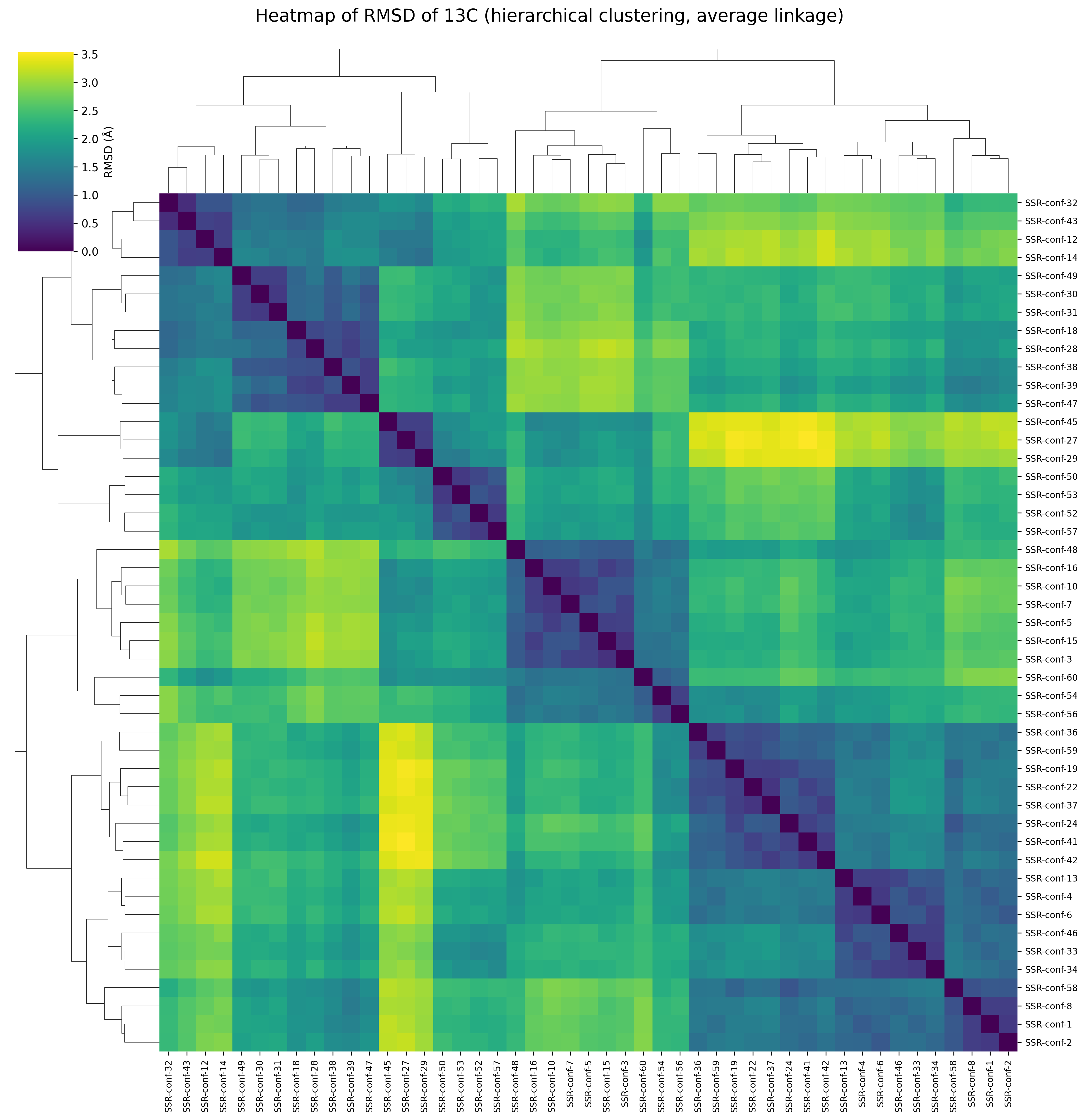


**Figure S73.** Heatmap of the RMSD analysis of the DFT-optimized set of the SSR-**1a** conformational pool. The color scale indicates the RMSD values (Å). A hierarchical dendrogram is shown at the top and on the left, where grouping was achieved using the Unweighted Pair Group Method with Arithmetic Mean. Conformers labeled as redundant are colored blue (Approx. RMSD < 0.5 Å).


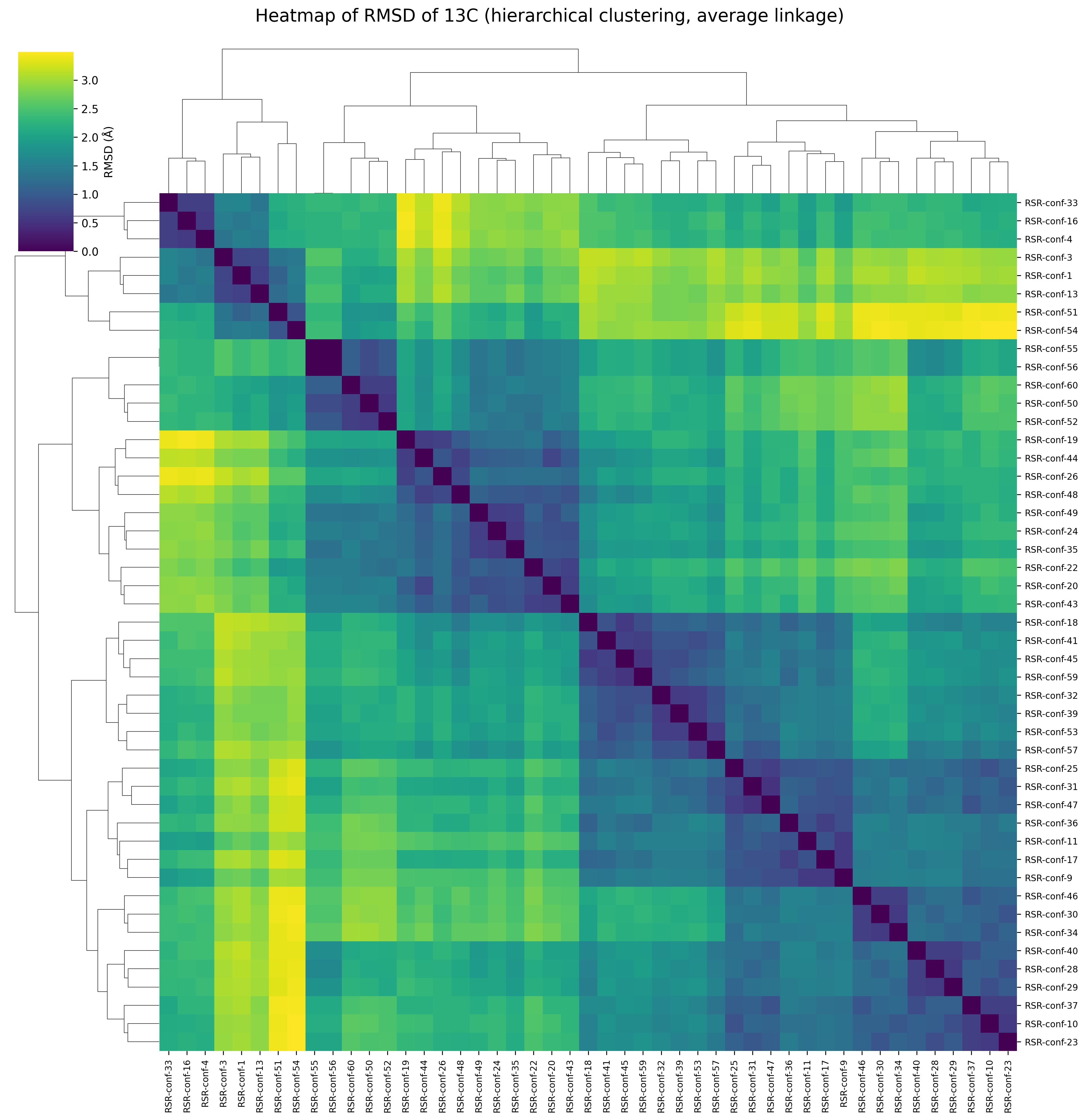


**Figure S74.** Heatmap of the RMSD analysis of the DFT-optimized set of the RSR-**1b** conformational pool. The color scale indicates the RMSD values (Å). A hierarchical dendrogram is shown at the top and on the left, where grouping was achieved using the Unweighted Pair Group Method with Arithmetic Mean. Conformers labeled as redundant are colored blue (Approx. RMSD < 0.5 Å).


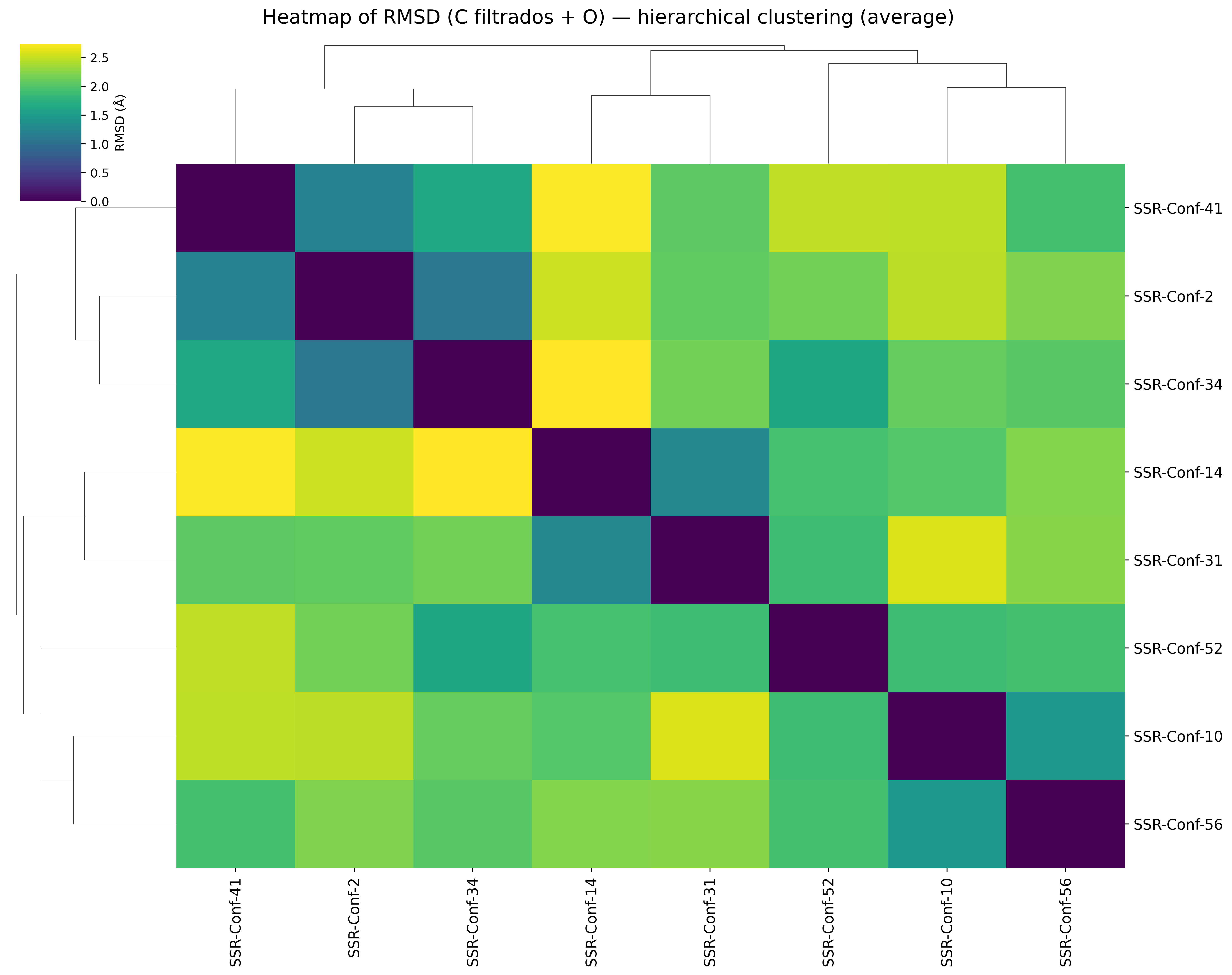


**Figure S75.** Heatmap of the RMSD analysis of the DFT-optimized set of the SSR-**1a** conformational space. The color scale indicates the RMSD values (Å). A hierarchical dendrogram is shown at the top and on the left, where grouping was achieved using the Unweighted Pair Group Method with Arithmetic Mean.


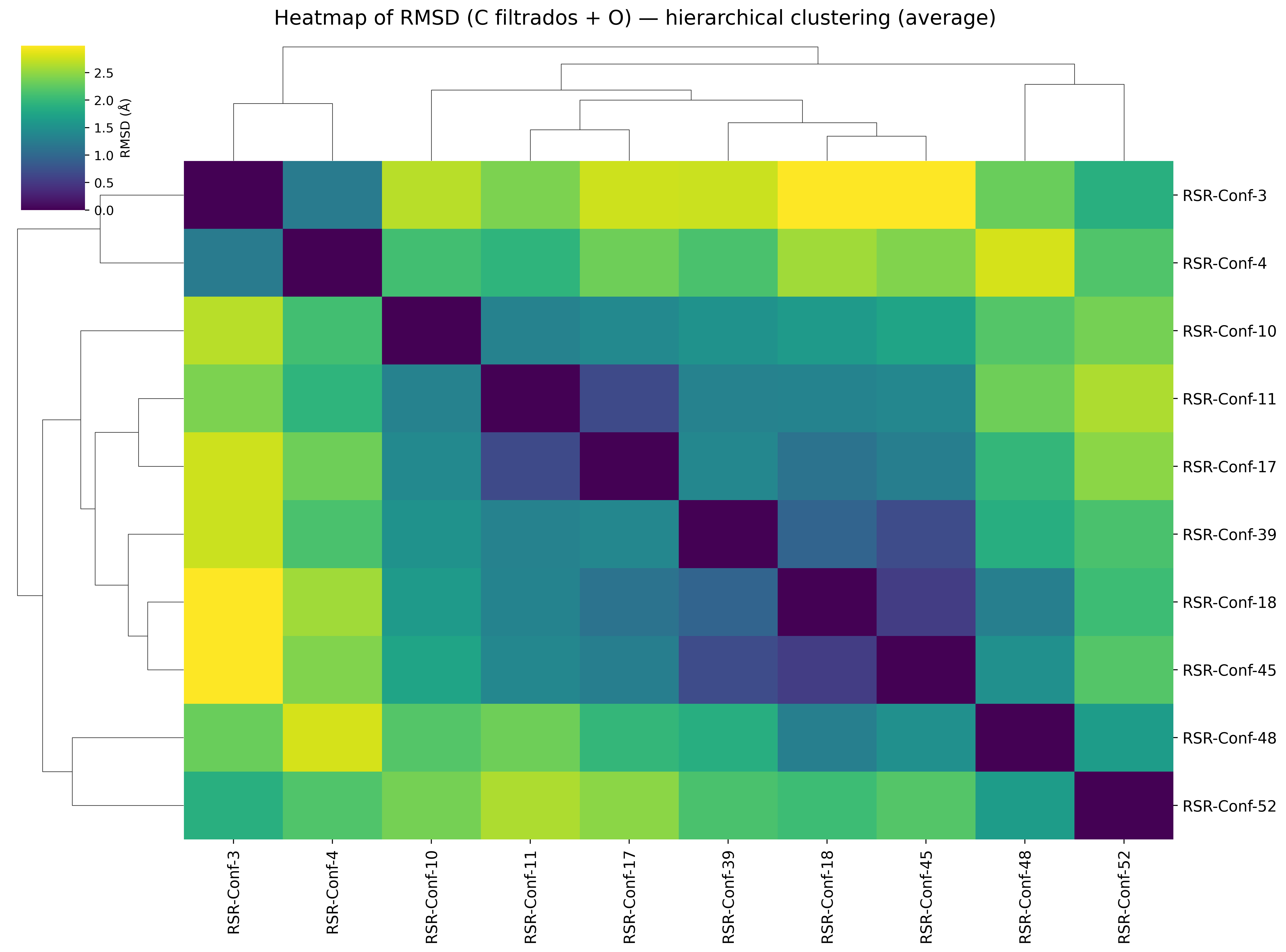


**Figure S76.** Heatmap of the RMSD analysis of the DFT-optimized set of the RSR-**1b** conformational space. The color scale indicates the RMSD values (Å). A hierarchical dendrogram is shown at the top and on the left, where grouping was achieved using the Unweighted Pair Group Method with Arithmetic Mean.


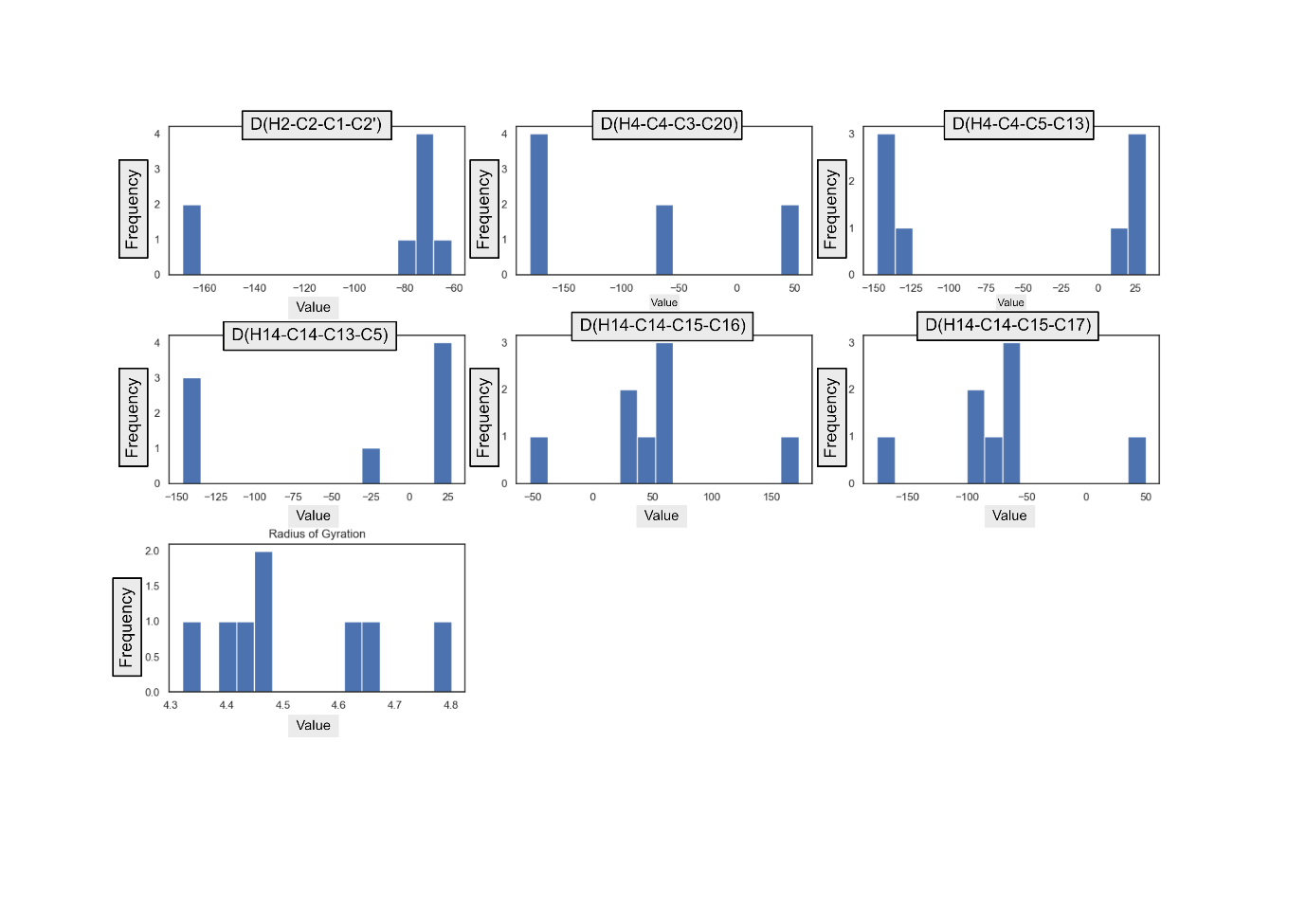


**Figure S77.** Histograms showing the dihedral angle distribution among the representative conformers of SSR-**1a**. After inspection of D(H14-C14-C15-C16) and D(H14-C14-C15-C17), its redundancy becomes evident.


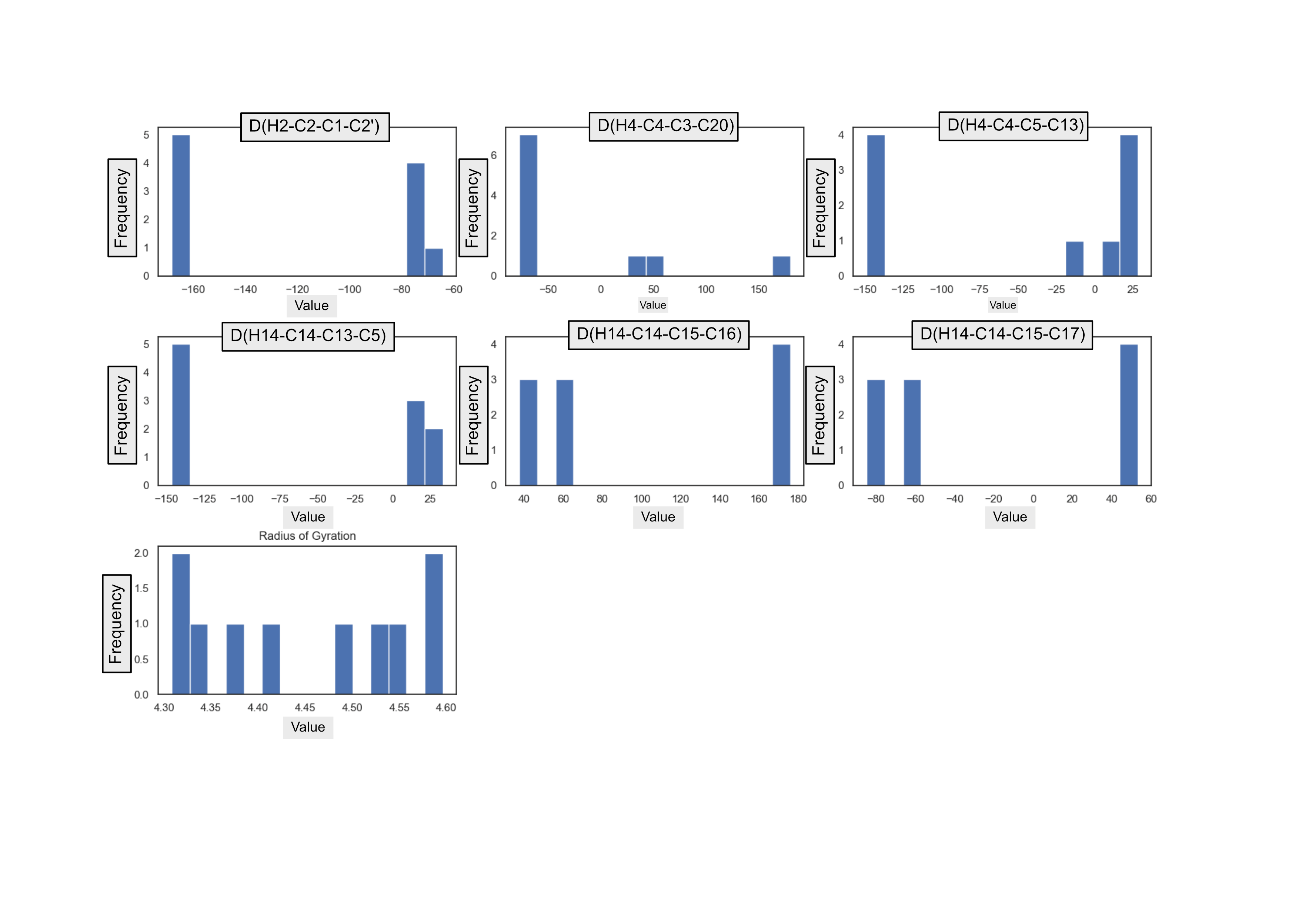


**Figure S78.** Histograms showing the dihedral angle distribution among the representative conformers of *RSR-***1b**. After inspection of D(41-11-18-19) and D(41-11-18-21), its redundancy becomes evident.


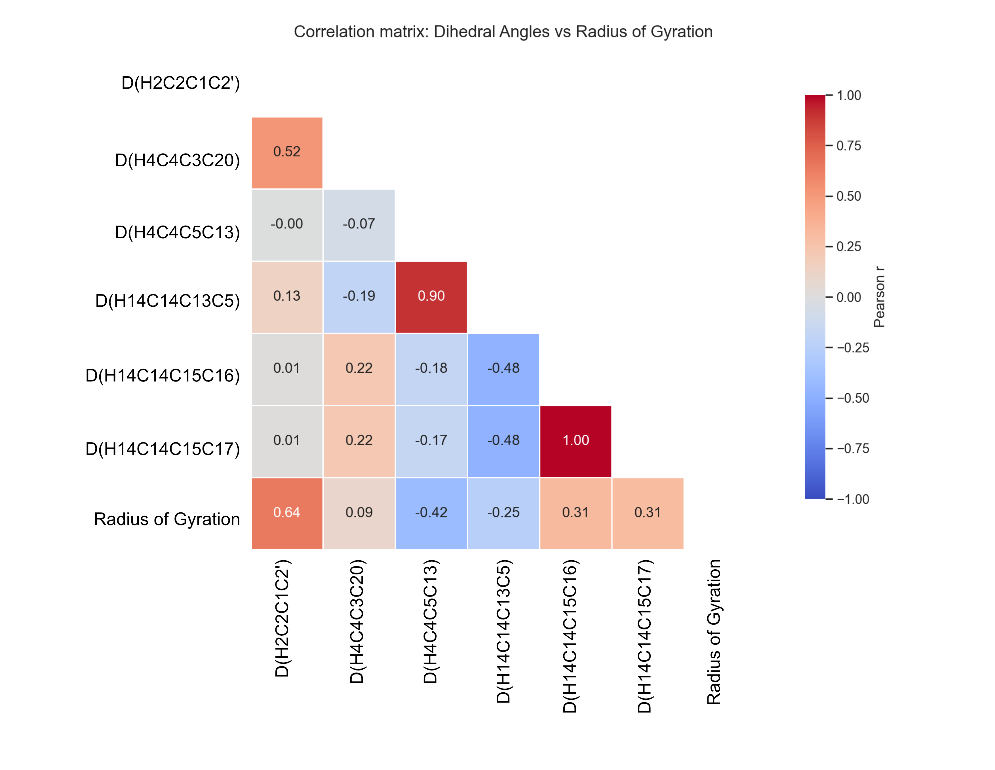


**Figure S79.** Correlation matrix among molecular and torsional descriptors of *SSR*-**1a** conformers. The level of correlation is expressed as the Pearson correlation coefficient. The correlation between dihedrals becomes evident upon visual inspection of the matrix. Dihedrals H14-C14-C15-C16 and H14-C14-C15-C17 are perfectly correlated (PCC = 1.00), meaning that they redundantly contribute to the description of the molecular landscape. Interestingly, dihedrals H14-C14-C13-C5 and H4-C4-C5-C14 appear to be highly correlated (PCC = 0.90), which can be attributed to steric hindrance within the molecular geometry.


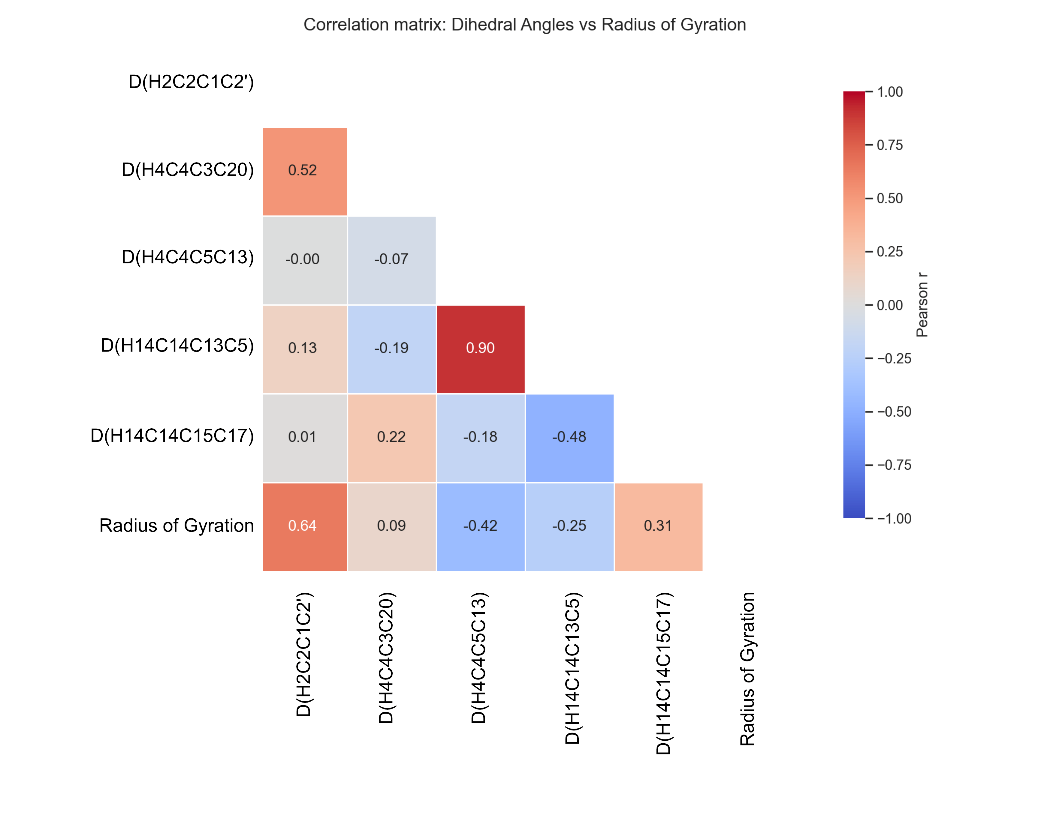


**Figure S80.** Correlation matrix among molecular and geometrical descriptors of conformers of *SSR*-**1a**, expressed in Pearson correlation coefficient (PCC), after dihedral H14-C14-C15-C17 removal.


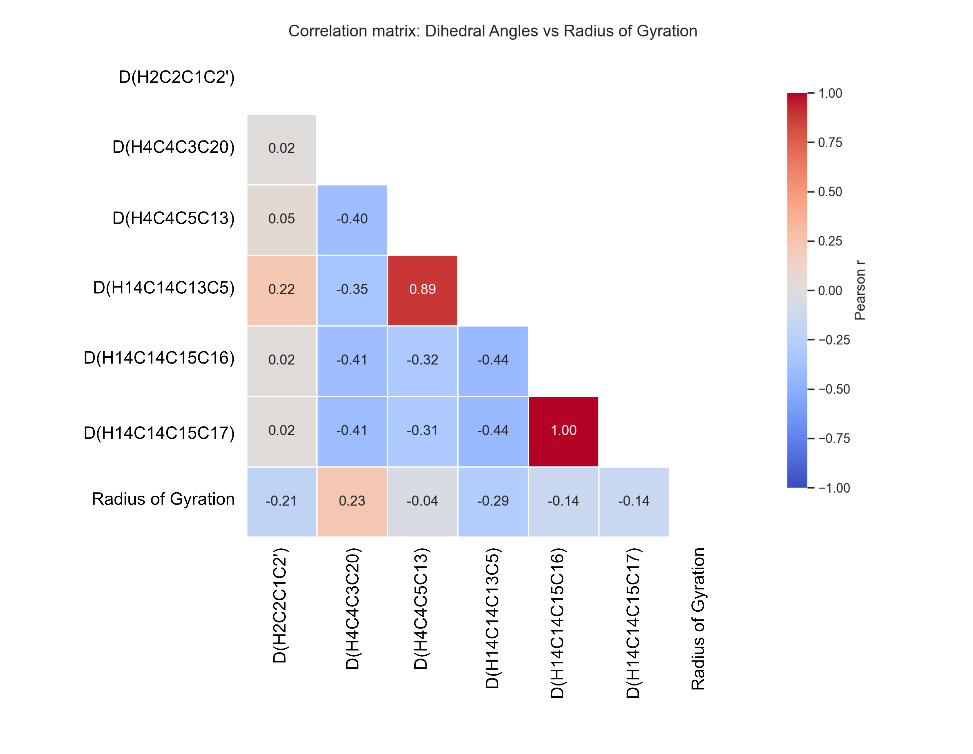


**Figure S81.** Correlation matrix among molecular and torsional descriptors of RSR-**1b** conformers. The level of correlation is expressed as the Pearson correlation coefficient (PCC). The correlation between dihedrals becomes evident upon visual inspection of the matrix. Dihedrals H14-C14-C15-C16 and H14-C14-C15-C17 are perfectly correlated (PCC = 1.00), meaning that they redundantly contribute to the description of the molecular landscape. Interestingly, dihedrals H14-C14-C13-C5 and H4-C4-C5-C14 appear to be highly correlated (PCC = 0.89), which can be attributed to steric hindrance within the molecular geometry.


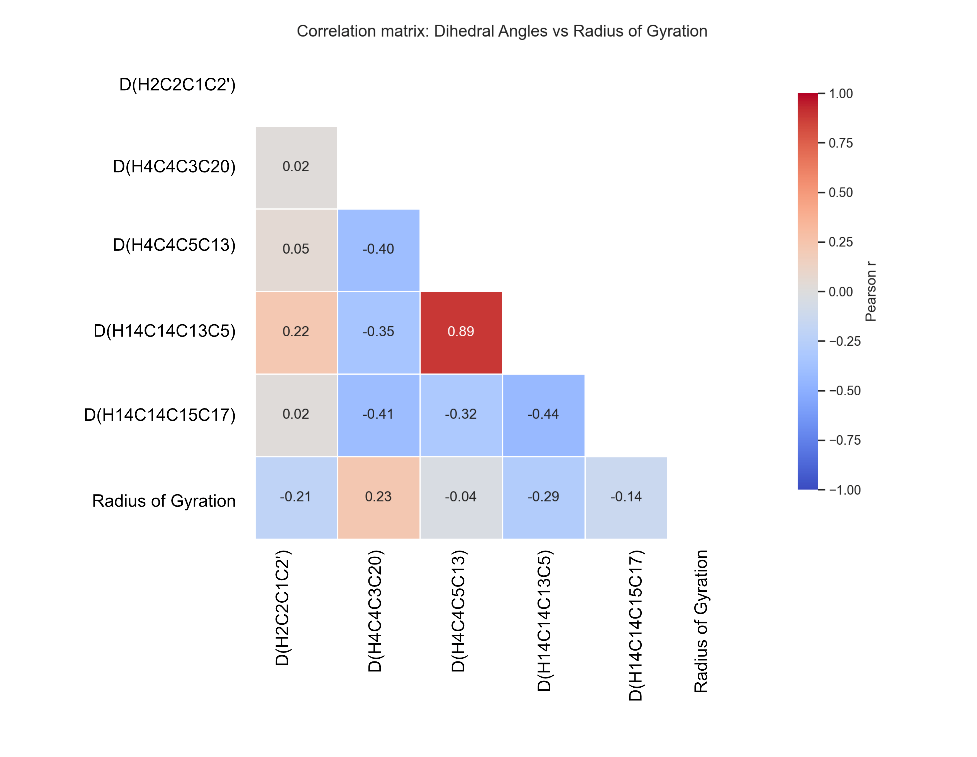


**Figure S82**. Correlation matrix among molecular and geometrical descriptors of conformers of RSR-**1b**, expressed in Pearson correlation coefficient (PCC), after dihedral H14-C14-C15-C17 removal.


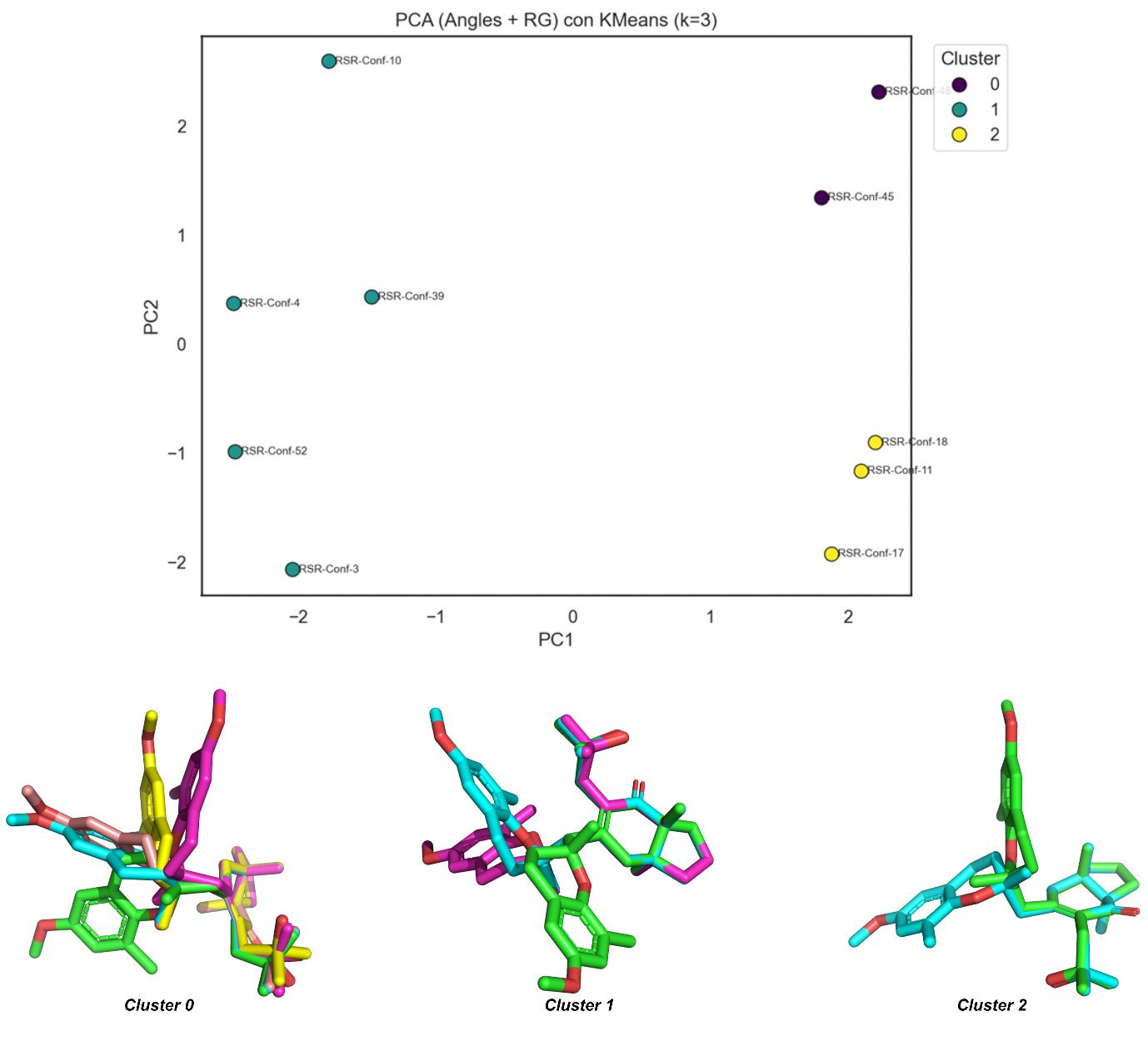


**Figure S83.** PCA of the conformational landscape of **1b** (*RSR*) based on significant dihedral and torsional descriptors and other molecular descriptors. The optimal number of groups was determined using the silhouette method and structural relevance. **Cluster 0**: *RSR*-Conf-45, *RSR*-Conf-48; **Cluster 1:** *RSR*-Conf-3, *RSR*-Conf-52, *RSR*-Conf-4, *RSR*-Conf-10, *RSR*-Conf-39, and **Cluster 2:** *RSR*-Conf-18, *RSR*-Conf-11, and RSR-Conf-17


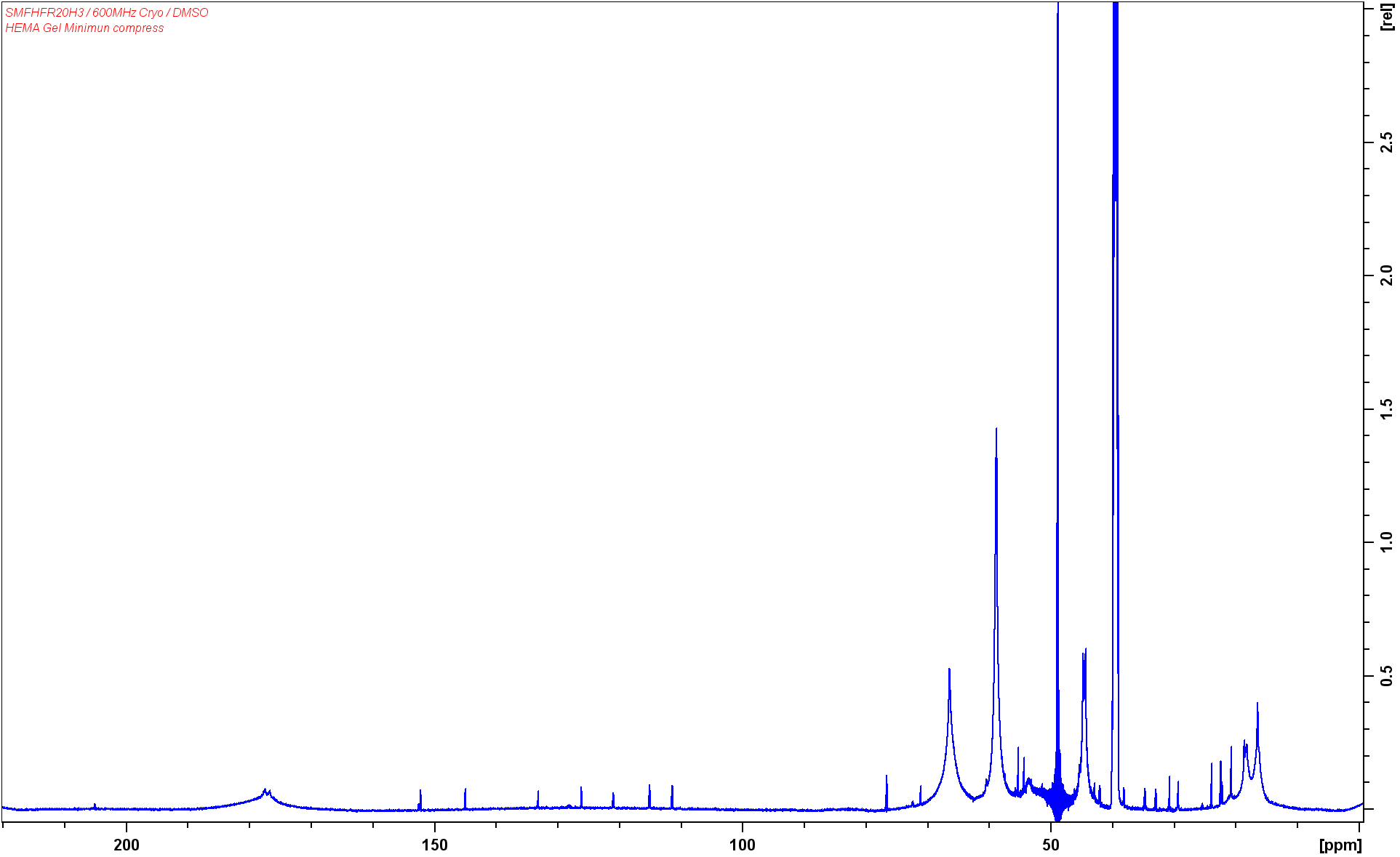


(a)


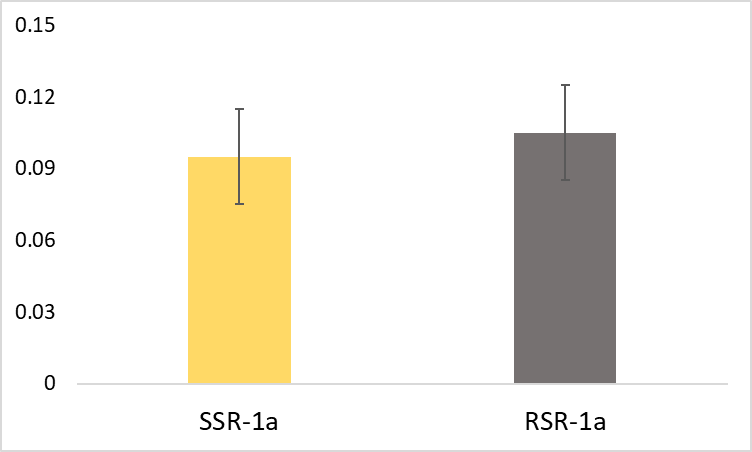


(b)

**Figure S84.** 1D ¹³C-{¹H} NMR spectrum of **1a** oriented in a Poly-HEMA matrix (DMSO-*d_6_*; 5 mm compression device, NS: 32k) measured at 600 MHz (a). Bar plot derived from the ¹³C-RCSA analysis of 1a in a 5 mm compression device (Poly-HEMA/DMSO) recorded at 600 MHz (b). Error bars, derived from the Monte Carlo error analysis, are shown. This analysis was carried out with a sample size of 10 K, assuming a normally distributed error. The average experimental error for the ¹³C RCSA was estimated to be 1.37 ppb.

**3 mm compression device:**

Below, you can find pictures and a detailed description designed to facilitate the construction of the 3 mm compression device. Its use is described here: <https://youtu.be/V8Lf48NJdLg>

**
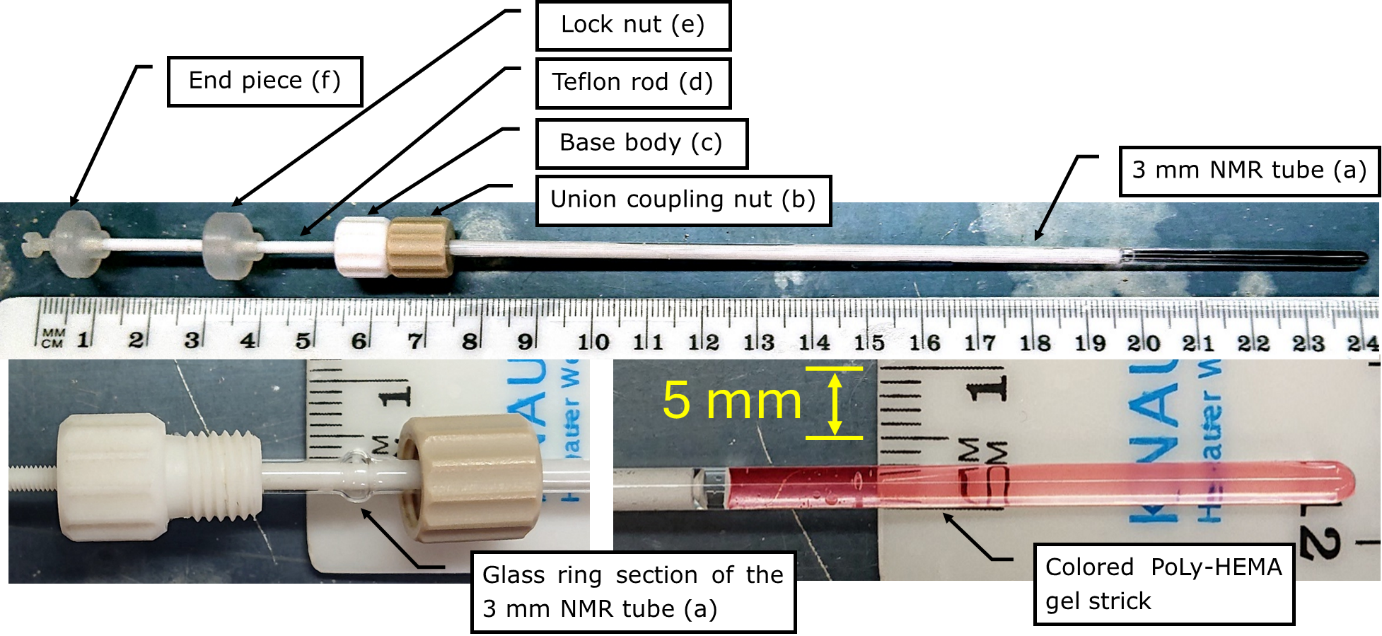
**

**
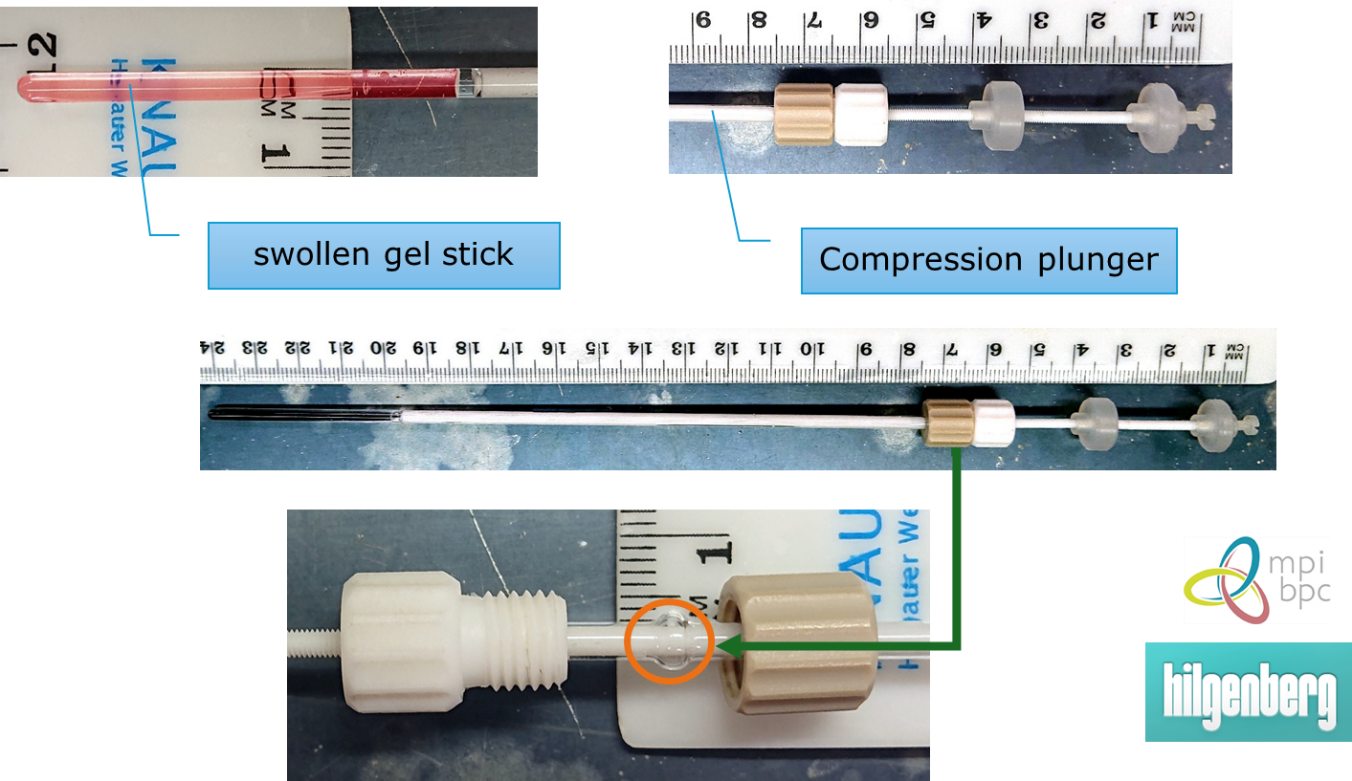
**

**Figure S85**. The 3 mm Semi-Micro Compression Device and its components.


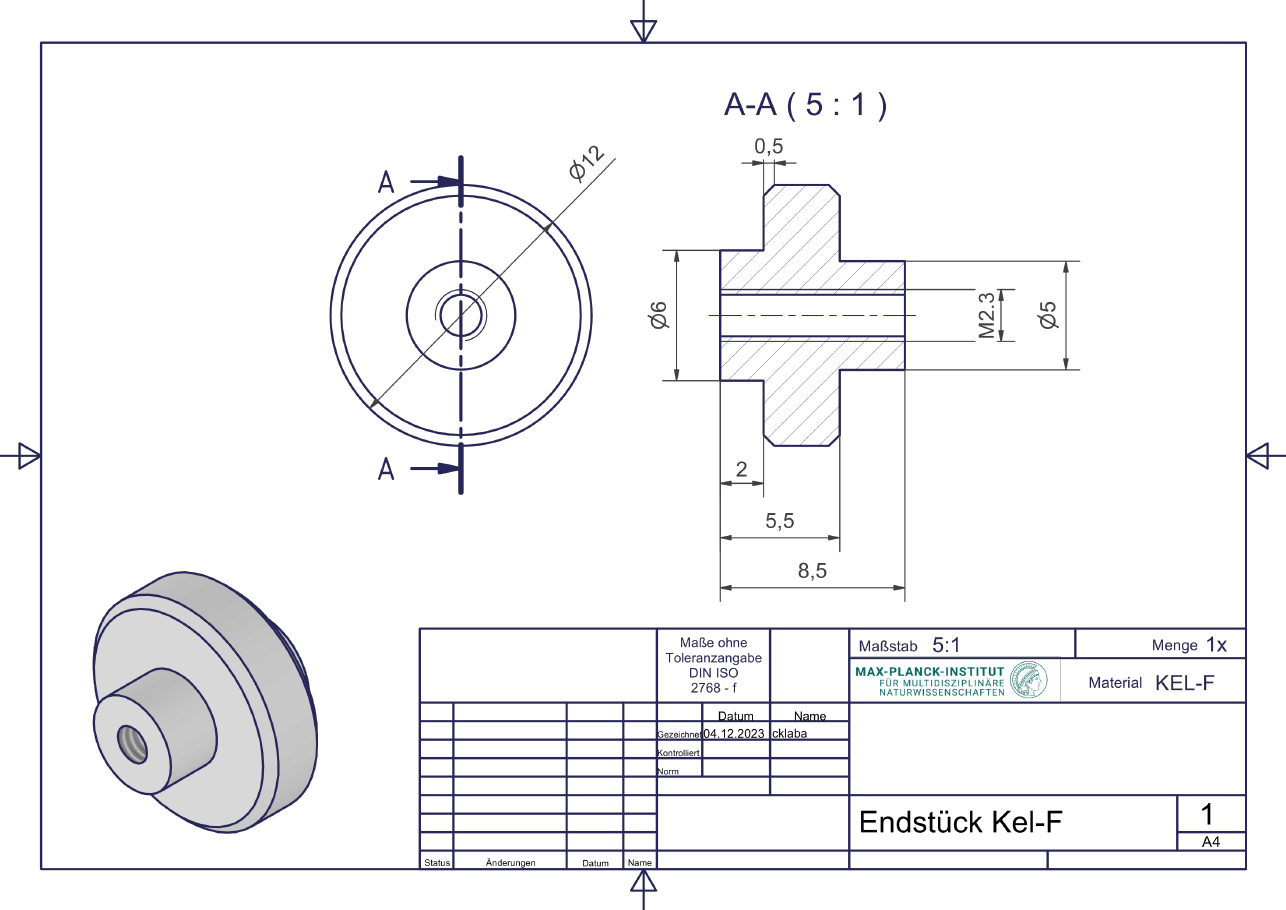


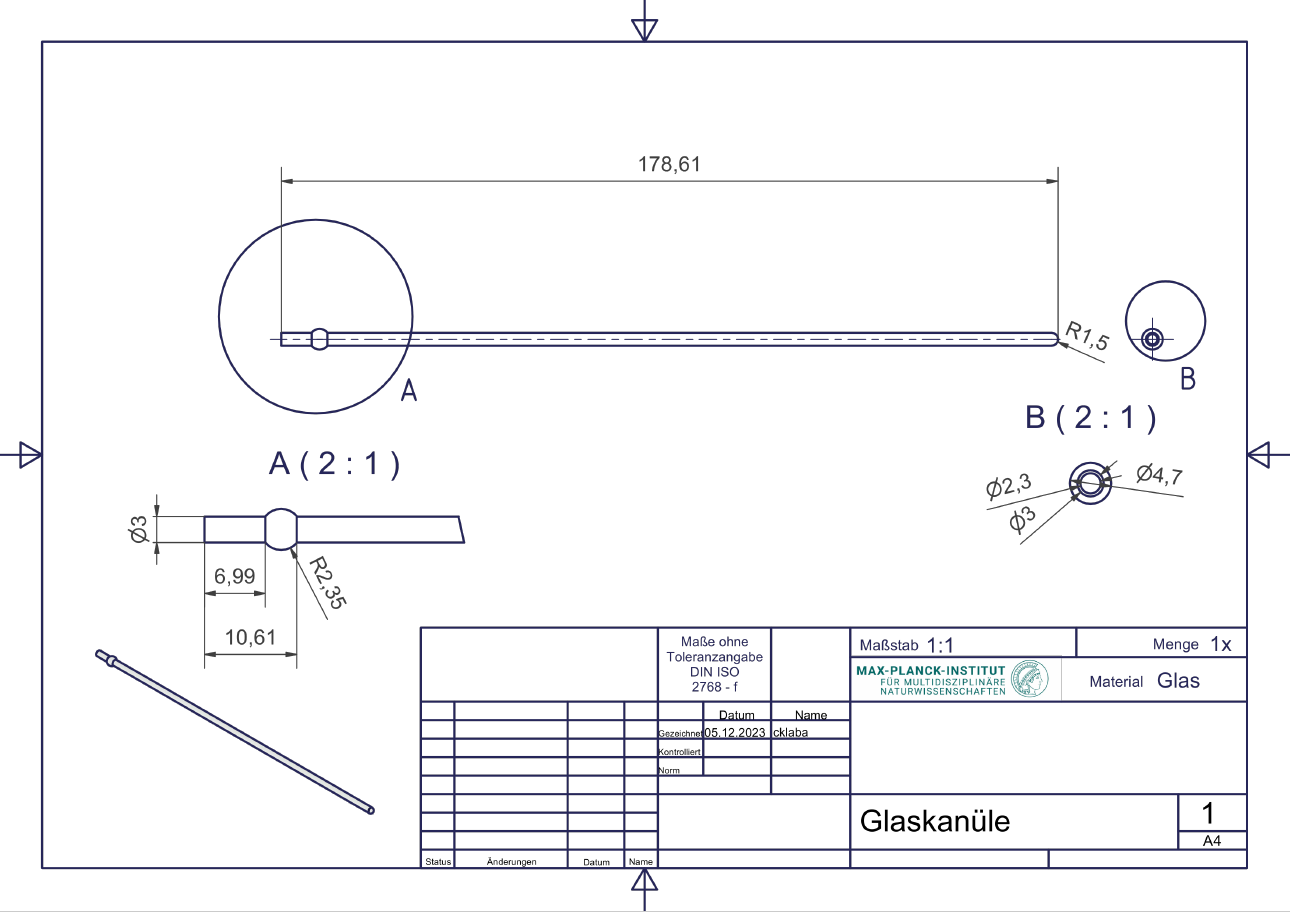


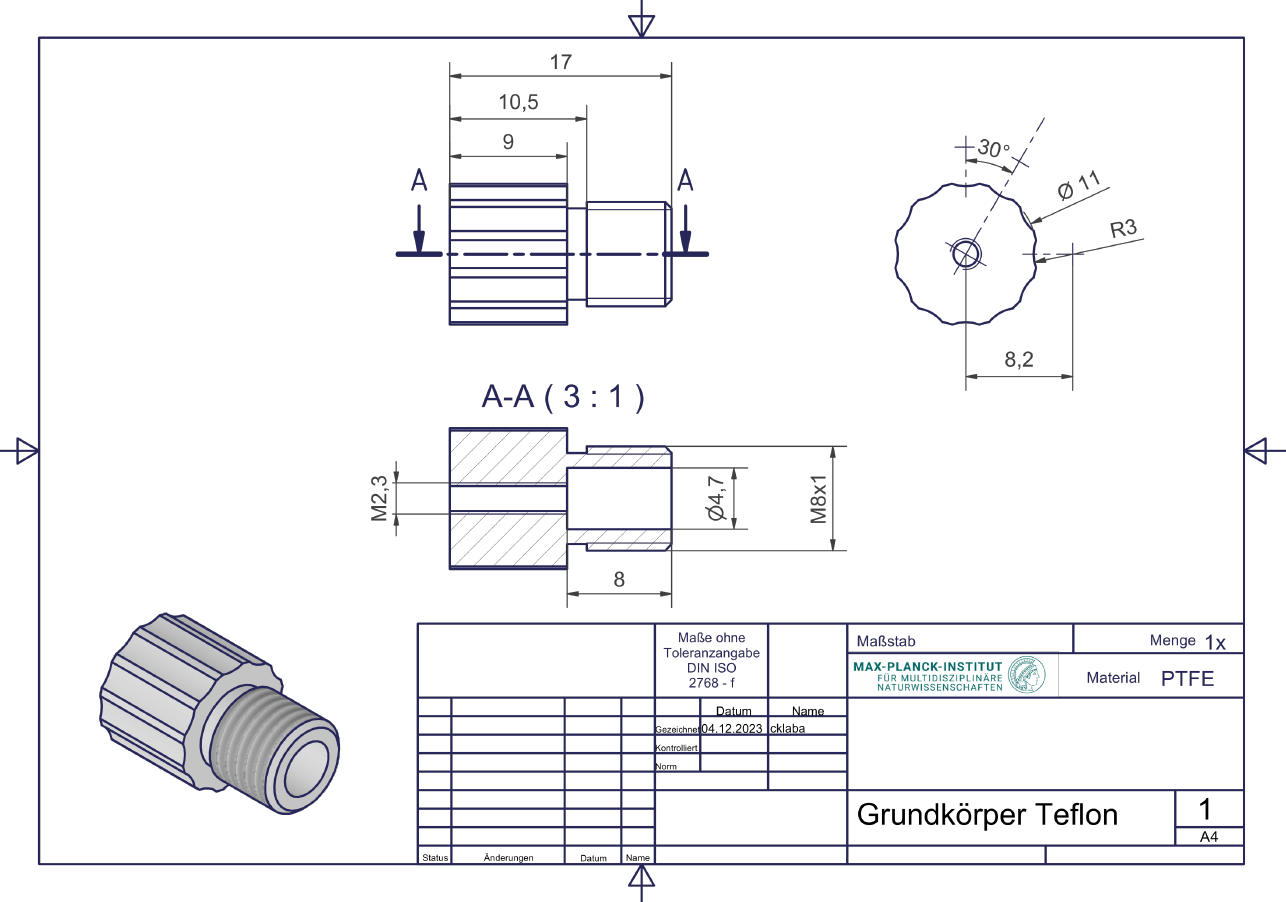


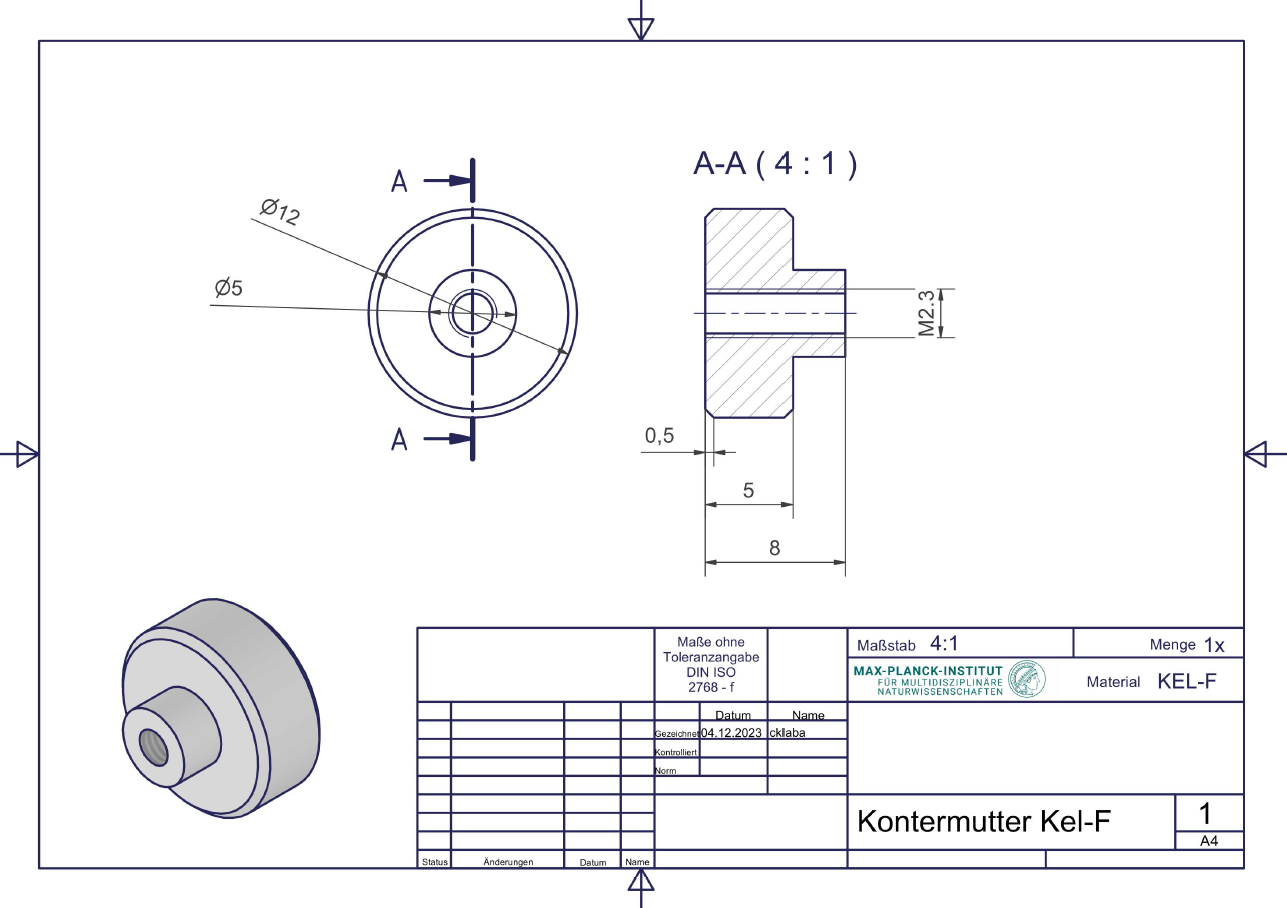


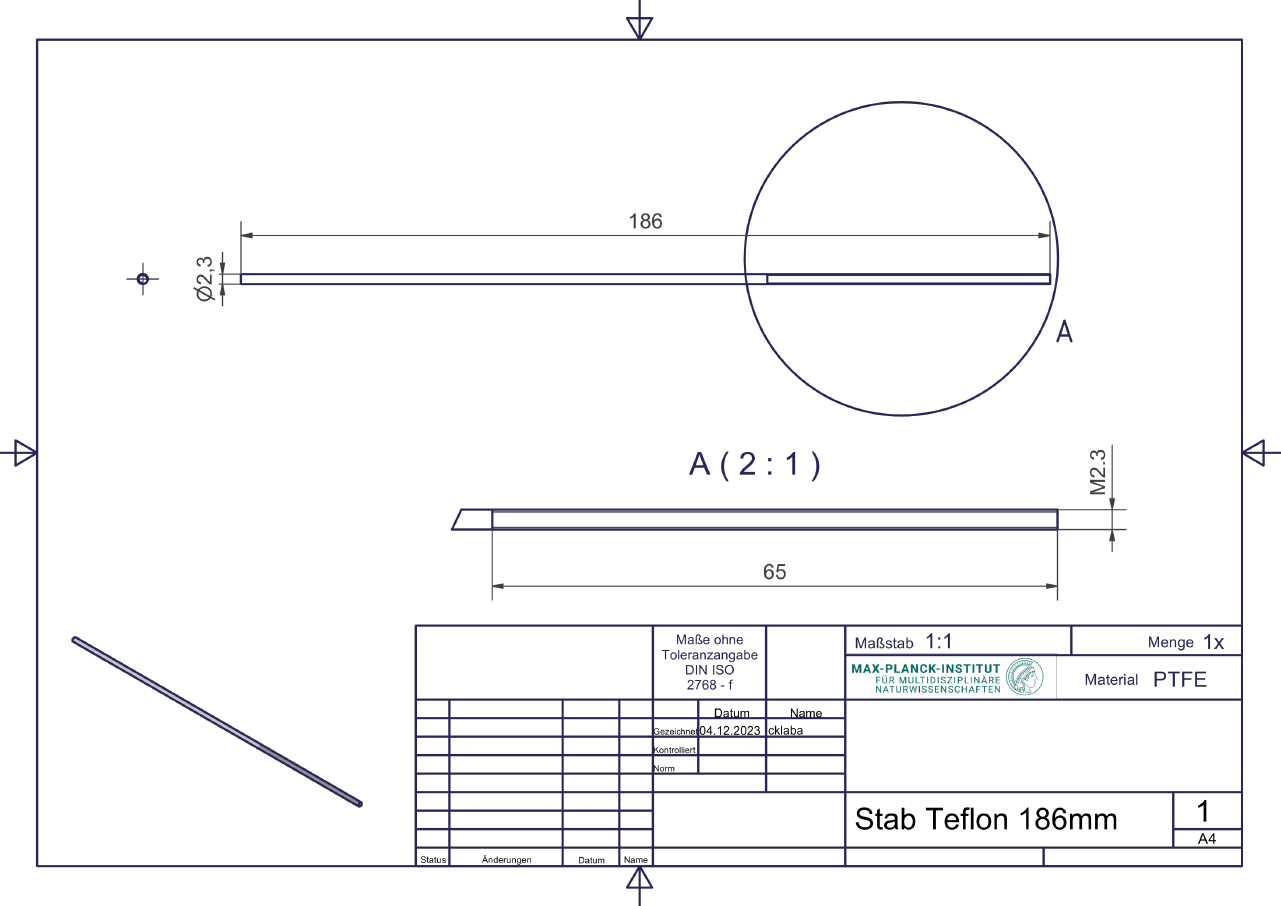


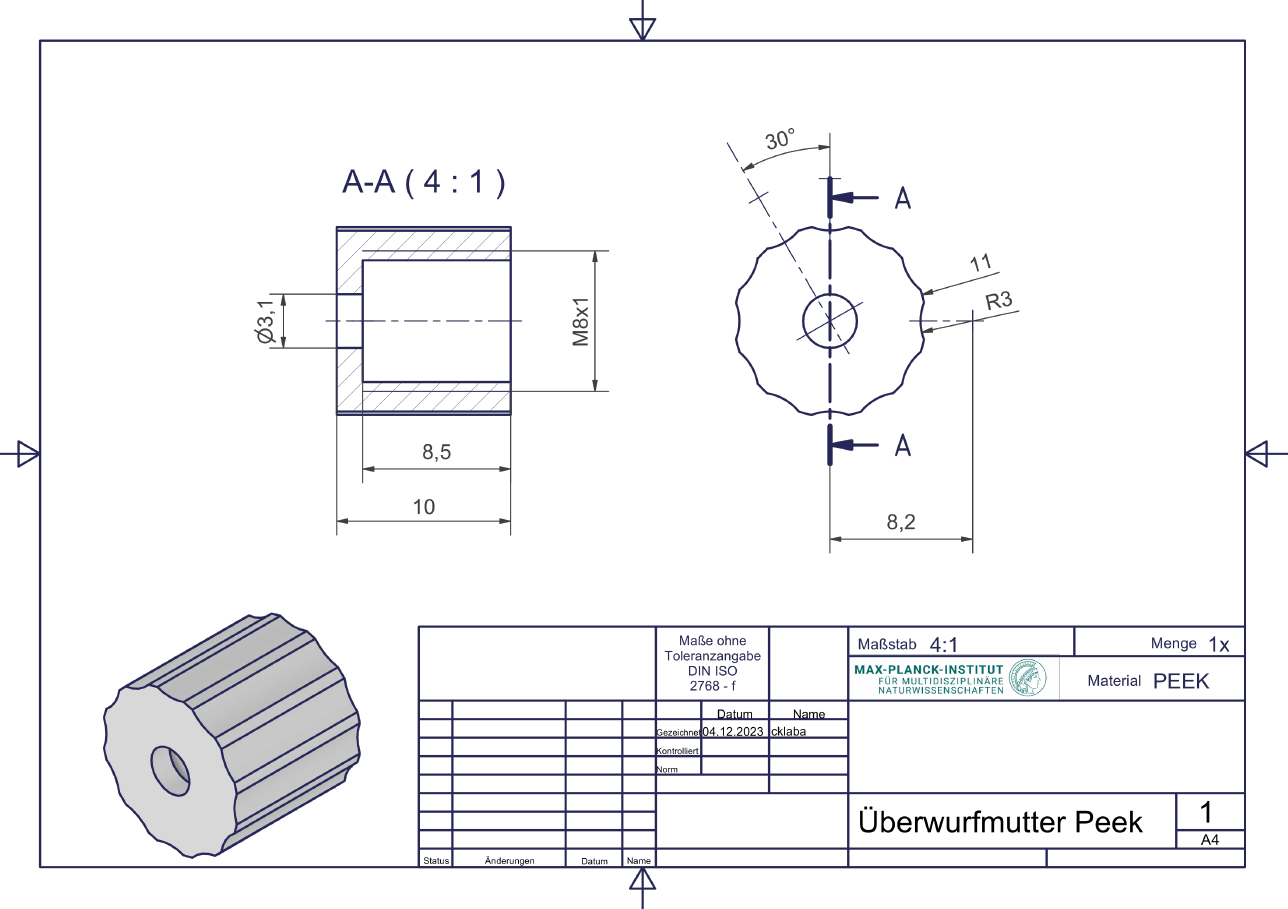


**Figure S86.** Technical drawings of the 3 mm Semi-Micro Compression Device.

**Preparation of Poly-Hema for the 3 mm compression device:**

It was prepared as described elsewhere, but maintaining a monomer concentration of 75 % v/v relative to the polymerization solvent. ^[6,7]^ Experimental details: The sonication of the polymerization solution (a mixture of monomer, solvent, crosslinker, and catalyst) was maintained for 10 minutes. Afterwards, oxygen was removed by passing a continuous argon flow through the polymerization solution for 8 minutes. During the transfer of the polymerization solution to the reaction chambers (1.7 mm NMR capillary tubes), the argon flow was maintained and never interrupted.

After completing the polymerization, gel sticks were sectioned to a length of 4.5 cm while swollen in DMSO. Poly-HEMA gel sticks for the SMCD analysis of **1a** were prepared in 1.7 mm NMR capillary tubes with an inner diameter (ID) of 1.3 mm; nevertheless, 2.0 mm NMR capillary tubes (ID = 1.6 mm) also produced gel sticks suitable for the 3 mm device. The ID of the capillary tube used during gel preparation highly depends on the solvent used for polymer swelling.


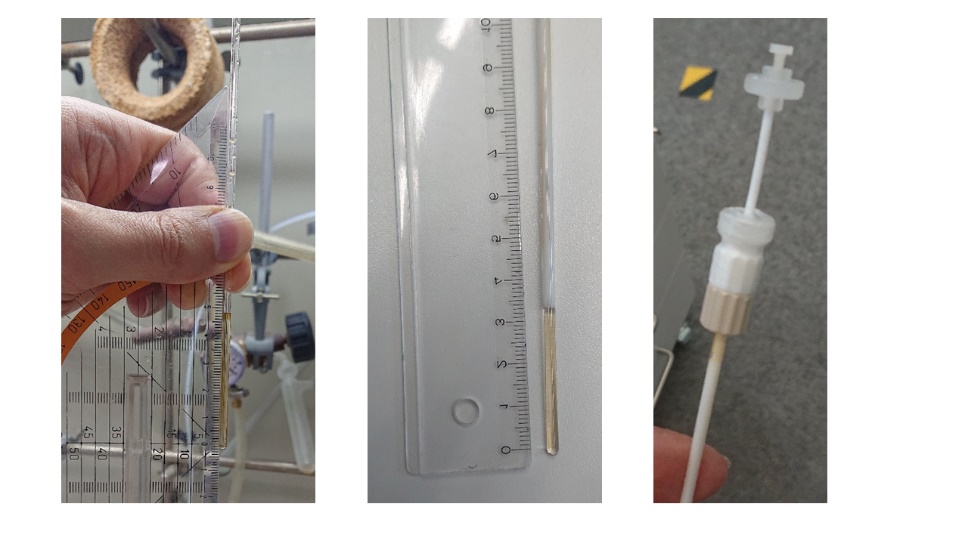


(a)

(b)

(c)

**Figure S87.** Relaxed micro gel stick of Poly-HEMA swollen in a sample of 1a in DMSO-d₆ **(a).** Ideally, the gel stick for the compression device should be 45 mm long. After compression, the gel stick shortens from 45 mm to 34 mm **(b)** and is then ready for anisotropic NMR experiments. The Semi-Micro Compression Device, where the lock nut and the base-body nut are kept tight by being wrapped in Parafilm paper **(c)**, prevents tiny changes in the level of alignment during NMR experiments.

**


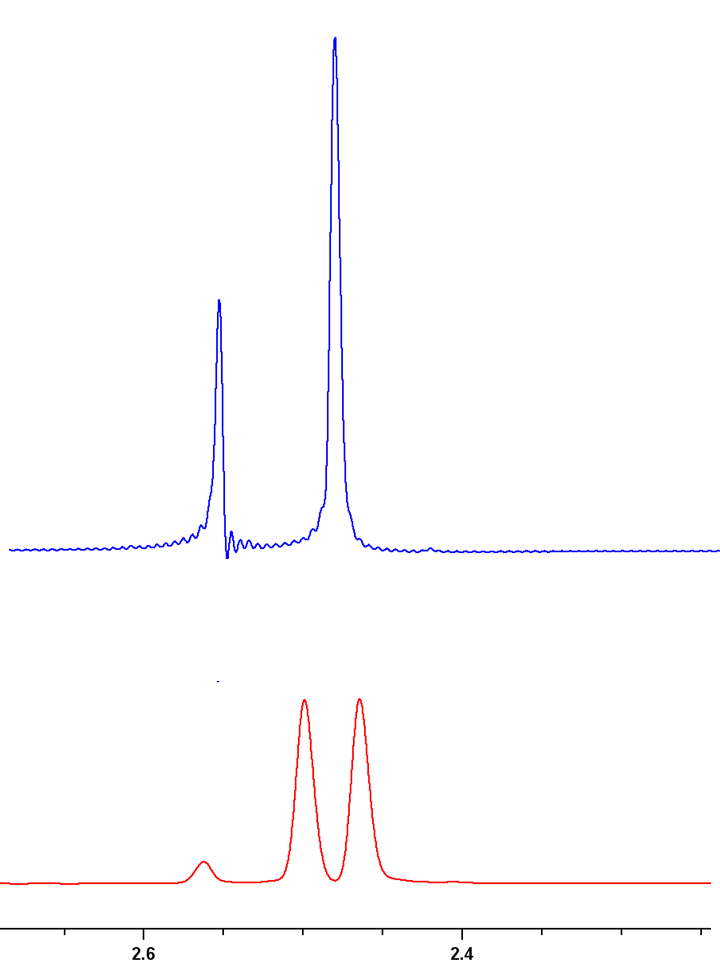


*

(a)

(b)

│-6 Hz-│

**Figure S88.** 1D ²H spectrum of DMSO-d₆ in a 3 mm compression device recorded on a 1.2 GHz Bruker spectrometer (zg2h, Poly-HEMA, NS = 8). The experiment in the relaxed state of the gel sample is shown in blue (a). Differences in magnetic susceptibility between DMSO-d₆ inside () and outside (*) the alignment medium become evident by observing two deuterium signals. The spectrum of the sample after compression is shown in red (b). The ²H residual quadrupolar coupling observed for the analysis of 1a was 6.0 Hz. Interestingly, a tiny deuterium signal from outside the gel stick is still visible (black arrow)

**Cornilescu´s quality factor:**

$$Q=\sqrt{\frac{\sum_{i=1}^{n} \left( {RDC}_{Exptl,i}-{RDC}_{Calc,i} \right)^{2}}{\sum_{i=1}^{n} {RDC}_{Exptl,i}^{2}}}$$

$$Q_{CSA}=\sqrt{\frac{\sum_{i} \left( ({\Delta RCSA}_{i,ax}^{exp}-{\Delta RCSA}_{i}^{theo})/{CSA}_{i,ax} \right)^{2}}{\sum_{i} \left( {\Delta RCSA}_{i}^{exp}/{CSA}_{i,ax} \right)^{2}}}$$

**Table S7** Experimental (Exp.) and back calculated (Comp.) RDC and ^13^C-RCSA of **1a** and **1b**.

| **1a** | | | **1b** | | |
| --- | --- | --- | --- | --- | --- |
| **RDC/^13^C-RCSA** | **Exp. (Hz)** | **Comp. (Hz)** | **RDC/13C-RCSA** | **Exp. (Hz)** | **Comp. (Hz)** |
| C5'-H5' | -12.2 | -11.66 | C5'-H5' | -11.83 | -11.5 |
| C3'-H3' | -9.26 | -7.28 | C3'-H3' | -13.02 | -12.35 |
| C4-H4a,b | -1.92 | -2.65 | C1-H1a,b | -0.89 | -0.98 |
| C1-H1a,b | 0.68 | -0.51 | C2-H2a,b | -2.78 | -2.64 |
| C2-H2a,b | -6.19 | -7.08 | C4-H4a,b | -6.76 | -6.75 |
| C9-H9a,b | -4.26 | -3.74 | C6-H6a,b | 2.91 | 2.99 |
| C10-H10a,b | 3.3 | 3.44 | C8-H8a,b | 0.72 | 1.32 |
| Me16 | -0.36 | 0.49 | C9-H9a,b | -3.79 | -3.79 |
| Me17 | -0.36 | 0.49 | C10-H10a,b | 3.15 | 2.48 |
| Me18 | 1.63 | 3.41 | C14-H14a,b | 3.1 | 3.41 |
| Me19 | 1.72 | 2.33 | Me16 | 1.33 | 0.55 |
| Me20 | -0.9 | -0.24 | Me17 | 1.33 | 0.55 |
| Me-C6' | 0.9 | 2.37 | Me18 | 0.17 | 0.42 |
| C14-H14a,b | -2.16 | -2.57 | Me19 | 0.58 | 0.57 |
| MeO- | 5.27 | 4.24 | Me20 | -0.7 | -0.69 |
| C3 | 3.02 | 2.41 | Me-C6' | 3.58 | 3.74 |
| C5 | -1.21 | -0.91 | C=O | 1.81 | 1.81 |
| C=O | -2.11 | -1.81 | C5 | 0.30 | 0.91 |
| C15 | 5.73 | 7.24 | C4' | -4.53 | -5.13 |
| C1' | 2.11 | 3.32 | C1' | -5.73 | -6.34 |
| C2' | 8.15 | 8.45 | C13 | 0.91 | 0.60 |
| C3' | 7.85 | 8.45 | C6' | 4.83 | 4.83 |
| C4' | 3.32 | 3.92 | C2' | 2.41 | 3.02 |
| C5' | 11.77 | 10.87 | C5' | 3.62 | 4.23 |
| C6' | 9.96 | 10.26 | C3' | 2.72 | 3.02 |
| C11 | 4.83 | 6.34 | C3 | -3.62 | -2.72 |
| C7 | 7.24 | 8.15 | C15 | 0.30 | 0.60 |
| C4 | 6.94 | 6.04 | C11 | -1.51 | -1.51 |
| C6 | 4.53 | 3.92 | C7 | -0.60 | -0.91 |
| C2 | 7.24 | 6.04 | C4 | -0.91 | -0.30 |
| C20 | 6.34 | 6.94 | C6 | -1.81 | -1.81 |
| C19 | 5.43 | 5.13 | C14 | -0.30 | -0.60 |
| C1 | 6.64 | 6.34 | C8 | -3.32 | -3.02 |
| C9 | 7.55 | 7.24 | C16 | -2.11 | -2.11 |
| C-C6' | 5.73 | 3.62 | C17 | -3.02 | -3.62 |
|  |  |  | C10 | -0.91 | -1.51 |
|  |  |  | C20 | -2.11 | -2.11 |
|  |  |  | C19 | -2.11 | -2.11 |
|  |  |  | C1 | -0.91 | -1.21 |
|  |  |  | C18 | -1.21 | -1.81 |
|  |  |  | C9 | -0.91 | -0.60 |
|  |  |  | C-C6' | -3.32 | -3.32 |

RCSAs are expressed in Hz for the reader convenience. Nevertheless, they were in ppm during the RCSA+RDC combined fitting. The reader is invited to visit Determination of Relative Configuration from Residual Chemical Shift Anisotropy^[8]^ and Measurement of residual chemical shift anisotropies in compressed polymethylmethacrylate gels. Automatic compensation of gel isotropic shift contribution.^[9]^


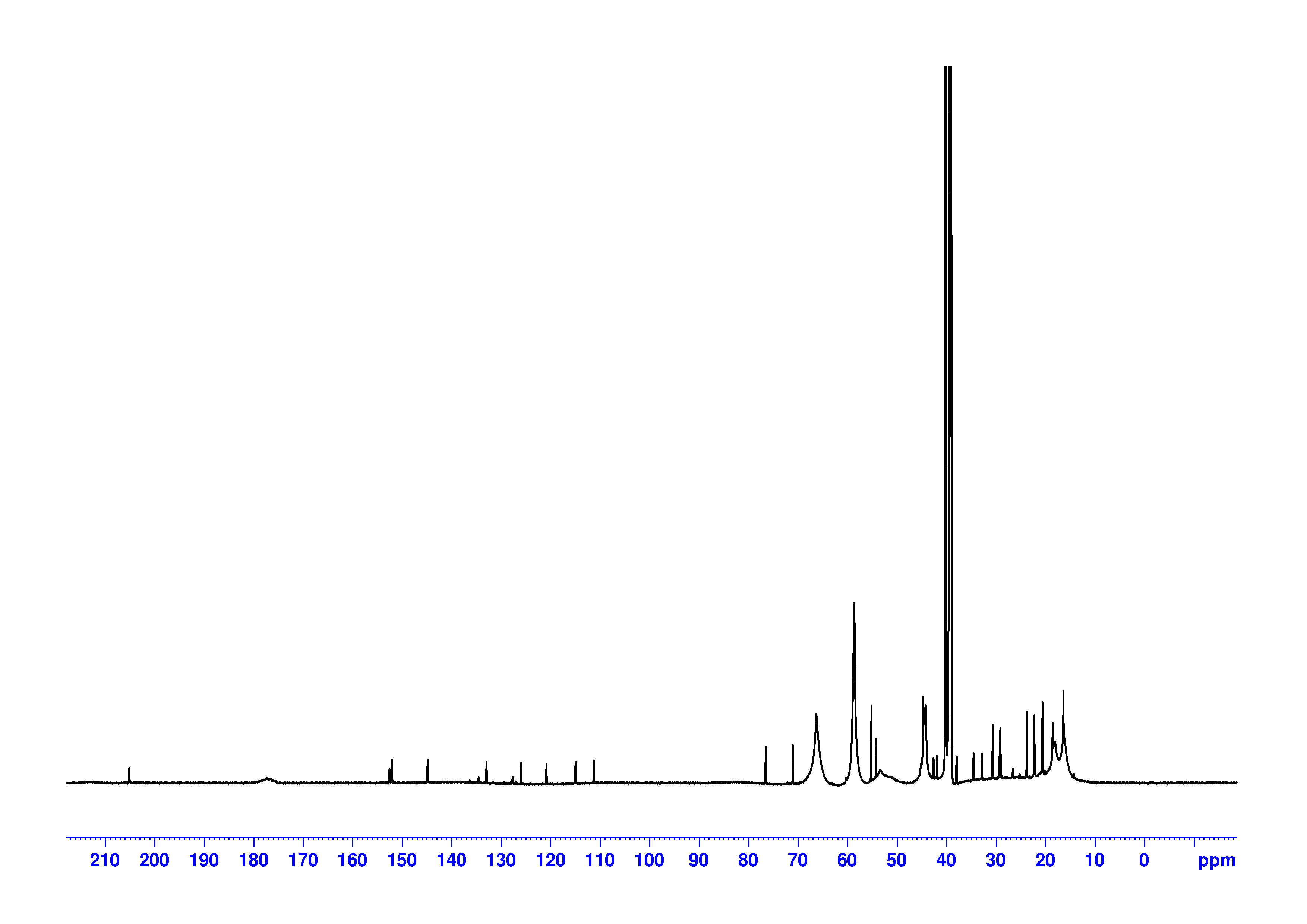


**Figure S89** ¹³C-{¹H} NMR spectrum of **1a** oriented in a Poly-HEMA matrix (DMSO-d_6_) using a 3 mm semi-micro compression device. The experiment was recorded on a 1.2 GHz spectrometer with 32 K.

**Figure S90** ^1^H-^13^C F1-resolved HSQC (hsqcbietgpjcsp.2) spectrum of **1a** oriented in Poly-HEMA (DMSO), recorded on a 1.2 GHz spectrometer using a 3 mm semi micro compression device. Experiment parameters: NS: 72; ^1^J_CH_ = 140 Hz; scaling factor = 6. The stripes along F2 are baseline humps from the gel, which have not been removed since they don’t overlap with peaks of interest.

**Figure S91** ¹³C-{¹H} NMR spectrum of **1b** oriented in a Poly-HEMA matrix (DMSO-d_6_) using a 3 mm semi-micro compression device. The experiment was recorded on a 1.2 GHz spectrometer with 16 K.

**Figure S92** ^1^H-^13^C F1-resolved HSQC (hsqcbietgpjcsp.2) spectrum of **1b** oriented in Poly-HEMA (DMSO), recorded on a 1.2 GHz spectrometer using a 3 mm semi micro compression device. Experiment parameters: NS: 64; ^1^J_CH_ = 140 Hz; scaling factor = 6.

**Single Tensor Analysis**

### ***SSR*-1a**

| **RDC** | **RCSA** | **RCSA+RDC** |
| --- | --- | --- |
| Conformationally averaged solution  Alignment tensor  A'x=-1.362e-04  A'y=-7.443e-04  A'z= 8.806e-04  Saupe tensor  S'x=-2.044e-04  S'y=-1.116e-03  S'z= 1.321e-03  Alignment tensor eigenvectors  e[x]=(-0.568, 0.819, 0.075)  e[y]=(-0.622,-0.488, 0.612)  e[z]=( 0.538, 0.301, 0.787)  Alignment tensor in laboratory coordinates:  [-7.709e-05,-1.959e-05,6.625e-04]  [-1.959e-05,-1.885e-04,4.228e-04]  [ 6.625e-04,4.228e-04,2.656e-04]  SVD condition number is 4.711e+00  Axial component Aa = 1.321e-03  Rhombic component Ar = 6.081e-04  rhombicity R = 0.460  Asimmetry parameter etha =6.906e-01  GDO = 1.697e-03  ZY'Z'' Euler Angles (degrees)  Set 1  (29.2,38.1,97.0)  Set 2  (-150.8,-38.1,-83.0) | Conformationally averaged solution  Alignment tensor  A'x=-4.632e-05  A'y=-7.819e-04  A'z= 8.282e-04  Saupe tensor  S'x=-6.947e-05  S'y=-1.173e-03  S'z= 1.242e-03  Alignment tensor eigenvectors  e[x]=( 0.658,-0.751,-0.054)  e[y]=( 0.582, 0.553,-0.597)  e[z]=( 0.478, 0.361, 0.801)  Alignment tensor in laboratory coordinates:  [-9.546e-05,-8.552e-05,5.902e-04]  [-8.552e-05,-1.569e-04,4.953e-04]  [ 5.902e-04,4.953e-04,2.523e-04]  SVD condition number is 1.020e+01  Axial component Aa = 1.242e-03  Rhombic component Ar = 7.355e-04  rhombicity R = 0.592  Asimmetry parameter etha =8.881e-01  GDO = 1.694e-03  ZY'Z'' Euler Angles (degrees)  Set 1  (37.1,36.8,-84.8)  Set 2  (-142.9,-36.8,95.2) | Conformationally averaged solution  Alignment tensor  A'x=-1.261e-04  A'y=-7.601e-04  A'z= 8.863e-04  Saupe tensor  S'x=-1.892e-04  S'y=-1.140e-03  S'z= 1.329e-03  Alignment tensor eigenvectors  e[x]=( 0.638,-0.767,-0.066)  e[y]=( 0.587, 0.540,-0.603)  e[z]=( 0.498, 0.346, 0.795)  Alignment tensor in laboratory coordinates:  [-9.271e-05,-2.622e-05,6.254e-04]  [-2.622e-05,-1.899e-04,4.853e-04]  [ 6.254e-04,4.853e-04,2.826e-04]  SVD condition number is 5.788e+00  Axial component Aa = 1.329e-03  Rhombic component Ar = 6.340e-04  rhombicity R = 0.477  Asimmetry parameter etha =7.153e-01  GDO = 1.720e-03  ZY'Z'' Euler Angles (degrees)  Set 1  (34.8,37.4,-83.7)  Set 2  (-145.2,-37.4,96.3) |

**Table S8.** Intertensor angle between RDC’s, RCSA’s and (RCSA+RDC)’s derived tensors for the analyses of **1a**

| **Anisotropic data** | **Intertensor angle (Degree)** | **Cos(θ)** |
| --- | --- | --- |
| RCSA+RDC/ RCSA | 5.1 | 0.996_0_ |
| RCSA+RDC/ RDC | 5.2 | 0.995_9_ |
| RCSA / RDC | 8.7 | 0.988_3_ |

### ***RSR*-1a**

| **RDC** | **RCSA** | **RCSA+RDC** |
| --- | --- | --- |
| Conformationally averaged solution  Alignment tensor  A'x= 1.464e-05  A'y= 6.195e-04  A'z=-6.342e-04  Saupe tensor  S'x= 2.196e-05  S'y= 9.293e-04  S'z=-9.513e-04  Alignment tensor eigenvectors  e[x]=( 0.714, 0.269, 0.646)  e[y]=(-0.088,-0.881, 0.464)  e[z]=( 0.694,-0.388,-0.606)  Alignment tensor in laboratory coordinates:  [-2.933e-04,2.217e-04,2.483e-04]  [ 2.217e-04,3.869e-04,-4.001e-04]  [ 2.483e-04,-4.001e-04,-9.353e-05]  SVD condition number is 5.143e+00  Axial component Aa = -9.513e-04  Rhombic component Ar = -6.049e-04  rhombicity R = 0.636  Asimmetry parameter etha =9.538e-01  GDO = 1.325e-03  ZY'Z'' Euler Angles (degrees)  Set 1  (-29.2,127.3,144.3)  Set 2  (150.8,-127.3,-35.7) | Conformationally averaged solution  Alignment tensor  A'x=-1.916e-04  A'y=-5.354e-04  A'z= 7.269e-04  Saupe tensor  S'x=-2.873e-04  S'y=-8.031e-04  S'z= 1.090e-03  Alignment tensor eigenvectors  e[x]=( 0.803, 0.213, 0.557)  e[y]=( 0.587,-0.449,-0.674)  e[z]=( 0.106, 0.868,-0.485)  Alignment tensor in laboratory coordinates:  [-2.997e-04,1.752e-04,8.867e-05]  [ 1.752e-04,4.311e-04,-4.908e-04]  [ 8.867e-05,-4.908e-04,-1.314e-04]  SVD condition number is 2.737e+01  Axial component Aa = 1.090e-03  Rhombic component Ar = 3.438e-04  rhombicity R = 0.315  Asimmetry parameter etha =4.730e-01  GDO = 1.328e-03  ZY'Z'' Euler Angles (degrees)  Set 1  (83.0,119.0,-129.6)  Set 2  (-97.0,-119.0,50.4) | Conformationally averaged solution  Alignment tensor  A'x=-9.338e-05  A'y=-6.173e-04  A'z= 7.107e-04  Saupe tensor  S'x=-1.401e-04  S'y=-9.260e-04  S'z= 1.066e-03  Alignment tensor eigenvectors  e[x]=( 0.768, 0.149, 0.623)  e[y]=( 0.623,-0.401,-0.672)  e[z]=( 0.149, 0.904,-0.401)  Alignment tensor in laboratory coordinates:  [-2.790e-04,2.394e-04,1.712e-04]  [ 2.394e-04,4.796e-04,-4.321e-04]  [ 1.712e-04,-4.321e-04,-2.006e-04]  SVD condition number is 7.203e+00  Axial component Aa = 1.066e-03  Rhombic component Ar = 5.239e-04  rhombicity R = 0.491  Asimmetry parameter etha =7.372e-01  GDO = 1.388e-03  ZY'Z'' Euler Angles (degrees)  Set 1  (80.6,113.6,-132.9)  Set 2  (-99.4,-113.6,47.1) |

### ***RSR*-1b**

| **RDC** | **RCSA** | **RCSA+RDC** |
| --- | --- | --- |
| Conformationally averaged solution  Alignment tensor  A'x= 8.569e-05  A'y= 4.107e-04  A'z=-4.964e-04  Saupe tensor  S'x= 1.285e-04  S'y= 6.161e-04  S'z=-7.447e-04  Alignment tensor eigenvectors  e[x]=(-0.358, 0.427,-0.830)  e[y]=( 0.372, 0.881, 0.293)  e[z]=( 0.856,-0.203,-0.475)  Alignment tensor in laboratory coordinates:  [-2.964e-04,2.078e-04,2.720e-04]  [ 2.078e-04,3.138e-04,2.773e-05]  [ 2.720e-04,2.773e-05,-1.747e-05]  SVD condition number is 8.754e+00  Axial component Aa = -7.447e-04  Rhombic component Ar = -3.251e-04  rhombicity R = 0.437  Asimmetry parameter etha =6.548e-01  GDO = 9.476e-04  ZY'Z'' Euler Angles (degrees)  Set 1  (-13.4,118.3,19.4)  Set 2  (166.6,-118.3,-160.6) | Conformationally averaged solution  Alignment tensor  A'x= 6.237e-05  A'y= 3.700e-04  A'z=-4.324e-04  Saupe tensor  S'x= 9.356e-05  S'y= 5.551e-04  S'z=-6.486e-04  Alignment tensor eigenvectors  e[x]=(-0.331, 0.419,-0.845)  e[y]=( 0.373, 0.881, 0.291)  e[z]=( 0.867,-0.219,-0.448)  Alignment tensor in laboratory coordinates:  [-2.668e-04,1.948e-04,2.255e-04]  [ 1.948e-04,2.777e-04,3.034e-05]  [ 2.255e-04,3.034e-05,-1.096e-05]  SVD condition number is 6.727e+00  Axial component Aa = -6.486e-04  Rhombic component Ar = -3.077e-04  rhombicity R = 0.474  Asimmetry parameter etha =7.115e-01  GDO = 8.384e-04  ZY'Z'' Euler Angles (degrees)  Set 1  (-14.2,116.6,19.0)  Set 2  (165.8,-116.6,-161.0) | Conformationally averaged solution  Alignment tensor  A'x= 7.667e-05  A'y= 3.969e-04  A'z=-4.735e-04  Saupe tensor  S'x= 1.150e-04  S'y= 5.953e-04  S'z=-7.103e-04  Alignment tensor eigenvectors  e[x]=(-0.341, 0.423,-0.839)  e[y]=( 0.378, 0.879, 0.290)  e[z]=( 0.861,-0.218,-0.460)  Alignment tensor in laboratory coordinates:  [-2.854e-04,2.097e-04,2.528e-04]  [ 2.097e-04,2.981e-04,2.640e-05]  [ 2.528e-04,2.640e-05,-1.269e-05]  SVD condition number is 5.521e+00  Axial component Aa = -7.103e-04  Rhombic component Ar = -3.202e-04  rhombicity R = 0.451  Asimmetry parameter etha =6.762e-01  GDO = 9.091e-04  ZY'Z'' Euler Angles (degrees)  Set 1  (-14.2,117.4,19.0)  Set 2  (165.8,-117.4,-161.0) |

**Table S9.** Intertensor angle between RDC’s, RCSA’s and (RCSA+RDC)’s derived tensors for the analyses of **1b**

| **Anisotropic data** | **Intertensor angle (Degree)** | **Cos(θ)** |
| --- | --- | --- |
| RCSA+RDC/ RCSA | 1.5 | 0.999_6_ |
| RCSA+RDC/ RDC | 1.8 | 0.999_5_ |
| RCSA / RDC | 2.8 | 0.998_7_ |

**Table S10**. Intertensor angle between (RCSA+RDC)’s derived tensors for the analyses of **1a** and **1b**

| **Anisotropic data** | **Intertensor angle (Degree)** | **Cos(θ)** |
| --- | --- | --- |
| RCSA+RDC-SSR-1a/RSR-1b | 66.0 | 0.406_3_ |

### *SSR*-1b

| **RDC** | **RCSA** | **RCSA+RDC** |
| --- | --- | --- |
| Conformationally averaged solution  Alignment tensor  A'x=-4.792e-05  A'y=-3.770e-04  A'z= 4.249e-04  Saupe tensor  S'x=-7.188e-05  S'y=-5.655e-04  S'z= 6.374e-04  Alignment tensor eigenvectors  e[x]=(-0.300, 0.397, 0.867)  e[y]=( 0.954, 0.136, 0.268)  e[z]=(-0.012, 0.908,-0.419)  Alignment tensor in laboratory coordinates:  [-3.473e-04,-4.797e-05,-8.163e-05]  [-4.797e-05,3.356e-04,-1.920e-04]  [-8.163e-05,-1.920e-04,1.165e-05]  SVD condition number is 5.200e+00  Axial component Aa = 6.374e-04  Rhombic component Ar = 3.291e-04  rhombicity R = 0.516  Asimmetry parameter etha =7.745e-01  GDO = 8.392e-04  ZY'Z'' Euler Angles (degrees)  Set 1  (90.8,114.8,162.9)  Set 2  (-89.2,-114.8,-17.1) | Conformationally averaged solution  Alignment tensor  A'x=-1.641e-05  A'y=-3.901e-04  A'z= 4.065e-04  Saupe tensor  S'x=-2.462e-05  S'y=-5.851e-04  S'z= 6.097e-04  Alignment tensor eigenvectors  e[x]=(-0.427, 0.426, 0.797)  e[y]=( 0.904, 0.186, 0.385)  e[z]=( 0.016, 0.885,-0.465)  Alignment tensor in laboratory coordinates:  [-3.216e-04,-5.691e-05,-1.332e-04]  [-5.691e-05,3.020e-04,-2.008e-04]  [-1.332e-04,-2.008e-04,1.958e-05]  SVD condition number is 6.936e+00  Axial component Aa = 6.097e-04  Rhombic component Ar = 3.737e-04  rhombicity R = 0.613  Asimmetry parameter etha =9.192e-01  GDO = 8.397e-04  ZY'Z'' Euler Angles (degrees)  Set 1  (89.0,117.7,154.2)  Set 2  (-91.0,-117.7,-25.8) | Conformationally averaged solution  Alignment tensor  A'x=-2.326e-05  A'y=-3.936e-04  A'z= 4.169e-04  Saupe tensor  S'x=-3.489e-05  S'y=-5.904e-04  S'z= 6.253e-04  Alignment tensor eigenvectors  e[x]=(-0.347, 0.396, 0.850)  e[y]=( 0.938, 0.156, 0.310)  e[z]=(-0.010, 0.905,-0.425)  Alignment tensor in laboratory coordinates:  [-3.489e-04,-5.830e-05,-1.058e-04]  [-5.830e-05,3.281e-04,-1.874e-04]  [-1.058e-04,-1.874e-04,2.079e-05]  SVD condition number is 4.414e+00  Axial component Aa = 6.253e-04  Rhombic component Ar = 3.703e-04  rhombicity R = 0.592  Asimmetry parameter etha =8.884e-01  GDO = 8.527e-04  ZY'Z'' Euler Angles (degrees)  Set 1  (90.6,115.2,160.0)  Set 2  (-89.4,-115.2,-20.0) |

$$\Delta Q=\sqrt{\left| {Q_{1a}}^{2}-{Q_{1b}}^{2} \right|}$$

**Table S11.** Quality factor, QCSA and discrimination power of RDC and RCSA over **1a** and **1b**.

| **Quality factor** | **ΔQ 1a to 1b** | **ΔQ 1b to 1a** |
| --- | --- | --- |
| Q-RDC | **0.40** | **0.14** |
| Q-RCSA | 0.21 | 0.26 |
| Q-CSA | 0.20 | 0.23 |
| Q-RDC/RCSA | 0.29 | 0.20 |

**Table S12**. Montecarlo error analysis for the anisotropic NMR parameters of **1a** and **1b**

| **Fitting** | **Configuration** | **Q** | **Std. Dev** | **Highest Q** | **Lowest Q** |
| --- | --- | --- | --- | --- | --- |
| RCSA analysis of 1a | *SSR* | 0.130 | 0.006 | 0.153 | 0.109 |
|  | *RSR* | 0.243 | 0.006 | 0.214 | 0.269 |
| RDC fitting of 1a | *SSR* | 0.185 | 0.005 | 0.207 | 0.167 |
|  | *RSR* | 0.441 | 0.005 | 0.463 | 0.421 |
| RDC+RCSA fitting of 1a | *SSR* | 0.167 | 0.004 | 0.185 | 0.152 |
|  | *RSR* | 0.337 | 0.005 | 0.353 | 0.32 |
| RCSA fitting of 1b | *SSR* | 0.307 | 0.017 | 0.376 | 0.246 |
|  | *RSR* | 0.161 | 0.015 | 0.222 | 0.109 |
| RDC fitting of 1b | *SSR* | 0.154 | 0.001 | 0.160 | 0.148 |
|  | *RSR* | 0.064 | 0.001 | 0.069 | 0.058 |
| RDC+RCSA fitting of 1b | *SSR* | 0.226 | 0.007 | 0.203 | 0.257 |
|  | *RSR* | 0.115 | 0.007 | 0.142 | 0.090 |

### **Conformers coordinates -*SSR* (XYZ format)**

72

#1

C -4.601602 0.978891 -0.120354

C -5.073020 -0.114843 -0.851044

C -4.410418 -1.337663 -0.764826

C -3.271337 -1.483428 0.025003

C -2.789882 -0.369421 0.726062

C -3.458539 0.861363 0.674741

C -2.577289 -2.821140 0.141579

C -1.617772 -2.825213 1.328542

C -0.783210 -1.541435 1.369170

O -1.674946 -0.420228 1.529181

C 3.026094 -1.790859 0.446785

C 2.369171 -0.453813 0.194023

C 1.034309 -0.273419 0.019356

C 0.036025 -1.405866 0.055212

C -2.963519 2.028904 1.488230

O -6.176505 -0.081276 -1.657142

C -6.877071 1.146920 -1.761647

C 3.528097 -2.508871 -0.828643

C 4.079124 -3.877031 -0.449124

O 2.424790 -2.773095 -1.704614

C 4.580880 -1.693576 -1.576923

C 3.293619 0.730530 0.189797

C 2.738857 2.008545 -0.392779

C 1.320009 2.232330 0.191225

C 0.416886 1.086738 -0.244798

C 3.453471 3.320568 -0.050341

C 2.346097 4.398716 -0.241627

C 0.988093 3.640192 -0.322305

C 1.354877 2.287492 1.733610

C 2.748487 1.832955 -1.928985

O 4.439361 0.643153 0.610277

C 0.098883 -1.512248 2.606628

H 2.052196 -1.926492 -1.989367

H -5.112875 1.934705 -0.150039

H -4.800805 -2.188933 -1.316791

H -2.037006 -3.041364 -0.787464

H -3.325839 -3.610931 0.256532

H -0.952653 -3.693254 1.292189

H -2.184217 -2.879266 2.266034

H 2.339858 -2.471655 0.956403

H 3.883738 -1.635419 1.108952

H -0.672707 -1.247578 -0.768670

H 0.523592 -2.366318 -0.128924

H -3.687178 2.846284 1.464039

H -2.010954 2.411979 1.106159

H -2.795639 1.739263 2.529523

H -7.705793 0.964318 -2.444362

H -6.233925 1.934352 -2.170247

H -7.268508 1.463356 -0.788437

H 3.305600 -4.473768 0.043834

H 4.927327 -3.770454 0.232815

H 4.414390 -4.409435 -1.343986

H 5.435537 -1.470970 -0.932305

H 4.166212 -0.742039 -1.927267

H 4.924852 -2.256177 -2.449792

H -0.548935 1.130061 0.265937

H 0.192389 1.149840 -1.320367

H 4.320021 3.493673 -0.694485

H 3.817818 3.301376 0.980654

H 2.517329 4.979100 -1.152228

H 2.352545 5.108704 0.589730

H 0.207216 4.117391 0.278995

H 0.621637 3.598709 -1.353295

H 1.826995 1.406360 2.179362

H 0.329077 2.343569 2.114391

H 1.888495 3.170834 2.094727

H 2.349446 2.722516 -2.424296

H 2.175300 0.965252 -2.266723

H 3.783023 1.706399 -2.264801

H -0.521700 -1.614982 3.501028

H 0.651869 -0.571155 2.668412

H 0.814755 -2.338192 2.583125

72

#2

C -4.623208 0.537397 -1.049705

C -5.153994 -0.726873 -0.778848

C -4.456247 -1.605397 0.047563

C -3.234535 -1.241911 0.612674

C -2.716060 0.029137 0.335192

C -3.401344 0.925293 -0.494831

C -2.475777 -2.192192 1.507799

C -1.023142 -1.746144 1.657849

C -0.941474 -0.244839 1.941845

O -1.508830 0.464803 0.824309

C 0.978047 1.981307 -0.393529

C 1.681765 0.671332 -0.128042

C 1.475529 -0.096680 0.972952

C 0.503062 0.284849 2.070624

C -2.809095 2.278754 -0.783372

O -6.340900 -1.184250 -1.281548

C -7.072612 -0.313237 -2.127344

C 1.760532 3.236124 0.056984

C 0.882044 4.464031 -0.145616

O 2.009968 3.175366 1.468244

C 3.088803 3.400055 -0.679621

C 2.632619 0.210312 -1.198145

C 3.630386 -0.845960 -0.788287

C 2.862217 -1.964055 -0.035729

C 2.248721 -1.380070 1.231096

C 4.355188 -1.617047 -1.896988

C 4.751003 -2.957727 -1.211757

C 3.950844 -3.035938 0.121652

C 1.738407 -2.556055 -0.911140

C 4.691808 -0.137995 0.085580

O 2.604075 0.690761 -2.322488

C -1.712948 0.142360 3.205720

H 2.552894 2.393868 1.645010

H -5.145699 1.238997 -1.690454

H -4.875932 -2.588711 0.244182

H -2.519174 -3.203588 1.092170

H -2.960476 -2.239474 2.490873

H -0.482844 -1.948764 0.726150

H -0.532739 -2.302943 2.462636

H 0.009264 1.990588 0.106496

H 0.795892 2.065842 -1.470456

H 0.895444 -0.095457 3.022083

H 0.434668 1.372134 2.162591

H -3.515986 2.901216 -1.335930

H -1.893002 2.187155 -1.376733

H -2.535034 2.791494 0.144074

H -7.967959 -0.859309 -2.421381

H -6.494624 -0.051155 -3.020437

H -7.360083 0.601651 -1.597212

H -0.048469 4.361852 0.421369

H 0.634616 4.584764 -1.204304

H 1.404000 5.362939 0.195836

H 2.935256 3.428141 -1.761970

H 3.768573 2.570416 -0.457123

H 3.572896 4.327896 -0.360556

H 1.595944 -2.111067 1.720176

H 3.031401 -1.149541 1.969952

H 5.223821 -1.067737 -2.270539

H 3.690547 -1.783394 -2.749109

H 5.826304 -2.998002 -1.018570

H 4.515813 -3.805631 -1.860637

H 3.520753 -4.027741 0.293033

H 4.593200 -2.808874 0.978803

H 0.994471 -1.807676 -1.203940

H 1.221914 -3.339599 -0.346297

H 2.130811 -3.015864 -1.821883

H 4.265097 0.367888 0.955866

H 5.208572 0.612151 -0.521943

H 5.444599 -0.848710 0.437756

H -1.554508 1.201253 3.428506

H -1.371670 -0.452046 4.058625

H -2.786297 -0.021387 3.077696

72

#3

C -4.115653 0.511563 1.456317

C -4.818022 0.339138 0.255436

C -4.338490 -0.546332 -0.702022

C -3.145714 -1.254520 -0.478998

C -2.445229 -1.047091 0.706919

C -2.932605 -0.170296 1.697782

C -2.648455 -2.254999 -1.494962

C -1.610710 -3.175385 -0.859066

C -0.579016 -2.375496 -0.056093

O -1.257330 -1.673175 1.003265

C 0.091664 1.325608 0.387282

C 1.358677 0.545883 0.105281

C 1.381934 -0.677334 -0.485787

C 0.126831 -1.356135 -0.991988

C -2.171605 -0.004539 2.984613

O -5.960886 1.076502 0.123760

C -6.712342 0.905748 -1.065884

C -0.297384 2.480589 -0.570132

C -1.760942 2.833085 -0.312817

O 0.459054 3.658671 -0.279000

C -0.095709 2.117826 -2.041045

C 2.637008 1.193303 0.525226

C 3.845135 0.301859 0.622262

C 3.908674 -0.531221 -0.686535

C 2.676567 -1.426332 -0.763944

C 5.224884 0.967534 0.670673

C 6.178187 -0.125479 0.103018

C 5.276782 -1.214567 -0.548954

C 3.955744 0.378934 -1.930711

C 3.669521 -0.558916 1.894104

O 2.681224 2.390687 0.801593

C 0.387221 -3.305504 0.656569

H 1.314389 3.381895 0.094317

H -4.511506 1.200015 2.197874

H -4.872050 -0.719846 -1.630598

H -2.223230 -1.733904 -2.362081

H -3.492133 -2.841535 -1.871733

H -1.096907 -3.774378 -1.616785

H -2.104409 -3.867054 -0.166120

H 0.162412 1.765131 1.390742

H -0.746098 0.630583 0.417089

H -0.614029 -0.603733 -1.271403

H 0.383215 -1.896813 -1.911026

H -2.141575 -0.946018 3.542836

H -2.636818 0.757947 3.612543

H -1.132875 0.280989 2.791712

H -7.055499 -0.129727 -1.169413

H -6.124733 1.185636 -1.947330

H -7.573138 1.567365 -0.978273

H -1.918427 3.048126 0.749627

H -2.418367 2.003029 -0.595794

H -2.037075 3.718844 -0.893385

H -0.713526 1.262441 -2.333601

H 0.951640 1.874498 -2.244354

H -0.378242 2.972424 -2.663038

H 2.602311 -1.897705 -1.751870

H 2.770608 -2.252265 -0.046666

H 5.493400 1.267526 1.687316

H 5.239439 1.870993 0.054993

H 6.793902 -0.559824 0.895105

H 6.865989 0.306325 -0.628797

H 5.661011 -1.550766 -1.517059

H 5.203567 -2.097250 0.094571

H 3.083338 1.035050 -2.007757

H 3.981154 -0.247627 -2.827925

H 4.850351 1.006609 -1.942304

H 2.753136 -1.153370 1.886685

H 3.633447 0.102057 2.765788

H 4.521011 -1.232196 2.025698

H 1.006239 -3.843289 -0.067091

H -0.173261 -4.033627 1.248973

H 1.035114 -2.739593 1.332337

72

#4

C 4.450328 -0.018979 -1.529983

C 5.425860 -0.400041 -0.604179

C 5.063394 -0.648480 0.718526

C 3.738445 -0.525410 1.134798

C 2.771344 -0.151355 0.194519

C 3.115632 0.107319 -1.137901

C 3.346940 -0.801598 2.567895

C 1.932194 -0.304544 2.863076

C 0.973877 -0.631657 1.711633

O 1.447561 0.018811 0.520000

C -0.913186 1.940278 -0.278970

C -1.657711 0.640648 -0.077069

C -1.472175 -0.209135 0.965817

C -0.397224 0.009328 2.009653

C 2.049615 0.529468 -2.113258

O 6.751711 -0.549870 -0.904946

C 7.155128 -0.285582 -2.237854

C -1.651083 3.204747 0.224575

C -0.883992 4.440790 -0.248748

O -2.985397 3.257012 -0.289541

C -1.796087 3.221702 1.741961

C -2.646753 0.302031 -1.149272

C -3.225444 -1.088480 -1.117442

C -3.619959 -1.402753 0.348041

C -2.359282 -1.420554 1.203634

C -4.530501 -1.333412 -1.884693

C -5.168822 -2.546782 -1.146478

C -4.386647 -2.723165 0.187798

C -4.598829 -0.346681 0.903173

C -2.138906 -2.033655 -1.680650

O -2.917332 1.096787 -2.046657

C 0.887881 -2.134040 1.448340

H -2.998759 2.814917 -1.153045

H 4.706070 0.183119 -2.564279

H 5.832896 -0.938170 1.429755

H 4.061625 -0.320724 3.243065

H 3.419523 -1.878611 2.762448

H 1.938802 0.784394 2.992175

H 1.555011 -0.745512 3.791028

H -0.734746 2.068637 -1.353690

H 0.068879 1.895620 0.195099

H -0.219531 1.075734 2.165190

H -0.741990 -0.401185 2.965444

H 2.444399 0.557346 -3.131005

H 1.666709 1.524878 -1.864237

H 1.194799 -0.153926 -2.080933

H 6.934699 0.750403 -2.518381

H 6.664568 -0.966734 -2.942192

H 8.231672 -0.448625 -2.265991

H -0.846680 4.467082 -1.342748

H 0.142077 4.438900 0.133254

H -1.385648 5.347644 0.102565

H -0.818640 3.297520 2.228452

H -2.302318 2.316897 2.094026

H -2.395162 4.088673 2.036921

H -2.616611 -1.444247 2.270819

H -1.786479 -2.339075 1.020325

H -4.345551 -1.539506 -2.942464

H -5.176858 -0.452470 -1.837958

H -5.105744 -3.454350 -1.752768

H -6.230761 -2.366722 -0.959170

H -5.048902 -2.913586 1.038355

H -3.691512 -3.566982 0.124213

H -4.180542 0.665205 0.892650

H -4.841026 -0.599058 1.940688

H -5.537242 -0.330391 0.342399

H -1.929076 -1.752213 -2.717457

H -2.488792 -3.069747 -1.682420

H -1.199681 -1.982361 -1.124201

H 1.880570 -2.561051 1.284926

H 0.296977 -2.325735 0.549363

H 0.426415 -2.647965 2.297267

72

#5

C -4.964035 1.527471 -0.156475

C -5.789833 0.424908 0.104725

C -5.240533 -0.850592 0.154501

C -3.863556 -1.037575 -0.049582

C -3.056817 0.070233 -0.293221

C -3.600626 1.368147 -0.355276

C -3.264737 -2.422531 -0.005471

C -1.868862 -2.430048 -0.622459

C -1.037896 -1.238107 -0.134270

O -1.705050 -0.019578 -0.521801

C 2.704472 -2.007882 0.824516

C 2.432957 -0.605995 0.328962

C 1.351071 -0.246523 -0.410205

C 0.315108 -1.245985 -0.876556

C -2.697992 2.538138 -0.638394

O -7.114741 0.705524 0.290254

C -7.983901 -0.387171 0.533872

C 3.609772 -2.861090 -0.094856

C 3.720980 -4.268024 0.478024

O 2.992635 -3.019112 -1.378971

C 4.996012 -2.245258 -0.275917

C 3.431315 0.433145 0.757386

C 3.376147 1.749479 0.019262

C 1.892707 2.195990 -0.036769

C 1.093867 1.184066 -0.847458

C 4.070742 2.956445 0.660576

C 3.306115 4.177536 0.069093

C 2.014737 3.619455 -0.598097

C 1.291968 2.295852 1.381186

C 3.997474 1.509586 -1.375533

O 4.252519 0.204030 1.634829

C -0.867454 -1.238497 1.383051

H 2.938775 -2.149256 -1.799754

H -5.412068 2.516307 -0.201171

H -5.854668 -1.724759 0.344514

H -3.916161 -3.121910 -0.539126

H -3.221787 -2.772606 1.033658

H -1.942132 -2.364095 -1.714976

H -1.345404 -3.359983 -0.380529

H 3.190749 -1.935869 1.802670

H 1.768361 -2.554329 0.966879

H 0.094903 -1.045202 -1.933565

H 0.713253 -2.261002 -0.822906

H -1.919332 2.623538 0.126937

H -3.266515 3.469895 -0.661508

H -2.186889 2.415407 -1.599035

H -7.702877 -0.918135 1.450295

H -7.980811 -1.087529 -0.308911

H -8.979679 0.038204 0.650845

H 2.729344 -4.720933 0.571244

H 4.187419 -4.239721 1.466860

H 4.331739 -4.894327 -0.178533

H 5.610552 -2.903543 -0.896941

H 5.488008 -2.098386 0.689350

H 4.932120 -1.271397 -0.773800

H 0.019763 1.381904 -0.775716

H 1.340263 1.257466 -1.917708

H 5.139121 2.980971 0.428420

H 3.980115 2.917890 1.749790

H 3.919499 4.708729 -0.663754

H 3.063586 4.896951 0.855934

H 1.129997 4.222239 -0.367920

H 2.116089 3.595679 -1.688226

H 0.227522 2.540595 1.303556

H 1.767917 3.085246 1.968981

H 1.378394 1.360730 1.942760

H 3.985176 2.428005 -1.969373

H 3.488373 0.725880 -1.942644

H 5.044383 1.213446 -1.251104

H -1.834835 -1.133935 1.881647

H -0.231044 -0.405521 1.693551

H -0.409343 -2.174598 1.717121

72

#6

C -4.874379 0.564251 0.419369

C -5.237393 -0.164419 -0.717393

C -4.361958 -1.117587 -1.233485

C -3.129938 -1.364656 -0.628505

C -2.785355 -0.636901 0.517137

C -3.646753 0.334242 1.044434

C -2.165383 -2.373642 -1.201644

C -0.750985 -2.083967 -0.708335

C -0.727584 -1.881305 0.808475

O -1.592253 -0.791341 1.176780

C 2.858379 -1.653587 -0.783341

C 2.269258 -0.349894 -0.282219

C 1.273038 -0.260002 0.637730

C 0.670780 -1.472051 1.314617

C -3.231736 1.107824 2.267386

O -6.419020 -0.003427 -1.387206

C -7.312476 0.988452 -0.910315

C 4.257455 -2.071078 -0.251627

C 4.429611 -3.573591 -0.454273

O 5.292847 -1.456512 -1.020588

C 4.439567 -1.705025 1.220602

C 2.831219 0.894476 -0.887958

C 2.000343 2.141791 -0.758388

C 1.559782 2.258801 0.725179

C 0.676408 1.067950 1.072732

C 2.678045 3.490510 -1.023799

C 1.820769 4.497178 -0.200031

C 0.913693 3.651285 0.740772

C 2.772667 2.284815 1.676562

C 0.815370 1.987848 -1.741156

O 3.899860 0.881501 -1.496526

C -1.193274 -3.130441 1.556834

H 4.993466 -0.556762 -1.241956

H -5.533147 1.318118 0.836083

H -4.646952 -1.665950 -2.127592

H -2.200170 -2.335079 -2.294508

H -2.468330 -3.388684 -0.916623

H -0.371252 -1.176385 -1.192681

H -0.075236 -2.903970 -0.970823

H 2.936478 -1.610782 -1.877340

H 2.164287 -2.466213 -0.563368

H 1.319329 -2.346896 1.229402

H 0.569414 -1.255407 2.385083

H -3.058478 0.435735 3.113909

H -3.999357 1.832235 2.546943

H -2.292486 1.642749 2.091784

H -6.850611 1.981757 -0.939849

H -7.639035 0.767984 0.112143

H -8.172690 0.967354 -1.577991

H 4.230934 -3.841137 -1.497080

H 3.745997 -4.135216 0.189618

H 5.456320 -3.863022 -0.210953

H 3.637439 -2.120769 1.839920

H 4.440776 -0.618053 1.347149

H 5.396480 -2.094580 1.580505

H 0.474794 1.027266 2.150563

H -0.310130 1.164366 0.599487

H 2.693896 3.731725 -2.090199

H 3.716587 3.476169 -0.682039

H 1.215026 5.128193 -0.855727

H 2.464846 5.167698 0.375307

H 0.861101 4.064005 1.753207

H -0.110776 3.602037 0.356928

H 3.369431 1.369414 1.620835

H 2.411828 2.382747 2.705467

H 3.433453 3.132342 1.477100

H 0.192675 1.115739 -1.528840

H 1.209427 1.883739 -2.757058

H 0.181461 2.878505 -1.722431

H -2.246744 -3.336280 1.347359

H -1.083414 -2.982297 2.634711

H -0.603128 -4.001825 1.257708

72

#7

C -5.861505 -0.194309 0.785677

C -6.062395 -0.224592 -0.597182

C -4.963992 -0.200404 -1.454011

C -3.663440 -0.151060 -0.953483

C -3.476557 -0.131098 0.434902

C -4.568283 -0.149796 1.312009

C -2.474317 -0.117070 -1.884220

C -1.224847 0.335981 -1.134406

C -1.093779 -0.398298 0.202238

O -2.234572 -0.065272 1.021613

C 1.723510 2.041526 -0.695906

C 2.188172 0.675882 -0.251352

C 1.477863 -0.185246 0.520273

C 0.091510 0.128199 1.042613

C -4.330684 -0.120362 2.798025

O -7.292623 -0.271690 -1.193122

C -8.428875 -0.318271 -0.346624

C 2.353167 3.217192 0.089726

C 1.895629 3.223149 1.543187

O 3.777664 3.093136 0.141646

C 1.982158 4.531849 -0.596340

C 3.549780 0.290806 -0.754277

C 4.231109 -0.839625 -0.031537

C 3.209676 -1.993654 0.131474

C 2.049459 -1.513015 0.993218

C 5.416997 -1.525319 -0.719443

C 5.443802 -2.942434 -0.074214

C 4.097585 -3.115576 0.688395

C 2.660308 -2.456902 -1.233578

C 4.716885 -0.252893 1.315116

O 4.082245 0.898541 -1.677098

C -1.070086 -1.914708 0.018196

H 4.097512 2.829175 -0.733136

H -6.699490 -0.207849 1.473576

H -5.132801 -0.214595 -2.527685

H -2.679590 0.561093 -2.718118

H -2.318010 -1.109985 -2.323345

H -1.304140 1.408661 -0.922334

H -0.323840 0.175180 -1.735915

H 0.640816 2.140524 -0.618978

H 1.976929 2.163017 -1.756210

H -0.009237 -0.319520 2.039473

H -0.052282 1.205298 1.162675

H -3.785297 0.782318 3.090593

H -3.722804 -0.974448 3.112341

H -5.277305 -0.146424 3.341480

H -8.410248 -1.209384 0.290603

H -9.295298 -0.360379 -1.005262

H -8.491130 0.577943 0.280633

H 0.818604 3.405238 1.608292

H 2.413825 4.018892 2.086489

H 2.123529 2.267127 2.025655

H 2.379768 4.553813 -1.616607

H 0.895734 4.655749 -0.647411

H 2.404137 5.374515 -0.040607

H 1.258577 -2.267547 1.033724

H 2.372148 -1.373827 2.036176

H 6.349628 -0.977232 -0.559944

H 5.258268 -1.580756 -1.799911

H 6.290132 -3.047194 0.609893

H 5.562446 -3.712012 -0.841584

H 3.651353 -4.102462 0.530409

H 4.240286 -2.993930 1.767202

H 3.452156 -2.832605 -1.886908

H 2.130570 -1.660816 -1.767678

H 1.954932 -3.278953 -1.071694

H 3.907816 0.177952 1.910276

H 5.440013 0.542752 1.111168

H 5.221130 -1.019293 1.910116

H -2.017241 -2.263244 -0.401560

H -0.929220 -2.411501 0.981924

H -0.267162 -2.212715 -0.662995

72

#8

C -5.259361 0.894178 -1.051649

C -5.746721 -0.341860 -0.608275

C -5.035668 -1.076520 0.341881

C -3.846326 -0.553317 0.857279

C -3.373126 0.672641 0.400950

C -4.065298 1.417681 -0.562871

C -2.957141 -1.255085 1.843857

C -1.522123 -1.136033 1.315138

C -1.094597 0.339176 1.183539

O -2.238959 1.206503 0.961392

C 2.373593 2.044447 0.733418

C 2.322325 0.591328 0.304821

C 1.183020 -0.076481 -0.018808

C -0.171934 0.596459 -0.035258

C -3.519084 2.739088 -1.030857

O -6.923180 -0.750748 -1.170371

C -7.451835 -1.996561 -0.748405

C 2.877392 3.091349 -0.299368

C 2.374295 4.469513 0.120166

O 4.303136 3.177251 -0.278959

C 2.414552 2.765116 -1.719103

C 3.636009 -0.119256 0.262978

C 3.591444 -1.622468 0.251426

C 2.562146 -2.049001 -0.828795

C 1.183558 -1.543520 -0.422731

C 4.859097 -2.375106 -0.169887

C 4.315555 -3.735387 -0.698876

C 2.777536 -3.568459 -0.874527

C 2.923475 -1.477097 -2.213811

C 3.206776 -2.067414 1.681331

O 4.696466 0.502690 0.273511

C -0.478430 0.840176 2.482862

H 4.644280 2.275113 -0.145896

H -5.830182 1.439125 -1.798429

H -5.382921 -2.041376 0.694756

H -3.242635 -2.304001 1.956795

H -3.035933 -0.782131 2.831564

H -1.484757 -1.632658 0.339723

H -0.815597 -1.649352 1.975870

H 3.023163 2.131704 1.613807

H 1.381067 2.354456 1.060276

H -0.070842 1.680570 -0.118545

H -0.720538 0.259675 -0.924947

H -2.489007 2.627607 -1.383701

H -3.498593 3.466774 -0.213381

H -4.127113 3.145485 -1.841571

H -6.762223 -2.816100 -0.979305

H -8.379460 -2.134957 -1.302239

H -7.665151 -1.990454 0.326242

H 2.648373 4.674181 1.160056

H 1.286057 4.533096 0.024464

H 2.826382 5.235802 -0.516608

H 1.327296 2.642589 -1.770340

H 2.880742 1.840394 -2.072567

H 2.706541 3.575305 -2.394062

H 0.462514 -1.684829 -1.237562

H 0.804997 -2.139043 0.418775

H 5.553089 -2.499407 0.665867

H 5.392287 -1.826634 -0.951094

H 4.537456 -4.546096 0.000116

H 4.793517 -3.997010 -1.646630

H 2.414391 -4.000768 -1.812136

H 2.235213 -4.059732 -0.060090

H 2.948215 -0.383502 -2.225926

H 2.167433 -1.799650 -2.936895

H 3.891814 -1.840292 -2.567312

H 2.247554 -1.663055 2.013997

H 3.976887 -1.721348 2.377973

H 3.167053 -3.157966 1.748474

H -1.130441 0.596380 3.326868

H -0.353168 1.926050 2.449262

H 0.497755 0.374964 2.651216

### **Conformers coordinates -*RSR* (XYZ format)**

72

#1

C -4.160245 -0.389808 1.184949

C -4.809589 -0.165740 -0.033104

C -4.276907 0.748075 -0.940280

C -3.088948 1.425584 -0.665200

C -2.430410 1.163645 0.542559

C -2.969253 0.274549 1.483650

C -2.528289 2.440578 -1.634118

C -1.475180 3.303971 -0.943046

C -0.498658 2.446608 -0.129824

O -1.236116 1.746822 0.890370

C 0.007029 -1.286772 0.298730

C 1.301252 -0.555613 0.045537

C 1.397186 0.670063 -0.528356

C 0.200372 1.423308 -1.066981

C -2.262402 0.058480 2.794448

O -5.969196 -0.784189 -0.409255

C -6.497417 -1.769702 0.462583

C -0.340224 -2.406920 -0.706178

C -0.607128 -1.855771 -2.101497

O 0.756918 -3.314668 -0.855304

C -1.567844 -3.159968 -0.191956

C 2.533467 -1.274458 0.505536

C 3.846301 -0.752786 -0.016853

C 3.846375 0.791976 0.102281

C 2.732092 1.354374 -0.770756

C 5.127598 -1.152058 0.723700

C 6.130466 -0.020753 0.350422

C 5.294517 1.134852 -0.275606

C 3.621643 1.240204 1.561314

C 3.945640 -1.260366 -1.475648

O 2.462406 -2.271057 1.218386

C 0.471503 3.325287 0.642082

H 1.162483 -3.451650 0.013604

H -4.561079 -1.083898 1.915534

H -4.803928 0.933474 -1.872704

H -3.340011 3.067116 -2.016245

H -2.096782 1.929301 -2.503949

H -1.962497 3.997916 -0.247617

H -0.916893 3.901109 -1.670228

H -0.831415 -0.591914 0.321221

H 0.065851 -1.742525 1.295078

H 0.532457 1.981738 -1.952364

H -0.559557 0.718669 -1.411093

H -1.212080 -0.204296 2.636302

H -2.743708 -0.737377 3.366617

H -2.271592 0.973222 3.396488

H -5.776263 -2.579109 0.622665

H -7.385432 -2.164286 -0.029532

H -6.778935 -1.334436 1.427875

H -0.782609 -2.683810 -2.794927

H 0.250201 -1.277446 -2.461207

H -1.496234 -1.215507 -2.097636

H -1.356059 -3.617069 0.780796

H -1.845770 -3.950824 -0.895427

H -2.418368 -2.478439 -0.073533

H 2.623940 2.433685 -0.619243

H 2.976051 1.231111 -1.836911

H 5.482133 -2.140214 0.418011

H 4.953211 -1.192361 1.802602

H 6.880100 -0.381225 -0.359031

H 6.673361 0.320274 1.235974

H 5.590224 2.117315 0.105745

H 5.414926 1.161465 -1.363593

H 2.677285 0.872754 1.975824

H 4.430029 0.907322 2.217493

H 3.601173 2.334648 1.598805

H 3.101998 -0.947520 -2.095958

H 3.964555 -2.354629 -1.464232

H 4.872172 -0.915303 -1.942854

H -0.092809 4.006793 1.284442

H 1.083260 3.919207 -0.042114

H 1.126832 2.716431 1.271442

72

#2

C -4.484385 0.203807 -1.511515

C -5.453626 -0.314558 -0.647980

C -5.082124 -0.757411 0.620095

C -3.754687 -0.695022 1.042184

C -2.793483 -0.179473 0.164179

C -3.147640 0.275129 -1.111995

C -3.352894 -1.183767 2.414125

C -1.947010 -0.709379 2.778423

C -0.984376 -0.858065 1.594608

O -1.467924 -0.053750 0.503622

C 0.885089 1.913244 -0.098815

C 1.691604 0.643231 0.023607

C 1.475463 -0.331792 0.941068

C 0.379281 -0.249348 1.981564

C -2.091329 0.844824 -2.020256

O -6.780820 -0.422399 -0.960445

C -7.190617 0.021630 -2.242834

C 1.570157 3.168327 0.487675

C 1.731071 3.074750 1.999946

O 2.899934 3.306869 -0.026844

C 0.750895 4.403745 0.112382

C 2.790596 0.494808 -0.984934

C 3.852845 -0.525245 -0.667321

C 3.153546 -1.834383 -0.218003

C 2.360977 -1.561148 1.054067

C 4.766005 -0.998418 -1.803871

C 5.250126 -2.398991 -1.323708

C 4.341014 -2.802555 -0.125320

C 2.193769 -2.358986 -1.305513

C 4.733276 0.113628 0.433229

O 2.838442 1.204305 -1.985273

C -0.883228 -2.305410 1.116692

H 2.888912 3.081009 -0.968336

H -4.747488 0.556611 -2.502604

H -5.845528 -1.153001 1.285163

H -3.401992 -2.279448 2.438187

H -4.073734 -0.827987 3.156804

H -1.562271 -1.269447 3.636346

H -1.971607 0.350715 3.058338

H -0.095370 1.796557 0.365132

H 0.706249 2.098195 -1.165395

H 0.719893 -0.777113 2.881062

H 0.196302 0.789241 2.269610

H -1.725656 1.803762 -1.637769

H -2.488612 1.003735 -3.024907

H -1.224932 0.178538 -2.079439

H -6.693525 -0.547678 -3.036085

H -6.983441 1.089395 -2.375793

H -8.265243 -0.148920 -2.292801

H 2.277224 2.167211 2.276925

H 0.754134 3.066068 2.492933

H 2.292313 3.941750 2.361531

H 0.701847 4.510727 -0.976521

H 1.215514 5.302353 0.529344

H -0.271116 4.327566 0.497347

H 1.754025 -2.429905 1.331194

H 3.039840 -1.397465 1.904948

H 5.595184 -0.305642 -1.971510

H 4.211034 -1.067999 -2.743443

H 6.299490 -2.369143 -1.018353

H 5.178652 -3.129416 -2.134103

H 4.018467 -3.847259 -0.178488

H 4.867435 -2.674179 0.826179

H 1.397784 -1.645308 -1.543482

H 1.722932 -3.281227 -0.948429

H 2.720268 -2.600090 -2.232516

H 5.541721 -0.562720 0.724558

H 4.168895 0.390519 1.326953

H 5.187815 1.024878 0.032085

H -1.866371 -2.686758 0.828672

H -0.484397 -2.948317 1.907523

H -0.233591 -2.369007 0.239979

72

#3

C 4.719112 0.578829 0.326552

C 5.092580 -0.231048 -0.749819

C 4.226866 -1.228361 -1.194190

C 2.993165 -1.435732 -0.578050

C 2.634805 -0.621616 0.502801

C 3.488779 0.390461 0.960696

C 2.047939 -2.508782 -1.059031

C 0.627493 -2.234538 -0.569704

C 0.609875 -1.884765 0.921008

O 1.433439 -0.727707 1.154982

C -2.778740 -1.600285 -0.894606

C -2.240414 -0.320569 -0.299076

C -1.384905 -0.261768 0.755492

C -0.802231 -1.492257 1.417223

C 3.069201 1.257696 2.117623

O 6.276929 -0.114577 -1.423660

C 7.174009 0.898181 -1.000768

C -4.134795 -2.050327 -0.300854

C -4.528336 -3.390857 -0.907217

O -3.990922 -2.294447 1.104271

C -5.239055 -1.017735 -0.522811

C -2.663763 0.941595 -0.998429

C -2.417019 2.235940 -0.262250

C -0.993053 2.190681 0.350006

C -0.928797 1.054792 1.361250

C -2.390791 3.532373 -1.079219

C -1.543403 4.499209 -0.200101

C -0.829123 3.624518 0.872359

C 0.080304 1.956454 -0.736290

C -3.537663 2.344469 0.798033

O -3.199338 0.904994 -2.098121

C 1.142549 -3.034262 1.776538

H -3.740322 -1.463149 1.531814

H 5.372796 1.363709 0.690556

H 4.522382 -1.844952 -2.039140

H 2.389014 -3.488769 -0.702905

H 2.067128 -2.553789 -2.152134

H -0.004360 -3.111841 -0.737931

H 0.195690 -1.398034 -1.132139

H -2.075373 -2.422957 -0.753904

H -2.907172 -1.458432 -1.972269

H -0.720204 -1.295554 2.493828

H -1.452100 -2.362554 1.296318

H 2.773129 0.649481 2.977532

H 2.204215 1.876957 1.854020

H 3.882905 1.920853 2.418264

H 7.488851 0.739096 0.036698

H 6.721608 1.891433 -1.097213

H 8.040127 0.828663 -1.657571

H -3.752000 -4.137968 -0.716559

H -4.664193 -3.294731 -1.988156

H -5.466756 -3.741629 -0.467746

H -5.365869 -0.802927 -1.587508

H -5.008233 -0.076035 -0.012880

H -6.181690 -1.398878 -0.119149

H 0.081105 0.932203 1.757488

H -1.575384 1.269278 2.225297

H -3.396840 3.915689 -1.270763

H -1.919862 3.362681 -2.051873

H -2.176916 5.252186 0.276512

H -0.818583 5.041454 -0.813327

H 0.225923 3.891871 0.992572

H -1.304086 3.737928 1.852498

H 0.164109 2.811626 -1.412633

H -0.116823 1.065722 -1.341385

H 1.054650 1.813693 -0.254820

H -3.572024 1.485034 1.472917

H -4.503392 2.411478 0.286197

H -3.419194 3.249981 1.399810

H 0.563924 -3.945581 1.597500

H 1.074127 -2.774203 2.836341

H 2.191292 -3.238916 1.543075

72

#4

C 4.515020 1.073099 -0.280824

C 5.006782 -0.091762 -0.876298

C 4.396486 -1.314605 -0.604746

C 3.293160 -1.394256 0.243821

C 2.794167 -0.214541 0.813046

C 3.405956 1.021932 0.567497

C 2.649354 -2.726312 0.553233

C 1.722164 -2.608882 1.760327

C 0.853733 -1.350592 1.666548

O 1.716588 -0.198964 1.667161

C -2.994798 -1.925929 0.303373

C -2.368605 -0.551214 0.225246

C -1.034049 -0.300269 0.251221

C 0.002546 -1.388583 0.372390

C 2.878254 2.269768 1.225223

O 6.079605 -0.127047 -1.722862

C 6.719460 1.102357 -2.021530

C -3.311353 -2.592764 -1.058038

C -4.107567 -3.874242 -0.806455

O -4.087334 -1.712212 -1.875891

C -2.051457 -2.899391 -1.859879

C -3.342026 0.581782 0.118831

C -2.794913 1.968945 0.333035

C -1.462630 2.087369 -0.451746

C -0.461680 1.099034 0.130389

C -3.610563 3.151798 -0.202844

C -2.543786 4.269716 -0.400191

C -1.149074 3.582533 -0.306646

C -1.664884 1.786055 -1.951325

C -2.632393 2.140811 1.860956

O -4.538091 0.378996 -0.077751

C -0.017642 -1.198323 2.902456

H -4.594591 -1.120207 -1.297815

H 4.981646 2.035140 -0.461224

H 4.800058 -2.216819 -1.057293

H 3.426036 -3.473490 0.742349

H 2.091087 -3.078257 -0.323579

H 2.313892 -2.530683 2.680108

H 1.083040 -3.492522 1.851201

H -3.940020 -1.839102 0.852587

H -2.366799 -2.609703 0.880698

H -0.450334 -2.379833 0.316405

H 0.678419 -1.302505 -0.489186

H 2.792156 2.137275 2.307751

H 1.879249 2.519819 0.854073

H 3.538473 3.116736 1.027249

H 7.131659 1.562478 -1.116608

H 6.026785 1.801042 -2.503750

H 7.530180 0.862090 -2.708111

H -5.044535 -3.643269 -0.289257

H -3.536659 -4.579404 -0.193772

H -4.347104 -4.355446 -1.759457

H -1.460254 -1.990934 -2.013109

H -2.337544 -3.293870 -2.839688

H -1.433579 -3.648424 -1.355138

H 0.452166 1.052230 -0.478220

H -0.127875 1.422082 1.126078

H -4.401112 3.448344 0.491882

H -4.094679 2.890385 -1.147928

H -2.643271 5.046103 0.362977

H -2.676377 4.760607 -1.367987

H -0.458408 3.929398 -1.081968

H -0.677499 3.784837 0.661049

H -2.329769 2.513512 -2.424455

H -2.073833 0.786558 -2.132072

H -0.696150 1.848483 -2.458029

H -1.982260 1.386417 2.309699

H -3.617222 2.057504 2.331152

H -2.232915 3.130224 2.100052

H -0.536701 -0.235874 2.894752

H 0.597437 -1.253341 3.804637

H -0.766920 -1.994966 2.936285

72

#5

C 4.314066 1.220787 0.313924

C 4.917421 0.304384 -0.559013

C 4.375980 -0.966942 -0.703263

C 3.213248 -1.326270 -0.001534

C 2.599853 -0.386673 0.823541

C 3.163719 0.891766 1.015805

C 2.663446 -2.729683 -0.107112

C 1.654756 -2.996356 1.007557

C 0.692047 -1.818556 1.192637

O 1.450858 -0.642086 1.532302

C -3.104740 -1.651835 0.477126

C -2.315903 -0.388205 0.221979

C -0.997769 -0.360797 -0.109113

C -0.119718 -1.591363 -0.113492

C 2.548107 1.846892 2.003621

O 6.039329 0.746249 -1.202164

C 6.693818 -0.165258 -2.068013

C -3.743189 -2.278615 -0.787498

C -4.786854 -1.367255 -1.430117

O -2.725834 -2.585544 -1.748312

C -4.365193 -3.619386 -0.417550

C -3.067023 0.897149 0.403689

C -2.233299 2.156188 0.411980

C -1.219397 2.082457 -0.756034

C -0.275768 0.912261 -0.510257

C -2.952333 3.487657 0.163226

C -1.827538 4.413687 -0.387654

C -0.619109 3.495842 -0.735951

C -1.931147 1.891068 -2.110244

C -1.555643 2.221230 1.801943

O -4.270282 0.908211 0.627025

C -0.207004 -2.049454 2.397240

H -2.315107 -1.756265 -2.031852

H 4.771715 2.197851 0.441840

H 4.839268 -1.710184 -1.343865

H 3.488580 -3.445980 -0.041781

H 2.204592 -2.882738 -1.091248

H 2.181732 -3.140142 1.958446

H 1.082012 -3.906731 0.806518

H -3.908765 -1.430851 1.186121

H -2.469101 -2.414896 0.931068

H -0.702439 -2.499021 -0.291119

H 0.584277 -1.497067 -0.947053

H 3.125380 2.772234 2.053427

H 2.514736 1.400104 3.002454

H 1.515680 2.098542 1.740853

H 7.032103 -1.053132 -1.522261

H 7.556160 0.364521 -2.470754

H 6.035401 -0.470526 -2.889068

H -4.344120 -0.419251 -1.752953

H -5.587504 -1.134563 -0.723215

H -5.212727 -1.865439 -2.306262

H -5.147612 -3.482916 0.334157

H -3.603773 -4.292876 -0.012712

H -4.808657 -4.084920 -1.302658

H 0.320457 0.694389 -1.407071

H 0.451589 1.151898 0.272989

H -3.404512 3.880676 1.078037

H -3.758924 3.361475 -0.564673

H -1.539365 5.165850 0.351691

H -2.174744 4.957549 -1.270287

H -0.159269 3.756175 -1.694628

H 0.162540 3.572535 0.027609

H -2.599238 2.724667 -2.342067

H -2.518483 0.968744 -2.150963

H -1.178823 1.841024 -2.904033

H -0.907264 1.364089 2.004011

H -2.332059 2.248229 2.573266

H -0.963130 3.136245 1.899051

H -0.905788 -1.218611 2.533038

H 0.409512 -2.138558 3.295813

H -0.775888 -2.975101 2.274773

72

#6

C -4.780312 1.153972 -0.962408

C -5.642456 0.323176 -0.235138

C -5.126084 -0.683901 0.582690

C -3.740294 -0.841506 0.674632

C -2.901207 -0.017862 -0.066874

C -3.398962 0.993894 -0.896854

C -3.040239 -1.887684 1.495242

C -1.972755 -2.535664 0.605372

C -0.987018 -1.488631 0.033621

O -1.542113 -0.148642 0.078200

C 2.688978 -1.861569 -0.859009

C 2.312649 -0.527321 -0.245646

C 1.248417 -0.329762 0.576604

C 0.272784 -1.431570 0.928652

C -2.447503 1.867042 -1.668298

O -6.976756 0.571650 -0.393941

C -7.885821 -0.248912 0.320299

C 3.908178 -2.610101 -0.253624

C 3.826913 -4.079423 -0.657787

O 5.131669 -2.123411 -0.807188

C 3.954567 -2.478672 1.268359

C 3.167602 0.634025 -0.627552

C 2.573429 1.997862 -0.400290

C 2.060052 2.030594 1.064163

C 0.935526 1.013553 1.212693

C 3.511893 3.207018 -0.467714

C 2.814708 4.263656 0.439115

C 1.700176 3.514844 1.226125

C 3.184686 1.700380 2.065437

C 1.445670 2.184701 -1.442994

O 4.273567 0.477168 -1.142804

C -0.675054 -1.789758 -1.428260

H 5.028232 -1.165189 -0.948386

H -5.213374 1.926203 -1.592275

H -5.771228 -1.341010 1.155431

H -3.743057 -2.637275 1.867695

H -2.563157 -1.419466 2.366952

H -2.479555 -3.047037 -0.220107

H -1.407765 -3.293823 1.156681

H 2.896686 -1.723606 -1.928184

H 1.834144 -2.535863 -0.802580

H 0.746441 -2.415858 0.887642

H -0.070182 -1.285558 1.960191

H -2.990469 2.551159 -2.323671

H -1.770957 1.256501 -2.275192

H -1.820701 2.457377 -0.990903

H -7.780176 -1.300043 0.029561

H -8.882205 0.102875 0.056277

H -7.738065 -0.152129 1.401674

H 3.721866 -4.169675 -1.743732

H 2.973455 -4.569249 -0.179323

H 4.742087 -4.595741 -0.352792

H 3.012513 -2.799953 1.726395

H 4.138972 -1.439949 1.558287

H 4.764926 -3.097068 1.665867

H 0.703423 0.841413 2.272090

H 0.005905 1.384612 0.764349

H 3.642212 3.559806 -1.494633

H 4.504418 2.948116 -0.089163

H 2.387555 5.074756 -0.156649

H 3.537347 4.720449 1.120745

H 1.660937 3.811216 2.279140

H 0.713933 3.717848 0.795127

H 4.016404 2.406929 1.998407

H 3.586695 0.691968 1.928347

H 2.781944 1.760201 3.081586

H 0.649236 1.442242 -1.346735

H 1.871882 2.100843 -2.448098

H 1.003947 3.181414 -1.353770

H 0.071864 -1.095099 -1.823936

H -1.596009 -1.685128 -2.011974

H -0.312191 -2.815617 -1.547803

72

#7

C 4.925916 1.531062 0.066364

C 5.755981 0.413470 0.234179

C 5.220286 -0.865018 0.134274

C 3.852819 -1.040295 -0.134277

C 3.042986 0.080808 -0.290473

C 3.571412 1.382783 -0.192626

C 3.264057 -2.425753 -0.253753

C 1.894709 -2.374445 -0.925886

C 1.034598 -1.246974 -0.341252

O 1.702239 0.006789 -0.577054

C -3.241316 -1.909803 -0.921586

C -2.659250 -0.564422 -0.550990

C -1.350852 -0.211631 -0.658606

C -0.291474 -1.189307 -1.122061

C 2.659708 2.566185 -0.373452

O 7.072011 0.683361 0.486686

C 7.942083 -0.422645 0.654936

C -3.588894 -2.839999 0.262224

C -2.345432 -3.241259 1.043248

O -4.424675 -2.161528 1.205727

C -4.319710 -4.074130 -0.266057

C -3.678251 0.452424 -0.127636

C -3.141346 1.718093 0.484955

C -1.997280 2.230303 -0.425841

C -0.866423 1.210751 -0.433792

C -4.079216 2.927559 0.575253

C -3.107074 4.144481 0.594467

C -1.704155 3.605887 0.189323

C -2.485978 2.448394 -1.872269

C -2.674997 1.350842 1.912944

O -4.881745 0.238667 -0.241640

C 0.824841 -1.427445 1.162100

H -5.036397 -1.588059 0.720726

H 5.363808 2.522243 0.146371

H 5.838251 -1.749198 0.251201

H 3.179694 -2.877533 0.742813

H 3.941492 -3.066310 -0.827095

H 1.372874 -3.329424 -0.809698

H 2.012582 -2.187221 -2.000159

H -2.584246 -2.455047 -1.602538

H -4.168926 -1.721831 -1.476017

H -0.686117 -2.206709 -1.126743

H -0.033695 -0.937227 -2.161070

H 3.210827 3.502037 -0.261405

H 1.846317 2.545650 0.359493

H 2.191363 2.551400 -1.363360

H 7.967199 -1.044744 -0.246668

H 7.640230 -1.034678 1.512254

H 8.931483 -0.004693 0.836061

H -1.840334 -2.349260 1.424430

H -1.649366 -3.804090 0.412396

H -2.628000 -3.869888 1.893091

H -4.584582 -4.732713 0.566435

H -3.692312 -4.633681 -0.967305

H -5.239664 -3.781039 -0.782663

H -0.132575 1.447152 -1.212584

H -0.297723 1.249321 0.505729

H -4.710746 2.884672 1.466997

H -4.747876 2.963174 -0.289291

H -3.071683 4.604803 1.585566

H -3.446817 4.917731 -0.099859

H -1.190185 4.266257 -0.516770

H -1.055967 3.503573 1.066404

H -1.649695 2.815822 -2.475881

H -3.283870 3.193657 -1.928491

H -2.850551 1.525988 -2.335329

H -1.913463 0.567342 1.924090

H -3.536078 0.989494 2.484332

H -2.279393 2.230615 2.428329

H 0.096209 -0.709228 1.548596

H 1.764753 -1.278346 1.700669

H 0.464049 -2.437831 1.374922

72

#8

C -4.761568 1.500943 -0.251408

C -5.649084 0.415976 -0.279118

C -5.174041 -0.869041 -0.047105

C -3.810240 -1.084104 0.211557

C -2.942301 0.004666 0.226285

C -3.410018 1.313352 -0.002771

C -3.291141 -2.478068 0.473244

C -1.906762 -2.424808 1.112837

C -1.007345 -1.418513 0.388131

O -1.597623 -0.108251 0.488986

C 2.605030 -1.960270 -0.800461

C 2.304949 -0.572509 -0.269394

C 1.301413 -0.283465 0.600242

C 0.344763 -1.329984 1.127895

C -2.444138 2.466759 0.031499

O -6.955202 0.724586 -0.536495

C -7.886940 -0.343287 -0.552634

C 3.829249 -2.708046 -0.205105

C 3.687167 -4.196683 -0.509889

O 5.037194 -2.294008 -0.845212

C 3.955677 -2.482909 1.301335

C 3.167883 0.529827 -0.787709

C 2.630688 1.927065 -0.630330

C 2.186764 2.083564 0.848616

C 1.043346 1.117272 1.125118

C 3.603046 3.095642 -0.824490

C 2.976822 4.238462 0.028759

C 1.877056 3.586071 0.915957

C 3.347059 1.791544 1.820774

C 1.467286 2.081957 -1.638173

O 4.236568 0.297667 -1.350496

C -0.843303 -1.763362 -1.091382

H 4.947521 -1.347720 -1.057753

H -5.152952 2.498661 -0.430156

H -5.836988 -1.727750 -0.052506

H -3.253620 -3.041003 -0.467903

H -3.987804 -3.013814 1.125699

H -1.433922 -3.411563 1.099179

H -1.992217 -2.109579 2.159636

H 2.761903 -1.904421 -1.885489

H 1.730690 -2.595846 -0.654112

H 0.788915 -2.328302 1.103906

H 0.123254 -1.103832 2.176968

H -1.640558 2.324273 -0.699414

H -1.970004 2.552220 1.015263

H -2.954685 3.406036 -0.190367

H -7.641402 -1.068943 -1.336240

H -8.857522 0.104528 -0.762102

H -7.920441 -0.851587 0.417678

H 3.529775 -4.351477 -1.582083

H 2.842890 -4.628775 0.035871

H 4.600096 -4.721558 -0.212995

H 3.034426 -2.757581 1.826550

H 4.172044 -1.432224 1.516038

H 4.775363 -3.091394 1.695044

H 0.840204 1.049678 2.201881

H 0.109439 1.473005 0.673816

H 3.701504 3.368127 -1.879003

H 4.601931 2.831347 -0.467050

H 2.547697 5.016173 -0.608566

H 3.741656 4.721139 0.643111

H 1.894098 3.958891 1.944960

H 0.878117 3.789270 0.514444

H 3.721555 0.767107 1.733373

H 2.990364 1.926762 2.846854

H 4.189280 2.472536 1.672332

H 1.852506 1.929511 -2.651490

H 1.052170 3.092780 -1.591759

H 0.658610 1.365519 -1.472047

H -0.507979 -2.798449 -1.210535

H -0.115003 -1.097839 -1.563190

H -1.796317 -1.652174 -1.615768

72

#9

C -4.058105 0.065237 -1.539140

C -4.993321 0.460404 -0.577645

C -4.649901 0.433385 0.772522

C -3.385862 0.011341 1.184627

C -2.464860 -0.392760 0.211384

C -2.787664 -0.365884 -1.151309

C -3.004396 -0.003763 2.645964

C -1.486920 -0.078183 2.806109

C -0.881161 -1.131373 1.871706

O -1.189334 -0.800667 0.508070

C 1.602803 -1.897570 -0.800069

C 1.803777 -0.525765 -0.189047

C 1.365318 -0.171819 1.045824

C 0.669751 -1.161797 1.952618

C -1.759884 -0.802840 -2.160280

O -6.258405 0.891513 -0.869632

C -6.635249 0.937899 -2.235118

C 2.812826 -2.866965 -0.825012

C 2.299471 -4.279448 -1.090183

O 3.684446 -2.552758 -1.914327

C 3.616396 -2.830554 0.474930

C 2.468916 0.487749 -1.055122

C 2.240161 1.928560 -0.689114

C 2.521435 2.073917 0.831342

C 1.506891 1.234232 1.598385

C 3.163358 2.981881 -1.310651

C 3.126445 4.153498 -0.286131

C 2.464948 3.597824 1.008538

C 3.944232 1.601628 1.191531

C 0.777979 2.255077 -1.076605

O 3.126608 0.158244 -2.041590

C -1.428409 -2.526887 2.173077

H 3.640894 -1.590825 -2.062774

H -4.298123 0.083394 -2.596545

H -5.381900 0.757204 1.508159

H -3.482994 -0.854680 3.145469

H -3.390164 0.896399 3.134138

H -1.217276 -0.319531 3.838886

H -1.050299 0.897776 2.573138

H 1.282536 -1.772817 -1.842835

H 0.770426 -2.388824 -0.291808

H 1.004767 -2.174811 1.724133

H 0.953168 -0.967123 2.992963

H -2.149809 -0.714052 -3.176328

H -1.460802 -1.841723 -1.985557

H -0.849511 -0.198831 -2.077859

H -6.587153 -0.056984 -2.691871

H -5.997337 1.628455 -2.798097

H -7.663552 1.296461 -2.253370

H 1.684335 -4.295648 -1.995526

H 1.697677 -4.638660 -0.249509

H 3.144521 -4.960131 -1.231671

H 2.994000 -3.075673 1.341590

H 4.052894 -1.839638 0.632855

H 4.429477 -3.560481 0.417510

H 1.762765 1.178198 2.664066

H 0.519951 1.715891 1.549492

H 2.819780 3.285103 -2.303546

H 4.175264 2.584685 -1.428816

H 2.555853 5.000308 -0.676761

H 4.136653 4.520342 -0.085337

H 2.985957 3.919923 1.915630

H 1.427085 3.936898 1.092602

H 4.095029 1.722908 2.269085

H 4.712500 2.190769 0.684052

H 4.111150 0.547728 0.948994

H 0.537641 3.295284 -0.838646

H 0.665539 2.130618 -2.158927

H 0.049295 1.603610 -0.585055

H -1.168185 -2.822825 3.194312

H -0.996318 -3.251613 1.476927

H -2.516105 -2.559887 2.067873

72

#10

C 5.356173 0.435909 -0.392757

C 5.338408 -0.901097 -0.800577

C 4.275377 -1.721998 -0.428749

C 3.224441 -1.229055 0.343195

C 3.249960 0.114386 0.735938

C 4.311312 0.954121 0.375994

C 2.071076 -2.120105 0.741199

C 1.236683 -1.467664 1.841263

C 0.989018 0.018528 1.565202

O 2.269494 0.679751 1.516133

C -2.014646 2.267348 0.588125

C -2.184205 0.806940 0.240121

C -1.160948 -0.072609 0.074844

C 0.290238 0.334105 0.210686

C 4.307593 2.391657 0.822355

O 6.315919 -1.485769 -1.557345

C 7.410793 -0.677021 -1.953418

C -1.990086 3.220711 -0.630371

C -1.697018 4.636042 -0.149445

O -0.900222 2.880343 -1.496754

C -3.294532 3.185767 -1.424788

C -3.609623 0.339413 0.125577

C -3.823038 -0.978945 -0.576156

C -2.807135 -1.996345 0.004954

C -1.394378 -1.524212 -0.312111

C -5.166923 -1.688172 -0.375579

C -4.833159 -3.185314 -0.642726

C -3.280712 -3.309803 -0.633950

C -2.956716 -2.132467 1.534263

C -3.629403 -0.717182 -2.087266

O -4.533980 1.012493 0.559290

C 0.271537 0.665708 2.740054

H -1.056522 1.992740 -1.849219

H 6.173272 1.094425 -0.665708

H 4.276531 -2.761875 -0.745598

H 2.453720 -3.086738 1.083117

H 1.459255 -2.329194 -0.145425

H 1.782545 -1.521676 2.790783

H 0.287556 -1.989869 1.994680

H -1.092089 2.429439 1.147083

H -2.846157 2.566272 1.234245

H 0.857156 -0.157633 -0.590830

H 0.402493 1.409374 0.055454

H 4.314345 2.464063 1.914561

H 3.404903 2.904546 0.475987

H 5.181211 2.919199 0.434223

H 7.959006 -0.301418 -1.082111

H 7.077510 0.167617 -2.566617

H 8.062781 -1.319164 -2.544002

H -0.737694 4.667934 0.375842

H -2.481086 4.977821 0.532021

H -1.652139 5.320305 -1.001707

H -4.148626 3.424934 -0.785432

H -3.465540 2.195462 -1.860587

H -3.243093 3.911007 -2.242128

H -0.660384 -2.164585 0.184872

H -1.181988 -1.619888 -1.387646

H -5.933091 -1.304474 -1.054815

H -5.535508 -1.535253 0.642517

H -5.240037 -3.513530 -1.602886

H -5.282745 -3.822527 0.123533

H -2.935619 -4.181666 -0.069269

H -2.888227 -3.408413 -1.651433

H -3.940079 -2.519412 1.813830

H -2.804650 -1.185029 2.060876

H -2.209853 -2.843839 1.902741

H -2.649901 -0.295557 -2.328616

H -4.395215 -0.012002 -2.426353

H -3.756587 -1.639889 -2.659931

H 0.779879 0.405765 3.672621

H -0.765441 0.322429 2.797450

H 0.279909 1.753887 2.638022

**Multi Tensor Multi Conformer Analysis (MTMC)**

Discrimination of both epimers (**1a** and **1b**) was further confirmed by an RDC-based Multi Tensor Multi Conformer (MTMC) analysis using a protocol inspired by the one described by Griesinger et al.^[10]^ The RDC-based MTMC analysis protocol implemented is shown in **Figure S93**. It corroborates our initial findings in the assignment of the relative configuration of **1a** and **1b**, as can be seen in **Figure S94**. A detailed description of the protocol and its implementation is in the lines below.

**Figure S93.** RDC-based multiple-tensor multi-conformer analysis workflow used for configurational assignment of Meroditerpene**-1a** and **-1b**. Conformers after DFT optimization are selected that show the least violations of experimental isotropic NMR parameters based on CASE approach.

|  |  |
| --- | --- |

**Figure S94.** Cornilescu’s quality factor (Q) analyses of the MTMC RDC-based analysis for **1a** (right) and **1b** (left).

**Relative configuration assignment of meroditerpene-1a and -1b based on MTMC analysis.**

Optimized conformers within an energetic window, where the thermal contribution was considered, of 5.1 Kcal mol^-1^ were experimentally constrained, using a CASE approach, together with isotropic constraints of both epimers **1a** and **1b** independently.^[11]^ Constrains used include ^1^H and ^13^C chemical shifts, heteronuclear constant couplings (^2,3^*J*_CH_), and interatomic distances, derived from quantitative NOE experiments. Following, the derived relative populations of the conformers found are shown in **Table S13**, and their structures in **Figure S95**, together with a bar plot showing the Akaike and the Chi-square in **Figure S96** resulting from the applied CASE analysis. It has clearly seemed that no discrimination based on NMR isotropic restrictions was observed whatsoever in both **1a** and **1b** (isotropic data are presented in Tables S1, S3, and S6). Then, we proceed to use NMR anisotropic observables, meaning ^1^*D*_CHs_. The RDCs used in the MTCM analysis were measured from a ^13^C-^1^H CLIP/CLAP-HSQC experiment (**Figure S97**),^[12,13]^ using a 3 mm compression device^[14]^ together with a poly-HEMA^[15]^ micro gel stick^[14]^ in a 1.2 GHz Bruker spectrometer, and they are presented in **Table S14**. Both CLIP/CLAP-HSQC experiments of **1a** and **1b** were recorded with a ^2^H quadrupolar coupling of 6.1 Hz. RDCs were fitted using the SVD^[16]^, diastereotopic protons assigned based on Q-factor minimization^[17]^ and conformer population derived from CASE analysis (*Vide supra*). All ^1^*D*_CH_ below the 22 C-Hs atomics pair presents in **1b** were recorded, while 20 out of 22 C-Hs atomics pair presents in **1a** were extracted. The average experimental error (σ_RDC_) of **1a** and **1b** was determined ^[18,19]^ to be 0.16 Hz and 0.18 Hz, respectively. The alignment tensor is defined by a symmetric second range tensor, thus at least 15 linearly independent RDCs are needed for fitting the set of 3 conformers shown in **Figure S95**. The study of both cosine matrixes generated during the SVD fitting revealed a rank value of 15, making the dataset suitable for discrimination with a set of conformations of up to 3 molecular geometries, which is our case. RDCs collected from **1a** spread from -17.01 Hz (H8 [1.69 ppm]) to 6.06 Hz (H14 [2.31 ppm]), implying a range of 23.07 Hz. While ^1^*D*_CHs_ from **1b** spread from -12.69 Hz (H4 [2.46 ppm]) to 10.95 Hz (H6 [2.38 ppm]), ranging as 23.64 Hz. The MTMC analysis of the RDCs collected from **1a** shows a favourable Q-factor of 0.104 for 3*S*,7*S*,11*R*-**1a** against a value of 0.287 for 3*S*,7*S*,11*R*-**1a** (**Figure S96-left**). It can be observed how the linearity increases as the MTMC analysis was employed against the STMC approaches, yet both yield indistinctly the same assignment of **1a** to the relative configuration of **3*S*,7*S*,11*R***. A similar situation was observed in the RDCs MTMC-fitting of **1b**. The fit is markedly better for **3*R*,7*S*,11*R*-1b**, for which the Q factor drops to 0.085, whereas fitting the RDCs of **1b** to **3*S*,*7S*,11*R*-1b** increases the Q factor to 0.157 (Figure S98). Details of the alignment tensors found are shown in **Table S15**. The robustness of the fitting was tested by carrying out a bootstrapping error analysis considering the experimental σ_RDC_ of both sets of data (*vide supra*). Bootstrapping analysis of both RDC analysis of **1a** and **1b** data sets is shown in **Figure S99** and **Figure S100**. The same tendency is observed in both; the epimeric discrimination is maintained. This finding demonstrates that the assignment established by STMC RDC-analysis in the determination of the configuration and conformation of flexible meroditerpens **1a** and **1b** is the same as that observed for the MTMC approximation. It is worth highlighting that the conformer selection was based on experimentally constrained molecular geometries. We do believe that assignment of chromonic meroditerpenes can be achieved regardless of the method used.

**Table S13**. Experimental populations of conformers based on experimental constraints^a^.

| **Fitted data** | **Configuration** | **Conformers population (%)** | | | **Q Factor^b^** |
| --- | --- | --- | --- | --- | --- |
| Isotropic NMR data of **1b** | 3*R*,7*S*,11*R* | **C3** | **C17** | **C39** | 0.085 |
|  |  | 3.87 | 74.61 | 21.51 |  |
|  | 3*S*,7*S*,11*R* | **C52** | **C58** | **C60** | 0.157 |
|  |  | 21.74 | 64.73 | 13.52 |  |
| Isotropic NMR data of **1a** | 3*R*,7*S*,11*R* | **C18** | **C22** | **C39** | 0.287 |
|  |  | 26.62 | 72.39 | 0.98 |  |
|  | 3*S*,7*S*,11*R* | **C14** | **C22** | **C56** | 0.104 |
|  |  | 6.98 | 59.25 | 33.75 |  |

**^a^** The populations were estimated based on the CASE approach over DFT optimized set of conformers constrained by ^1^H, ^13^C, interatomic NOE-derived distances, and *J*-BCA (^2,3^*J*_CH_). b Q-factor derived from a Multi Tensor Multi Conformer RDC-based analysis.

| Experimentally constrained conformers used in the RDC-MTMC fitting of **1a-data** | Experimentally constrained conformers used in the RDC-MTMC fitting of **1b-data** |
| --- | --- |
|  |  |
| 3*S*,7*S*,11*R*-**1a** | 3*S*,7*S*,11*R*-**1b** |
|  |  |
| 3*R*,7*S*,11*R* -**1a** | 3*R*,7*S*,11*R*-**1b** |

**Figure S95.** Conformer overlapping of geometries used in the multi tensor multi conformer analysis of the stereochemical assignment of **1a** (right) and **1b** (left). Conformers were constrained using quantitative NOEs, ^2,3^J_CH,_ and ^1^H/^13^C chemical shift.

|  |  |
| --- | --- |

**Figure S96.** Bar plots from the CASE study of **1a** and **1b**, from which conformer populations were derived. The analysis was based on isotropic NMR constraints, including quantitative NOEs, ^2,3^*J*_CH_ couplings, and ^1^H/^13^C chemical shifts. The resulting population estimates are reported in **Table S13** and were used in the MTMC analysis.

**Figure S97.** Anisotropic ^13^C-^1^H CLIP/CLAP-HSQC experiment (hsqcetgpiajcsp.2) of **1a** (i) and **1b** (ii), swollen in poly-HEMA in a 3 mm compression device, recorded in a 1.2 GHz Bruker spectrometer. Both anisotropic NMR experiments were recorded with a ^2^H quadrupolar splitting of 6.1 Hz. Spectra acquisition time was sped up by implementing NUS (512/39/97).

**Table S14.** RDCs of **1a** and **1b** measure from a Clip/Clap-HSQC experiment recorded in a 1.2 GHz Bruker spectrometer.

| **Atomic Pair** | **^1^*D*_CHs_ of 1a (Hz)** | **^1^*D*_CHs_ of 1b (Hz)** |
| --- | --- | --- |
| C5'-H5' | -13.08 | -10.91 |
| C3'-H3' | -6.38 | -11.90 |
| OMe- | 4.70 | -0.23 |
| C4-H4 (2.87 ppm) | -9.00 | 1.07 |
| C4-H4 (2.46 ppm) | -10.02 | -12.69 |
| C6-H6 (2.38 ppm) | NR | 10.95 |
| C6-H6 (2.77 ppm) | -3.46 | -3.49 |
| C14-H14 (2.31 ppm) | 6.06 | 7.36 |
| C14-H14 (2.72 ppm) | 5.34 | 0.18 |
| C1-H1 | -4.32 | -2.81 |
| Me-C6' | -0.10 | 0.20 |
| C10-H10 (1.28 ppm) | -2.43 | -3.58 |
| C10-H10 (1.85 ppm) | NR | 7.48 |
| C2-H2 | -0.01 | -1.99 |
| C9-H9 | -8.11 | -1.73 |
| Me20 | -0.35 | -0.22 |
| Me16 | 0.23 | 0.86 |
| Me17 | 0.49 | 0.31 |
| Me18 | 0.90 | 0.26 |
| Me19 | 0.36 | 0.13 |
| C8-H8 (1.69 ppm) | -17.01 | -0.23 |
| C8-H8 (1.42 ppm) | -4.91 | 7.13 |

NR: Not readable

| MTMC fitting to ^1^*D*_CHs_ measured from **1a** | MTMC fitting to ^1^*D*_CHs_ measured from **1b** |
| --- | --- |
| RDC calc. (Hz)  RDC exp. (Hz) | RDC exp. (Hz)  RDC calc. (Hz) |
|  |  |
| RDC calc. (Hz)  RDC exp. (Hz) | RDC calc. (Hz)  RDC exp. (Hz) |

**Figure S98.** Correlation plot of the experimental and back-calculated RDCs derived from the ^1^*D*_CH_-MTMC fitting of **1a** (Left column) and **1b** (Right column).

| **3*S*,7*S*,11*R*-1a** | **3*R*,7*S*,11*R*-1a** |
| --- | --- |
| (a) | (b) |

**Figure S99.** The bootstrapping error analysis for the MTMC analysis of both possible configurations: 3S,7S,11R-**1a** (a) and 3R,7S,11R-**1a** (b), using an RDC experimental error of 0.16 Hz.

| **3*R*,7*S*,11*R*-1b** | **3*S*,7*S*,11*R*-1b** |
| --- | --- |
| (a) | (b) |

**Figure S100.** The bootstrapping error analysis for the MTMC analysis of both possible configurations: 3*R*,7*S*,11*R*-**1b** (a) and 3*S*,7*S*,11*R*-**1b** (b), using an RDC experimental error of 0.18 Hz.

**Table S15**. Alignment tensors parameters of each conformer found during the MTMC analysis of 3S,7S,11R-**1a** and 3R,7S,11R-**1b**.

| **Analysis of 1a RDC data** | | | | | | | | |
| --- | --- | --- | --- | --- | --- | --- | --- | --- |
| Conf. | A_xx_ | A_yy_ | A_zz_ | A_a_ | A_r_ | Alpha | Beta | Gamma |
| 3*S*7*S*11*R*-C14 | -4.26949e-04 | -1.62792e-03 | 2.05487e-03 | 3.08230e-03 | 1.20097e-03 | 128.03 | 95.50 | -179.71 |
| 3*S*7*S*11*R*-C22 | -6.47239e-04 | -1.47263e-03 | 2.11987e-03 | 3.17980e-03 | 8.25389e-04 | 88.72 | 4.11 | 55.63 |
| 3*S*7*S*11*R*-C56 | 1.42512e-03 | 1.72424e-03 | 3.14936e-03 | 4.72405e-03 | 2.99123e-04 | 78.20 | 37.38 | 24.79 |
| **Analysis of 1b RDC data** | | | | | | | | |
| Conf. | A_xx_ | A_yy_ | A_zz_ | A_a_ | A_r_ | Alpha | Beta | Gamma |
| 3*R*7*S*11*R*-C3 | -2.38e-04 | -3.91e-04 | 6.29e-04 | 9.43e-04 | 1.53e-04 | -15.27 | 53.16 | 74.67 |
| 3*R*7*S*11*R*-C17 | 6.67e-05 | 1.06e-03 | -1.13e-03 | -1.70e-03 | -9.99e-04 | 5.08 | 101.48 | 94.34 |
| 3*R*7*S*11*R*-C39 | 4.35e-04 | 6.66e-04 | -1.10e-03 | -1.65e-03 | -2.31e-04 | 113.25 | 110.54 | -173.40 |

Conf.: Conformers. A_xx_, A_yy_, A_zz_, A_a_, A_r_ are the eigenvalues of the alignment tensor; A_a_ and A_r_ are axial and rhombic component of the alignment tensor, respectively; and Alpha, Beta, and Gamma are Euler angles in degrees for rotation of the alignment tensor into the principal axis frame. The alignment tensor was determined individually for each conformer using the SVD method.

**Conformers Geometries**

**3*S*7*S*11*R*-C14**

C -4.115653 0.511563 1.456317

C -4.818022 0.339138 0.255436

C -4.338490 -0.546332 -0.702022

C -3.145714 -1.254520 -0.478998

C -2.445229 -1.047091 0.706919

C -2.932605 -0.170296 1.697782

C -2.648455 -2.254999 -1.494962

C -1.610710 -3.175385 -0.859066

C -0.579016 -2.375496 -0.056093

O -1.257330 -1.673175 1.003265

C 0.091664 1.325608 0.387282

C 1.358677 0.545883 0.105281

C 1.381934 -0.677334 -0.485787

C 0.126831 -1.356135 -0.991988

C -2.171605 -0.004539 2.984613

O -5.960886 1.076502 0.123760

C -6.712342 0.905748 -1.065884

C -0.297384 2.480589 -0.570132

C -1.760942 2.833085 -0.312817

O 0.459054 3.658671 -0.279000

C -0.095709 2.117826 -2.041045

C 2.637008 1.193303 0.525226

C 3.845135 0.301859 0.622262

C 3.908674 -0.531221 -0.686535

C 2.676567 -1.426332 -0.763944

C 5.224884 0.967534 0.670673

C 6.178187 -0.125479 0.103018

C 5.276782 -1.214567 -0.548954

C 3.955744 0.378934 -1.930711

C 3.669521 -0.558916 1.894104

O 2.681224 2.390687 0.801593

C 0.387221 -3.305504 0.656569

H 1.314389 3.381895 0.094317

H -4.511506 1.200015 2.197874

H -4.872050 -0.719846 -1.630598

H -2.223230 -1.733904 -2.362081

H -3.492133 -2.841535 -1.871733

H -1.096907 -3.774378 -1.616785

H -2.104409 -3.867054 -0.166120

H 0.162412 1.765131 1.390742

H -0.746098 0.630583 0.417089

H -0.614029 -0.603733 -1.271403

H 0.383215 -1.896813 -1.911026

H -2.141575 -0.946018 3.542836

H -2.636818 0.757947 3.612543

H -1.132875 0.280989 2.791712

H -7.055499 -0.129727 -1.169413

H -6.124733 1.185636 -1.947330

H -7.573138 1.567365 -0.978273

H -1.918427 3.048126 0.749627

H -2.418367 2.003029 -0.595794

H -2.037075 3.718844 -0.893385

H -0.713526 1.262441 -2.333601

H 0.951640 1.874498 -2.244354

H -0.378242 2.972424 -2.663038

H 2.602311 -1.897705 -1.751870

H 2.770608 -2.252265 -0.046666

H 5.493400 1.267526 1.687316

H 5.239439 1.870993 0.054993

H 6.793902 -0.559824 0.895105

H 6.865989 0.306325 -0.628797

H 5.661011 -1.550766 -1.517059

H 5.203567 -2.097250 0.094571

H 3.083338 1.035050 -2.007757

H 3.981154 -0.247627 -2.827925

H 4.850351 1.006609 -1.942304

H 2.753136 -1.153370 1.886685

H 3.633447 0.102057 2.765788

H 4.521011 -1.232196 2.025698

H 1.006239 -3.843289 -0.067091

H -0.173261 -4.033627 1.248973

H 1.035114 -2.739593 1.332337

**3*S*7*S*11*R*-C22:**

72

#1

C -5.303819 0.034165 0.939314

C -5.454721 0.227168 -0.439881

C -4.354169 0.111302 -1.290939

C -3.102484 -0.184381 -0.744398

C -2.972532 -0.380912 0.625732

C -4.066145 -0.280851 1.494346

C -1.833883 -0.341306 -1.533205

C -1.112966 -1.614147 -1.067369

C -0.977121 -1.692735 0.476314

O -1.720290 -0.634645 1.135023

C 2.773225 -1.724828 -1.016587

C 2.289277 -0.424971 -0.403793

C 1.230528 -0.316645 0.442814

C 0.476099 -1.522382 0.954867

C -3.884457 -0.509312 2.970098

O -6.720976 0.518674 -0.861556

C -6.914413 0.741058 -2.248476

C 4.032923 -2.395759 -0.399133

C 4.027278 -3.877174 -0.763428

O 5.222423 -1.858850 -0.981075

C 4.093439 -2.218809 1.117878

C 3.074191 0.793853 -0.769878

C 2.414110 2.123551 -0.526648

C 1.829309 2.100652 0.911507

C 0.759833 1.018059 0.998702

C 3.294775 3.377799 -0.516771

C 2.487972 4.381121 0.359790

C 1.383190 3.559232 1.085914

C 2.922829 1.813859 1.960500

C 1.340113 2.283161 -1.626704

O 4.194299 0.699578 -1.267774

C -1.517645 -3.023492 0.996914

H 5.075956 -0.905294 -1.110387

H -6.181550 0.123495 1.573490

H -4.444408 0.251107 -2.362517

H -1.193893 0.531481 -1.356197

H -2.034338 -0.385027 -2.606871

H -0.123090 -1.675802 -1.531850

H -1.687019 -2.478118 -1.418322

H 2.998402 -1.552520 -2.076464

H 1.961600 -2.454840 -0.997445

H 1.000486 -2.448285 0.711404

H 0.443372 -1.457690 2.050500

H -3.220811 0.244231 3.405536

H -3.423887 -1.484884 3.154416

H -4.843562 -0.468627 3.490283

H -6.656899 -0.150201 -2.831140

H -6.317628 1.591134 -2.597332

H -7.973137 0.963684 -2.374264

H 3.920970 -4.001611 -1.845849

H 3.202622 -4.397184 -0.266607

H 4.969388 -4.337043 -0.450390

H 4.943026 -2.780075 1.518131

H 3.178756 -2.576400 1.602590

H 4.226889 -1.163759 1.375429

H 0.430364 0.871331 2.033961

H -0.143907 1.320853 0.452250

H 3.472581 3.756222 -1.527065

H 4.272691 3.160740 -0.078232

H 2.041911 5.167857 -0.254433

H 3.145536 4.877607 1.078389

H 1.290065 3.828670 2.142695

H 0.404064 3.722670 0.624003

H 3.414057 0.848634 1.806010

H 2.462790 1.795484 2.953816

H 3.694839 2.587728 1.969551

H 0.640306 1.445794 -1.653321

H 1.836244 2.335530 -2.600957

H 0.775209 3.209330 -1.490435

H -1.015056 -3.861707 0.505140

H -2.589576 -3.094788 0.783159

H -1.365420 -3.101428 2.077507

**3*S*7*S*11*R*-C56:**

C -5.259361 0.894178 -1.051649

C -5.746721 -0.341860 -0.608275

C -5.035668 -1.076520 0.341881

C -3.846326 -0.553317 0.857279

C -3.373126 0.672641 0.400950

C -4.065298 1.417681 -0.562871

C -2.957141 -1.255085 1.843857

C -1.522123 -1.136033 1.315138

C -1.094597 0.339176 1.183539

O -2.238959 1.206503 0.961392

C 2.373593 2.044447 0.733418

C 2.322325 0.591328 0.304821

C 1.183020 -0.076481 -0.018808

C -0.171934 0.596459 -0.035258

C -3.519084 2.739088 -1.030857

O -6.923180 -0.750748 -1.170371

C -7.451835 -1.996561 -0.748405

C 2.877392 3.091349 -0.299368

C 2.374295 4.469513 0.120166

O 4.303136 3.177251 -0.278959

C 2.414552 2.765116 -1.719103

C 3.636009 -0.119256 0.262978

C 3.591444 -1.622468 0.251426

C 2.562146 -2.049001 -0.828795

C 1.183558 -1.543520 -0.422731

C 4.859097 -2.375106 -0.169887

C 4.315555 -3.735387 -0.698876

C 2.777536 -3.568459 -0.874527

C 2.923475 -1.477097 -2.213811

C 3.206776 -2.067414 1.681331

O 4.696466 0.502690 0.273511

C -0.478430 0.840176 2.482862

H 4.644280 2.275113 -0.145896

H -5.830182 1.439125 -1.798429

H -5.382921 -2.041376 0.694756

H -3.242635 -2.304001 1.956795

H -3.035933 -0.782131 2.831564

H -1.484757 -1.632658 0.339723

H -0.815597 -1.649352 1.975870

H 3.023163 2.131704 1.613807

H 1.381067 2.354456 1.060276

H -0.070842 1.680570 -0.118545

H -0.720538 0.259675 -0.924947

H -2.489007 2.627607 -1.383701

H -3.498593 3.466774 -0.213381

H -4.127113 3.145485 -1.841571

H -6.762223 -2.816100 -0.979305

H -8.379460 -2.134957 -1.302239

H -7.665151 -1.990454 0.326242

H 2.648373 4.674181 1.160056

H 1.286057 4.533096 0.024464

H 2.826382 5.235802 -0.516608

H 1.327296 2.642589 -1.770340

H 2.880742 1.840394 -2.072567

H 2.706541 3.575305 -2.394062

H 0.462514 -1.684829 -1.237562

H 0.804997 -2.139043 0.418775

H 5.553089 -2.499407 0.665867

H 5.392287 -1.826634 -0.951094

H 4.537456 -4.546096 0.000116

H 4.793517 -3.997010 -1.646630

H 2.414391 -4.000768 -1.812136

H 2.235213 -4.059732 -0.060090

H 2.948215 -0.383502 -2.225926

H 2.167433 -1.799650 -2.936895

H 3.891814 -1.840292 -2.567312

H 2.247554 -1.663055 2.013997

H 3.976887 -1.721348 2.377973

H 3.167053 -3.157966 1.748474

H -1.130441 0.596380 3.326868

H -0.353168 1.926050 2.449262

H 0.497755 0.374964 2.651216

**3*R*7*S*11*R*-C3:**

C -4.160245 -0.389808 1.184949

C -4.809589 -0.165740 -0.033104

C -4.276907 0.748075 -0.940280

C -3.088948 1.425584 -0.665200

C -2.430410 1.163645 0.542559

C -2.969253 0.274549 1.483650

C -2.528289 2.440578 -1.634118

C -1.475180 3.303971 -0.943046

C -0.498658 2.446608 -0.129824

O -1.236116 1.746822 0.890370

C 0.007029 -1.286772 0.298730

C 1.301252 -0.555613 0.045537

C 1.397186 0.670063 -0.528356

C 0.200372 1.423308 -1.066981

C -2.262402 0.058480 2.794448

O -5.969196 -0.784189 -0.409255

C -6.497417 -1.769702 0.462583

C -0.340224 -2.406920 -0.706178

C -0.607128 -1.855771 -2.101497

O 0.756918 -3.314668 -0.855304

C -1.567844 -3.159968 -0.191956

C 2.533467 -1.274458 0.505536

C 3.846301 -0.752786 -0.016853

C 3.846375 0.791976 0.102281

C 2.732092 1.354374 -0.770756

C 5.127598 -1.152058 0.723700

C 6.130466 -0.020753 0.350422

C 5.294517 1.134852 -0.275606

C 3.621643 1.240204 1.561314

C 3.945640 -1.260366 -1.475648

O 2.462406 -2.271057 1.218386

C 0.471503 3.325287 0.642082

H 1.162483 -3.451650 0.013604

H -4.561079 -1.083898 1.915534

H -4.803928 0.933474 -1.872704

H -3.340011 3.067116 -2.016245

H -2.096782 1.929301 -2.503949

H -1.962497 3.997916 -0.247617

H -0.916893 3.901109 -1.670228

H -0.831415 -0.591914 0.321221

H 0.065851 -1.742525 1.295078

H 0.532457 1.981738 -1.952364

H -0.559557 0.718669 -1.411093

H -1.212080 -0.204296 2.636302

H -2.743708 -0.737377 3.366617

H -2.271592 0.973222 3.396488

H -5.776263 -2.579109 0.622665

H -7.385432 -2.164286 -0.029532

H -6.778935 -1.334436 1.427875

H -0.782609 -2.683810 -2.794927

H 0.250201 -1.277446 -2.461207

H -1.496234 -1.215507 -2.097636

H -1.356059 -3.617069 0.780796

H -1.845770 -3.950824 -0.895427

H -2.418368 -2.478439 -0.073533

H 2.623940 2.433685 -0.619243

H 2.976051 1.231111 -1.836911

H 5.482133 -2.140214 0.418011

H 4.953211 -1.192361 1.802602

H 6.880100 -0.381225 -0.359031

H 6.673361 0.320274 1.235974

H 5.590224 2.117315 0.105745

H 5.414926 1.161465 -1.363593

H 2.677285 0.872754 1.975824

H 4.430029 0.907322 2.217493

H 3.601173 2.334648 1.598805

H 3.101998 -0.947520 -2.095958

H 3.964555 -2.354629 -1.464232

H 4.872172 -0.915303 -1.942854

H -0.092809 4.006793 1.284442

H 1.083260 3.919207 -0.042114

H 1.126832 2.716431 1.271442

**3*R*7*S*11*R*-C17:**

72

#1

C 4.314066 1.220787 0.313924

C 4.917421 0.304384 -0.559013

C 4.375980 -0.966942 -0.703263

C 3.213248 -1.326270 -0.001534

C 2.599853 -0.386673 0.823541

C 3.163719 0.891766 1.015805

C 2.663446 -2.729683 -0.107112

C 1.654756 -2.996356 1.007557

C 0.692047 -1.818556 1.192637

O 1.450858 -0.642086 1.532302

C -3.104740 -1.651835 0.477126

C -2.315903 -0.388205 0.221979

C -0.997769 -0.360797 -0.109113

C -0.119718 -1.591363 -0.113492

C 2.548107 1.846892 2.003621

O 6.039329 0.746249 -1.202164

C 6.693818 -0.165258 -2.068013

C -3.743189 -2.278615 -0.787498

C -4.786854 -1.367255 -1.430117

O -2.725834 -2.585544 -1.748312

C -4.365193 -3.619386 -0.417550

C -3.067023 0.897149 0.403689

C -2.233299 2.156188 0.411980

C -1.219397 2.082457 -0.756034

C -0.275768 0.912261 -0.510257

C -2.952333 3.487657 0.163226

C -1.827538 4.413687 -0.387654

C -0.619109 3.495842 -0.735951

C -1.931147 1.891068 -2.110244

C -1.555643 2.221230 1.801943

O -4.270282 0.908211 0.627025

C -0.207004 -2.049454 2.397240

H -2.315107 -1.756265 -2.031852

H 4.771715 2.197851 0.441840

H 4.839268 -1.710184 -1.343865

H 3.488580 -3.445980 -0.041781

H 2.204592 -2.882738 -1.091248

H 2.181732 -3.140142 1.958446

H 1.082012 -3.906731 0.806518

H -3.908765 -1.430851 1.186121

H -2.469101 -2.414896 0.931068

H -0.702439 -2.499021 -0.291119

H 0.584277 -1.497067 -0.947053

H 3.125380 2.772234 2.053427

H 2.514736 1.400104 3.002454

H 1.515680 2.098542 1.740853

H 7.032103 -1.053132 -1.522261

H 7.556160 0.364521 -2.470754

H 6.035401 -0.470526 -2.889068

H -4.344120 -0.419251 -1.752953

H -5.587504 -1.134563 -0.723215

H -5.212727 -1.865439 -2.306262

H -5.147612 -3.482916 0.334157

H -3.603773 -4.292876 -0.012712

H -4.808657 -4.084920 -1.302658

H 0.320457 0.694389 -1.407071

H 0.451589 1.151898 0.272989

H -3.404512 3.880676 1.078037

H -3.758924 3.361475 -0.564673

H -1.539365 5.165850 0.351691

H -2.174744 4.957549 -1.270287

H -0.159269 3.756175 -1.694628

H 0.162540 3.572535 0.027609

H -2.599238 2.724667 -2.342067

H -2.518483 0.968744 -2.150963

H -1.178823 1.841024 -2.904033

H -0.907264 1.364089 2.004011

H -2.332059 2.248229 2.573266

H -0.963130 3.136245 1.899051

H -0.905788 -1.218611 2.533038

H 0.409512 -2.138558 3.295813

H -0.775888 -2.975101 2.274773

**3*R*7*S*11*R*-C39:**

72

#1

C 4.925916 1.531062 0.066364

C 5.755981 0.413470 0.234179

C 5.220286 -0.865018 0.134274

C 3.852819 -1.040295 -0.134277

C 3.042986 0.080808 -0.290473

C 3.571412 1.382783 -0.192626

C 3.264057 -2.425753 -0.253753

C 1.894709 -2.374445 -0.925886

C 1.034598 -1.246974 -0.341252

O 1.702239 0.006789 -0.577054

C -3.241316 -1.909803 -0.921586

C -2.659250 -0.564422 -0.550990

C -1.350852 -0.211631 -0.658606

C -0.291474 -1.189307 -1.122061

C 2.659708 2.566185 -0.373452

O 7.072011 0.683361 0.486686

C 7.942083 -0.422645 0.654936

C -3.588894 -2.839999 0.262224

C -2.345432 -3.241259 1.043248

O -4.424675 -2.161528 1.205727

C -4.319710 -4.074130 -0.266057

C -3.678251 0.452424 -0.127636

C -3.141346 1.718093 0.484955

C -1.997280 2.230303 -0.425841

C -0.866423 1.210751 -0.433792

C -4.079216 2.927559 0.575253

C -3.107074 4.144481 0.594467

C -1.704155 3.605887 0.189323

C -2.485978 2.448394 -1.872269

C -2.674997 1.350842 1.912944

O -4.881745 0.238667 -0.241640

C 0.824841 -1.427445 1.162100

H -5.036397 -1.588059 0.720726

H 5.363808 2.522243 0.146371

H 5.838251 -1.749198 0.251201

H 3.179694 -2.877533 0.742813

H 3.941492 -3.066310 -0.827095

H 1.372874 -3.329424 -0.809698

H 2.012582 -2.187221 -2.000159

H -2.584246 -2.455047 -1.602538

H -4.168926 -1.721831 -1.476017

H -0.686117 -2.206709 -1.126743

H -0.033695 -0.937227 -2.161070

H 3.210827 3.502037 -0.261405

H 1.846317 2.545650 0.359493

H 2.191363 2.551400 -1.363360

H 7.967199 -1.044744 -0.246668

H 7.640230 -1.034678 1.512254

H 8.931483 -0.004693 0.836061

H -1.840334 -2.349260 1.424430

H -1.649366 -3.804090 0.412396

H -2.628000 -3.869888 1.893091

H -4.584582 -4.732713 0.566435

H -3.692312 -4.633681 -0.967305

H -5.239664 -3.781039 -0.782663

H -0.132575 1.447152 -1.212584

H -0.297723 1.249321 0.505729

H -4.710746 2.884672 1.466997

H -4.747876 2.963174 -0.289291

H -3.071683 4.604803 1.585566

H -3.446817 4.917731 -0.099859

H -1.190185 4.266257 -0.516770

H -1.055967 3.503573 1.066404

H -1.649695 2.815822 -2.475881

H -3.283870 3.193657 -1.928491

H -2.850551 1.525988 -2.335329

H -1.913463 0.567342 1.924090

H -3.536078 0.989494 2.484332

H -2.279393 2.230615 2.428329

H 0.096209 -0.709228 1.548596

H 1.764753 -1.278346 1.700669

H 0.464049 -2.437831 1.374922

**Monte carlo error analysis for the epimeric differentiation of 1a and 1b by ^13^C-RCSA**

The QCSA Monte Carlo simulation was performed by incorporating the standard deviation (σ) of each component in the QCSA equation (Equation 1), using a random sampling size of 500,000 trials. The simulation accounts for potential variations in the parameters ΔRCSAi,ax_exp ( σΔRCSAi,ax_exp: σ1a = 0.58 ppb and σ1b = 0.66 ppb), ΔRCSAi,ax_theo, and CSAi,ax (SEE = 5.91_8_ ppm). Each component was assumed—albeit simplistically—to follow a normal distribution within a ±3σ range, thereby encompassing approximately 99.7% of the data according to the empirical rule. For simplicity, ΔRCSAi,ax_theo was considered to have the same standard deviation as ΔRCSAi,ax_exp in each case.

The quality of discrimination was evaluated using statistical descriptors, namely the Overlap Coefficient^[20]^ (OVL) and the Kolmogorov–Smirnov (K–S) statistic.^[21]^ Results are reported as the mean QCSA value, along with its standard deviation and 95% confidence interval.

**Monte Carlo simulation (MCS) results for the ^13^C-RCSA data of compound 1a are shown in Figure S101.** The simulation reveals that the resulting distributions exhibit minimal overlap (6.2%), indicating a clear separation between the two datasets. The QCSA value for *SSR*-**1a** is 0.143 ± 0.022, while for its epimer *RSR*-**1a**, it is 0.246 ± 0.030. The Kolmogorov–Smirnov (K–S) statistic further supports this distinction, with a D-value of 0.9893 and a p-value < 0.0000. These results confirm that the two simulated datasets originate from statistically distinct distributions, thereby demonstrating successful structural discrimination between epimers using ^13^C-RCSA.

**The MCS results for compound 1b are presented in Figure S102.** In this case, the distributions show a higher degree of overlap (26.1%), suggesting reduced discriminative power compared to **1a**. Literature names this degree of overlap as acceptable.^[20]^ This can be attributed to the lower magnitude of the axial component Aa of the alignment tensor, which is on the order of 10⁻⁴ in 1b, compared to 10⁻³ in **1a**—making the dataset more susceptible to error. Nonetheless, the application of the Kolmogorov–Smirnov test yields a D-value of 0.7474 with a p-value < 0.0000, confirming that the datasets arise from significantly different distributions. This result validates the structural discrimination even in the presence of higher uncertainty.

**Figure S101.** Monte Carlo Error Propagation Analysis in the ^13^C-RCSA-Based Discrimination of **1a**-SSR/RSR Epimers. The simulation was performed by independently propagating the uncertainty associated with each variable in the QCSA equation, assuming Gaussian error distributions. A total of 500,000 random samples were generated within a ±3σ range to cover 99.7% of the expected variation. The resulting plot displays the mean QCSA value, its standard deviation, and the corresponding 95% confidence interval.

**Figure S102.** Monte Carlo Error Propagation Analysis in the ^13^C-RCSA-Based Discrimination of **1b**-SSR/RSR Epimers. The simulation was performed by independently propagating the uncertainty associated with each variable in the QCSA equation, assuming Gaussian error distributions. A total of 500,000 random samples were generated within a ±3σ range to cover 99.7% of the expected variation. The resulting plot displays the mean QCSA value, its standard deviation, and the corresponding 95% confidence interval.

Equation 1

$$Q_{CSA}=\sqrt{\frac{\sum_{i} \left( ({\Delta RCSA}_{i,ax}^{exp}-{\Delta RCSA}_{i}^{theo})/{CSA}_{i,ax} \right)^{2}}{\sum_{i} \left( {\Delta RCSA}_{i}^{exp}/{CSA}_{i,ax} \right)^{2}}}$$

**Determination of the σΔRCSAi,ax_exp:**

The estimation of the standard deviation associated with the experimental ΔRCSAi,ax values was performed using the error analysis module integrated in *MSpin*. In this module, the experimental uncertainty is introduced as a standard deviation derived from the experimentally recorded RCSA data. The estimation was carried out using Equation 3, where two primary sources of error are considered:

σ_exp-1_: accounts for the uncertainty arising from signal quality. Broad peaks or low signal-to-noise ratios reduce the precision of the chemical shift measurements.

σ_exp-2_: captures the uncertainty due to frequency domain resolution, specifically the number of digital points per unit of frequency, which influences the accuracy of peak position determination.

Equation 3

$${\sigma RCSA}^{exp}=\sqrt{{\sigma_{exp-1}}^{2}+{\sigma_{exp-2}}^{2}}$$

Equation 4

$$\sigma_{exp-1}\left( ppb \right)= \sum_{i=1}^{n} \frac{\frac{LW1/2}{SINO}*B_{0}*1000}{n}$$

Equation 5

$$\sigma_{exp-2}\left( ppb \right)= \frac{SW}{FDIRES}*B_{0}*1000$$

LW1/2: Peak width at half maximum (in Hz); represents the full width of the peak at 50% of its maximum intensity. SINO: ^13^C: Signal-to-noise ratio of the ^13^C NMR signal; reflects the quality of the acquired spectrum. SW: Spectral width (in ppm); defines the total frequency range captured during acquisition. B0: Operating magnetic field strength for ^13^C nuclei (in MHz); determines the Larmor frequency and FDIRES: Frequency domain digital resolution (in Hz/point); represents the number of data points per unit of line width in the frequency domain, affecting peak definition.

**σ(CSA_i,ax_):**

The uncertainty associated with CSAi,ax was estimated based on experimental deviations reported by Holmes et al.^[22]^ and Veeman.^[23]^ This approach provides a more representative assessment of the real error involved in **CSA_i,ax_** estimations, compared to evaluating deviations between DFT calculations performed at different levels of theory.

Experimentally determined values were compared with their DFT-calculated counterparts using the **mPW1PW91/6-311+G(2d,p)** level of theory within the **IEFPCM (DMSO)** solvation model, which is consistent with the methodology employed for the estimation of CST tensors used in the ¹³C-RCSA fitting protocol.

A total of **29 axial CSA components** derived from **13 distinct molecular structures** were analysed (**Figure S103**). The correlation between experimental and theoretical values showed a coefficient of determination R² = 0.9845. The correlation plot is shown in **Figure S104**. The standard error of the estimated CSA (SEE) was 5.91_8_ ppm.

Initial molecular geometries were obtained from **PubChem** (<https://pubchem.ncbi.nlm.nih.gov/>) and subjected to a **conformational search** using the **ETKDG** algorithm,^[24]^ as implemented in **RDKit**. The resulting conformers were energy-ranked, and the **lowest-energy structures** were selected for further quantum chemical calculations. Geometry optimizations and NMR parameter calculations were carried out using a composite DFT protocol, specifically: GIAO mPW1PW91/6-311+G(2d,p) IEFPCM = DMSO // B3LYP/6-31G(d,p). This protocol ensures consistency with the level of theory used for estimating the chemical shift tensors (CSTs) employed in the ¹³C-RCSA fitting procedures.

**Figure S103.** Molecular structures of the compounds used to determine the absolute error and standard deviation associated with the axial chemical shift anisotropy.

**Figure S104.** Correlation plot between the absolute value of the experimental and computed axial chemical shift anisotropy of several carbon atoms (│CSAax,i^cacl.^│). Theoretical CSAax values were computed at DFT level using mPW1PW91/6311+(2d,p) iefpcm = dmso.

**Table S16**. Derived fitting parameters for the spin-spin couplings (^2,3^J_CH_ and ^3^J_HH_)

| **Parameter** | **Slope** | **Coefficient correlation** | **Standard deviation (Hz)** | **Number of spin-spin coupling fitted** |
| --- | --- | --- | --- | --- |
| ^3^*J*_HH_ | 0.8841 | 0.991_5_ | 3.0_2_ | 25 |
| ^2^*J*_CH_ | 0.9906 | 0.991_8_ | 1.4_0_ | 20 |
| ^3^*J*_CH_ | 0.9212 | 0.996_0_ | 2.4_1_ | 28 |

Spin-spin coupling were computed at mpw1pw91/6-311+g(2d,p) iefpcm=DMSO

**Figure S105.** Individual ECD spectra of the derived conformers from anisotropic data were used to absolute configuration of **1a.**

**References**

[1] F. Mohamadi, N. G. J. Richards, W. C. Guida, R. Liskamp, M. Lipton, C. Caufield, G. Chang, T. Hendrickson, W. C. Still, “Macromodel—an integrated software system for modeling organic and bioorganic molecules using molecular mechanics” *J. Comput. Chem.* 1990, *11*, 440–467.

[2] K. S. Watts, P. Dalal, A. J. Tebben, D. L. Cheney, J. C. Shelley, “Macrocycle Conformational Sampling with MacroModel” *J. Chem. Inf. Model.* 2014, *54*, 2680–2696.

[3] Y. Zhao, D. G. Truhlar, “The M06 suite of density functionals for main group thermochemistry, thermochemical kinetics, noncovalent interactions, excited states, and transition elements: two new functionals and systematic testing of four M06-class functionals and 12 other functionals” *Theor. Chem. Acc.* 2008, *120*, 215–241.

[4] C. Adamo, V. Barone, “Exchange functionals with improved long-range behavior and adiabatic connection methods without adjustable parameters: The mPW and mPW1PW models” *J. Chem. Phys.* 1998, *108*, 664–675.

[5] P. J. Rousseeuw, “Silhouettes: A graphical aid to the interpretation and validation of cluster analysis” *J. Comput. Appl. Math.* 1987, *20*, 53–65.

[6] L. F. Gil-Silva, R. Santamaría-Fernández, A. Navarro-Vázquez, R. R. Gil, “Collection of NMR Scalar and Residual Dipolar Couplings Using a Single Experiment” *Chemistry – A European Journal* 2015, *22*, 472–476.

[7] N. Nath, M. Schmidt, A. Navarro-Vazquez, R. R Gil, C. Griesinger, M. Nath, R. R Schmidt, R. T Gil, G. E Williamson, A. Martin, C. Navarro-Vázquez, Y. Griesinger, J. Liu, *Determination of configuration of small molecules from residual chemical shift anisotropy (RCSAs) at microgram levels CDCl 3 compatible Poly(methyl methacrylate)(PMMA) gel : Different alignment conditions could be induced by moving the piston of the New E*, 2016.

[8] N. Nath, M. Schmidt, R. R. Gil, R. T. Williamson, G. E. Martin, A. Navarro-Vázquez, C. Griesinger, Y. Liu, “Determination of Relative Configuration from Residual Chemical Shift Anisotropy” *J. Am. Chem. Soc.* 2016, *138*, 9548–9556.

[9] F. Hallwass, R. R. Teles, E. Hellemann, C. Griesinger, R. R. Gil, A. Navarro-Vázquez, “Measurement of residual chemical shift anisotropies in compressed polymethylmethacrylate gels. Automatic compensation of gel isotropic shift contribution” *Magn Reson Chem.* 2018, *56*, 321–328.

[10] H. Sun, U. M. Reinscheid, E. L. Whitson, E. J. d’Auvergne, C. M. Ireland, A. Navarro-Vázquez, C. Griesinger, “Challenge of Large-Scale Motion for Residual Dipolar Coupling Based Analysis of Configuration: The Case of Fibrosterol Sulfate A” *J. Am. Chem. Soc.* 2011, *133*, 14629–14636.

[11] A. Navarro-Vázquez, R. R. Gil, K. Blinov, “Computer-Assisted 3D Structure Elucidation (CASE-3D) of Natural Products Combining Isotropic and Anisotropic NMR Parameters” *J. Nat. Prod.* 2018, *81*, 203–210.

[12] C. M. Thiele, W. Bermel, “Speeding up the measurement of one-bond scalar (1J) and residual dipolar couplings (1D) by using non-uniform sampling (NUS)” *J. Magn. Reson.* 2012, *216*, 134–143.

[13] A. Enthart, J. C. Freudenberger, J. Furrer, H. Kessler, B. Luy, “The CLIP/CLAP-HSQC: Pure absorptive spectra for the measurement of one-bond couplings” *J. Magn. Reson.* 2008, *192*, 314–322.

[14] J. C. C. Fuentes-Monteverde, N. Nath, A. M. Forero, E. M. Balboa, A. Navarro-Vázquez, C. Griesinger, C. Jiménez, J. Rodríguez, “Connection of Isolated Stereoclusters by Combining 13C-RCSA, RDC, and J-Based Configurational Analyses and Structural Revision of a Tetraprenyltoluquinol Chromane Meroterpenoid from Sargassum muticum” *Mar. Drugs* 2022, *20*, 462.

[15] L. F. Gil‐Silva, R. Santamaría‐Fernández, A. Navarro‐Vázquez, R. R. Gil, “Collection of NMR Scalar and Residual Dipolar Couplings Using a Single Experiment” *Chem. Eur. J.* 2016, *22*, 472–476.

[16] J. A. Losonczi, M. Andrec, M. W. F. Fischer, J. H. Prestegard, “Order Matrix Analysis of Residual Dipolar Couplings Using Singular Value Decomposition” *J. Magn. Reson.* 1999, *138*, 334–342.

[17] C. M. Thiele, “Simultaneous Assignment of All Diastereotopic Protons in Strychnine Using RDCs:  PELG as Alignment Medium for Organic Molecules” *J. Org. Chem.* 2004, *69*, 7403–7413.

[18] N. Tjandra, A. Bax, “Direct Measurement of Distances and Angles in Biomolecules by NMR in a Dilute Liquid Crystalline Medium” *Science (1979).* 1997, *278*, 1111–1114.

[19] G. Kontaxis, G. M. Clore, A. Bax, “Evaluation of Cross-Correlation Effects and Measurement of One-Bond Couplings in Proteins with Short Transverse Relaxation Times” *J. Magn. Reson.* 2000, *143*, 184–196.

[20] H. F. Inman, E. L. Bradley, “The overlapping coefficient as a measure of agreement between probability distributions and point estimation of the overlap of two normal densities” *Commun. Stat. - Theory Methods.* 1989, *18*, 3851–3874.

[21] F. J. Massey, “The Kolmogorov-Smirnov Test for Goodness of Fit” *J. Am. Stat. Assoc.* 1951, *46*, 68.

[22] S. T. Holmes, C. M. Boley, A. Dewicki, Z. T. Gardner, C. S. Vojvodin, R. J. Iuliucci, R. W. Schurko, “Carbon‐13 chemical shift tensor measurements for nitrogen‐dense compounds” *Magn. Reson. Chem.* 2024, *62*, 179–189.

[23] W. S. Veeman, “Carbon-13 chemical shift anisotropy” *Prog. Nucl. Magn. Reson. Spectrosc.* 1984, *16*, 193–235.

[24] S. Riniker, G. A. Landrum, “Better Informed Distance Geometry: Using What We Know To Improve Conformation Generation” *J. Chem. Inf. Model.* 2015, *55*, 2562–2574.

1. No prior structural information was provided in the initial CS regarding the chromane torsion. The input geometry used for the conformational search (seed structure) did not include any prior assumptions about the experimentally known torsion in the chromane moiety. As a result, Maestro-Schrödinger generated a conformational pool covering both existing helicities (M and P). It is worth noting that other geometries might require more than one seed structure to generate a significant conformational pool that captures all the peculiarities of their geometries. [↑](#footnote-ref-1)
